# Causality between urate levels with sarcopenia-related traits: a bi-directional Mendelian randomization study

**DOI:** 10.3389/fendo.2023.1252968

**Published:** 2023-10-26

**Authors:** Yanze Lin, Xun Wang, Wenchao Yao, Yuanting Sun, Jinlei Zhou, Fabo Feng

**Affiliations:** ^1^ Center for Plastic & Reconstructive Surgery, Department of Orthopedics, Zhejiang Provincial People’s Hospital (Affiliated People’s Hospital, Hangzhou Medical College), Hangzhou, Zhejiang, China; ^2^ Second Clinical Medical College, Zhejiang Chinese Medical University, Hangzhou, Zhejiang, China; ^3^ Department of Orthopaedics, the First People’s Hospital of Chun’an County, Hangzhou, Zhejiang, China; ^4^ Graduate School, Hunan University of Chinese Medicine, Changsha, China

**Keywords:** urate levels, hand grip strength, LEAN MASS, sarcopenia, causal relationship, mendelian randomization

## Abstract

**Background:**

Observational studies have suggested associations between serum urate levels and sarcopenia, but the causality underlying this correlation remains uncertain. The principal objective of this study is to investigate a causal relationship of serum urate levels with sarcopenia-related traits (hand grip strength, lean mass, walking pace) using bidirectional two-sample Mendelian randomization (MR) approach. The utilization of MR methodology serves to minimize bias caused by reverse causality and confounding factors from observational studies.

**Methods:**

The summary statistics of serum urate levels were derived from a cohort consisting of 288,659 individuals participating in CKDGen study. The parameters of right-hand grip strength (N=461,089), left-hand grip strength (N=461,026), appendicular lean mass (ALM) (N=450,243), whole-body lean mass(N=454,850),right-leg fat-free mass(FFM;N=454,835),left-leg FFM(N=454,805), right-arm FFM(N=454,753),left-arm FFM(N=454,672) and walking pace (N=459,915)were sourced from the UK Biobank. MR analysis was conducted utilizing inverse variance weighted (IVW), weighted median, and MR-Egger to evaluate causality. Sensitivity analysis was performed using Cochran’s Q test, MR-Egger intercept test, leave-one-out analysis and the funnel plot.

**Results:**

IVW estimates demonstrated that serum urate levels exhibited no causal association with sarcopenia-related traits. In the inverse MR investigation, we had exclusively discerned an inverse correlation between walking pace and serum urate levels. No compelling evidence had surfaced to substantiate any association of other sarcopenia-related traits with serum urate. Supplemental MR methods consistently validated the findings obtained from the primary analysis. Sensitivity analysis demonstrated the robustness of findings.

**Conclusion:**

Our MR study revealed the absence of the bidirectional causal relationship between serum urate levels and sarcopenia. It is imperative to acknowledge that advanced age and an individual’s health status are pivotal determinants influencing urate level and the initiation and advancement of sarcopenia. However, it is worth underscoring that these aspects remain unexamined within the purview of this study. Thus, future investigations should delve deeper into these intricate facets.

## Introduction

Sarcopenia, a progressive and prevalent skeletal muscle disorder, is intricately associated with a range of adverse outcomes encompassing falls, functional deterioration, frailty, and mortality (1). As expounded by the European Working Group on Sarcopenia in Older People (EWGSOP)-2, sarcopenia manifests as a diminution in muscular strength accompanied by a reduction in muscle mass, the severity of which determines the extent of functional impairment (2). Statistical data reveal that sarcopenia currently affects approximately 10% to 16% of the elderly population worldwide (3). However, the prevalence of this condition is projected to soar over the course of the next four decades, impacting over 200 million individuals globally (4). Of particular note, elderly patients admitted to hospitals who are afflicted by sarcopenia incur hospitalization expenses that exceed fivefold compared to those without sarcopenia, exacting a considerable toll on the healthcare system (5).

Urate, the ultimate byproduct arising from the metabolic breakdown of purines, exerts both potentially beneficial and detrimental consequences (6). Substantiated investigations have established that uric acid exhibits pro-inflammatory properties, intricately associated with the initiation and progression of a multitude of ailments, including chronic kidney disease, cardiovascular disease, hypertension, diabetes, and metabolic syndrome (7). Nevertheless, urate assumes a pivotal role as an antioxidant, effectively neutralizing oxygen free radicals, manifesting as a crucial combatant against the perils of oxidative stress (8). Low levels of serum uric acid have been associated with various adverse health outcomes, including increased all-cause mortality and the progression of neurodegenerative diseases like Parkinson’s, Alzheimer’s, and amyotrophic lateral sclerosis (9).

With advancing age, the capacity of human musculature to regulate Reactive Oxygen Species (ROS) levels gradually wanes, compromising the homeostasis of intracellular milieu and instigating an escalated state of oxidative stress (10). Mounting evidence underscores the crucial role of augmented oxidative stress and inflammation in the pathogenesis of muscle loss and functional decline (11, 12). However, the relationship between uric acid and sarcopenia remains obscured, as uncertainties persist regarding the intricate interplay between antioxidant stress and inflammatory mechanisms. A retrospective study unveiled a noteworthy correlation between hyperuricemia and elevated muscle mass and strength, suggesting a conceivable protective effect of uric acid against sarcopenia (13). Furthermore, an investigation focusing on renal transplant patients divulged a positive association between serum urate levels and both muscle mass and strength (14). In the western region of China, a cross-sectional study conducted among the adult population aged over 50 years revealed the correlation between elevated uric acid levels and augmented muscle mass as well as grip strength, confirming that the potential role of heightened serum urate levels in impeding the progression of sarcopenia (15). However, the cross-sectional study conducted in the United States reported that participants categorized within the highest group of serum uric acid concentration (>8 mg/dL) faced twice the likelihood of developing sarcopenia compared to those in the lowest concentration group (<6 mg/dL), implicating that hyperuricemia serves as an independent risk factor associated with sarcopenia (16). Nevertheless, it is important to note that observational studies inherently possess certain limitations that prevent them from eliminating the impact of reverse causality and confounding factors, which possibly introduce biased conclusions (17).

Mendelian randomization (MR) analysis is a method rooted in Mendel’s laws of inheritance that have gained increasing prominence in establishing plausible causal associations between risk factors and disease outcomes (18). This analytical approach capitalizes on the random classification properties of genetic variants and leverages these variants associated with environmental exposures as instrumental variables (IVs) to assess the associations between exposures and outcomes (19). By virtue of genetic variants being randomly assigned during conception prior to disease onset, MR analysis effectively circumvents the influence of confounding factors, measurement errors, and reverse causality, greatly improving the reliability of study results (20).

Recent MR investigations have delved into the causal association between sarcopenia and a myriad of factors, such as lifestyle, coronary heart disease, osteoarthritis, major depressive disorder and autoimmune conditions (21–25). However, MR studies remains devoid of any pertinent investigations concerning the association between urate and sarcopenia. Consequently, we have undertaken the application of MR analysis to elucidate the causal link between genetically determined serum urate levels and sarcopenia.

## Methods

### Study design overview

**Figure 1:**
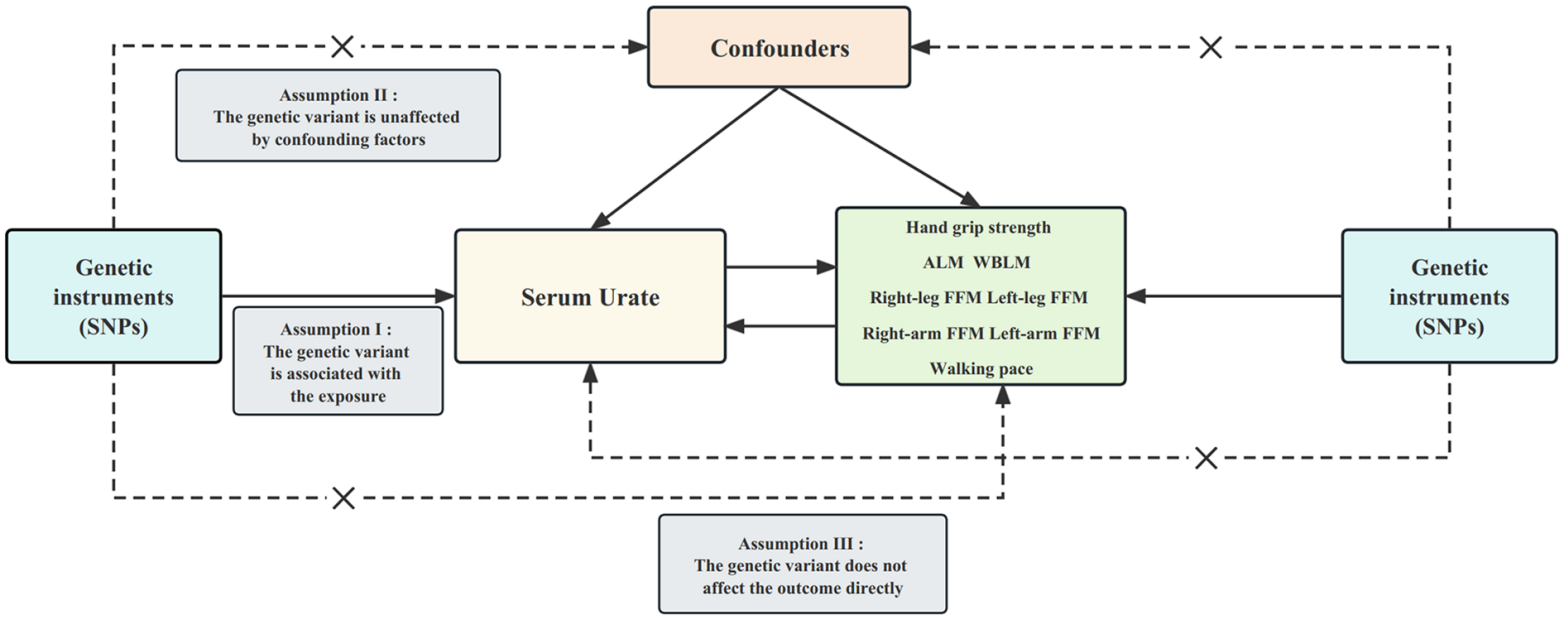
Design of the current two-sample bidirectional Mendelian randomization study. The three core assumptions are as follows: **(I)** relevance assumption; **(II)** independence assumption; and (III) exclusion restriction. Bold line represents direct effect and dotted line represents indirect effect. SNP, single nucleotide polymorphism; ALM, appendicular lean mass. WBLM, whole-body lean mass; FFM, fat-free mass.

**Figure 1** presents an overview of the design employed in our two-sample bidirectional MR study. Briefly, we initially explored the causal impact of serum urate levels on sarcopenia-related traits, followed by an assessment of the causal influence exerted by sarcopenia-related traits on serum urate levels. To establish genetic variants as instrumental variables (IVs), we followed three stringent assumptions. First, genetic variants needed to exhibit a high degree of correlation with the exposure. Second, these genetic variants had to remain unaffected by confounding factors like body mass index. Lastly, the influence of the genetic variants on the outcomes had to be solely mediated through the exposure pathway (26). Importantly, all MR analyses conducted in our study utilized publicly available summary statistics, obviating the need for additional ethical approval or informed consent.

### Data sources

The summary data for serum urate levels were extracted from the CKDGen consortium, which recently conducted an expansive genome-wide association study (GWAS) encompassing 288,649 participants of European descent (27). Furthermore, summary data concerning traits associated with sarcopenia were procured from the UK Biobank—an extensive biomedical database and research repository housing comprehensive genetic and health profiles of nearly half a million participants hailing from the UK, all within the age range of 40 to 69 years (28). The characteristics of the study population were delineated in **Supplementary Table 1**.

Hand grip strength, a frequently used indicator of muscular health, serves as a straightforward and non-intrusive gauge of overall musculoskeletal robustness (29). The summary statistics for grip strength have been culled from a recently disseminated GWAS conducted within the expansive UK Biobank cohort, encompassing a substantial sample size comprising both right-hand grip strength (N=461,089) and left-hand grip strength (N=461,026). The precision of these grip strength measurements was ensured through the employment of a meticulously calibrated Jamar J00105 hydraulic hand dynamometer, which facilitated individualized adjustments based on hand size (28). Moreover, each Single Nucleotide Polymorphism (SNP) underwent rigorous evaluation for its potential relationship with hand grip strength, with adjustments made for covariates including age and sex (28).

Lean mass, acknowledged as a robust metric for quantifying muscle mass, encompasses the entirety of fat-free soft tissue, encompassing not only muscle mass but also constituents such as body water, protein, glycerol, and soft tissue mineral mass (30, 31). In our investigation, we harnessed the GWAS summary statistics pertaining to various facets of lean mass: whole-body lean mass (WBLM; N=454,850), appendicular lean mass (ALM; N=450,243), and fat-free mass (FFM) in specific anatomical regions, including the right leg (N=454,835), left leg (N=454,805), right arm (N=454,753), and left arm (N=454,672). These measurements were meticulously acquired through the utilization of bioelectrical impedance analysis, with rigorous statistical adjustments accounting for covariates such as age and sex (28).

The evaluation of walking pace stands as a pivotal diagnostic criterion in the context of sarcopenia, given its close association with diminished physical performance—a hallmark trait of this condition (32). In our pursuit of genetic determinants underlying walking pace, we leveraged the summary statistics derived from the UK Biobank dataset, encompassing a cohort of 459,915 individuals of European descent.

### Selection of IVs

To ensure adherence to the assumptions of MR analysis, we extracted SNPs that exhibited a robust association with the exposure, meeting the genome-wide significance threshold of P<5×10^-8^. Furthermore, we performed linkage disequilibrium (LD) tests on these SNPs (r^2^<0.001 and clump distance >10,000kb) to ascertain their independence (33). In addition, F-statistics were computed to evaluate the strength of IVs, taking account of the sample size of the dataset, the number of IVs, and genetic variance. IVs exhibiting F-statistics below 10 were excluded from the analysis to mitigate the potential bias introduced by weak instrumental variables (34). Finally, we conducted a search in the Phenoscanner database (http://www.phenoscanner.medschl.cam.ac.uk/) to identify all SNPs associated with the exposure (P < 1 × 10^-5^). Our examination focused on determining whether any of these SNPs displayed discernible correlations with the confounding factors. Subsequently, SNPs demonstrating associations with relevant confounding factors were excluded from our analysis to minimize the potential influence of pleiotropic effects. Comprehensive information of SNPs for serum urate levels and sarcopenia-related traits can be found in **Supplementary Tables 2–11**.

### Mendelian randomization analyses

To evaluate the causal association of serum urate levels with sarcopenia-related traits, our study employed various MR analyses. The primary analysis utilized the inverse variance weighted (IVW) approach, which combines the Wald ratio estimates of causal effects derived from multiple SNPs. This approach ensures a consistent evaluation of the causal impact of exposure on the outcome under the assumption that each genetic variable satisfies the criteria of IVs (35). In addition, we employed the MR-Egger regression method, which is particularly effective in testing the null causal hypothesis and facilitating a consistent assessment of causality, even in cases where the IVs exhibit no valid genetic variation (36). Furthermore, we incorporated the WM methods, which provide reliable estimates of causal effects, even when up to 50% of analytical information is derived from invalid IVs (37).The estimates were reported as beta(β) values along with their corresponding 95% confidence intervals (CI) per one standard deviation increase in the exposures.

### Sensitivity analysis

To ensure the reliability and robustness of results, we undertook the sensitivity analysis including Cochran’s Q test, MR-Egger intercept test, funnel plot, and leave-one-out analysis. The Cochran’s Q test was utilized to evaluate potential heterogeneity, which ascertained whether variations in the IVs could potentially result in divergent outcomes (38). The MR-Egger intercept test was conducted with the aim of detecting the potential existence of directional pleiotropy, a phenomenon in which IVs exert effects on the outcomes through pathways beyond exposure (36). Funnel plots were used to visually examine the symmetry of the distribution of effect estimates. Any noticeable asymmetry in the funnel plot may indicate the presence of heterogeneity. And leave-one-out analysis entails the systematic exclusion of each SNP in succession, followed by a re-evaluation of the effect estimates, determining the reliability and robustness of the results by evaluating the influence of each SNP on the overall findings.

### Statistical analysis

Considering the multitude of tests conducted in this study, the criterion for establishing statistical significance regarding the primary outcome was set at a threshold of P value <0.006 (0.05/9) following the correction via the Bonferroni method. All statistical analyses were two-sided. All analyses were executed employing the TwoSampleMR package within R software (version 4.2.3).

## Results

### Causal effect of serum urate levels on sarcopenia-related traits

**Figure 2:**
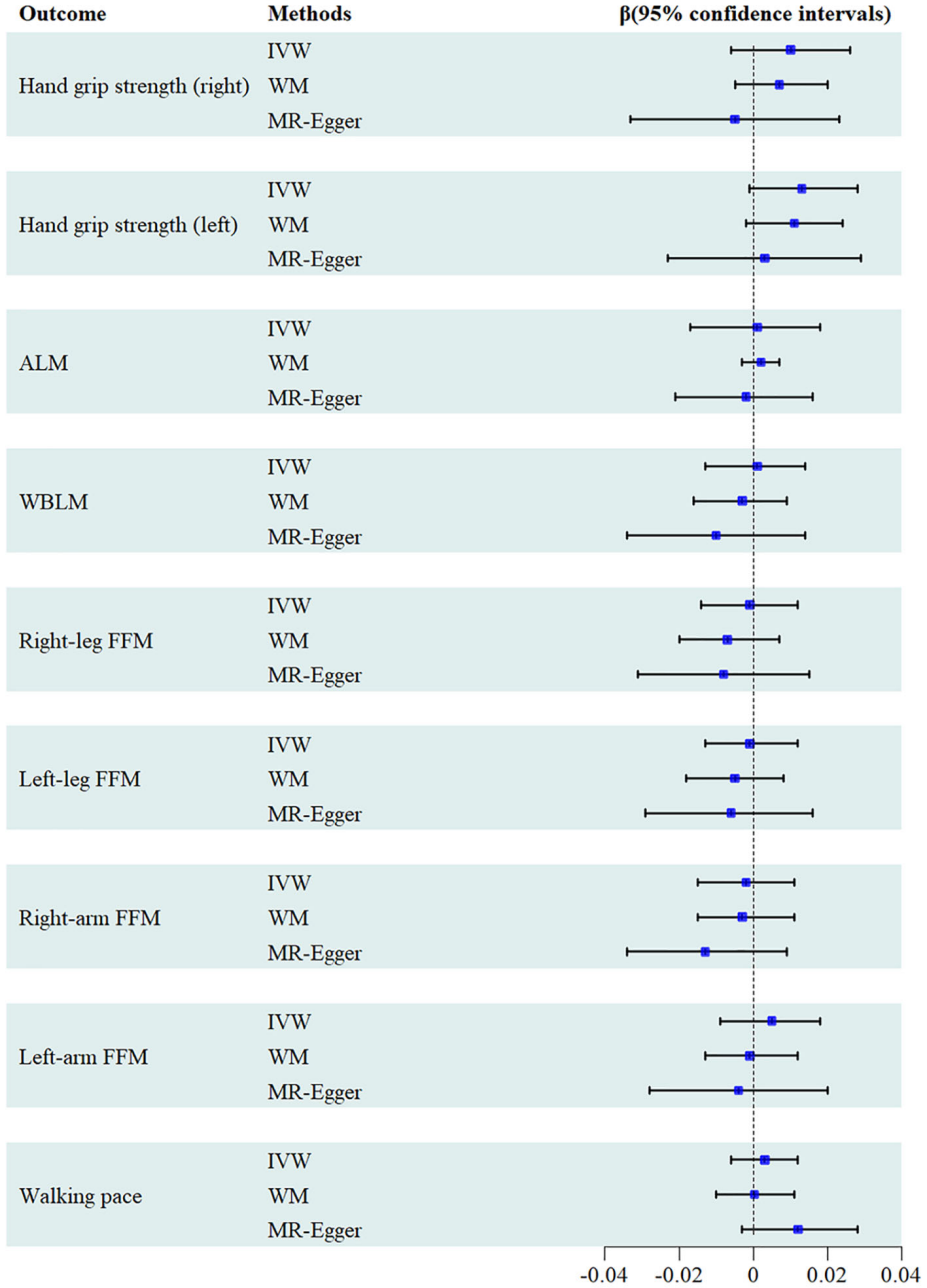
Forest plot of the causal effects of serum urate levels on sarcopenia.

A total of 86 SNPs were selected as IVs from the available GWAS data on serum urate levels. Employing the IVW analysis, we revealed no significant causal association of serum urate level with all sarcopenia-related traits. These findings, duly substantiated by both the MR-Egger and WM methods, aligned consistently with the results obtained from the IVW analysis (**Figure 2**). As shown in **Table 1**, the MR-Egger intercept tests indicated the absence of directional pleiotropic effects. However, the Cochran’s Q test uncovered significant heterogeneity among the relationships of urate levels with sarcopenia-related traits. Despite the presence of this observed heterogeneity, it should be underscored that it does not invalidate the applicability of the MR assessment using the random effects IVW approach in the current study. The utilization of random effects IVW analysis holds the potential to balance out the heterogeneity within the pooled set. Furthermore, the Egger intercept test did not identify any evidence of such bias, suggesting that the MR assessment remained robust and unaffected by pleiotropy bias, even within the context of heterogeneity. Leave-one-out analysis demonstrated the results are not driven by a single SNP, and funnel plots exhibited symmetrical distribution. Scatter plots, funnel plots and leave-one-out sensitivity analysis are represented in **Supplementary Figures 1–3**.

**Table 1:** MR results of the causal effect of urate on sarcopenia-related traits.

Exposures	Outcomes	No. of SNPs	Methods	β (95% CI)	P	Heterogeneity test			Pleiotropy test
						Cochran’s Q	P*		P Intercept
Urate	Hand grip strength (right)	81	IVW	0.010 (-0.006, 0.026)	0.199	374.23	<0.001		0.188
			WM	0.007 (-0.005, 0.020)	0.255				
			MR Egger	-0.005 (-0.033,0.023)	0.719				
Urate	Hand grip strength (left)	82	IVW	0.013 (-0.001, 0.028)	0.074	309.64	<0.001		0.349
			WM	0.011 (-0.002, 0.024)	0.086				
			MR Egger	0.003 (-0.023, 0.029)	0.813				
Urate	ALM	83	IVW	0.001 (-0.017, 0.018)	0.918	1570.12	<0.001		0.365
			WM	0.002 (-0.003, 0.007)	0.432				
			MR Egger	-0.002 (-0.021, 0.016)	0.805				
Urate	WBLM	63	IVW	0.001 (-0.013,0.014)	0.899	208.38	<0.001		0.279
			WM	-0.003 (-0.016,0.009)	0.614				
			MR Egger	-0.010 (-0.034,0.014)	0.409				
Urate	Right-leg FFM	65	IVW	-0.001 (-0.014,0.012)	0.0924	184.84	<0.001		0.442
			WM	-0.007 (-0.020,0.007)	0.333				
			MR Egger	-0.008 (-0.031,0.015)	0.490				
Urate	Left-leg FFM	65	IVW	-0.001 (-0.013,0.012)	0.937	172.80	<0.001		0.548
			WM	-0.005 (-0.018,0.008)	0.429				
			MR Egger	-0.006 (-0.029,0.016)	0.588				
Urate	Right-arm FFM	60	IVW	-0.002 (-0.015,0.011)	0.762	164.57	<0.001		0.247
			WM	-0.003 (-0.015,0.011)	0.667				
			MR Egger	-0.013 (-0.034,0.009)	0.263				
Urate	Left-arm FFM	66	IVW	0.005 (-0.009,0.018)	0.488	214.71	<0.001		0.374
			WM	-0.001 (-0.013,0.012)	0.933				
			MR Egger	-0.004 (-0.028,0.020)	0.721				
Urate	Walking pace	81	IVW	0.003 (-0.006,0.012)	0.562	168.07	<0.001		0.152
			WM	0.0004 (-0.010,0.011)	0.943				
			MR Egger	0.012 (-0.004,0.028)	0.132				

MR, Mendelian randomization; SNPs, single nucleotide polymorphisms; IVW, inverse variance weighted;

CI, confidence interval; WM, weighted median; ALM, appendicular lean mass; WBLM, whole-body lean mass;

FFM, fat-free mass.

p* represents heterogeneity.

### Causal effect of sarcopenia-related traits on serum urate levels

**Figure 3:**
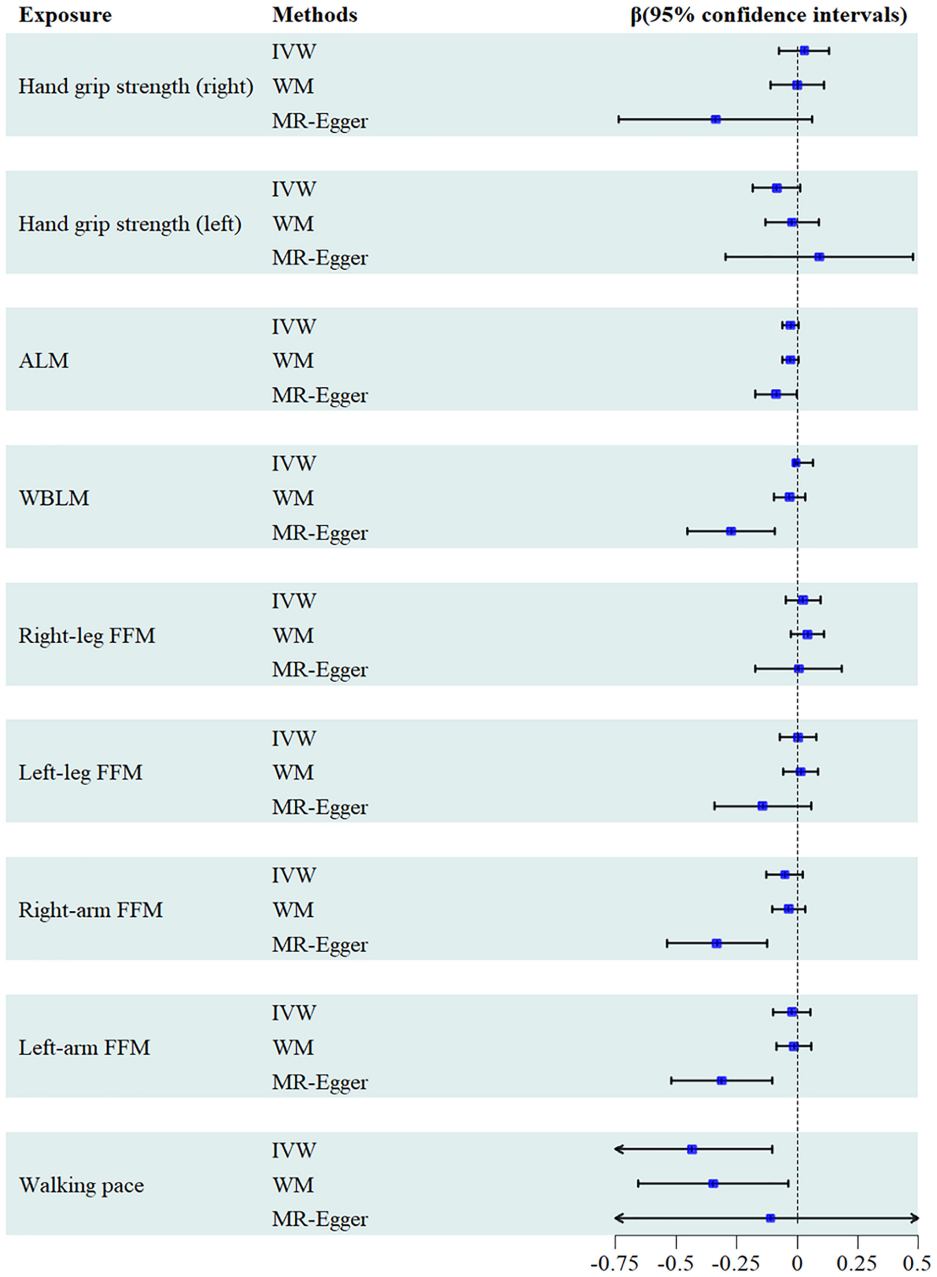
Forest plots of the causal effects of sarcopenia on serum urate levels.

A comprehensive selection encompassing 138 SNPs for right-hand grip strength, 127 SNPs for left-hand grip strength, 614 SNPs for ALM, 411SNPs for WBLM, 361 SNPs for right-leg fat-free mass(FFM), 339 SNPs for left-leg FFM, 365 SNPs for right-arm FFM, 356 SNPs for left-arm FFM, and 26 SNPs for walking pace were harnessed to explore their respective associations with urate levels. From the outcomes generated by the IVW method, it becomes evident that walking pace [β= -0.435, 95% confidence interval = -0.766, -0.103)] exhibited an inverse correlation with serum uric acid levels. However, no causal relationship could be established between other facets of sarcopenia-related traits and urate levels. These findings were consistently upheld by the WM or MR-Egger methodologies (**Figure 3**). The correlation between urate levels and sarcopenia-related traits displayed heterogeneity, as indicated by Cochran’s Q test (**Table 2**). Furthermore, the MR-Egger intercept test unveiled the presence of horizontal pleiotropy among WBLM, right-arm FFM and left-arm FFM with urate level. The results of the funnel plot demonstrated a symmetrical pattern. And the leave-one-out analysis indicated that the exclusion of any single SNP did not substantially alter or dominate the overall assessment conducted by the IVW method. Scatter plots, funnel plots and leave-one-out sensitivity analysis are represented in **Supplementary Figures 1–3**.

**Table 2:** MR results of the causal effect of hand grip strength and ALM on urate.

Exposures	Outcomes	No. of SNPs	Methods	β (95% CI)	P	Heterogeneity test		Pleiotropy test
						Cochran’s Q	P*	P Intercept
Hand grip strength (right)	Urate	130	IVW	0.029 (-0.074, 0.131)	0.583	285.42	<0.001	0.065
			WM	0.000 (-0.112, 0.111)	0.997			
			MR Egger	-0.337 (-0.736,0.061)	0.100			
Hand grip strength (left)	Urate	125	IVW	-0.085 (-0.183, 0.013)	0.088	241.77	<0.001	0.360
			WM	-0.022 (-0.133, 0.089)	0.704			
			MR Egger	0.091 (-0.297, 0.479)	0.647			
ALM	Urate	614	IVW	-0.028 (-0.061, 0.006)	0.110	1735.91	<0.001	0.132
			WM	0.029 (-0.063, 0.006)	0.103			
			MR Egger	-0.087 (-0.172,-0.003)	0.044			
WBLM	Urate	396	IVW	-0.006(-0.076,0.063)	0.855	1474.52	<0.001	0.002
			WM	-0.032(-0.097,0.033)	0.332			
			MR Egger	-0.273(-0.452,-0.094)	0.003			
Right-leg FFM	Urate	341	IVW	0.024(-0.047,0.096)	0.509	1252.44	<0.001	0.828
			WM	0.042(-0.027,0.110)	0.035			
			MR Egger	0.006(-0.172,0.184)	0.947			
Left-leg FFM	Urate	318	IVW	0.004(-0.072,0.080)	0.918	1155.99	<0.001	0.120
			WM	0.014(-0.058,0.086)	0.704			
			MR Egger	-0.143(-0.342,0.057)	0.162			
Right-arm FFM	Urate	352	IVW	-0.051(-0.127,0.024)	0.179	1223.75	<0.001	0.004
			WM	-0.036(-0.104,0.032)	0.301			
			MR Egger	-0.332(-0.539,-0.125)	0.002			
Left-arm FFM	Urate	346	IVW	-0.022(-0.1,0.055)	0.577	1318.97	<0.001	0.004
			WM	-0.014(-0.085,0.057)	0.7			
			MR Egger	-0.312(-0.52,-0.104)	0.004			
Walking pace	Urate	26	IVW	-0.435(-0.766, -0.103)	0.01	67.51	<0.001	0.644
			WM	-0.347(-0.656, -0.038)	0.028			
			MR Egger	-0.110(-1.510, 1.290)	0.879			

MR, Mendelian randomization; SNPs, single nucleotide polymorphisms; IVW, inverse variance weighted;

CI, confidence interval; WM, weighted median; ALM, appendicular lean mass. WBLM, whole-body lean mass;

FFM, fat-free mass.

p* represents heterogeneity.

## Discussion

To the best of our acknowledge, this investigation represents the inaugural genetic exploration with the purpose of unraveling the potential causal association linking serum urate levels and sarcopenia. By virtue of our MR analysis, we uncovered no substantiated indications endorsing the causal association between serum urate levels and sarcopenia. Similarly, our study did not reveal any discernible causal effect of sarcopenia on serum urate levels.

The majority of previous observational investigations has indicated a positive correlation between serum urate levels and sarcopenia. However, these findings remain uncertain, as divergent conclusions have been presented by several studies. Multiple cross-sectional studies conducted in China (39), Japan (40), Korea (41), the United States (42), and Italy (43) have intimated the potential protective effect of augmented serum urate levels on muscle strength. A longitudinally conducted follow-up study spanning three years demonstrated that escalated uric acid levels were prospectively and independently associated with improved muscle strength (44). Conversely, a decade-long longitudinal investigation, focusing on a Tasmanian cohort of elderly individuals, revealed the significant association between elevated urate concentrations and the rate of muscle strength degeneration (45). Additionally, a Japanese study encompassing individuals aged 30 and above identified a distinctive inverse-J relationship between serum urate quartiles and muscle strength (46). Similarly, the relationship between urate levels and muscle mass remains uncertain, with the establishment of a causal association yet to be ascertained. Two prospective studies from Israel (47) and China (48) have provided evidence of the positive association between serum urate levels and muscle mass. Nevertheless, a prospective study executed among Spanish community-dwelling elderly adults found an inverse association between urate levels and muscle mass (49). Additionally, a cross-sectional study conducted in the United States did not identify any substantial correlation between serum uric acid and muscle mass (50). It is important to acknowledge that these observational studies are susceptible to the influence of confounding factors and the potential concern of reverse causality, which poses challenges in establishing the causal link between serum uric acid levels and muscle strength and mass. Nevertheless, these limitations can be mitigated through the application of MR studies.

Oxidative stress denotes an intricate disturbance in the equilibrium between oxidative and antioxidant activity within the biological system, culminating in the excessive production and accumulation of oxidative radicals, which inflicts deleterious effects upon cells and tissues (51). A multitude of investigations has substantiated the pivotal engagement of oxidative stress in the pathogenesis of sarcopenia-induced muscle loss (52). Oxidative stress has the capacity to trigger the occurrence of oxidative protein damage in muscle cells, wherein the structural integrity of proteins is compromised by various oxidative modifications, including protein oxidation and hydroxylation. Consequently, the functionality and stability of muscular proteins are diminished, impeding their intrinsic physiological functions (53). Furthermore, mitochondria, acknowledged as the primary source of ROS production in skeletal muscle, emerge as susceptible targets of the oxidative onslaught. The sustained impact of oxidative stress impairs mitochondrial function, compromising the energy supply of muscle cells, and ultimately resulting in the waning of muscular performance (54–56). Simultaneously, oxidative stress exerts severe interference with the repair system of mitochondrial DNA, undermining the regenerative capacity of muscle tissue (57). Furthermore, it is noteworthy that oxidative stress has the ability to elicit an inflammatory response within muscle tissue. The escalated production of ROS activates signaling pathways associated with inflammation, resulting in the release of inflammatory cytokines and chemokines (58, 59). While the inflammatory response serves as a defensive mechanism against oxidative stress to a certain extent, an excessive or protracted inflammatory reaction can inflict detrimental consequences upon muscle tissue, culminating in damage and degeneration (60, 61). In this intricate milieu, the emergence of uric acid assumes a distinctive role. With its profound antioxidative properties, uric acid efficiently scavenges ROS, asserting dominance over approximately two-thirds of the overall plasma antioxidant capacity (8, 62). Elevated levels of uric acid have consistently demonstrated a positive correlation with heightened serum antioxidant capacity and enhanced resistance against oxidative stress (63). These findings postulate that uric acid may assume a protective function in mitigating muscle loss by fortifying the defense against oxidative stress.

The significance of inflammation in the pathogenesis and progression of sarcopenia cannot be disregarded, as an abundance of studies has revealed a negative correlation between elevated levels of inflammatory cytokines and both muscular strength and mass (64, 65). Inflammatory cytokines possess the capacity to activate diverse molecular pathways connected to skeletal muscle atrophy, precipitating an imbalance in protein synthesis and catabolism, consequently expediting the process of muscular wasting (66). Notably, uric acid has been shown to exhibit an intimate connection with the inflammatory response (67, 68). Uric acid is capable of directly stimulating mitogen-activated protein kinases, instigating inflammatory signaling pathways, and augmenting the production of pro-inflammatory cytokines including interleukin 1(IL-1), interleukin 6(IL-6), and tumor necrosis factor (TNF), which exert pro-inflammatory effects independent of ROS (69, 70). Consequently, the inflammatory response elicited by uric acid may exert potentially detrimental effects on muscle mass and function. This implies that while uric acid may harbor a protective effect on muscle, it also gives rise to unfavorable consequences. However, the ultimate ramifications of uric acid-induced inflammation and antioxidant stress on muscle remain uncertain at present. The analysis conducted in the present MR study unequivocally establishes no association between urate levels and both muscle strength and mass, which engenders the speculation that such an outcome may arise from a compensatory interplay between uric acid-induced inflammation and the counteractive influence of antioxidative stress.

The proposition that elderly individuals afflicted with asymptomatic hyperuricemia could refrain from pursuing aggressive uric acid-lowering therapy to impede the advancement of sarcopenia (13), is unequivocally disproved by our findings. It is of paramount importance to acknowledge that the presence of hyperuricemia, even in the absence of symptoms, manifests a profound correlation with the emergence of various comorbidities, type 2 diabetes mellitus, and cardiovascular disease (71). Consequently, it becomes imperative to approach the correlation between uric acid and sarcopenia with the utmost caution, lest capitulating to the administration of misguided interventions that have the potential to engender deleterious consequences. Furthermore, it is imperative to acknowledge the potential heterogeneity within the populace of distinct clinical subcategories of sarcopenia. Genetic variations present in individuals exhibiting various subtypes of sarcopenia may elicit divergent responses to serum uric acid concentrations. Consequently, it becomes evident that future investigations should be directed towards a comprehensive exploration of these nuanced distinctions.

Our investigation boasts several strengths. Primarily, the utilization of the MR analysis harnessed genetic variation, effectively attenuating the influence of potential confounding factors and reverse causality. Secondly, we employed a diverse array of methods within our MR analysis to ensure the accuracy and validity of the results. By incorporating multiple statistical models in our sensitivity analysis, we effectively assessed the consistency of the primary outcomes, furnishing compelling and reliable evidence. Finally, our study was underpinned by the selection of the most recent and dependable data from a substantial sample size. Nevertheless, Certain limitations should be acknowledged in our study. Firstly, the composition of our study population was exclusively European, impeding the direct generalization of our results to individuals from different ethnic backgrounds and distinct cultural milieus. Secondly, old age and an individual’s specific health condition are profoundly intertwined with serum urate levels and development of sarcopenia. Regrettably, we were precluded from investigating these variables in greater depth owing to the unavailability of individual-level data. Furthermore, the utilization of ALM as a gauge of muscle mass may introduce a measure of inaccuracy, owing to potential biases stemming from other non-fat soft tissue constituents. Lastly, although grip strength serves as an objective and common marker of muscle strength, it predominantly represents upper body potency, failing short of comprehensively capturing the strength across the entire body.

## Conclusion

In conclusion, our bi-directional MR analysis found no causal association between serum urate levels and sarcopenia. In light of this finding, it is incumbent upon us to exercise prudence when considering interventions targeting serum urate levels as a means to postpone the onset or progression of sarcopenia.

## Data availability statement

The original contributions presented in the study are included in the article/**Supplementary Material**. Further inquiries can be directed to the corresponding author.

## Author contributions

FF and YL took involved in the study design. YL and XW conducted clinical assessments. YS, WY and JZ were responsible for data collection. YL, and XW analyzed the data. The text was written by YL and was reviewed by additional writers. All the authors contributed to this article and approved the submitted version.
