# Supplementary material for: Causality between urate levels with sarcopenia-related traits: a bi-directional Mendelian randomization study
Source: Front Endocrinol (Lausanne). 2023 Oct 26;14:1252968. doi: 10.3389/fendo.2023.1252968 (PMC10637380; doi:10.3389/fendo.2023.1252968)
Supplement: Supplementary file 1 [file DataSheet_1.docx]

Supplementary Table S1 Baseline characteristics of the study population

| **Traits** | **Consortium** | **Sample Size** | **Total number of**  **SNPs tested** | **Ancestry** |
| --- | --- | --- | --- | --- |
| Urate | CKDGen | 288,649 | 8,217,338 | European |
| Right hand grip | MRC-IEU | 461,089 | 9,851,867 | European |
| Left hand grip | MRC-IEU | 461,026 | 9,851,867 | European |
| ALM | NA | 450,243 | 18,071,518 | European |
| WBLM | MRC-IEU | 454,850 | 9,851,867 | European |
| Right-leg FFM | MRC-IEU | 454,835 | 9,851,867 | European |
| Left-leg FFM | MRC-IEU | 454,805 | 9,851,867 | European |
| Right-arm FFM | MRC-IEU | 454,753 | 9,851,867 | European |
| Left-arm FFM | MRC-IEU | 454,672 | 9,851,867 | European |
| Walking pace | MRC-IEU | 459,915 | 9,851,867 | European |

SNPs, single nucleotide polymorphisms; ALM, appendicular lean mass;

FFM,fat-free mass;WBLM,whole-body lean mass.

Supplementary Table 2 Characteristics of SNPs used as genetic instruments for urate

| Exposure | SNP | Position | EA | NEA | EAF | SNP-Exposure association | | | | R^2 a^ | F-statistic ^b^ | Confounders ^c^ |
| --- | --- | --- | --- | --- | --- | --- | --- | --- | --- | --- | --- | --- |
|  |  |  |  |  |  | SE | Beta | | P value |  |  |  |
| Urate | rs2480712 | 13419 | C | G | 0.662 | 0.024 | 0.004 | 6.25E-09 | | 0.0001 | 34 |  |
| Urate | rs4646068 | 130919 | T | C | 0.692 | 0.024 | 0.004 | 7.71E-09 | | 0.0001 | 33 |  |
| Urate | rs79598313 | 227228 | T | C | 0.026 | 0.1 | 0.013 | 9.22E-15 | | 0.0002 | 60 |  |
| Urate | rs77923202 | 962607 | A | T | 0.02 | -0.18 | 0.031 | 1.50E-08 | | 0.0001 | 32 |  |
| Urate | rs10910845 | 968734 | A | C | 0.469 | 0.058 | 0.004 | 1.50E-51 | | 0.0008 | 228 |  |
| Urate | rs2070803 | 1020478 | A | G | 0.578 | 0.053 | 0.004 | 4.09E-41 | | 0.0006 | 180 |  |
| Urate | rs139429135 | 1092699 | A | G | 0.515 | -0.04 | 0.006 | 4.33E-11 | | 0.0002 | 44 |  |
| Urate | rs12037861 | 1565584 | A | T | 0.704 | 0.023 | 0.004 | 3.39E-08 | | 0.0001 | 31 |  |
| Urate | rs2867112 | 1821711 | T | G | 0.83 | 0.035 | 0.005 | 9.84E-12 | | 0.0002 | 46 | BMI |
| Urate | rs72782806 | 1962980 | A | G | 0.26 | 0.025 | 0.004 | 8.12E-09 | | 0.0001 | 33 |  |
| Urate | rs1260326 | 2060515 | T | C | 0.398 | 0.07 | 0.004 | 4.61E-69 | | 0.0011 | 309 |  |
| Urate | rs12472381 | 2367727 | A | G | 0.39 | 0.022 | 0.004 | 1.80E-08 | | 0.0001 | 32 | BMI |
| Urate | rs12987661 | 2454789 | T | C | 0.866 | 0.041 | 0.006 | 1.44E-12 | | 0.0002 | 50 |  |
| Urate | rs17050272 | 2819524 | A | G | 0.421 | 0.032 | 0.004 | 1.57E-15 | | 0.0002 | 64 |  |
| Urate | rs373773367 | 2921165 | T | C | 0.011 | 3.593 | 0.414 | 3.92E-18 | | 0.0003 | 75 |  |
| Urate | rs11683692 | 3022608 | T | C | 0.944 | -0.05 | 0.008 | 1.32E-08 | | 0.0001 | 32 |  |
| Urate | rs1234413 | 3045512 | T | C | 0.442 | -0.02 | 0.004 | 7.08E-09 | | 0.0001 | 34 |  |
| Urate | rs9287911 | 3212114 | A | T | 0.25 | 0.038 | 0.004 | 1.13E-17 | | 0.0003 | 73 |  |
| Urate | rs187355703 | 3270384 | C | G | 0.975 | -0.09 | 0.013 | 2.70E-11 | | 0.0002 | 44 |  |
| Urate | rs1047891 | 3542703 | A | C | 0.311 | -0.02 | 0.004 | 2.09E-08 | | 0.0001 | 31 | BMI |
| Urate | rs9288447 | 3557763 | T | C | 0.546 | -0.02 | 0.004 | 3.27E-09 | | 0.0001 | 35 |  |
| Urate | rs2581817 | 4303866 | C | G | 0.42 | 0.048 | 0.004 | 4.87E-35 | | 0.0005 | 153 |  |
| Urate | rs11128111 | 4451327 | T | C | 0.48 | -0.02 | 0.004 | 4.64E-08 | | 0.0001 | 30 |  |
| Urate | rs7640441 | 4907252 | A | C | 0.246 | -0.03 | 0.005 | 1.26E-09 | | 0.0001 | 37 |  |
| Urate | rs76651220 | 4970189 | A | C | 0.051 | 0.062 | 0.011 | 1.78E-08 | | 0.0001 | 32 |  |
| Urate | rs62294340 | 5273822 | A | G | 0.364 | -0.02 | 0.004 | 5.00E-08 | | 0.0001 | 30 |  |
| Urate | rs6283 | 5627174 | T | C | 0.661 | 0.044 | 0.005 | 3.09E-19 | | 0.0003 | 80 |  |
| Urate | rs16889260 | 5627591 | T | C | 0.186 | -0.15 | 0.005 | 1.00E-200 | | 0.0034 | 984 |  |
| Urate | rs16892069 | 5629576 | T | C | 0.946 | 0.189 | 0.009 | 3.65E-97 | | 0.0015 | 438 |  |
| Urate | rs114750851 | 5635228 | A | G | 0.969 | 0.27 | 0.012 | 2.19E-107 | | 0.0017 | 485 |  |
| Urate | rs2108878 | 5636682 | T | C | 0.703 | 0.107 | 0.004 | 7.54E-143 | | 0.0022 | 648 |  |
| Urate | rs98270 | 5975006 | A | G | 0.362 | 0.022 | 0.004 | 4.21E-08 | | 0.0001 | 30 |  |
| Urate | rs1383585 | 6305906 | A | G | 0.902 | -0.2 | 0.006 | 1.00E-200 | | 0.0035 | 1004 |  |
| Urate | rs62310614 | 6306492 | A | G | 0.11 | -0.06 | 0.006 | 2.80E-19 | | 0.0003 | 81 |  |
| Urate | rs111625726 | 6306530 | T | C | 0.702 | 0.076 | 0.004 | 9.33E-71 | | 0.0011 | 316 |  |
| Urate | rs1440411 | 6777332 | T | C | 0.571 | -0.03 | 0.004 | 1.08E-12 | | 0.0002 | 51 |  |
| Urate | rs455213 | 7513542 | T | C | 0.543 | -0.03 | 0.004 | 6.05E-12 | | 0.0002 | 47 |  |
| Urate | rs10942549 | 7788574 | C | G | 0.312 | -0.04 | 0.004 | 1.64E-22 | | 0.0003 | 95 |  |
| Urate | rs76004499 | 8676188 | C | G | 0.972 | -0.07 | 0.013 | 3.27E-08 | | 0.0001 | 31 |  |
| Urate | rs12530084 | 8782354 | T | C | 0.22 | 0.066 | 0.005 | 9.55E-48 | | 0.0007 | 211 |  |
| Urate | rs1359232 | 8953858 | A | C | 0.466 | -0.09 | 0.004 | 4.12E-126 | | 0.002 | 571 |  |
| Urate | rs2396025 | 9168247 | A | T | 0.22 | 0.05 | 0.009 | 1.65E-08 | | 0.0001 | 32 |  |
| Urate | rs10223666 | 9171534 | C | G | 0.704 | 0.046 | 0.004 | 6.62E-28 | | 0.0004 | 120 |  |
| Urate | rs4897160 | 9860463 | A | G | 0.483 | 0.03 | 0.004 | 1.96E-14 | | 0.0002 | 59 |  |
| Urate | rs62435145 | 10267522 | T | G | 0.689 | 0.042 | 0.005 | 2.36E-16 | | 0.0002 | 67 |  |
| Urate | rs13226650 | 10907092 | A | G | 0.809 | 0.049 | 0.005 | 1.35E-23 | | 0.0003 | 100 |  |
| Urate | rs11551890 | 11099302 | A | G | 0.509 | 0.023 | 0.004 | 2.40E-08 | | 0.0001 | 31 |  |
| Urate | rs10480300 | 11533388 | T | C | 0.276 | 0.03 | 0.004 | 4.26E-12 | | 0.0002 | 48 |  |
| Urate | rs34861762 | 11921338 | T | C | 0.419 | 0.034 | 0.004 | 3.50E-19 | | 0.0003 | 80 |  |
| Urate | rs2943539 | 12348843 | T | C | 0.475 | 0.041 | 0.004 | 6.42E-28 | | 0.0004 | 120 |  |
| Urate | rs10956924 | 12506721 | T | C | 0.279 | -0.02 | 0.004 | 1.79E-08 | | 0.0001 | 32 |  |
| Urate | rs10971420 | 13288009 | T | C | 0.688 | 0.031 | 0.004 | 4.14E-14 | | 0.0002 | 57 |  |
| Urate | rs56106601 | 13845245 | A | C | 0.946 | 0.061 | 0.009 | 2.68E-11 | | 0.0002 | 44 |  |
| Urate | rs74440730 | 14110427 | A | C | 0.892 | -0.04 | 0.006 | 2.22E-09 | | 0.0001 | 36 |  |
| Urate | rs10994731 | 14382328 | A | G | 0.882 | -0.06 | 0.006 | 4.01E-23 | | 0.0003 | 98 |  |
| Urate | rs1171617 | 14463997 | T | G | 0.768 | 0.079 | 0.005 | 1.81E-66 | | 0.001 | 297 |  |
| Urate | rs9420446 | 14701189 | T | C | 0.137 | -0.04 | 0.006 | 1.13E-11 | | 0.0002 | 46 |  |
| Urate | rs35198068 | 14915719 | T | C | 0.706 | 0.025 | 0.004 | 5.85E-09 | | 0.0001 | 34 | BMI |
| Urate | rs35506085 | 15128390 | A | G | 0.189 | -0.03 | 0.005 | 1.50E-08 | | 0.0001 | 32 |  |
| Urate | rs3925584 | 15391475 | T | C | 0.552 | 0.03 | 0.004 | 1.66E-15 | | 0.0002 | 63 |  |
| Urate | rs71456318 | 15656231 | A | C | 0.484 | 0.079 | 0.004 | 4.41E-92 | | 0.0014 | 414 |  |
| Urate | rs7126110 | 15657417 | C | G | 0.855 | -0.07 | 0.006 | 1.40E-32 | | 0.0005 | 141 |  |
| Urate | rs12576996 | 15665877 | T | G | 0.737 | -0.05 | 0.004 | 3.75E-26 | | 0.0004 | 112 |  |
| Urate | rs10892354 | 16130416 | T | C | 0.38 | 0.03 | 0.004 | 2.60E-13 | | 0.0002 | 54 |  |
| Urate | rs7303595 | 16407269 | A | T | 0.336 | 0.025 | 0.004 | 7.05E-10 | | 0.0001 | 38 |  |
| Urate | rs11614136 | 16704177 | A | T | 0.644 | -0.03 | 0.004 | 9.90E-13 | | 0.0002 | 51 |  |
| Urate | rs12313306 | 16748855 | T | C | 0.246 | -0.08 | 0.004 | 6.74E-65 | | 0.001 | 289 |  |
| Urate | rs10774625 | 17181004 | A | G | 0.483 | 0.032 | 0.004 | 5.54E-17 | | 0.0002 | 70 | BMI |
| Urate | rs28530689 | 17268767 | A | C | 0.512 | 0.032 | 0.004 | 1.27E-16 | | 0.0002 | 69 | BMI |
| Urate | rs12423664 | 17368650 | A | G | 0.152 | 0.042 | 0.006 | 1.75E-13 | | 0.0002 | 54 |  |
| Urate | rs7986094 | 17488422 | A | C | 0.302 | -0.02 | 0.004 | 1.74E-08 | | 0.0001 | 32 | BMI |
| Urate | rs626277 | 17833627 | A | C | 0.594 | 0.026 | 0.004 | 2.69E-11 | | 0.0002 | 44 |  |
| Urate | rs861536 | 18931156 | A | G | 0.621 | 0.024 | 0.004 | 2.16E-09 | | 0.0001 | 36 | BMI |
| Urate | rs9708153 | 18961105 | T | C | 0.015 | 2.986 | 0.289 | 4.58E-25 | | 0.0004 | 107 |  |
| Urate | rs1478604 | 19098578 | T | C | 0.706 | -0.03 | 0.004 | 4.49E-10 | | 0.0001 | 39 |  |
| Urate | rs6495044 | 19364788 | A | G | 0.709 | 0.024 | 0.004 | 1.20E-08 | | 0.0001 | 33 | Body fat percentage |
| Urate | rs10851885 | 19386509 | A | G | 0.756 | -0.05 | 0.005 | 4.16E-32 | | 0.0005 | 139 |  |
| Urate | rs12908437 | 19586442 | T | C | 0.376 | 0.046 | 0.004 | 1.56E-30 | | 0.0005 | 132 | Body fat percentage |
| Urate | rs4997081 | 19831982 | C | G | 0.196 | -0.03 | 0.005 | 4.18E-10 | | 0.0001 | 39 |  |
| Urate | rs8050136 | 19981022 | A | C | 0.403 | 0.025 | 0.004 | 2.34E-10 | | 0.0001 | 40 | BMI |
| Urate | rs62052820 | 20112742 | A | G | 0.212 | 0.041 | 0.005 | 2.81E-18 | | 0.0003 | 76 | BMI |
| Urate | rs12920245 | 20132147 | A | C | 0.105 | 0.038 | 0.006 | 4.13E-09 | | 0.0001 | 35 |  |
| Urate | rs57652769 | 20220225 | T | C | 0.309 | -0.04 | 0.004 | 8.56E-18 | | 0.0003 | 74 |  |
| Urate | rs9925837 | 20222718 | A | G | 0.845 | -0.04 | 0.005 | 5.85E-15 | | 0.0002 | 61 |  |
| Urate | rs2453580 | 20528145 | T | C | 0.598 | 0.025 | 0.004 | 7.01E-10 | | 0.0001 | 38 |  |
| Urate | rs4617927 | 20755876 | T | G | 0.446 | 0.035 | 0.004 | 4.55E-19 | | 0.0003 | 80 |  |
| Urate | rs9895661 | 20798446 | T | C | 0.817 | 0.05 | 0.005 | 7.23E-23 | | 0.0003 | 97 |  |
| Urate | rs11663816 | 21443775 | T | C | 0.73 | -0.03 | 0.004 | 1.40E-12 | | 0.0002 | 50 | BMI |
| Urate | rs117864137 | 21678875 | A | G | 0.731 | 0.028 | 0.004 | 4.87E-11 | | 0.0001 | 43 |  |
| Urate | rs10405423 | 21700229 | A | C | 0.663 | 0.039 | 0.004 | 1.07E-20 | | 0.0003 | 87 |  |
| Urate | rs4808762 | 21797199 | T | C | 0.72 | -0.02 | 0.004 | 1.36E-08 | | 0.0001 | 32 | BMI |
| Urate | rs2868194 | 21908207 | T | C | 0.408 | -0.03 | 0.004 | 8.90E-12 | | 0.0002 | 47 |  |
| Urate | rs148062412 | 22048373 | A | T | 0.037 | 0.164 | 0.028 | 5.71E-09 | | 0.0001 | 34 |  |
| Urate | rs62128132 | 22055499 | T | C | 0.966 | -0.12 | 0.015 | 1.32E-15 | | 0.0002 | 64 |  |
| Urate | rs7267595 | 22237465 | A | C | 0.51 | 0.023 | 0.004 | 3.15E-09 | | 0.0001 | 35 |  |
| Urate | rs6119510 | 22399390 | T | G | 0.596 | -0.02 | 0.004 | 3.20E-09 | | 0.0001 | 35 |  |
| Urate | rs1800961 | 22474922 | T | C | 0.034 | -0.08 | 0.012 | 1.63E-10 | | 0.0001 | 41 |  |
| Urate | rs219781 | 22860591 | T | G | 0.246 | -0.03 | 0.004 | 1.56E-08 | | 0.0001 | 32 |  |
| Urate | rs549120832 | 22960610 | T | C | 0.012 | 3.675 | 0.378 | 2.27E-22 | | 0.0003 | 95 |  |
| Urate | rs12485100 | 23192443 | T | G | 0.173 | -0.03 | 0.005 | 2.44E-10 | | 0.0001 | 40 |  |

Abbreviation: SNP, single nucleotide polymorphism; EA, Effect allele; NEA, Non-effect allele; EAF, effect allele frequency; SE, standard error; BMI, body mass index.

*^a^ R^2^* was calculated using the following formula: (2×EAF×(1-EAF)×beta^2^)/[(2×EAF×(1-EAF)×beta^2^)+(2×EAF×(1-EAF)×N×SE^2^)], where EAF is the effect allele

frequency, beta is the estimated effect on urate. Ν is the sample size of the GWAS for the SNP-urate association and SE is the standard error of the estimated effect.

*^b^ F* statistic was calculated using the following formula: *R^2^*(N-2)/(1-*R^2^*), where *R^2^* is the proportion of variance in urate explained by each instrument and N is the sample size of the GWAS for the SNP-urate association.

^c^ SNPs associated with confounding factors were removed after searching Phenoscanner database.

Supplementary Table 3 Characteristics of SNPs used as genetic instruments for hand grip strength(right)

| Exposure | SNP | Position | EA | NEA | EAF | SNP-Exposure association | | | R^2 a^ | F-statistic ^b^ | Confounders ^c^ |
| --- | --- | --- | --- | --- | --- | --- | --- | --- | --- | --- | --- |
|  |  |  |  |  |  | SE Beta P value | | |  |  |  |
| Hand grip strength (right) | rs58670122 | 22492613 | G | A | 0.143 | 0.002 | -0.013 | 7.40E-10 | 0.0001 | 38 |  |
| Hand grip strength (right) | rs10798876 | 32074514 | G | C | 0.552 | 0.001 | 0.009 | 4.30E-09 | 0.0001 | 35 |  |
| Hand grip strength (right) | rs10798483 | 176799143 | A | G | 0.547 | 0.001 | 0.015 | 2.80E-22 | 0.0002 | 94 |  |
| Hand grip strength (right) | rs6693965 | 10378416 | T | G | 0.128 | 0.002 | -0.016 | 3.20E-13 | 0.0001 | 53 |  |
| Hand grip strength (right) | rs4927015 | 54072471 | A | G | 0.583 | 0.002 | 0.013 | 5.00E-18 | 0.0002 | 75 |  |
| Hand grip strength (right) | rs1952256 | 184035116 | G | A | 0.345 | 0.002 | 0.010 | 4.10E-10 | 0.0001 | 39 |  |
| Hand grip strength (right) | rs35304341 | 200971049 | A | G | 0.089 | 0.003 | -0.014 | 4.00E-08 | 0.0001 | 30 |  |
| Hand grip strength (right) | rs823130 | 205714372 | T | C | 0.433 | 0.002 | -0.012 | 1.70E-16 | 0.0001 | 68 |  |
| Hand grip strength (right) | rs56144131 | 208977656 | C | T | 0.149 | 0.002 | -0.013 | 3.80E-10 | 0.0001 | 39 |  |
| Hand grip strength (right) | rs7549184 | 215412906 | A | G | 0.787 | 0.002 | 0.011 | 5.80E-09 | 0.0001 | 34 |  |
| Hand grip strength (right) | rs10799428 | 227798565 | T | C | 0.187 | 0.002 | -0.014 | 4.60E-14 | 0.0001 | 57 |  |
| Hand grip strength (right) | rs12562146 | 86247267 | A | T | 0.145 | 0.002 | 0.012 | 2.00E-08 | 0.0001 | 32 |  |
| Hand grip strength (right) | rs2147461 | 191051627 | C | T | 0.117 | 0.002 | 0.013 | 6.10E-09 | 0.0001 | 34 |  |
| Hand grip strength (right) | rs4121165 | 78276977 | A | G | 0.211 | 0.002 | -0.012 | 4.80E-11 | 0.0001 | 43 | BMI |
| Hand grip strength (right) | rs6693567 | 150510660 | T | C | 0.733 | 0.002 | -0.010 | 6.30E-09 | 0.0001 | 34 |  |
| Hand grip strength (right) | rs1892425 | 41744821 | A | G | 0.239 | 0.002 | 0.011 | 1.10E-10 | 0.0001 | 42 |  |
| Hand grip strength (right) | rs150330307 | 160160801 | C | T | 0.032 | 0.004 | -0.033 | 1.20E-14 | 0.0001 | 60 |  |
| Hand grip strength (right) | rs1550115 | 25041620 | T | C | 0.749 | 0.002 | 0.015 | 4.50E-19 | 0.0002 | 80 | BMI |
| Hand grip strength (right) | rs1442883 | 59970660 | A | C | 0.253 | 0.002 | -0.011 | 5.80E-10 | 0.0001 | 39 |  |
| Hand grip strength (right) | rs6711390 | 135629439 | T | C | 0.371 | 0.002 | 0.013 | 4.00E-18 | 0.0002 | 75 | body fat percentage |
| Hand grip strength (right) | rs35833641 | 179462494 | G | A | 0.312 | 0.002 | 0.009 | 8.50E-09 | 0.0001 | 33 |  |
| Hand grip strength (right) | rs1840753 | 199060177 | T | C | 0.063 | 0.003 | 0.018 | 1.40E-08 | 0.0001 | 32 |  |
| Hand grip strength (right) | rs1047891 | 211540507 | A | C | 0.316 | 0.002 | 0.010 | 1.30E-09 | 0.0001 | 37 | BMI |
| Hand grip strength (right) | rs2894602 | 227249802 | G | A | 0.766 | 0.002 | 0.010 | 9.90E-09 | 0.0001 | 33 |  |
| Hand grip strength (right) | rs7565148 | 44188396 | G | T | 0.501 | 0.001 | -0.010 | 3.60E-12 | 0.0001 | 48 |  |
| Hand grip strength (right) | rs7576964 | 48601448 | T | G | 0.342 | 0.002 | 0.010 | 5.30E-10 | 0.0001 | 39 |  |
| Hand grip strength (right) | rs34030812 | 144248905 | C | T | 0.367 | 0.002 | -0.009 | 3.30E-09 | 0.0001 | 35 |  |
| Hand grip strength (right) | rs7575451 | 152352843 | G | C | 0.650 | 0.002 | -0.011 | 9.30E-12 | 0.0001 | 47 |  |
| Hand grip strength (right) | rs12616285 | 169156907 | G | T | 0.149 | 0.002 | 0.012 | 5.40E-09 | 0.0001 | 34 | BMI |
| Hand grip strength (right) | rs6715064 | 220041928 | T | C | 0.310 | 0.002 | -0.009 | 9.60E-09 | 0.0001 | 33 |  |
| Hand grip strength (right) | rs1641457 | 40421990 | G | T | 0.223 | 0.002 | 0.013 | 2.40E-13 | 0.0001 | 54 |  |
| Hand grip strength (right) | rs3771498 | 70720070 | T | C | 0.514 | 0.001 | 0.014 | 1.90E-21 | 0.0002 | 91 |  |
| Hand grip strength (right) | rs12052508 | 72757843 | T | C | 0.879 | 0.002 | -0.013 | 4.70E-09 | 0.0001 | 34 |  |
| Hand grip strength (right) | rs10193039 | 99692373 | T | A | 0.281 | 0.002 | -0.010 | 4.10E-10 | 0.0001 | 39 |  |
| Hand grip strength (right) | rs2194747 | 218126181 | G | A | 0.707 | 0.002 | 0.010 | 1.90E-09 | 0.0001 | 36 |  |
| Hand grip strength (right) | rs6792762 | 38574491 | A | G | 0.420 | 0.002 | -0.009 | 1.80E-09 | 0.0001 | 36 |  |
| Hand grip strength (right) | rs7652177 | 171969077 | G | C | 0.505 | 0.001 | 0.009 | 7.90E-09 | 0.0001 | 33 |  |
| Hand grip strength (right) | rs2194411 | 185548663 | A | G | 0.128 | 0.002 | 0.014 | 2.10E-10 | 0.0001 | 40 | BMI |
| Hand grip strength (right) | rs1440152 | 98489915 | G | C | 0.445 | 0.001 | 0.008 | 3.50E-08 | 0.0001 | 30 |  |
| Hand grip strength (right) | rs2362972 | 158163272 | A | C | 0.578 | 0.002 | -0.009 | 1.30E-08 | 0.0001 | 32 | BMI |
| Hand grip strength (right) | rs62234790 | 13750642 | A | C | 0.246 | 0.002 | 0.011 | 8.80E-10 | 0.0001 | 38 |  |
| Hand grip strength (right) | rs35701422 | 85575775 | C | T | 0.626 | 0.002 | -0.009 | 1.50E-08 | 0.0001 | 32 | BMI |
| Hand grip strength (right) | rs2341184 | 196930781 | C | T | 0.272 | 0.002 | 0.010 | 1.70E-09 | 0.0001 | 36 | body fat percentage |
| Hand grip strength (right) | rs35457492 | 70194564 | C | A | 0.495 | 0.001 | 0.008 | 2.20E-08 | 0.0001 | 31 |  |
| Hand grip strength (right) | rs9757079 | 53155158 | T | C | 0.318 | 0.002 | 0.010 | 8.40E-10 | 0.0001 | 38 | BMI |
| Hand grip strength (right) | rs71298370 | 71164965 | A | G | 0.086 | 0.003 | 0.017 | 8.90E-10 | 0.0001 | 38 |  |
| Hand grip strength (right) | rs9853018 | 141101961 | T | C | 0.443 | 0.001 | 0.010 | 8.80E-12 | 0.0001 | 47 | body fat percentage |
| Hand grip strength (right) | rs34587452 | 1009900 | C | G | 0.215 | 0.002 | -0.011 | 9.10E-10 | 0.0001 | 38 |  |
| Hand grip strength (right) | rs7657558 | 30648636 | G | T | 0.720 | 0.002 | 0.011 | 1.10E-10 | 0.0001 | 42 |  |
| Hand grip strength (right) | rs114924396 | 119755621 | G | A | 0.054 | 0.003 | -0.019 | 8.20E-09 | 0.0001 | 33 |  |
| Hand grip strength (right) | rs13146142 | 17931318 | C | T | 0.159 | 0.002 | -0.021 | 1.40E-24 | 0.0002 | 105 | body fat percentage |
| Hand grip strength (right) | rs13106087 | 145566864 | C | T | 0.830 | 0.002 | 0.013 | 6.20E-11 | 0.0001 | 43 |  |
| Hand grip strength (right) | rs997850 | 154838434 | C | G | 0.605 | 0.002 | -0.009 | 3.70E-09 | 0.0001 | 35 |  |
| Hand grip strength (right) | rs13107325 | 103188709 | T | C | 0.075 | 0.003 | -0.028 | 2.10E-22 | 0.0002 | 95 | BMI |
| Hand grip strength (right) | rs13169333 | 153064994 | C | T | 0.260 | 0.002 | 0.009 | 4.30E-08 | 0.0001 | 30 |  |
| Hand grip strength (right) | rs75457267 | 102658770 | T | C | 0.051 | 0.003 | -0.019 | 3.40E-08 | 0.0001 | 30 |  |
| Hand grip strength (right) | rs12522139 | 122699812 | G | T | 0.171 | 0.002 | -0.011 | 6.80E-09 | 0.0001 | 34 | BMI |
| Hand grip strength (right) | rs6882168 | 39402647 | T | C | 0.337 | 0.002 | -0.009 | 7.90E-09 | 0.0001 | 33 |  |
| Hand grip strength (right) | rs13356200 | 67820946 | G | T | 0.394 | 0.002 | -0.009 | 1.80E-09 | 0.0001 | 36 |  |
| Hand grip strength (right) | rs6870324 | 141787317 | G | C | 0.270 | 0.002 | -0.010 | 3.00E-09 | 0.0001 | 35 |  |
| Hand grip strength (right) | rs13355365 | 154942606 | T | C | 0.380 | 0.002 | -0.008 | 3.40E-08 | 0.0001 | 31 |  |
| Hand grip strength (right) | rs4868110 | 171164168 | T | A | 0.323 | 0.002 | -0.010 | 1.00E-09 | 0.0001 | 37 |  |
| Hand grip strength (right) | rs2431112 | 103931707 | A | G | 0.441 | 0.001 | -0.011 | 8.60E-14 | 0.0001 | 56 | BMI |
| Hand grip strength (right) | rs2631360 | 131707429 | A | G | 0.519 | 0.001 | -0.011 | 5.40E-14 | 0.0001 | 57 |  |
| Hand grip strength (right) | rs2322754 | 81046299 | A | G | 0.832 | 0.002 | -0.012 | 3.80E-09 | 0.0001 | 35 |  |
| Hand grip strength (right) | rs9322822 | 105369598 | T | C | 0.320 | 0.002 | 0.011 | 3.50E-12 | 0.0001 | 48 |  |
| Hand grip strength (right) | rs9388051 | 122577108 | A | G | 0.186 | 0.002 | 0.011 | 2.70E-08 | 0.0001 | 31 |  |
| Hand grip strength (right) | rs7451021 | 130381246 | C | T | 0.689 | 0.002 | -0.016 | 7.70E-23 | 0.0002 | 97 |  |
| Hand grip strength (right) | rs113835839 | 13784625 | T | C | 0.248 | 0.002 | -0.010 | 1.10E-08 | 0.0001 | 33 |  |
| Hand grip strength (right) | rs11243202 | 7719065 | C | T | 0.486 | 0.001 | 0.012 | 6.50E-15 | 0.0001 | 61 |  |
| Hand grip strength (right) | rs645144 | 141180780 | C | T | 0.330 | 0.002 | -0.009 | 4.30E-08 | 0.0001 | 30 |  |
| Hand grip strength (right) | rs721101 | 155632844 | C | T | 0.271 | 0.002 | 0.009 | 1.60E-08 | 0.0001 | 32 |  |
| Hand grip strength (right) | rs77485342 | 30842866 | T | C | 0.018 | 0.006 | 0.035 | 2.60E-10 | 0.0001 | 40 |  |
| Hand grip strength (right) | rs9267806 | 32110886 | A | G | 0.256 | 0.002 | -0.017 | 7.70E-23 | 0.0002 | 97 |  |
| Hand grip strength (right) | rs185320691 | 32490292 | C | G | 0.104 | 0.003 | -0.020 | 5.60E-14 | 0.0001 | 57 |  |
| Hand grip strength (right) | rs113315602 | 32574575 | C | A | 0.096 | 0.003 | -0.021 | 1.40E-15 | 0.0001 | 64 | body fat percentage |
| Hand grip strength (right) | rs1125 | 149979416 | A | G | 0.337 | 0.002 | -0.010 | 1.90E-10 | 0.0001 | 41 |  |
| Hand grip strength (right) | rs9396861 | 18404133 | A | C | 0.599 | 0.002 | -0.010 | 5.70E-10 | 0.0001 | 39 |  |
| Hand grip strength (right) | rs35175534 | 32530029 | C | A | 0.140 | 0.002 | -0.019 | 4.20E-16 | 0.0001 | 66 |  |
| Hand grip strength (right) | rs1885690 | 109672998 | A | C | 0.410 | 0.002 | -0.008 | 2.80E-08 | 0.0001 | 31 |  |
| Hand grip strength (right) | rs852520 | 5695267 | A | C | 0.662 | 0.002 | -0.009 | 1.50E-08 | 0.0001 | 32 |  |
| Hand grip strength (right) | rs4549685 | 39326478 | T | C | 0.330 | 0.002 | 0.010 | 8.10E-10 | 0.0001 | 38 | BMI |
| Hand grip strength (right) | rs112330055 | 23109316 | A | G | 0.063 | 0.003 | 0.018 | 1.70E-08 | 0.0001 | 32 |  |
| Hand grip strength (right) | rs7790322 | 2830498 | T | C | 0.416 | 0.002 | -0.009 | 1.20E-08 | 0.0001 | 33 |  |
| Hand grip strength (right) | rs6962338 | 69160985 | G | A | 0.044 | 0.004 | -0.020 | 2.00E-08 | 0.0001 | 32 |  |
| Hand grip strength (right) | rs6977081 | 150542515 | T | G | 0.334 | 0.002 | 0.013 | 5.70E-16 | 0.0001 | 66 | body fat percentage |
| Hand grip strength (right) | rs2389763 | 17307847 | C | T | 0.592 | 0.002 | -0.008 | 3.80E-08 | 0.0001 | 30 |  |
| Hand grip strength (right) | rs2717351 | 19019880 | G | A | 0.212 | 0.002 | 0.013 | 3.40E-12 | 0.0001 | 48 | body fat percentage |
| Hand grip strength (right) | rs9639938 | 46262729 | G | C | 0.541 | 0.001 | 0.009 | 5.20E-09 | 0.0001 | 34 |  |
| Hand grip strength (right) | rs10278546 | 100516003 | C | A | 0.195 | 0.002 | 0.011 | 1.10E-08 | 0.0001 | 33 |  |
| Hand grip strength (right) | rs4730984 | 120655676 | T | G | 0.240 | 0.002 | 0.010 | 1.80E-09 | 0.0001 | 36 |  |
| Hand grip strength (right) | rs4737446 | 57665019 | T | G | 0.695 | 0.002 | 0.010 | 2.40E-10 | 0.0001 | 40 |  |
| Hand grip strength (right) | rs62509875 | 110360944 | G | A | 0.171 | 0.002 | -0.013 | 2.40E-11 | 0.0001 | 45 |  |
| Hand grip strength (right) | rs1486925 | 78827617 | C | T | 0.315 | 0.002 | -0.010 | 2.90E-09 | 0.0001 | 35 |  |
| Hand grip strength (right) | rs6473015 | 78178485 | C | A | 0.286 | 0.002 | 0.010 | 5.50E-09 | 0.0001 | 34 |  |
| Hand grip strength (right) | rs7871404 | 99262296 | G | A | 0.189 | 0.002 | 0.012 | 3.40E-10 | 0.0001 | 39 | body fat percentage |
| Hand grip strength (right) | rs116922558 | 118802375 | G | A | 0.040 | 0.004 | -0.025 | 1.80E-10 | 0.0001 | 41 |  |
| Hand grip strength (right) | rs2208562 | 119344528 | T | C | 0.610 | 0.002 | -0.012 | 3.10E-14 | 0.0001 | 58 |  |
| Hand grip strength (right) | rs7034200 | 4289050 | A | C | 0.480 | 0.001 | 0.009 | 5.30E-09 | 0.0001 | 34 |  |
| Hand grip strength (right) | rs113851275 | 98297220 | A | G | 0.108 | 0.002 | 0.013 | 4.30E-08 | 0.0001 | 30 |  |
| Hand grip strength (right) | rs11998884 | 33684436 | T | C | 0.062 | 0.003 | 0.017 | 2.80E-08 | 0.0001 | 31 |  |
| Hand grip strength (right) | rs600038 | 136151806 | C | T | 0.207 | 0.002 | -0.010 | 1.50E-08 | 0.0001 | 32 |  |
| Hand grip strength (right) | rs10761411 | 136973826 | T | C | 0.812 | 0.002 | -0.011 | 1.30E-08 | 0.0001 | 32 | body fat percentage |
| Hand grip strength (right) | rs72820369 | 81251539 | T | A | 0.118 | 0.002 | 0.016 | 4.40E-12 | 0.0001 | 48 |  |
| Hand grip strength (right) | rs4751671 | 116138744 | A | G | 0.531 | 0.002 | 0.008 | 4.10E-08 | 0.0001 | 30 |  |
| Hand grip strength (right) | rs4752689 | 124131176 | A | G | 0.584 | 0.002 | 0.009 | 6.40E-09 | 0.0001 | 34 |  |
| Hand grip strength (right) | rs12412806 | 24860913 | A | G | 0.294 | 0.002 | -0.009 | 3.00E-08 | 0.0001 | 31 |  |
| Hand grip strength (right) | rs2273555 | 104127171 | A | G | 0.606 | 0.002 | 0.011 | 4.20E-13 | 0.0001 | 53 |  |
| Hand grip strength (right) | rs4962700 | 126479989 | G | C | 0.302 | 0.002 | 0.009 | 1.20E-08 | 0.0001 | 33 |  |
| Hand grip strength (right) | rs1556659 | 130834698 | T | C | 0.382 | 0.002 | 0.018 | 3.80E-30 | 0.0003 | 130 |  |
| Hand grip strength (right) | rs12763284 | 104508202 | G | A | 0.466 | 0.001 | 0.010 | 1.20E-10 | 0.0001 | 42 |  |
| Hand grip strength (right) | rs11022513 | 12840986 | T | C | 0.569 | 0.002 | -0.009 | 1.10E-09 | 0.0001 | 37 | body fat percentage |
| Hand grip strength (right) | rs11039348 | 47728617 | A | G | 0.348 | 0.002 | -0.010 | 3.90E-10 | 0.0001 | 39 | BMI |
| Hand grip strength (right) | rs2244621 | 64026219 | T | C | 0.144 | 0.002 | 0.012 | 4.50E-08 | 0.0001 | 30 |  |
| Hand grip strength (right) | rs61389091 | 74427921 | T | C | 0.042 | 0.004 | 0.022 | 3.60E-09 | 0.0001 | 35 |  |
| Hand grip strength (right) | rs34845616 | 133792644 | A | G | 0.246 | 0.002 | 0.010 | 1.70E-08 | 0.0001 | 32 |  |
| Hand grip strength (right) | rs12790261 | 66988048 | A | C | 0.082 | 0.003 | -0.026 | 2.00E-22 | 0.0002 | 95 |  |
| Hand grip strength (right) | rs72977282 | 74300441 | A | T | 0.414 | 0.002 | -0.017 | 1.20E-28 | 0.0003 | 123 |  |
| Hand grip strength (right) | rs6592737 | 77322619 | T | A | 0.372 | 0.002 | -0.009 | 2.00E-09 | 0.0001 | 36 |  |
| Hand grip strength (right) | rs10770125 | 2169014 | G | A | 0.477 | 0.001 | 0.008 | 1.40E-08 | 0.0001 | 32 |  |
| Hand grip strength (right) | rs1635527 | 48396364 | C | G | 0.547 | 0.001 | 0.010 | 6.80E-11 | 0.0001 | 43 |  |
| Hand grip strength (right) | rs76895963 | 4384844 | G | T | 0.021 | 0.006 | 0.036 | 4.10E-10 | 0.0001 | 39 | BMI |
| Hand grip strength (right) | rs7301953 | 124405871 | A | G | 0.312 | 0.002 | -0.012 | 6.40E-13 | 0.0001 | 52 | body fat percentage |
| Hand grip strength (right) | rs10846071 | 15016236 | T | C | 0.394 | 0.002 | -0.016 | 6.50E-25 | 0.0002 | 106 |  |
| Hand grip strength (right) | rs12823922 | 24186697 | G | A | 0.222 | 0.002 | -0.011 | 1.60E-10 | 0.0001 | 41 |  |
| Hand grip strength (right) | rs10784502 | 66343810 | T | C | 0.512 | 0.001 | -0.011 | 7.10E-14 | 0.0001 | 56 | BMI |
| Hand grip strength (right) | rs7963801 | 79685226 | C | T | 0.572 | 0.002 | -0.011 | 8.40E-14 | 0.0001 | 56 |  |
| Hand grip strength (right) | rs12316046 | 15054415 | G | A | 0.378 | 0.002 | -0.016 | 3.80E-26 | 0.0002 | 112 |  |
| Hand grip strength (right) | rs4768725 | 46848478 | C | T | 0.700 | 0.002 | 0.009 | 2.30E-08 | 0.0001 | 32 |  |
| Hand grip strength (right) | rs7953280 | 94136009 | C | G | 0.507 | 0.001 | -0.009 | 1.80E-09 | 0.0001 | 36 |  |
| Hand grip strength (right) | rs3118914 | 51116901 | T | G | 0.215 | 0.002 | -0.019 | 5.90E-27 | 0.0003 | 116 | waist hip ratio |
| Hand grip strength (right) | rs2296316 | 65520246 | C | T | 0.464 | 0.002 | -0.008 | 4.60E-08 | 0.0001 | 30 |  |
| Hand grip strength (right) | rs12889267 | 21542766 | G | A | 0.167 | 0.002 | -0.012 | 6.70E-10 | 0.0001 | 38 |  |
| Hand grip strength (right) | rs935728 | 80957923 | T | C | 0.328 | 0.002 | 0.010 | 1.80E-09 | 0.0001 | 36 |  |
| Hand grip strength (right) | rs7148603 | 36683779 | A | G | 0.359 | 0.002 | 0.009 | 4.70E-09 | 0.0001 | 34 |  |
| Hand grip strength (right) | rs10483727 | 61072875 | C | T | 0.610 | 0.002 | -0.009 | 3.30E-09 | 0.0001 | 35 |  |
| Hand grip strength (right) | rs9652468 | 56823913 | A | G | 0.249 | 0.002 | -0.013 | 3.30E-13 | 0.0001 | 53 |  |
| Hand grip strength (right) | rs2871865 | 99194896 | G | C | 0.116 | 0.002 | -0.024 | 1.80E-24 | 0.0002 | 104 |  |
| Hand grip strength (right) | rs2165241 | 74222202 | C | T | 0.509 | 0.001 | 0.012 | 1.90E-16 | 0.0001 | 68 |  |
| Hand grip strength (right) | rs4553566 | 58336319 | C | T | 0.453 | 0.001 | -0.009 | 4.60E-10 | 0.0001 | 39 |  |
| Hand grip strength (right) | rs12914702 | 96887277 | A | G | 0.300 | 0.002 | 0.011 | 1.90E-10 | 0.0001 | 41 |  |
| Hand grip strength (right) | rs12101479 | 74248548 | C | G | 0.237 | 0.002 | -0.011 | 1.50E-09 | 0.0001 | 37 |  |
| Hand grip strength (right) | rs12899474 | 77391603 | T | C | 0.108 | 0.002 | -0.015 | 5.40E-10 | 0.0001 | 39 |  |
| Hand grip strength (right) | rs246181 | 14392641 | T | C | 0.373 | 0.002 | 0.010 | 3.70E-10 | 0.0001 | 39 |  |
| Hand grip strength (right) | rs11642954 | 24824248 | A | G | 0.195 | 0.002 | -0.013 | 2.20E-12 | 0.0001 | 49 | BMI |
| Hand grip strength (right) | rs7196917 | 69896527 | G | A | 0.430 | 0.002 | -0.011 | 1.90E-12 | 0.0001 | 50 | BMI |
| Hand grip strength (right) | rs8055199 | 84867404 | A | G | 0.660 | 0.002 | -0.009 | 1.20E-08 | 0.0001 | 33 |  |
| Hand grip strength (right) | rs7206195 | 2145280 | T | C | 0.180 | 0.002 | -0.015 | 2.80E-15 | 0.0001 | 62 | body fat percentage |
| Hand grip strength (right) | rs248831 | 11281218 | A | G | 0.265 | 0.002 | 0.010 | 8.40E-09 | 0.0001 | 33 |  |
| Hand grip strength (right) | rs62037412 | 28917746 | A | G | 0.357 | 0.002 | 0.009 | 1.90E-09 | 0.0001 | 36 | BMI |
| Hand grip strength (right) | rs4785574 | 89568875 | G | A | 0.555 | 0.001 | -0.010 | 4.50E-12 | 0.0001 | 48 |  |
| Hand grip strength (right) | rs3848369 | 415078 | T | C | 0.387 | 0.002 | -0.010 | 4.70E-10 | 0.0001 | 39 |  |
| Hand grip strength (right) | rs76749769 | 3291408 | T | C | 0.091 | 0.003 | 0.014 | 2.20E-08 | 0.0001 | 31 |  |
| Hand grip strength (right) | rs4784329 | 53910261 | C | A | 0.426 | 0.002 | -0.013 | 9.00E-19 | 0.0002 | 78 | BMI |
| Hand grip strength (right) | rs7214252 | 27486673 | A | G | 0.211 | 0.002 | -0.010 | 2.00E-08 | 0.0001 | 32 |  |
| Hand grip strength (right) | rs2854152 | 61986027 | G | A | 0.678 | 0.002 | 0.011 | 6.00E-12 | 0.0001 | 47 | body fat percentage |
| Hand grip strength (right) | rs1043515 | 36922196 | G | A | 0.566 | 0.001 | 0.014 | 2.80E-20 | 0.0002 | 85 |  |
| Hand grip strength (right) | rs12452505 | 63556402 | G | C | 0.142 | 0.002 | -0.014 | 1.40E-11 | 0.0001 | 46 |  |
| Hand grip strength (right) | rs2587505 | 77784268 | C | T | 0.420 | 0.002 | -0.009 | 8.10E-10 | 0.0001 | 38 | BMI |
| Hand grip strength (right) | rs4793658 | 45878733 | C | A | 0.110 | 0.002 | -0.014 | 3.80E-08 | 0.0001 | 30 |  |
| Hand grip strength (right) | rs56074046 | 7358930 | A | G | 0.372 | 0.002 | -0.009 | 4.40E-09 | 0.0001 | 34 | body fat percentage |
| Hand grip strength (right) | rs56365901 | 43960323 | G | A | 0.223 | 0.002 | -0.014 | 1.80E-15 | 0.0001 | 63 |  |
| Hand grip strength (right) | rs10520770 | 46602964 | C | T | 0.449 | 0.001 | 0.012 | 4.50E-15 | 0.0001 | 62 |  |
| Hand grip strength (right) | rs635538 | 53273614 | A | G | 0.914 | 0.003 | -0.022 | 1.50E-16 | 0.0001 | 68 |  |
| Hand grip strength (right) | rs4369779 | 20735408 | C | T | 0.789 | 0.002 | 0.017 | 3.40E-21 | 0.0002 | 89 | waist hip ratio |
| Hand grip strength (right) | rs34217742 | 37376830 | A | T | 0.124 | 0.002 | 0.015 | 1.40E-10 | 0.0001 | 41 |  |
| Hand grip strength (right) | rs7249 | 18391171 | T | C | 0.366 | 0.002 | 0.008 | 4.00E-08 | 0.0001 | 30 | BMI |
| Hand grip strength (right) | rs4802848 | 52218342 | C | G | 0.730 | 0.002 | 0.011 | 3.80E-11 | 0.0001 | 44 |  |
| Hand grip strength (right) | rs36065733 | 2163771 | G | T | 0.473 | 0.001 | 0.010 | 8.20E-11 | 0.0001 | 42 |  |
| Hand grip strength (right) | rs79723785 | 55818225 | C | T | 0.016 | 0.006 | -0.034 | 1.60E-08 | 0.0001 | 32 |  |
| Hand grip strength (right) | rs7266065 | 47531817 | A | G | 0.323 | 0.002 | 0.010 | 4.60E-10 | 0.0001 | 39 |  |
| Hand grip strength (right) | rs911642 | 13260252 | T | C | 0.376 | 0.002 | 0.009 | 2.00E-08 | 0.0001 | 32 |  |
| Hand grip strength (right) | rs6063504 | 48981014 | G | C | 0.493 | 0.001 | 0.009 | 7.80E-09 | 0.0001 | 33 |  |
| Hand grip strength (right) | rs143384 | 34025756 | G | A | 0.404 | 0.002 | 0.023 | 2.50E-52 | 0.0005 | 232 | waist hip ratio |
| Hand grip strength (right) | rs2226685 | 40069825 | C | T | 0.759 | 0.002 | 0.010 | 3.10E-09 | 0.0001 | 35 |  |
| Hand grip strength (right) | rs6006984 | 45714937 | C | T | 0.278 | 0.002 | 0.010 | 5.10E-10 | 0.0001 | 39 |  |
| Hand grip strength (right) | rs58670122 | 22492613 | G | A | 0.143 | 0.002 | -0.013 | 7.40E-10 | 0.0001 | 38 |  |
| Hand grip strength (right) | rs10798876 | 32074514 | G | C | 0.552 | 0.001 | 0.009 | 4.30E-09 | 0.0001 | 35 |  |

Abbreviation: SNP, single nucleotide polymorphism; EA, Effect allele; NEA, Non-effect allele; EAF, effect allele frequency; SE, standard error; BMI, body mass index.

*^a^ R^2^* was calculated using the following formula: (2×EAF×(1-EAF)×beta^2^)/[(2×EAF×(1-EAF)×beta^2^)+(2×EAF×(1-EAF)×N×SE^2^)], where EAF is the effect allele

frequency, beta is the estimated effect on urate. Ν is the sample size of the GWAS for the SNP-urate association and SE is the standard error of the estimated effect.

*^b^ F* statistic was calculated using the following formula: *R^2^*(N-2)/(1-*R^2^*), where *R^2^* is the proportion of variance in urate explained by each instrument and N is the sample size of the GWAS for the SNP-urate association.

^c^ SNPs associated with confounding factors were removed after searching Phenoscanner database.

Supplementary Table 4 Characteristics of SNPs used as genetic instruments for hand grip strength(left)

| Exposure | SNP | Position | EA | NEA | EAF | SNP-Exposure association | | | R^2 a^ | F-statistic ^b^ | Confounders ^c^ |
| --- | --- | --- | --- | --- | --- | --- | --- | --- | --- | --- | --- |
|  |  |  |  |  |  | Beta | SE | P value |  |  |  |
| Hand grip strength (left) | rs6680160 | 32072737 | G | A | 0.628 | 0.010 | 0.0015 | 6.00E-11 | 0.00009 | 43 |  |
| Hand grip strength (left) | rs7516571 | 40733658 | G | A | 0.259 | 0.009 | 0.0017 | 3.10E-08 | 0.00007 | 31 |  |
| Hand grip strength (left) | rs150330307 | 160160801 | C | T | 0.032 | -0.031 | 0.0042 | 2.90E-13 | 0.00012 | 53 |  |
| Hand grip strength (left) | rs2800789 | 164578242 | C | A | 0.480 | 0.008 | 0.0015 | 3.10E-08 | 0.00007 | 31 |  |
| Hand grip strength (left) | rs1044299 | 176811873 | T | C | 0.546 | 0.014 | 0.0015 | 6.60E-21 | 0.00019 | 88 |  |
| Hand grip strength (left) | rs11121542 | 10393920 | A | G | 0.123 | -0.016 | 0.0023 | 3.00E-12 | 0.00011 | 49 |  |
| Hand grip strength (left) | rs4121165 | 78276977 | A | G | 0.211 | -0.011 | 0.0018 | 3.40E-10 | 0.00009 | 39 | BMI |
| Hand grip strength (left) | rs58670122 | 22492613 | G | A | 0.143 | -0.012 | 0.0021 | 3.60E-08 | 0.00007 | 30 |  |
| Hand grip strength (left) | rs10788958 | 54040670 | G | C | 0.645 | 0.014 | 0.0016 | 1.00E-19 | 0.00018 | 83 |  |
| Hand grip strength (left) | rs4335354 | 88899964 | A | C | 0.316 | -0.009 | 0.0016 | 4.60E-09 | 0.00007 | 34 |  |
| Hand grip strength (left) | rs1884447 | 185021410 | A | G | 0.401 | 0.008 | 0.0015 | 2.30E-08 | 0.00007 | 31 |  |
| Hand grip strength (left) | rs61818100 | 190962663 | C | T | 0.117 | 0.013 | 0.0023 | 6.20E-09 | 0.00007 | 34 |  |
| Hand grip strength (left) | rs823130 | 205714372 | T | C | 0.433 | -0.011 | 0.0015 | 4.40E-14 | 0.00012 | 57 |  |
| Hand grip strength (left) | rs11204664 | 150531380 | C | T | 0.579 | -0.009 | 0.0015 | 8.10E-09 | 0.00007 | 33 | body fat percentage |
| Hand grip strength (left) | rs6689375 | 227721627 | T | A | 0.185 | -0.016 | 0.0019 | 5.30E-17 | 0.00015 | 70 |  |
| Hand grip strength (left) | rs6433478 | 175241482 | C | T | 0.544 | 0.009 | 0.0015 | 1.50E-09 | 0.00008 | 36 |  |
| Hand grip strength (left) | rs12473732 | 44118428 | T | C | 0.487 | 0.011 | 0.0015 | 1.40E-13 | 0.00012 | 55 |  |
| Hand grip strength (left) | rs7571789 | 70714793 | C | T | 0.523 | 0.013 | 0.0015 | 3.00E-18 | 0.00016 | 76 |  |
| Hand grip strength (left) | rs7575451 | 152352843 | G | C | 0.650 | -0.010 | 0.0016 | 3.80E-10 | 0.00009 | 39 |  |
| Hand grip strength (left) | rs1434095 | 179254330 | C | T | 0.875 | 0.014 | 0.0023 | 5.30E-10 | 0.00008 | 39 |  |
| Hand grip strength (left) | rs17630248 | 201137782 | C | T | 0.348 | -0.009 | 0.0016 | 4.10E-09 | 0.00008 | 35 |  |
| Hand grip strength (left) | rs1981612 | 199235664 | A | C | 0.456 | 0.009 | 0.0015 | 1.00E-09 | 0.00008 | 37 |  |
| Hand grip strength (left) | rs11125803 | 25052177 | T | C | 0.741 | 0.014 | 0.0017 | 3.80E-17 | 0.00015 | 71 | BMI |
| Hand grip strength (left) | rs1641457 | 40421990 | G | T | 0.223 | 0.012 | 0.0018 | 1.40E-11 | 0.00010 | 46 |  |
| Hand grip strength (left) | rs3819121 | 135622860 | C | T | 0.369 | 0.014 | 0.0015 | 2.90E-20 | 0.00018 | 85 | body fat percentage |
| Hand grip strength (left) | rs10176878 | 59952274 | C | T | 0.191 | -0.013 | 0.0019 | 8.60E-12 | 0.00010 | 47 |  |
| Hand grip strength (left) | rs61286123 | 60205600 | C | T | 0.228 | -0.010 | 0.0018 | 1.20E-08 | 0.00007 | 33 |  |
| Hand grip strength (left) | rs34030812 | 144248905 | C | T | 0.367 | -0.010 | 0.0015 | 4.10E-11 | 0.00009 | 44 |  |
| Hand grip strength (left) | rs10205394 | 218150948 | C | G | 0.201 | -0.011 | 0.0019 | 1.10E-09 | 0.00008 | 37 |  |
| Hand grip strength (left) | rs1551042 | 85630551 | C | A | 0.647 | -0.011 | 0.0016 | 7.40E-13 | 0.00011 | 51 | BMI |
| Hand grip strength (left) | rs9866627 | 135522715 | A | C | 0.084 | -0.016 | 0.0027 | 5.70E-09 | 0.00007 | 34 |  |
| Hand grip strength (left) | rs112485536 | 195971019 | T | C | 0.076 | 0.016 | 0.0028 | 9.40E-09 | 0.00007 | 33 |  |
| Hand grip strength (left) | rs62253653 | 53013267 | G | A | 0.295 | 0.011 | 0.0016 | 5.50E-11 | 0.00009 | 43 | BMI |
| Hand grip strength (left) | rs6802071 | 38574237 | T | C | 0.435 | -0.009 | 0.0015 | 3.80E-10 | 0.00009 | 39 |  |
| Hand grip strength (left) | rs71298370 | 71164965 | A | G | 0.086 | 0.015 | 0.0027 | 3.80E-08 | 0.00007 | 30 |  |
| Hand grip strength (left) | rs13091492 | 81891476 | G | A | 0.373 | -0.008 | 0.0015 | 3.30E-08 | 0.00007 | 31 | body fat percentage |
| Hand grip strength (left) | rs10934857 | 128199662 | A | G | 0.259 | 0.009 | 0.0017 | 4.80E-08 | 0.00006 | 30 |  |
| Hand grip strength (left) | rs4498020 | 13810820 | A | C | 0.724 | -0.010 | 0.0017 | 4.30E-10 | 0.00008 | 39 |  |
| Hand grip strength (left) | rs4677601 | 71368790 | G | A | 0.510 | 0.009 | 0.0015 | 1.10E-09 | 0.00008 | 37 |  |
| Hand grip strength (left) | rs2871960 | 141121814 | C | A | 0.445 | 0.012 | 0.0015 | 5.50E-16 | 0.00014 | 66 |  |
| Hand grip strength (left) | rs35609019 | 7847892 | C | G | 0.398 | 0.009 | 0.0015 | 7.70E-10 | 0.00008 | 38 |  |
| Hand grip strength (left) | rs13107325 | 103188709 | T | C | 0.075 | -0.026 | 0.0028 | 1.80E-20 | 0.00019 | 86 | BMI |
| Hand grip strength (left) | rs56338231 | 30867393 | G | A | 0.258 | -0.011 | 0.0017 | 1.70E-10 | 0.00009 | 41 |  |
| Hand grip strength (left) | rs13146142 | 17931318 | C | T | 0.159 | -0.020 | 0.0020 | 2.30E-23 | 0.00022 | 99 | body fat percentage |
| Hand grip strength (left) | rs34587452 | 1009900 | C | G | 0.215 | -0.011 | 0.0018 | 3.20E-10 | 0.00009 | 40 |  |
| Hand grip strength (left) | rs13106087 | 145566864 | C | T | 0.830 | 0.012 | 0.0020 | 3.90E-09 | 0.00008 | 35 |  |
| Hand grip strength (left) | rs997850 | 154838434 | C | G | 0.605 | -0.009 | 0.0015 | 6.30E-09 | 0.00007 | 34 |  |
| Hand grip strength (left) | rs34722008 | 38659594 | A | G | 0.353 | 0.009 | 0.0016 | 3.30E-08 | 0.00007 | 31 | BMI |
| Hand grip strength (left) | rs2850379 | 102917419 | A | C | 0.432 | -0.008 | 0.0015 | 3.30E-08 | 0.00007 | 31 |  |
| Hand grip strength (left) | rs75497896 | 119636703 | C | T | 0.051 | -0.021 | 0.0034 | 9.10E-10 | 0.00008 | 38 |  |
| Hand grip strength (left) | rs116409670 | 37327472 | T | C | 0.080 | -0.015 | 0.0027 | 2.60E-08 | 0.00007 | 31 |  |
| Hand grip strength (left) | rs55681913 | 42687629 | C | T | 0.106 | 0.014 | 0.0024 | 1.60E-08 | 0.00007 | 32 |  |
| Hand grip strength (left) | rs13356200 | 67820946 | G | T | 0.394 | -0.009 | 0.0015 | 9.70E-09 | 0.00007 | 33 |  |
| Hand grip strength (left) | rs2431112 | 103931707 | A | G | 0.441 | -0.010 | 0.0015 | 1.40E-10 | 0.00009 | 41 | BMI |
| Hand grip strength (left) | rs2631360 | 131707429 | A | G | 0.519 | -0.011 | 0.0015 | 1.90E-13 | 0.00012 | 54 |  |
| Hand grip strength (left) | rs6882168 | 39402647 | T | C | 0.337 | -0.009 | 0.0016 | 2.80E-09 | 0.00008 | 35 |  |
| Hand grip strength (left) | rs113918482 | 161289270 | G | A | 0.223 | -0.010 | 0.0018 | 1.50E-08 | 0.00007 | 32 |  |
| Hand grip strength (left) | rs2974438 | 168250903 | A | G | 0.211 | -0.010 | 0.0018 | 3.30E-08 | 0.00007 | 31 |  |
| Hand grip strength (left) | rs185320691 | 32490292 | C | G | 0.104 | -0.017 | 0.0027 | 5.50E-10 | 0.00008 | 39 |  |
| Hand grip strength (left) | rs12528131 | 105389104 | G | A | 0.488 | -0.009 | 0.0015 | 2.80E-09 | 0.00008 | 35 |  |
| Hand grip strength (left) | rs9371201 | 150145001 | T | C | 0.335 | -0.009 | 0.0016 | 3.00E-09 | 0.00008 | 35 |  |
| Hand grip strength (left) | rs77485342 | 30842866 | T | C | 0.018 | 0.033 | 0.0056 | 3.50E-09 | 0.00008 | 35 |  |
| Hand grip strength (left) | rs9388769 | 130379954 | A | G | 0.673 | -0.014 | 0.0016 | 5.10E-19 | 0.00017 | 79 |  |
| Hand grip strength (left) | rs35175534 | 32530029 | C | A | 0.140 | -0.016 | 0.0024 | 3.60E-12 | 0.00010 | 48 |  |
| Hand grip strength (left) | rs113315602 | 32574575 | C | A | 0.096 | -0.018 | 0.0027 | 8.60E-12 | 0.00010 | 47 | body fat percentage |
| Hand grip strength (left) | rs723588 | 81056634 | C | T | 0.143 | 0.013 | 0.0021 | 1.60E-09 | 0.00008 | 36 |  |
| Hand grip strength (left) | rs11243202 | 7719065 | C | T | 0.486 | 0.010 | 0.0015 | 7.00E-11 | 0.00009 | 43 |  |
| Hand grip strength (left) | rs4713506 | 32113980 | A | G | 0.256 | -0.016 | 0.0017 | 2.00E-20 | 0.00019 | 86 | BMI |
| Hand grip strength (left) | rs2038760 | 2680732 | T | C | 0.171 | -0.012 | 0.0020 | 6.60E-09 | 0.00007 | 34 |  |
| Hand grip strength (left) | rs41271299 | 19839415 | T | C | 0.051 | 0.021 | 0.0034 | 3.00E-10 | 0.00009 | 40 |  |
| Hand grip strength (left) | rs9371881 | 155638213 | A | G | 0.359 | 0.009 | 0.0015 | 9.60E-10 | 0.00008 | 37 |  |
| Hand grip strength (left) | rs4621706 | 39303296 | T | C | 0.544 | -0.012 | 0.0015 | 6.00E-15 | 0.00013 | 61 | body fat percentage |
| Hand grip strength (left) | rs11769549 | 23122239 | A | T | 0.062 | 0.020 | 0.0031 | 4.50E-11 | 0.00009 | 43 |  |
| Hand grip strength (left) | rs12533765 | 127699186 | G | A | 0.280 | -0.009 | 0.0017 | 2.60E-08 | 0.00007 | 31 |  |
| Hand grip strength (left) | rs16870531 | 120660682 | T | C | 0.238 | 0.011 | 0.0017 | 1.30E-10 | 0.00009 | 41 |  |
| Hand grip strength (left) | rs13227429 | 140560023 | C | T | 0.561 | -0.009 | 0.0015 | 9.80E-09 | 0.00007 | 33 |  |
| Hand grip strength (left) | rs6977081 | 150542515 | T | G | 0.334 | 0.015 | 0.0016 | 1.40E-20 | 0.00019 | 86 | body fat percentage |
| Hand grip strength (left) | rs12673062 | 4710677 | A | G | 0.216 | -0.011 | 0.0018 | 2.60E-09 | 0.00008 | 35 |  |
| Hand grip strength (left) | rs73307079 | 19020024 | C | T | 0.211 | 0.011 | 0.0018 | 1.40E-09 | 0.00008 | 37 | body fat percentage |
| Hand grip strength (left) | rs6962338 | 69160985 | G | A | 0.044 | -0.021 | 0.0036 | 3.20E-09 | 0.00008 | 35 |  |
| Hand grip strength (left) | rs17282763 | 82520166 | C | T | 0.296 | 0.009 | 0.0016 | 4.40E-08 | 0.00007 | 30 |  |
| Hand grip strength (left) | rs821100 | 89448877 | G | A | 0.266 | -0.010 | 0.0017 | 1.50E-09 | 0.00008 | 37 |  |
| Hand grip strength (left) | rs59116179 | 22603454 | T | C | 0.617 | 0.009 | 0.0015 | 2.40E-08 | 0.00007 | 31 |  |
| Hand grip strength (left) | rs4737446 | 57665019 | T | G | 0.695 | 0.010 | 0.0016 | 1.20E-10 | 0.00009 | 41 |  |
| Hand grip strength (left) | rs1486925 | 78827617 | C | T | 0.315 | -0.010 | 0.0016 | 6.30E-11 | 0.00009 | 43 |  |
| Hand grip strength (left) | rs4398863 | 135695110 | C | G | 0.737 | -0.009 | 0.0017 | 2.00E-08 | 0.00007 | 31 |  |
| Hand grip strength (left) | rs4739739 | 81304576 | G | A | 0.415 | -0.009 | 0.0015 | 1.40E-08 | 0.00007 | 32 |  |
| Hand grip strength (left) | rs10097417 | 110361477 | G | A | 0.171 | -0.013 | 0.0020 | 1.90E-11 | 0.00010 | 45 |  |
| Hand grip strength (left) | rs7026798 | 16427378 | C | T | 0.432 | 0.008 | 0.0015 | 4.60E-08 | 0.00006 | 30 |  |
| Hand grip strength (left) | rs7856625 | 119345083 | T | C | 0.610 | -0.011 | 0.0015 | 2.50E-13 | 0.00012 | 54 |  |
| Hand grip strength (left) | rs16910750 | 99084471 | C | G | 0.160 | 0.011 | 0.0020 | 3.60E-08 | 0.00007 | 30 |  |
| Hand grip strength (left) | rs116922558 | 118802375 | G | A | 0.040 | -0.022 | 0.0039 | 2.10E-08 | 0.00007 | 31 |  |
| Hand grip strength (left) | rs2789514 | 129833029 | A | G | 0.868 | 0.012 | 0.0022 | 4.90E-08 | 0.00006 | 30 |  |
| Hand grip strength (left) | rs10988217 | 131888116 | G | A | 0.604 | -0.009 | 0.0015 | 1.70E-09 | 0.00008 | 36 | BMI |
| Hand grip strength (left) | rs11002322 | 79649653 | T | G | 0.340 | -0.010 | 0.0016 | 2.10E-10 | 0.00009 | 40 |  |
| Hand grip strength (left) | rs10786706 | 104500659 | T | C | 0.466 | 0.010 | 0.0015 | 1.70E-11 | 0.00010 | 45 |  |
| Hand grip strength (left) | rs35236379 | 5727292 | T | G | 0.142 | 0.012 | 0.0021 | 5.80E-09 | 0.00007 | 34 |  |
| Hand grip strength (left) | rs11003014 | 81231387 | G | A | 0.161 | 0.011 | 0.0020 | 1.60E-08 | 0.00007 | 32 |  |
| Hand grip strength (left) | rs4962700 | 126479989 | G | C | 0.302 | 0.009 | 0.0016 | 3.20E-08 | 0.00007 | 31 |  |
| Hand grip strength (left) | rs772014 | 104062494 | G | A | 0.393 | -0.011 | 0.0015 | 2.70E-12 | 0.00011 | 49 |  |
| Hand grip strength (left) | rs10821939 | 63751748 | A | G | 0.573 | -0.009 | 0.0015 | 4.90E-10 | 0.00008 | 39 |  |
| Hand grip strength (left) | rs1556659 | 130834698 | T | C | 0.382 | 0.016 | 0.0015 | 2.50E-26 | 0.00024 | 113 |  |
| Hand grip strength (left) | rs72977282 | 74300441 | A | T | 0.414 | -0.016 | 0.0015 | 7.80E-25 | 0.00023 | 106 |  |
| Hand grip strength (left) | rs4930236 | 68414000 | A | C | 0.836 | 0.012 | 0.0020 | 3.80E-09 | 0.00008 | 35 |  |
| Hand grip strength (left) | rs10831903 | 12758660 | T | C | 0.423 | 0.009 | 0.0015 | 8.10E-10 | 0.00008 | 38 |  |
| Hand grip strength (left) | rs12790261 | 66988048 | A | C | 0.082 | -0.025 | 0.0027 | 1.20E-20 | 0.00019 | 87 |  |
| Hand grip strength (left) | rs7124681 | 47529947 | A | C | 0.408 | -0.012 | 0.0015 | 1.00E-14 | 0.00013 | 60 | BMI |
| Hand grip strength (left) | rs61389091 | 74427921 | T | C | 0.042 | 0.026 | 0.0037 | 2.40E-12 | 0.00011 | 49 |  |
| Hand grip strength (left) | rs34845616 | 133792644 | A | G | 0.246 | 0.011 | 0.0017 | 4.00E-10 | 0.00008 | 39 |  |
| Hand grip strength (left) | rs76895963 | 4384844 | G | T | 0.021 | 0.036 | 0.0057 | 3.90E-10 | 0.00008 | 39 | BMI |
| Hand grip strength (left) | rs10846071 | 15016236 | T | C | 0.394 | -0.017 | 0.0015 | 5.30E-28 | 0.00026 | 120 |  |
| Hand grip strength (left) | rs11168357 | 48412138 | A | G | 0.246 | -0.010 | 0.0017 | 2.50E-08 | 0.00007 | 31 |  |
| Hand grip strength (left) | rs4575361 | 124410529 | T | A | 0.312 | -0.011 | 0.0016 | 1.60E-11 | 0.00010 | 45 | body fat percentage |
| Hand grip strength (left) | rs12316046 | 15054415 | G | A | 0.378 | -0.017 | 0.0015 | 5.00E-30 | 0.00028 | 130 |  |
| Hand grip strength (left) | rs7970350 | 66360164 | T | C | 0.494 | -0.010 | 0.0015 | 8.60E-12 | 0.00010 | 47 | BMI |
| Hand grip strength (left) | rs7963801 | 79685226 | C | T | 0.572 | -0.010 | 0.0015 | 4.60E-12 | 0.00010 | 48 |  |
| Hand grip strength (left) | rs11111267 | 102811239 | G | A | 0.181 | 0.011 | 0.0019 | 1.90E-08 | 0.00007 | 32 |  |
| Hand grip strength (left) | rs3118903 | 51099577 | A | G | 0.220 | -0.017 | 0.0018 | 2.70E-22 | 0.00020 | 94 |  |
| Hand grip strength (left) | rs56060323 | 60473485 | T | C | 0.315 | 0.009 | 0.0016 | 1.50E-08 | 0.00007 | 32 |  |
| Hand grip strength (left) | rs12889267 | 21542766 | G | A | 0.167 | -0.014 | 0.0020 | 4.70E-12 | 0.00010 | 48 |  |
| Hand grip strength (left) | rs7148603 | 36683779 | A | G | 0.359 | 0.010 | 0.0016 | 1.60E-09 | 0.00008 | 36 |  |
| Hand grip strength (left) | rs2359239 | 75326771 | T | C | 0.392 | -0.009 | 0.0015 | 6.90E-09 | 0.00007 | 34 |  |
| Hand grip strength (left) | rs10144445 | 39695362 | G | C | 0.350 | -0.009 | 0.0016 | 1.90E-09 | 0.00008 | 36 |  |
| Hand grip strength (left) | rs28542042 | 74213357 | T | C | 0.309 | 0.011 | 0.0016 | 1.20E-11 | 0.00010 | 46 |  |
| Hand grip strength (left) | rs12906830 | 56963503 | C | T | 0.601 | 0.011 | 0.0015 | 9.30E-13 | 0.00011 | 51 |  |
| Hand grip strength (left) | rs3959716 | 73106615 | G | C | 0.567 | -0.008 | 0.0015 | 3.10E-08 | 0.00007 | 31 | BMI |
| Hand grip strength (left) | rs17466480 | 77390870 | G | A | 0.387 | -0.012 | 0.0015 | 9.80E-15 | 0.00013 | 60 |  |
| Hand grip strength (left) | rs12914702 | 96887277 | A | G | 0.300 | 0.011 | 0.0017 | 9.30E-11 | 0.00009 | 42 |  |
| Hand grip strength (left) | rs7176095 | 74886411 | G | A | 0.128 | -0.013 | 0.0022 | 1.70E-09 | 0.00008 | 36 |  |
| Hand grip strength (left) | rs2871865 | 99194896 | G | C | 0.116 | -0.022 | 0.0023 | 5.00E-21 | 0.00019 | 89 |  |
| Hand grip strength (left) | rs13337177 | 2175323 | T | G | 0.181 | -0.014 | 0.0019 | 1.60E-13 | 0.00012 | 54 | BMI |
| Hand grip strength (left) | rs11642954 | 24824248 | A | G | 0.195 | -0.012 | 0.0019 | 4.50E-10 | 0.00008 | 39 | BMI |
| Hand grip strength (left) | rs217181 | 72114002 | T | C | 0.193 | 0.012 | 0.0019 | 2.10E-10 | 0.00009 | 40 |  |
| Hand grip strength (left) | rs9944324 | 80929342 | G | A | 0.457 | -0.009 | 0.0015 | 1.20E-08 | 0.00007 | 32 |  |
| Hand grip strength (left) | rs7197751 | 84940033 | T | G | 0.363 | -0.009 | 0.0016 | 1.40E-09 | 0.00008 | 37 |  |
| Hand grip strength (left) | rs7196917 | 69896527 | G | A | 0.430 | -0.012 | 0.0015 | 5.40E-15 | 0.00013 | 61 | BMI |
| Hand grip strength (left) | rs181766 | 14394878 | C | T | 0.322 | 0.010 | 0.0016 | 1.90E-09 | 0.00008 | 36 |  |
| Hand grip strength (left) | rs3814877 | 30042677 | T | G | 0.402 | 0.011 | 0.0015 | 3.40E-12 | 0.00011 | 48 | BMI |
| Hand grip strength (left) | rs11076004 | 53913930 | A | G | 0.418 | -0.012 | 0.0015 | 2.10E-14 | 0.00013 | 58 |  |
| Hand grip strength (left) | rs113434679 | 44126765 | A | C | 0.200 | -0.015 | 0.0019 | 1.90E-15 | 0.00014 | 63 |  |
| Hand grip strength (left) | rs755547 | 43011908 | A | G | 0.189 | 0.017 | 0.0019 | 3.20E-18 | 0.00016 | 76 |  |
| Hand grip strength (left) | rs2532111 | 62017421 | G | A | 0.640 | 0.010 | 0.0016 | 4.10E-11 | 0.00009 | 44 | body fat percentage |
| Hand grip strength (left) | rs999493 | 46625519 | A | G | 0.622 | 0.013 | 0.0015 | 6.20E-17 | 0.00015 | 70 |  |
| Hand grip strength (left) | rs2587505 | 77784268 | C | T | 0.420 | -0.009 | 0.0015 | 2.30E-09 | 0.00008 | 36 | BMI |
| Hand grip strength (left) | rs635538 | 53273614 | A | G | 0.914 | -0.022 | 0.0027 | 3.80E-16 | 0.00014 | 66 |  |
| Hand grip strength (left) | rs4308051 | 20735461 | G | T | 0.789 | 0.016 | 0.0018 | 1.40E-18 | 0.00017 | 77 | waist hip ratio |
| Hand grip strength (left) | rs62081464 | 35142133 | T | C | 0.227 | -0.010 | 0.0018 | 2.80E-08 | 0.00007 | 31 |  |
| Hand grip strength (left) | rs143002906 | 12992162 | T | C | 0.028 | 0.026 | 0.0046 | 9.80E-09 | 0.00007 | 33 |  |
| Hand grip strength (left) | rs35054365 | 46612306 | A | T | 0.438 | 0.013 | 0.0015 | 1.20E-17 | 0.00016 | 73 |  |
| Hand grip strength (left) | rs10403906 | 37376756 | A | G | 0.476 | -0.010 | 0.0015 | 1.50E-11 | 0.00010 | 46 |  |
| Hand grip strength (left) | rs11669079 | 52219938 | T | A | 0.705 | 0.011 | 0.0016 | 1.90E-11 | 0.00010 | 45 |  |
| Hand grip strength (left) | rs8101782 | 12507992 | C | A | 0.703 | 0.010 | 0.0017 | 2.90E-08 | 0.00007 | 31 |  |
| Hand grip strength (left) | rs8108461 | 2186757 | C | T | 0.573 | 0.010 | 0.0015 | 2.60E-10 | 0.00009 | 40 |  |
| Hand grip strength (left) | rs143384 | 34025756 | G | A | 0.404 | 0.021 | 0.0015 | 1.50E-43 | 0.00042 | 192 | waist hip ratio |
| Hand grip strength (left) | rs4811040 | 48968438 | G | C | 0.277 | -0.009 | 0.0017 | 3.00E-08 | 0.00007 | 31 |  |
| Hand grip strength (left) | rs9611273 | 40534466 | T | C | 0.253 | 0.011 | 0.0017 | 4.50E-10 | 0.00008 | 39 |  |
| Hand grip strength (left) | rs6006984 | 45714937 | C | T | 0.278 | 0.010 | 0.0017 | 2.60E-09 | 0.00008 | 35 |  |
| Hand grip strength (left) | rs6680160 | 32072737 | G | A | 0.628 | 0.010 | 0.0015 | 6.00E-11 | 0.00009 | 43 |  |

Abbreviation: SNP, single nucleotide polymorphism; EA, Effect allele; NEA, Non-effect allele; EAF, effect allele frequency; SE, standard error; BMI, body mass index.

*^a^ R^2^* was calculated using the following formula: (2×EAF×(1-EAF)×beta^2^)/[(2×EAF×(1-EAF)×beta^2^)+(2×EAF×(1-EAF)×N×SE^2^)], where EAF is the effect allele

frequency, beta is the estimated effect on urate. Ν is the sample size of the GWAS for the SNP-urate association and SE is the standard error of the estimated effect.

*^b^ F* statistic was calculated using the following formula: *R^2^*(N-2)/(1-*R^2^*), where *R^2^* is the proportion of variance in urate explained by each instrument and N is the sample size of the GWAS for the SNP-urate association.

^c^ SNPs associated with confounding factors were removed after searching Phenoscanner database.

Supplementary Table 5 Characteristics of SNPs used as genetic instruments for appendicular lean mass

| Exposure | | SNP | Position | EA | NEA | EAF | SNP-Exposure association | | | R^2 a^ | F-statistic ^b^ | Confounders ^c^ |
| --- | --- | --- | --- | --- | --- | --- | --- | --- | --- | --- | --- | --- |
|  | |  |  |  |  |  | Beta | SE | P value |  |  |  |
| Appendicular lean mass | | rs200439 | 6716083 | C | A | 0.221 | -0.013 | 0.0023 | 1.51E-08 | 0.00007 | 31 |  |
| Appendicular lean mass | | rs2807339 | 22578063 | C | T | 0.759 | 0.016 | 0.0022 | 1.24E-13 | 0.00012 | 54 |  |
| Appendicular lean mass | | rs60804050 | 118870373 | A | G | 0.256 | -0.022 | 0.0021 | 5.01E-24 | 0.00024 | 107 |  |
| Appendicular lean mass | | rs2025808 | 184161757 | A | C | 0.254 | 0.012 | 0.0022 | 1.72E-08 | 0.00007 | 31 |  |
| Appendicular lean mass | | rs6693481 | 203766395 | C | T | 0.695 | -0.014 | 0.0020 | 2.21E-12 | 0.00011 | 51 |  |
| Appendicular lean mass | | rs61827272 | 203810763 | C | T | 0.275 | 0.014 | 0.0021 | 7.89E-12 | 0.00010 | 47 |  |
| Appendicular lean mass | | rs12724708 | 219620569 | T | A | 0.357 | 0.024 | 0.0020 | 1.66E-35 | 0.00033 | 148 | body fat percentage |
| Appendicular lean mass | | rs11260623 | 1781456 | T | G | 0.509 | 0.012 | 0.0019 | 4.86E-10 | 0.00008 | 38 |  |
| Appendicular lean mass | | rs301807 | 8484823 | G | A | 0.582 | -0.014 | 0.0019 | 2.53E-14 | 0.00013 | 57 | BMI |
| Appendicular lean mass | | rs212526 | 21584941 | C | T | 0.601 | 0.021 | 0.0019 | 3.84E-29 | 0.00028 | 127 |  |
| Appendicular lean mass | | rs7522400 | 36613380 | G | A | 0.768 | 0.013 | 0.0022 | 5.62E-09 | 0.00008 | 34 |  |
| Appendicular lean mass | | rs670318 | 63727542 | C | T | 0.952 | 0.041 | 0.0044 | 2.52E-21 | 0.00020 | 88 |  |
| Appendicular lean mass | | rs2025609 | 67422990 | G | C | 0.851 | 0.019 | 0.0026 | 2.00E-12 | 0.00011 | 51 |  |
| Appendicular lean mass | | rs7543202 | 73872885 | G | A | 0.623 | 0.013 | 0.0019 | 2.86E-11 | 0.00010 | 46 |  |
| Appendicular lean mass | | rs11590254 | 92316573 | T | A | 0.311 | 0.019 | 0.0020 | 4.34E-20 | 0.00019 | 86 |  |
| Appendicular lean mass | | rs4644481 | 155130900 | T | C | 0.433 | -0.011 | 0.0019 | 3.54E-09 | 0.00008 | 35 |  |
| Appendicular lean mass | | rs7367519 | 204479176 | C | T | 0.685 | 0.016 | 0.0020 | 4.68E-16 | 0.00015 | 67 |  |
| Appendicular lean mass | | rs951366 | 205685352 | C | T | 0.393 | 0.021 | 0.0019 | 9.15E-27 | 0.00026 | 116 |  |
| Appendicular lean mass | | rs7418410 | 10236402 | T | C | 0.409 | 0.016 | 0.0019 | 5.64E-16 | 0.00015 | 67 |  |
| Appendicular lean mass | | rs7543136 | 22472451 | T | C | 0.721 | -0.021 | 0.0021 | 9.96E-24 | 0.00022 | 100 |  |
| Appendicular lean mass | | rs4360494 | 38455891 | C | G | 0.554 | -0.020 | 0.0019 | 7.88E-26 | 0.00024 | 109 |  |
| Appendicular lean mass | | rs55717234 | 150999863 | G | A | 0.573 | 0.012 | 0.0019 | 1.34E-10 | 0.00009 | 41 |  |
| Appendicular lean mass | | rs11580040 | 155198222 | G | A | 0.080 | 0.033 | 0.0035 | 6.76E-21 | 0.00019 | 86 | body fat percentage |
| Appendicular lean mass | | rs2209098 | 172167226 | C | T | 0.311 | 0.024 | 0.0020 | 1.73E-32 | 0.00032 | 144 |  |
| Appendicular lean mass | | rs17278379 | 172381284 | C | T | 0.124 | 0.023 | 0.0029 | 2.40E-15 | 0.00013 | 61 |  |
| Appendicular lean mass | | rs4655345 | 214608704 | G | A | 0.397 | -0.025 | 0.0019 | 5.81E-38 | 0.00037 | 168 |  |
| Appendicular lean mass | | rs1797070 | 218630201 | A | G | 0.268 | 0.022 | 0.0021 | 4.93E-25 | 0.00024 | 109 |  |
| Appendicular lean mass | | rs12563442 | 19786695 | C | T | 0.267 | 0.012 | 0.0021 | 9.93E-09 | 0.00008 | 34 |  |
| Appendicular lean mass | | rs80295797 | 23341690 | T | C | 0.327 | -0.020 | 0.0020 | 3.83E-23 | 0.00022 | 98 |  |
| Appendicular lean mass | | rs4274112 | 26746199 | G | A | 0.373 | -0.022 | 0.0020 | 2.47E-28 | 0.00026 | 118 |  |
| Appendicular lean mass | | rs113107560 | 36747842 | G | T | 0.412 | -0.019 | 0.0019 | 6.99E-23 | 0.00022 | 100 |  |
| Appendicular lean mass | | rs11210892 | 44100084 | A | G | 0.675 | 0.012 | 0.0020 | 3.57E-09 | 0.00008 | 35 |  |
| Appendicular lean mass | | rs12074850 | 51248316 | G | A | 0.090 | 0.039 | 0.0033 | 2.72E-33 | 0.00031 | 142 |  |
| Appendicular lean mass | | rs1514134 | 56116513 | C | T | 0.385 | -0.011 | 0.0019 | 3.57E-09 | 0.00008 | 36 |  |
| Appendicular lean mass | | rs34517439 | 78450517 | A | C | 0.122 | 0.042 | 0.0029 | 5.80E-48 | 0.00047 | 211 | BMI |
| Appendicular lean mass | | rs10922475 | 89142142 | A | C | 0.540 | 0.016 | 0.0019 | 2.17E-17 | 0.00016 | 70 |  |
| Appendicular lean mass | | rs3768495 | 109935325 | T | C | 0.717 | -0.018 | 0.0021 | 1.07E-17 | 0.00016 | 72 |  |
| Appendicular lean mass | | rs28736838 | 120148713 | T | C | 0.302 | -0.012 | 0.0020 | 1.07E-08 | 0.00008 | 34 |  |
| Appendicular lean mass | | rs905938 | 154991389 | C | T | 0.265 | 0.039 | 0.0021 | 8.43E-77 | 0.00078 | 352 | BMI |
| Appendicular lean mass | | rs6675858 | 224559936 | T | C | 0.214 | -0.014 | 0.0023 | 2.40E-09 | 0.00008 | 35 |  |
| Appendicular lean mass | | rs2789365 | 235515534 | T | C | 0.481 | -0.015 | 0.0019 | 1.14E-14 | 0.00013 | 58 |  |
| Appendicular lean mass | | rs377599 | 2164699 | T | C | 0.383 | 0.022 | 0.0019 | 3.50E-29 | 0.00029 | 130 |  |
| Appendicular lean mass | | rs11121615 | 10825577 | T | C | 0.689 | -0.020 | 0.0020 | 3.32E-23 | 0.00023 | 102 |  |
| Appendicular lean mass | | rs2791654 | 11129317 | A | G | 0.706 | -0.024 | 0.0022 | 1.21E-28 | 0.00026 | 118 |  |
| Appendicular lean mass | | rs6425817 | 33873034 | G | A | 0.672 | 0.016 | 0.0020 | 2.98E-15 | 0.00014 | 62 |  |
| Appendicular lean mass | | rs2885697 | 41544279 | T | G | 0.665 | -0.032 | 0.0020 | 9.21E-60 | 0.00058 | 261 |  |
| Appendicular lean mass | | rs4847378 | 93324634 | T | G | 0.612 | 0.014 | 0.0019 | 1.64E-12 | 0.00011 | 51 |  |
| Appendicular lean mass | | rs1405227 | 98873390 | A | G | 0.317 | 0.013 | 0.0020 | 1.57E-10 | 0.00009 | 42 |  |
| Appendicular lean mass | | rs1325596 | 176794066 | A | G | 0.548 | 0.029 | 0.0019 | 2.77E-52 | 0.00051 | 228 |  |
| Appendicular lean mass | | rs234640 | 184867830 | T | C | 0.514 | -0.013 | 0.0019 | 3.87E-12 | 0.00011 | 48 |  |
| Appendicular lean mass | | rs1005723 | 243646251 | T | C | 0.191 | 0.016 | 0.0024 | 1.79E-11 | 0.00010 | 45 |  |
| Appendicular lean mass | | rs10171272 | 25946636 | A | C | 0.304 | 0.014 | 0.0020 | 2.87E-11 | 0.00010 | 46 |  |
| Appendicular lean mass | | rs17681189 | 65976175 | A | C | 0.423 | -0.013 | 0.0019 | 5.82E-12 | 0.00011 | 48 |  |
| Appendicular lean mass | | rs76517946 | 68354936 | A | C | 0.081 | -0.037 | 0.0035 | 1.69E-26 | 0.00025 | 111 |  |
| Appendicular lean mass | | rs867529 | 88913273 | C | G | 0.280 | 0.018 | 0.0021 | 1.00E-18 | 0.00017 | 77 |  |
| Appendicular lean mass | | rs6543146 | 103096695 | G | T | 0.558 | 0.015 | 0.0019 | 4.19E-16 | 0.00015 | 66 |  |
| Appendicular lean mass | | rs6738207 | 105989716 | A | G | 0.401 | 0.013 | 0.0019 | 4.14E-11 | 0.00010 | 45 |  |
| Appendicular lean mass | | rs71414738 | 127876242 | T | C | 0.176 | 0.015 | 0.0025 | 1.00E-09 | 0.00008 | 36 |  |
| Appendicular lean mass | | rs2390669 | 169091942 | C | A | 0.129 | 0.017 | 0.0028 | 7.00E-10 | 0.00009 | 39 | BMI |
| Appendicular lean mass | | rs13430869 | 218146818 | T | G | 0.743 | 0.027 | 0.0021 | 6.37E-37 | 0.00037 | 168 |  |
| Appendicular lean mass | | rs17408561 | 225474277 | G | A | 0.391 | 0.012 | 0.0020 | 6.00E-10 | 0.00008 | 38 |  |
| Appendicular lean mass | | rs17246129 | 227259964 | A | G | 0.304 | 0.025 | 0.0020 | 1.27E-35 | 0.00036 | 161 |  |
| Appendicular lean mass | | rs2305141 | 233684402 | G | A | 0.596 | 0.018 | 0.0019 | 1.07E-21 | 0.00021 | 93 |  |
| Appendicular lean mass | | rs2971857 | 234369487 | A | G | 0.575 | -0.012 | 0.0019 | 3.70E-10 | 0.00009 | 39 |  |
| Appendicular lean mass | | rs10203320 | 9771620 | C | T | 0.329 | 0.014 | 0.0020 | 7.86E-12 | 0.00011 | 48 | BMI |
| Appendicular lean mass | | rs144627572 | 20583907 | A | G | 0.033 | 0.044 | 0.0053 | 1.30E-16 | 0.00015 | 69 |  |
| Appendicular lean mass | | rs1260326 | 27730940 | C | T | 0.605 | 0.032 | 0.0019 | 6.16E-64 | 0.00064 | 289 |  |
| Appendicular lean mass | | rs650508 | 45880122 | C | G | 0.301 | -0.013 | 0.0020 | 1.86E-10 | 0.00009 | 42 |  |
| Appendicular lean mass | | rs75022676 | 60293216 | A | G | 0.208 | -0.016 | 0.0023 | 2.84E-12 | 0.00011 | 50 |  |
| Appendicular lean mass | | rs199647708 | 125352195 | A | G | 0.397 | 0.011 | 0.0019 | 3.43E-09 | 0.00008 | 36 |  |
| Appendicular lean mass | | rs17400325 | 178565913 | C | T | 0.042 | 0.035 | 0.0047 | 2.10E-13 | 0.00012 | 54 |  |
| Appendicular lean mass | | rs1035583 | 207326937 | A | G | 0.618 | 0.015 | 0.0019 | 1.99E-14 | 0.00013 | 61 |  |
| Appendicular lean mass | | rs7598430 | 219193963 | T | C | 0.505 | -0.016 | 0.0019 | 1.37E-17 | 0.00016 | 71 |  |
| Appendicular lean mass | | rs10202701 | 232328681 | T | C | 0.542 | 0.023 | 0.0019 | 3.11E-33 | 0.00032 | 143 |  |
| Appendicular lean mass | | rs10205141 | 11313340 | G | A | 0.048 | 0.024 | 0.0044 | 4.71E-08 | 0.00007 | 30 |  |
| Appendicular lean mass | | rs10203386 | 25136866 | A | T | 0.452 | -0.024 | 0.0019 | 1.75E-36 | 0.00035 | 157 | BMI |
| Appendicular lean mass | | rs10202845 | 42575820 | G | A | 0.113 | -0.029 | 0.0030 | 5.35E-22 | 0.00020 | 92 |  |
| Appendicular lean mass | | rs67716382 | 46890317 | C | G | 0.221 | 0.023 | 0.0023 | 1.65E-23 | 0.00021 | 97 | BMI |
| Appendicular lean mass | | rs2717008 | 58149158 | C | T | 0.384 | -0.013 | 0.0019 | 4.99E-11 | 0.00010 | 45 |  |
| Appendicular lean mass | | rs4852257 | 71678520 | G | T | 0.576 | -0.023 | 0.0019 | 6.21E-34 | 0.00033 | 148 |  |
| Appendicular lean mass | | rs201570119 | 112256320 | C | T | 0.210 | 0.019 | 0.0023 | 5.48E-17 | 0.00016 | 71 |  |
| Appendicular lean mass | | rs55852614 | 172416869 | C | T | 0.247 | -0.039 | 0.0022 | 3.29E-73 | 0.00071 | 319 |  |
| Appendicular lean mass | | rs700677 | 198702424 | A | C | 0.351 | 0.017 | 0.0020 | 1.13E-18 | 0.00017 | 75 | BMI |
| Appendicular lean mass | | rs12997625 | 202970250 | T | C | 0.527 | -0.017 | 0.0019 | 1.50E-19 | 0.00018 | 80 | body fat percentage |
| Appendicular lean mass | | rs3116194 | 233061266 | A | T | 0.098 | -0.030 | 0.0032 | 8.29E-21 | 0.00019 | 85 |  |
| Appendicular lean mass | | rs7570235 | 242491353 | C | T | 0.591 | -0.017 | 0.0019 | 2.08E-18 | 0.00017 | 78 |  |
| Appendicular lean mass | | rs7563362 | 620297 | G | A | 0.857 | 0.035 | 0.0027 | 3.27E-39 | 0.00038 | 170 | BMI |
| Appendicular lean mass | | rs6721191 | 10190115 | G | A | 0.578 | -0.014 | 0.0019 | 3.17E-14 | 0.00013 | 57 |  |
| Appendicular lean mass | | rs12713004 | 23896049 | G | A | 0.725 | 0.037 | 0.0021 | 2.40E-68 | 0.00068 | 305 |  |
| Appendicular lean mass | | rs6739278 | 44401055 | C | T | 0.808 | -0.021 | 0.0024 | 1.27E-18 | 0.00017 | 77 |  |
| Appendicular lean mass | | rs2347603 | 47297426 | A | T | 0.742 | -0.018 | 0.0022 | 5.65E-17 | 0.00015 | 68 | body fat percentage |
| Appendicular lean mass | | rs702886 | 65753310 | G | A | 0.351 | 0.012 | 0.0020 | 1.11E-09 | 0.00008 | 36 |  |
| Appendicular lean mass | | rs55980611 | 74771027 | A | C | 0.146 | 0.016 | 0.0028 | 4.22E-09 | 0.00008 | 34 |  |
| Appendicular lean mass | | rs14976 | 85818886 | T | C | 0.306 | 0.014 | 0.0020 | 1.43E-12 | 0.00012 | 52 |  |
| Appendicular lean mass | | rs72809820 | 97360079 | T | C | 0.324 | -0.011 | 0.0020 | 3.13E-08 | 0.00007 | 31 |  |
| Appendicular lean mass | | rs9636364 | 111992435 | A | G | 0.543 | 0.011 | 0.0019 | 5.09E-09 | 0.00007 | 34 |  |
| Appendicular lean mass | | rs10864899 | 112929481 | G | A | 0.564 | -0.011 | 0.0019 | 3.85E-09 | 0.00008 | 35 |  |
| Appendicular lean mass | | rs488621 | 169707552 | G | A | 0.469 | 0.019 | 0.0019 | 2.86E-24 | 0.00022 | 101 |  |
| Appendicular lean mass | | rs2138374 | 190014317 | C | T | 0.696 | -0.015 | 0.0020 | 2.79E-13 | 0.00012 | 56 |  |
| Appendicular lean mass | | rs10221831 | 202107829 | T | C | 0.032 | 0.030 | 0.0053 | 1.80E-08 | 0.00007 | 32 |  |
| Appendicular lean mass | | rs17773965 | 217631338 | T | C | 0.141 | -0.016 | 0.0027 | 1.51E-09 | 0.00008 | 36 |  |
| Appendicular lean mass | | rs62106258 | 417167 | C | T | 0.049 | -0.050 | 0.0044 | 6.45E-31 | 0.00029 | 131 | BMI |
| Appendicular lean mass | | rs3769598 | 32679732 | G | A | 0.144 | 0.017 | 0.0027 | 1.32E-10 | 0.00009 | 40 |  |
| Appendicular lean mass | | rs59985551 | 56106928 | T | C | 0.226 | -0.031 | 0.0022 | 2.43E-44 | 0.00045 | 202 |  |
| Appendicular lean mass | | rs62143873 | 72035050 | A | G | 0.503 | -0.012 | 0.0019 | 1.19E-09 | 0.00008 | 37 |  |
| Appendicular lean mass | | rs12616192 | 121568931 | A | G | 0.068 | -0.026 | 0.0038 | 6.83E-12 | 0.00010 | 47 |  |
| Appendicular lean mass | | rs61397287 | 144223279 | T | A | 0.075 | 0.024 | 0.0036 | 4.45E-11 | 0.00009 | 43 |  |
| Appendicular lean mass | | rs13391980 | 165504841 | A | G | 0.120 | -0.023 | 0.0029 | 7.60E-15 | 0.00013 | 60 | BMI |
| Appendicular lean mass | | rs1047891 | 211540507 | A | C | 0.316 | 0.023 | 0.0020 | 5.70E-31 | 0.00030 | 136 | BMI |
| Appendicular lean mass | | rs1478575 | 218278555 | A | T | 0.684 | 0.031 | 0.0020 | 5.20E-54 | 0.00054 | 243 |  |
| Appendicular lean mass | | rs11684531 | 219835489 | G | A | 0.133 | -0.017 | 0.0028 | 4.17E-10 | 0.00008 | 38 |  |
| Appendicular lean mass | | rs1899040 | 223901896 | T | C | 0.796 | 0.015 | 0.0023 | 9.04E-11 | 0.00010 | 44 |  |
| Appendicular lean mass | | rs2270894 | 9975386 | G | C | 0.203 | -0.033 | 0.0024 | 1.25E-42 | 0.00042 | 191 |  |
| Appendicular lean mass | | rs113671109 | 12620885 | C | T | 0.220 | -0.015 | 0.0023 | 4.23E-11 | 0.00009 | 43 |  |
| Appendicular lean mass | | rs6789000 | 25188002 | T | G | 0.645 | 0.012 | 0.0020 | 1.61E-09 | 0.00008 | 37 |  |
| Appendicular lean mass | | rs4504126 | 33600582 | C | A | 0.028 | 0.046 | 0.0058 | 1.62E-15 | 0.00014 | 63 |  |
| Appendicular lean mass | | rs140440099 | 50632595 | A | G | 0.023 | 0.061 | 0.0063 | 1.45E-22 | 0.00021 | 95 |  |
| Appendicular lean mass | | rs17718736 | 71555205 | A | C | 0.323 | 0.012 | 0.0020 | 1.39E-08 | 0.00007 | 33 |  |
| Appendicular lean mass | | rs4682483 | 112993982 | A | G | 0.153 | -0.017 | 0.0026 | 2.65E-10 | 0.00009 | 40 |  |
| Appendicular lean mass | | rs4683435 | 142624732 | G | A | 0.772 | 0.014 | 0.0022 | 1.60E-10 | 0.00010 | 43 |  |
| Appendicular lean mass | | rs900399 | 156798732 | G | A | 0.399 | 0.016 | 0.0019 | 1.35E-17 | 0.00017 | 75 | waist hip ratio |
| Appendicular lean mass | | rs1290786 | 169097381 | T | C | 0.431 | -0.014 | 0.0019 | 7.14E-14 | 0.00013 | 57 |  |
| Appendicular lean mass | | rs9647379 | 171785168 | C | G | 0.411 | 0.022 | 0.0019 | 5.55E-29 | 0.00028 | 128 |  |
| Appendicular lean mass | | rs2194411 | 185548663 | A | G | 0.128 | 0.044 | 0.0029 | 2.43E-54 | 0.00052 | 233 | BMI |
| Appendicular lean mass | | rs11720869 | 185619716 | A | G | 0.669 | 0.014 | 0.0020 | 2.54E-12 | 0.00011 | 50 |  |
| Appendicular lean mass | | rs73052033 | 185828465 | C | T | 0.185 | -0.015 | 0.0024 | 4.79E-10 | 0.00009 | 40 | BMI |
| Appendicular lean mass | | rs336630 | 18607538 | T | C | 0.430 | -0.011 | 0.0019 | 2.90E-08 | 0.00007 | 31 |  |
| Appendicular lean mass | | rs9838614 | 38537671 | G | T | 0.388 | -0.019 | 0.0019 | 1.21E-21 | 0.00021 | 95 |  |
| Appendicular lean mass | | rs6762851 | 56686329 | C | T | 0.357 | -0.022 | 0.0020 | 1.50E-28 | 0.00026 | 119 |  |
| Appendicular lean mass | | rs182798714 | 128960869 | T | A | 0.027 | 0.038 | 0.0062 | 1.45E-09 | 0.00008 | 37 |  |
| Appendicular lean mass | | rs591668 | 27535931 | A | G | 0.396 | -0.017 | 0.0019 | 2.00E-19 | 0.00019 | 84 |  |
| Appendicular lean mass | | rs36012032 | 52814709 | A | C | 0.091 | 0.030 | 0.0033 | 9.93E-20 | 0.00018 | 82 |  |
| Appendicular lean mass | | rs839255 | 57974580 | G | T | 0.685 | -0.013 | 0.0021 | 8.38E-10 | 0.00008 | 36 |  |
| Appendicular lean mass | | rs9809116 | 72397279 | G | A | 0.408 | -0.016 | 0.0019 | 1.31E-16 | 0.00016 | 71 |  |
| Appendicular lean mass | | rs7633464 | 98715823 | A | G | 0.479 | 0.018 | 0.0019 | 1.27E-20 | 0.00019 | 85 |  |
| Appendicular lean mass | | rs115010283 | 172162393 | C | G | 0.316 | 0.034 | 0.0020 | 2.35E-63 | 0.00064 | 289 |  |
| Appendicular lean mass | | rs61732778 | 187443314 | A | G | 0.071 | 0.023 | 0.0037 | 3.13E-10 | 0.00009 | 39 |  |
| Appendicular lean mass | | rs4076108 | 13736088 | T | A | 0.245 | 0.017 | 0.0022 | 2.30E-15 | 0.00014 | 63 |  |
| Appendicular lean mass | | rs34312629 | 24079795 | G | C | 0.261 | -0.017 | 0.0021 | 2.12E-15 | 0.00015 | 66 |  |
| Appendicular lean mass | | rs200739311 | 85650323 | C | T | 0.619 | -0.013 | 0.0020 | 1.55E-10 | 0.00009 | 41 |  |
| Appendicular lean mass | | rs544136 | 101041229 | G | T | 0.748 | 0.012 | 0.0022 | 2.50E-08 | 0.00007 | 30 | body fat percentage |
| Appendicular lean mass | | rs4073154 | 129035485 | G | A | 0.778 | 0.027 | 0.0023 | 1.92E-33 | 0.00032 | 142 |  |
| Appendicular lean mass | | rs2871960 | 141121814 | C | A | 0.445 | 0.047 | 0.0019 | 2.17E-135 | 0.00135 | 609 |  |
| Appendicular lean mass | | rs1730028 | 157900789 | G | T | 0.417 | 0.013 | 0.0019 | 7.39E-12 | 0.00011 | 48 | BMI |
| Appendicular lean mass | | rs71635721 | 171960170 | G | C | 0.065 | 0.032 | 0.0039 | 3.46E-16 | 0.00015 | 66 |  |
| Appendicular lean mass | | rs34390533 | 184030838 | A | C | 0.248 | -0.026 | 0.0022 | 6.69E-32 | 0.00030 | 136 |  |
| Appendicular lean mass | | rs7610055 | 12388409 | A | G | 0.121 | -0.037 | 0.0029 | 3.55E-38 | 0.00037 | 165 | body fat percentage |
| Appendicular lean mass | | rs56239180 | 32937951 | G | T | 0.024 | -0.046 | 0.0062 | 1.85E-13 | 0.00012 | 55 |  |
| Appendicular lean mass | | rs9828525 | 61552810 | T | C | 0.411 | 0.012 | 0.0019 | 2.51E-10 | 0.00009 | 41 |  |
| Appendicular lean mass | | rs116493405 | 114733556 | A | G | 0.054 | 0.029 | 0.0042 | 9.52E-12 | 0.00010 | 47 |  |
| Appendicular lean mass | | rs9832919 | 132184526 | G | A | 0.357 | -0.018 | 0.0020 | 7.90E-20 | 0.00018 | 80 |  |
| Appendicular lean mass | | rs1823217 | 134380959 | G | A | 0.645 | -0.018 | 0.0020 | 4.06E-20 | 0.00018 | 82 |  |
| Appendicular lean mass | | rs113289555 | 996998 | T | G | 0.233 | -0.021 | 0.0023 | 7.33E-20 | 0.00018 | 80 |  |
| Appendicular lean mass | | rs13127468 | 8599658 | A | C | 0.463 | -0.012 | 0.0019 | 9.86E-11 | 0.00009 | 42 |  |
| Appendicular lean mass | | rs10005035 | 12865684 | G | C | 0.282 | -0.018 | 0.0021 | 6.51E-17 | 0.00015 | 69 |  |
| Appendicular lean mass | | rs1472852 | 17910236 | A | C | 0.158 | -0.064 | 0.0026 | 8.22E-135 | 0.00134 | 602 | body fat percentage |
| Appendicular lean mass | | rs963317 | 45129970 | G | A | 0.663 | -0.014 | 0.0020 | 1.03E-11 | 0.00010 | 46 | BMI |
| Appendicular lean mass | | rs781669 | 57819794 | T | C | 0.524 | 0.016 | 0.0019 | 3.14E-18 | 0.00017 | 75 |  |
| Appendicular lean mass | | rs13103161 | 106216459 | A | T | 0.389 | -0.028 | 0.0019 | 2.38E-48 | 0.00050 | 223 |  |
| Appendicular lean mass | | rs6849302 | 156665074 | G | A | 0.198 | 0.016 | 0.0024 | 7.11E-11 | 0.00009 | 42 |  |
| Appendicular lean mass | | rs1443536 | 82174165 | G | A | 0.305 | 0.022 | 0.0021 | 1.91E-26 | 0.00024 | 108 |  |
| Appendicular lean mass | | rs72657800 | 90822051 | C | T | 0.077 | -0.022 | 0.0035 | 6.39E-10 | 0.00009 | 39 |  |
| Appendicular lean mass | | rs11098677 | 123833516 | T | G | 0.788 | -0.026 | 0.0023 | 3.94E-30 | 0.00029 | 131 |  |
| Appendicular lean mass | | rs12512942 | 177766307 | A | G | 0.638 | -0.016 | 0.0020 | 1.58E-16 | 0.00015 | 66 |  |
| Appendicular lean mass | | rs59950280 | 3452345 | A | G | 0.332 | -0.025 | 0.0020 | 7.32E-36 | 0.00036 | 161 |  |
| Appendicular lean mass | | rs10019221 | 21785364 | T | G | 0.597 | -0.012 | 0.0019 | 1.22E-10 | 0.00009 | 43 |  |
| Appendicular lean mass | | rs3103223 | 42402721 | C | T | 0.740 | 0.013 | 0.0022 | 6.02E-09 | 0.00007 | 33 |  |
| Appendicular lean mass | | rs139921635 | 73181637 | T | G | 0.024 | 0.039 | 0.0062 | 6.16E-10 | 0.00009 | 39 |  |
| Appendicular lean mass | | rs13123591 | 120105990 | G | T | 0.338 | 0.019 | 0.0020 | 2.35E-20 | 0.00019 | 86 |  |
| Appendicular lean mass | | rs6821305 | 122713863 | C | A | 0.399 | 0.020 | 0.0019 | 3.15E-26 | 0.00026 | 115 |  |
| Appendicular lean mass | | rs72695791 | 184059452 | G | C | 0.036 | -0.030 | 0.0051 | 4.58E-09 | 0.00008 | 34 |  |
| Appendicular lean mass | | rs2324154 | 24027226 | A | C | 0.510 | 0.015 | 0.0019 | 1.92E-15 | 0.00014 | 62 |  |
| Appendicular lean mass | | rs116339650 | 26200972 | G | A | 0.127 | -0.018 | 0.0029 | 1.05E-09 | 0.00008 | 36 |  |
| Appendicular lean mass | | rs2303423 | 38120029 | C | T | 0.110 | 0.017 | 0.0030 | 2.64E-08 | 0.00007 | 31 |  |
| Appendicular lean mass | | rs13109280 | 54380513 | G | A | 0.662 | 0.013 | 0.0020 | 9.15E-11 | 0.00010 | 43 |  |
| Appendicular lean mass | | rs116052377 | 124787756 | A | G | 0.081 | 0.023 | 0.0035 | 7.89E-11 | 0.00009 | 41 |  |
| Appendicular lean mass | | rs7689420 | 145568352 | C | T | 0.831 | 0.047 | 0.0025 | 1.50E-76 | 0.00077 | 347 |  |
| Appendicular lean mass | | rs11721522 | 156976051 | G | A | 0.413 | 0.011 | 0.0019 | 4.03E-08 | 0.00007 | 31 |  |
| Appendicular lean mass | | rs73856768 | 157788804 | C | T | 0.081 | -0.025 | 0.0035 | 1.55E-12 | 0.00011 | 50 |  |
| Appendicular lean mass | | rs111622870 | 2613109 | C | T | 0.048 | -0.028 | 0.0044 | 1.86E-10 | 0.00009 | 41 | body fat percentage |
| Appendicular lean mass | | rs190823861 | 73505911 | A | G | 0.047 | -0.035 | 0.0045 | 2.09E-14 | 0.00013 | 59 |  |
| Appendicular lean mass | | rs11727162 | 88606761 | T | C | 0.499 | -0.017 | 0.0019 | 2.15E-19 | 0.00018 | 80 |  |
| Appendicular lean mass | | rs2035901 | 145521867 | G | A | 0.468 | 0.024 | 0.0019 | 9.43E-37 | 0.00035 | 160 |  |
| Appendicular lean mass | | rs7679276 | 146860186 | G | A | 0.954 | -0.033 | 0.0048 | 5.93E-12 | 0.00010 | 47 |  |
| Appendicular lean mass | | rs395980 | 177430072 | G | T | 0.264 | -0.018 | 0.0021 | 1.02E-17 | 0.00017 | 77 |  |
| Appendicular lean mass | | rs2578565 | 5460569 | T | C | 0.658 | -0.014 | 0.0020 | 1.37E-12 | 0.00011 | 50 |  |
| Appendicular lean mass | | rs12655296 | 15890643 | T | C | 0.625 | -0.011 | 0.0020 | 1.62E-08 | 0.00007 | 30 |  |
| Appendicular lean mass | | rs1177765 | 32829929 | C | T | 0.468 | -0.023 | 0.0019 | 1.32E-34 | 0.00033 | 149 |  |
| Appendicular lean mass | | rs11959466 | 42803824 | T | C | 0.056 | 0.038 | 0.0042 | 2.24E-19 | 0.00018 | 82 |  |
| Appendicular lean mass | | rs62370472 | 52767109 | C | T | 0.209 | -0.025 | 0.0023 | 1.45E-27 | 0.00027 | 121 |  |
| Appendicular lean mass | | rs10471339 | 67823773 | G | C | 0.382 | -0.011 | 0.0019 | 1.45E-08 | 0.00007 | 34 |  |
| Appendicular lean mass | | rs36048468 | 122879901 | T | C | 0.209 | 0.025 | 0.0023 | 9.34E-28 | 0.00027 | 122 |  |
| Appendicular lean mass | | rs7735891 | 131597005 | T | C | 0.463 | 0.026 | 0.0019 | 1.14E-42 | 0.00041 | 186 |  |
| Appendicular lean mass | | rs55758152 | 171317318 | A | G | 0.326 | 0.015 | 0.0020 | 1.05E-12 | 0.00012 | 53 |  |
| Appendicular lean mass | | rs57059662 | 33217275 | C | T | 0.677 | 0.012 | 0.0020 | 5.63E-09 | 0.00008 | 35 |  |
| Appendicular lean mass | | rs10461725 | 39437129 | C | G | 0.656 | 0.013 | 0.0020 | 1.98E-11 | 0.00010 | 45 |  |
| Appendicular lean mass | | rs4865956 | 54882505 | A | T | 0.697 | -0.026 | 0.0021 | 3.85E-36 | 0.00034 | 151 |  |
| Appendicular lean mass | | rs12517711 | 60754661 | C | T | 0.392 | -0.015 | 0.0019 | 2.79E-14 | 0.00013 | 60 |  |
| Appendicular lean mass | | rs12188208 | 77442791 | C | A | 0.235 | -0.020 | 0.0022 | 1.40E-18 | 0.00017 | 79 | BMI |
| Appendicular lean mass | | rs115912456 | 82815158 | G | A | 0.041 | 0.058 | 0.0047 | 3.69E-34 | 0.00033 | 151 | body fat percentage |
| Appendicular lean mass | | rs861674 | 112064475 | T | A | 0.458 | 0.013 | 0.0019 | 1.36E-11 | 0.00010 | 45 |  |
| Appendicular lean mass | | rs10068640 | 123981977 | A | G | 0.365 | 0.011 | 0.0020 | 1.28E-08 | 0.00007 | 31 |  |
| Appendicular lean mass | | rs2545339 | 149911219 | G | A | 0.629 | 0.012 | 0.0020 | 3.48E-09 | 0.00007 | 33 |  |
| Appendicular lean mass | | rs31196 | 158300798 | A | C | 0.572 | -0.011 | 0.0019 | 2.07E-08 | 0.00007 | 32 |  |
| Appendicular lean mass | | rs111365325 | 170865229 | T | C | 0.231 | -0.027 | 0.0022 | 1.12E-33 | 0.00034 | 152 |  |
| Appendicular lean mass | | rs6874142 | 172753555 | G | T | 0.114 | 0.029 | 0.0031 | 5.15E-20 | 0.00019 | 86 |  |
| Appendicular lean mass | | rs10075249 | 52846505 | T | C | 0.495 | 0.014 | 0.0019 | 4.56E-14 | 0.00013 | 57 | body fat percentage |
| Appendicular lean mass | | rs10036789 | 71695918 | G | C | 0.461 | 0.016 | 0.0019 | 1.16E-17 | 0.00016 | 74 |  |
| Appendicular lean mass | | rs261223 | 95901046 | C | A | 0.370 | 0.018 | 0.0019 | 2.30E-19 | 0.00019 | 85 |  |
| Appendicular lean mass | | rs12519407 | 137651012 | C | A | 0.257 | 0.018 | 0.0022 | 3.38E-17 | 0.00015 | 68 |  |
| Appendicular lean mass | | rs249677 | 141539339 | A | C | 0.633 | -0.011 | 0.0020 | 2.40E-08 | 0.00007 | 30 |  |
| Appendicular lean mass | | rs13170063 | 157895013 | A | G | 0.592 | -0.015 | 0.0019 | 4.11E-15 | 0.00014 | 64 |  |
| Appendicular lean mass | | rs447352 | 678750 | T | C | 0.141 | -0.018 | 0.0029 | 6.62E-10 | 0.00009 | 39 |  |
| Appendicular lean mass | | rs7731023 | 36181627 | G | A | 0.575 | 0.017 | 0.0019 | 3.48E-18 | 0.00017 | 76 |  |
| Appendicular lean mass | | rs7448554 | 95711603 | A | C | 0.500 | -0.013 | 0.0020 | 1.77E-11 | 0.00010 | 44 |  |
| Appendicular lean mass | | rs3822742 | 139059017 | A | C | 0.371 | 0.016 | 0.0020 | 1.03E-16 | 0.00015 | 66 | BMI |
| Appendicular lean mass | | rs4282339 | 168256240 | A | G | 0.208 | -0.031 | 0.0023 | 6.16E-41 | 0.00041 | 183 |  |
| Appendicular lean mass | | rs244711 | 176509193 | T | C | 0.686 | 0.028 | 0.0022 | 1.54E-37 | 0.00036 | 161 |  |
| Appendicular lean mass | | rs40270 | 55804552 | C | A | 0.772 | 0.015 | 0.0022 | 1.90E-11 | 0.00010 | 47 | body fat percentage |
| Appendicular lean mass | | rs34287 | 67585143 | A | G | 0.341 | 0.019 | 0.0020 | 1.17E-20 | 0.00019 | 87 |  |
| Appendicular lean mass | | rs331917 | 98158524 | G | A | 0.580 | -0.013 | 0.0019 | 3.54E-11 | 0.00010 | 45 |  |
| Appendicular lean mass | | rs6860245 | 127367998 | C | G | 0.248 | 0.059 | 0.0022 | 9.66E-160 | 0.00159 | 717 | body fat percentage |
| Appendicular lean mass | | rs4976262 | 134379531 | C | T | 0.316 | -0.025 | 0.0020 | 4.37E-33 | 0.00033 | 150 |  |
| Appendicular lean mass | | rs258794 | 142540040 | T | G | 0.276 | 0.015 | 0.0021 | 6.23E-12 | 0.00011 | 49 |  |
| Appendicular lean mass | | rs7701233 | 171218388 | C | T | 0.428 | -0.018 | 0.0019 | 5.12E-21 | 0.00020 | 89 |  |
| Appendicular lean mass | | rs3792819 | 172576296 | G | A | 0.085 | 0.021 | 0.0034 | 4.42E-10 | 0.00008 | 38 |  |
| Appendicular lean mass | | rs11243202 | 7719065 | C | T | 0.486 | 0.030 | 0.0019 | 2.83E-57 | 0.00056 | 253 |  |
| Appendicular lean mass | | rs13209685 | 7779729 | T | G | 0.159 | 0.028 | 0.0026 | 7.49E-27 | 0.00025 | 114 |  |
| Appendicular lean mass | | rs2142644 | 19053843 | A | C | 0.672 | -0.018 | 0.0020 | 3.37E-19 | 0.00018 | 82 |  |
| Appendicular lean mass | | rs78000963 | 30502802 | A | G | 0.104 | 0.017 | 0.0031 | 4.39E-08 | 0.00007 | 30 |  |
| Appendicular lean mass | | rs2268718 | 52415023 | T | C | 0.270 | 0.014 | 0.0021 | 3.24E-11 | 0.00010 | 45 |  |
| Appendicular lean mass | | rs6931421 | 80880138 | G | T | 0.322 | -0.028 | 0.0020 | 2.31E-43 | 0.00043 | 195 |  |
| Appendicular lean mass | | rs9375188 | 98555272 | T | C | 0.484 | 0.014 | 0.0019 | 6.80E-13 | 0.00011 | 51 | BMI |
| Appendicular lean mass | | rs9391254 | 105377347 | T | C | 0.321 | 0.017 | 0.0020 | 2.10E-16 | 0.00015 | 69 |  |
| Appendicular lean mass | | rs113898003 | 130341235 | C | T | 0.264 | -0.036 | 0.0021 | 1.19E-63 | 0.00065 | 294 |  |
| Appendicular lean mass | | rs1933081 | 151651505 | A | T | 0.083 | 0.027 | 0.0034 | 5.33E-15 | 0.00014 | 62 |  |
| Appendicular lean mass | | rs9385002 | 117552469 | T | A | 0.239 | -0.015 | 0.0022 | 3.26E-11 | 0.00010 | 45 |  |
| Appendicular lean mass | | rs7768382 | 166341870 | C | T | 0.477 | -0.020 | 0.0019 | 1.57E-26 | 0.00025 | 112 |  |
| Appendicular lean mass | | rs2788213 | 703249 | A | G | 0.710 | 0.012 | 0.0021 | 3.80E-09 | 0.00008 | 34 |  |
| Appendicular lean mass | | rs2569888 | 1625803 | A | G | 0.245 | 0.013 | 0.0022 | 2.29E-09 | 0.00008 | 37 |  |
| Appendicular lean mass | | rs876122 | 6886297 | G | A | 0.879 | 0.016 | 0.0029 | 2.19E-08 | 0.00007 | 31 |  |
| Appendicular lean mass | | rs41271299 | 19839415 | T | C | 0.051 | 0.062 | 0.0043 | 3.19E-47 | 0.00046 | 205 |  |
| Appendicular lean mass | | rs188617336 | 20610730 | T | C | 0.298 | 0.014 | 0.0021 | 6.66E-11 | 0.00010 | 43 |  |
| Appendicular lean mass | | rs4380799 | 32571864 | G | T | 0.390 | -0.026 | 0.0021 | 6.45E-33 | 0.00033 | 147 |  |
| Appendicular lean mass | | rs1319012 | 41852616 | A | T | 0.926 | -0.052 | 0.0037 | 3.30E-45 | 0.00044 | 198 |  |
| Appendicular lean mass | | rs1324538 | 45080144 | A | T | 0.384 | 0.024 | 0.0019 | 1.73E-34 | 0.00035 | 156 |  |
| Appendicular lean mass | | rs655113 | 52269151 | C | T | 0.301 | 0.019 | 0.0021 | 7.42E-20 | 0.00018 | 80 |  |
| Appendicular lean mass | | rs9344126 | 81907559 | C | T | 0.514 | -0.019 | 0.0019 | 2.21E-22 | 0.00021 | 95 |  |
| Appendicular lean mass | | rs293517 | 83662455 | C | T | 0.701 | -0.013 | 0.0021 | 2.80E-10 | 0.00009 | 38 |  |
| Appendicular lean mass | | rs7768973 | 109745325 | A | T | 0.411 | -0.024 | 0.0019 | 5.44E-36 | 0.00035 | 160 |  |
| Appendicular lean mass | | rs78051210 | 131379491 | C | T | 0.077 | 0.026 | 0.0036 | 1.63E-13 | 0.00012 | 53 |  |
| Appendicular lean mass | | rs10807137 | 34183026 | T | C | 0.825 | -0.046 | 0.0025 | 5.68E-75 | 0.00074 | 331 | BMI |
| Appendicular lean mass | | rs9343327 | 76606296 | T | A | 0.499 | 0.014 | 0.0019 | 1.09E-13 | 0.00012 | 54 |  |
| Appendicular lean mass | | rs2754255 | 88393572 | G | A | 0.225 | -0.015 | 0.0023 | 1.05E-11 | 0.00010 | 44 |  |
| Appendicular lean mass | | rs9388490 | 126704795 | T | C | 0.439 | 0.046 | 0.0019 | 1.33E-130 | 0.00131 | 591 | body fat percentage |
| Appendicular lean mass | | rs6902109 | 130316559 | G | A | 0.540 | -0.017 | 0.0019 | 1.03E-18 | 0.00017 | 77 |  |
| Appendicular lean mass | | rs599004 | 140439740 | T | C | 0.281 | -0.016 | 0.0021 | 6.53E-14 | 0.00012 | 56 |  |
| Appendicular lean mass | | rs2748501 | 146312258 | A | G | 0.440 | -0.020 | 0.0019 | 1.31E-24 | 0.00023 | 105 |  |
| Appendicular lean mass | | rs718603 | 2644245 | T | C | 0.277 | 0.013 | 0.0021 | 6.68E-10 | 0.00009 | 39 |  |
| Appendicular lean mass | | rs9266244 | 31325692 | A | G | 0.708 | -0.043 | 0.0021 | 1.21E-94 | 0.00092 | 413 |  |
| Appendicular lean mass | | rs72894003 | 34775096 | T | C | 0.065 | -0.042 | 0.0038 | 1.90E-28 | 0.00028 | 124 |  |
| Appendicular lean mass | | rs2764264 | 108934461 | T | C | 0.695 | 0.020 | 0.0021 | 5.07E-23 | 0.00021 | 93 | BMI |
| Appendicular lean mass | | rs6570509 | 142716286 | T | G | 0.287 | -0.024 | 0.0021 | 1.27E-31 | 0.00030 | 135 |  |
| Appendicular lean mass | | rs543650 | 152110943 | G | T | 0.599 | 0.025 | 0.0020 | 1.49E-37 | 0.00035 | 156 |  |
| Appendicular lean mass | | rs3828729 | 155554707 | G | A | 0.309 | -0.016 | 0.0020 | 4.67E-15 | 0.00014 | 64 |  |
| Appendicular lean mass | | rs2105333 | 158755437 | G | T | 0.665 | -0.019 | 0.0020 | 1.70E-21 | 0.00020 | 90 |  |
| Appendicular lean mass | | rs2763263 | 168814392 | A | T | 0.245 | -0.017 | 0.0022 | 1.37E-14 | 0.00013 | 60 |  |
| Appendicular lean mass | | rs798548 | 2760935 | C | T | 0.301 | -0.036 | 0.0021 | 2.86E-68 | 0.00065 | 292 | body fat percentage |
| Appendicular lean mass | | rs12533452 | 19016871 | T | C | 0.157 | 0.024 | 0.0026 | 1.31E-19 | 0.00018 | 83 | body fat percentage |
| Appendicular lean mass | | rs34776209 | 23513093 | T | C | 0.248 | -0.032 | 0.0022 | 1.78E-47 | 0.00046 | 208 |  |
| Appendicular lean mass | | rs12536902 | 33213009 | A | C | 0.014 | 0.048 | 0.0081 | 3.66E-09 | 0.00008 | 35 |  |
| Appendicular lean mass | | rs1880318 | 46028167 | A | G | 0.204 | 0.015 | 0.0024 | 6.86E-10 | 0.00008 | 38 |  |
| Appendicular lean mass | | rs73696333 | 46669400 | G | C | 0.201 | 0.019 | 0.0024 | 3.20E-15 | 0.00014 | 63 |  |
| Appendicular lean mass | | rs35732917 | 73013269 | C | T | 0.284 | 0.020 | 0.0021 | 2.08E-22 | 0.00021 | 94 |  |
| Appendicular lean mass | | rs139163241 | 76709213 | G | T | 0.139 | -0.016 | 0.0027 | 1.79E-09 | 0.00008 | 37 |  |
| Appendicular lean mass | | rs62466110 | 92623541 | C | T | 0.067 | -0.037 | 0.0041 | 5.74E-20 | 0.00018 | 82 |  |
| Appendicular lean mass | | rs987666 | 116267938 | A | G | 0.119 | 0.019 | 0.0029 | 2.33E-10 | 0.00009 | 41 |  |
| Appendicular lean mass | | rs62621812 | 127015083 | A | G | 0.020 | 0.074 | 0.0069 | 3.16E-27 | 0.00026 | 116 | BMI |
| Appendicular lean mass | | rs757834 | 139717200 | C | T | 0.186 | 0.026 | 0.0024 | 1.25E-25 | 0.00025 | 114 |  |
| Appendicular lean mass | | rs28529426 | 4678264 | T | C | 0.171 | -0.017 | 0.0026 | 5.82E-11 | 0.00009 | 42 |  |
| Appendicular lean mass | | rs10242866 | 17920613 | T | C | 0.398 | 0.016 | 0.0019 | 3.66E-16 | 0.00015 | 68 |  |
| Appendicular lean mass | | rs2529090 | 24662280 | G | C | 0.181 | 0.014 | 0.0025 | 3.39E-08 | 0.00007 | 30 |  |
| Appendicular lean mass | | rs2237485 | 50749870 | A | G | 0.223 | 0.019 | 0.0023 | 3.73E-17 | 0.00015 | 69 |  |
| Appendicular lean mass | | rs12672217 | 156310948 | A | G | 0.361 | 0.014 | 0.0020 | 1.33E-12 | 0.00011 | 48 |  |
| Appendicular lean mass | | rs177591 | 28556199 | G | C | 0.149 | -0.019 | 0.0027 | 1.51E-12 | 0.00011 | 50 | body fat percentage |
| Appendicular lean mass | | rs60408354 | 70158495 | A | G | 0.073 | 0.026 | 0.0036 | 1.15E-12 | 0.00011 | 52 |  |
| Appendicular lean mass | | rs42039 | 92244422 | T | C | 0.244 | 0.048 | 0.0022 | 3.53E-106 | 0.00106 | 478 |  |
| Appendicular lean mass | | rs56363908 | 96611052 | G | A | 0.042 | -0.038 | 0.0047 | 3.88E-16 | 0.00015 | 66 | body fat percentage |
| Appendicular lean mass | | rs2140619 | 114007270 | G | A | 0.413 | 0.011 | 0.0019 | 5.18E-09 | 0.00008 | 35 |  |
| Appendicular lean mass | | rs3778858 | 129963356 | T | G | 0.370 | 0.011 | 0.0020 | 4.26E-08 | 0.00006 | 29 |  |
| Appendicular lean mass | | rs822530 | 148631555 | T | A | 0.795 | 0.026 | 0.0024 | 2.36E-27 | 0.00025 | 113 |  |
| Appendicular lean mass | | rs12702693 | 8101039 | T | C | 0.454 | 0.017 | 0.0019 | 6.56E-20 | 0.00018 | 83 |  |
| Appendicular lean mass | | rs680882 | 18325278 | G | T | 0.761 | 0.013 | 0.0022 | 1.98E-09 | 0.00008 | 37 |  |
| Appendicular lean mass | | rs723149 | 46577056 | G | A | 0.563 | -0.028 | 0.0019 | 1.43E-47 | 0.00047 | 211 |  |
| Appendicular lean mass | | rs1202186 | 87213258 | T | C | 0.655 | -0.012 | 0.0020 | 1.65E-09 | 0.00008 | 36 |  |
| Appendicular lean mass | | rs9640283 | 150485659 | C | G | 0.518 | -0.012 | 0.0019 | 4.53E-10 | 0.00009 | 39 |  |
| Appendicular lean mass | | rs6977416 | 150542711 | A | G | 0.334 | 0.046 | 0.0020 | 1.43E-113 | 0.00116 | 522 | body fat percentage |
| Appendicular lean mass | | rs10225945 | 28250083 | G | A | 0.150 | -0.015 | 0.0026 | 3.28E-08 | 0.00007 | 32 |  |
| Appendicular lean mass | | rs12700901 | 28783171 | A | C | 0.406 | -0.018 | 0.0019 | 1.67E-21 | 0.00021 | 94 |  |
| Appendicular lean mass | | rs6593210 | 55254186 | A | G | 0.208 | 0.015 | 0.0024 | 4.88E-10 | 0.00008 | 37 |  |
| Appendicular lean mass | | rs11562101 | 56059458 | T | A | 0.458 | 0.012 | 0.0020 | 9.39E-09 | 0.00007 | 33 |  |
| Appendicular lean mass | | rs2188805 | 93078400 | C | A | 0.336 | 0.011 | 0.0020 | 2.00E-08 | 0.00007 | 32 |  |
| Appendicular lean mass | | rs76364830 | 13372120 | A | G | 0.063 | -0.047 | 0.0039 | 2.70E-33 | 0.00032 | 146 |  |
| Appendicular lean mass | | rs7826059 | 22512068 | C | T | 0.642 | 0.011 | 0.0020 | 7.61E-09 | 0.00007 | 32 |  |
| Appendicular lean mass | | rs1063582 | 23167353 | G | T | 0.765 | -0.019 | 0.0022 | 1.12E-16 | 0.00016 | 71 |  |
| Appendicular lean mass | | rs117818446 | 67223589 | A | G | 0.020 | 0.042 | 0.0068 | 5.29E-10 | 0.00009 | 39 |  |
| Appendicular lean mass | | rs7014590 | 89335647 | C | T | 0.261 | -0.023 | 0.0022 | 4.48E-26 | 0.00024 | 107 |  |
| Appendicular lean mass | | rs112537273 | 38248306 | C | T | 0.230 | -0.021 | 0.0022 | 3.34E-21 | 0.00021 | 93 |  |
| Appendicular lean mass | | rs4602848 | 92186933 | G | A | 0.681 | 0.016 | 0.0020 | 3.14E-15 | 0.00014 | 64 |  |
| Appendicular lean mass | | rs2142331 | 116636719 | T | C | 0.602 | -0.017 | 0.0019 | 1.38E-17 | 0.00017 | 75 |  |
| Appendicular lean mass | | rs6470771 | 130743726 | C | A | 0.169 | -0.027 | 0.0025 | 1.54E-26 | 0.00026 | 115 |  |
| Appendicular lean mass | | rs12334478 | 141998765 | G | C | 0.496 | -0.016 | 0.0019 | 2.27E-17 | 0.00016 | 72 |  |
| Appendicular lean mass | | rs10112506 | 13164746 | G | A | 0.390 | -0.012 | 0.0019 | 5.76E-10 | 0.00009 | 40 |  |
| Appendicular lean mass | | rs7816345 | 36846109 | T | C | 0.168 | 0.026 | 0.0025 | 6.22E-24 | 0.00023 | 104 | body fat percentage |
| Appendicular lean mass | | rs2923411 | 42455206 | C | T | 0.595 | 0.013 | 0.0019 | 4.77E-11 | 0.00010 | 45 |  |
| Appendicular lean mass | | rs72656010 | 57122215 | C | T | 0.132 | -0.067 | 0.0028 | 7.31E-126 | 0.00126 | 569 |  |
| Appendicular lean mass | | rs7828086 | 120843775 | C | T | 0.239 | 0.014 | 0.0022 | 1.11E-09 | 0.00008 | 38 |  |
| Appendicular lean mass | | rs62501195 | 24041988 | C | A | 0.171 | -0.020 | 0.0025 | 8.11E-15 | 0.00014 | 63 |  |
| Appendicular lean mass | | rs62515437 | 57160328 | T | G | 0.225 | 0.037 | 0.0023 | 8.79E-60 | 0.00057 | 257 |  |
| Appendicular lean mass | | rs2925155 | 75886297 | T | C | 0.261 | -0.015 | 0.0022 | 5.47E-12 | 0.00010 | 46 |  |
| Appendicular lean mass | | rs11778491 | 120451362 | C | G | 0.252 | -0.025 | 0.0022 | 8.39E-30 | 0.00028 | 126 |  |
| Appendicular lean mass | | rs1340022 | 131334465 | C | T | 0.487 | 0.012 | 0.0019 | 4.47E-10 | 0.00009 | 39 |  |
| Appendicular lean mass | | rs72721979 | 135827942 | G | T | 0.143 | -0.023 | 0.0027 | 2.20E-17 | 0.00016 | 72 |  |
| Appendicular lean mass | | rs7007389 | 25355022 | A | T | 0.373 | -0.013 | 0.0020 | 2.94E-11 | 0.00010 | 45 |  |
| Appendicular lean mass | | rs115105539 | 49409496 | C | G | 0.167 | 0.023 | 0.0025 | 7.83E-20 | 0.00019 | 85 |  |
| Appendicular lean mass | | rs4077103 | 49557732 | A | C | 0.843 | -0.014 | 0.0026 | 4.18E-08 | 0.00007 | 30 |  |
| Appendicular lean mass | | rs61729527 | 77761919 | T | C | 0.052 | -0.035 | 0.0043 | 4.86E-16 | 0.00014 | 65 | BMI |
| Appendicular lean mass | | rs4735761 | 78097161 | C | A | 0.286 | 0.033 | 0.0021 | 3.66E-56 | 0.00055 | 248 |  |
| Appendicular lean mass | | rs10283100 | 120596023 | G | A | 0.945 | 0.058 | 0.0041 | 4.11E-44 | 0.00044 | 197 |  |
| Appendicular lean mass | | rs4870941 | 126498828 | C | G | 0.238 | -0.030 | 0.0023 | 1.09E-39 | 0.00037 | 167 |  |
| Appendicular lean mass | | rs12541381 | 135649848 | A | G | 0.258 | -0.032 | 0.0022 | 2.81E-49 | 0.00047 | 210 |  |
| Appendicular lean mass | | rs10107388 | 145004944 | C | T | 0.369 | -0.016 | 0.0020 | 6.95E-16 | 0.00014 | 63 |  |
| Appendicular lean mass | | rs10815274 | 5728968 | C | A | 0.456 | 0.012 | 0.0019 | 6.46E-11 | 0.00009 | 43 |  |
| Appendicular lean mass | | rs7858712 | 16738312 | G | A | 0.914 | 0.035 | 0.0034 | 1.04E-24 | 0.00023 | 104 |  |
| Appendicular lean mass | | rs34522021 | 23350420 | T | C | 0.455 | 0.013 | 0.0019 | 3.38E-11 | 0.00010 | 44 |  |
| Appendicular lean mass | | rs75508358 | 96926382 | T | C | 0.046 | 0.027 | 0.0045 | 4.29E-09 | 0.00008 | 35 |  |
| Appendicular lean mass | | rs12347137 | 119122721 | C | A | 0.202 | -0.046 | 0.0024 | 9.80E-85 | 0.00082 | 367 |  |
| Appendicular lean mass | | rs10123619 | 119353611 | G | A | 0.843 | -0.017 | 0.0026 | 4.05E-11 | 0.00010 | 43 |  |
| Appendicular lean mass | | rs10975935 | 6954579 | G | A | 0.246 | -0.012 | 0.0022 | 4.15E-08 | 0.00007 | 30 |  |
| Appendicular lean mass | | rs1056747 | 35690102 | G | A | 0.412 | -0.016 | 0.0019 | 8.05E-16 | 0.00015 | 67 |  |
| Appendicular lean mass | | rs143554698 | 95538573 | T | C | 0.141 | -0.026 | 0.0027 | 3.39E-21 | 0.00020 | 91 |  |
| Appendicular lean mass | | rs10982888 | 118468947 | A | T | 0.114 | -0.033 | 0.0030 | 3.97E-28 | 0.00027 | 120 |  |
| Appendicular lean mass | | rs73384223 | 3869315 | C | T | 0.197 | -0.021 | 0.0024 | 1.27E-17 | 0.00016 | 73 |  |
| Appendicular lean mass | | rs10962212 | 15911745 | C | G | 0.418 | 0.014 | 0.0019 | 7.47E-14 | 0.00013 | 57 | BMI |
| Appendicular lean mass | | rs7863102 | 73963468 | T | A | 0.447 | -0.011 | 0.0019 | 9.97E-09 | 0.00007 | 34 |  |
| Appendicular lean mass | | rs3901421 | 96204538 | C | G | 0.487 | 0.022 | 0.0019 | 7.55E-30 | 0.00028 | 128 |  |
| Appendicular lean mass | | rs2236406 | 98221861 | C | T | 0.349 | 0.039 | 0.0020 | 1.26E-87 | 0.00086 | 388 |  |
| Appendicular lean mass | | rs3205136 | 136126631 | A | C | 0.095 | -0.018 | 0.0033 | 1.85E-08 | 0.00007 | 31 |  |
| Appendicular lean mass | | rs10858246 | 139102831 | C | G | 0.318 | -0.019 | 0.0020 | 2.35E-20 | 0.00020 | 88 |  |
| Appendicular lean mass | | rs1330826 | 85129970 | C | G | 0.227 | 0.016 | 0.0023 | 1.04E-12 | 0.00011 | 50 |  |
| Appendicular lean mass | | rs7020491 | 128144477 | T | C | 0.427 | -0.018 | 0.0019 | 1.22E-20 | 0.00019 | 88 | BMI |
| Appendicular lean mass | | rs74458759 | 136940614 | G | C | 0.289 | 0.017 | 0.0022 | 3.12E-15 | 0.00013 | 60 |  |
| Appendicular lean mass | | rs12340775 | 13226945 | A | G | 0.055 | -0.029 | 0.0043 | 1.73E-11 | 0.00010 | 45 |  |
| Appendicular lean mass | | rs12351226 | 98405230 | T | C | 0.171 | 0.022 | 0.0025 | 9.13E-18 | 0.00017 | 76 |  |
| Appendicular lean mass | | rs1341215 | 111662350 | A | G | 0.137 | 0.023 | 0.0027 | 6.32E-17 | 0.00016 | 72 |  |
| Appendicular lean mass | | rs12344515 | 113801231 | T | C | 0.240 | -0.016 | 0.0022 | 2.28E-13 | 0.00012 | 55 |  |
| Appendicular lean mass | | rs80280630 | 117030861 | T | C | 0.112 | -0.017 | 0.0030 | 2.28E-08 | 0.00007 | 31 |  |
| Appendicular lean mass | | rs10793931 | 133436478 | C | G | 0.356 | -0.013 | 0.0020 | 3.23E-11 | 0.00010 | 44 |  |
| Appendicular lean mass | | rs7082659 | 12017584 | C | T | 0.866 | 0.016 | 0.0028 | 2.27E-08 | 0.00007 | 31 |  |
| Appendicular lean mass | | rs10829226 | 27573952 | A | G | 0.636 | -0.011 | 0.0020 | 1.33E-08 | 0.00007 | 31 |  |
| Appendicular lean mass | | rs10776560 | 50542358 | T | C | 0.500 | -0.016 | 0.0019 | 7.88E-17 | 0.00015 | 68 |  |
| Appendicular lean mass | | rs68049170 | 72432047 | A | G | 0.276 | -0.026 | 0.0021 | 2.69E-34 | 0.00034 | 152 | body fat percentage |
| Appendicular lean mass | | rs2274351 | 104264107 | T | C | 0.543 | 0.017 | 0.0019 | 3.07E-19 | 0.00018 | 80 |  |
| Appendicular lean mass | | rs72841270 | 104642237 | G | T | 0.135 | 0.029 | 0.0028 | 2.25E-26 | 0.00024 | 110 | BMI |
| Appendicular lean mass | | rs1556659 | 130834698 | T | C | 0.382 | 0.016 | 0.0020 | 7.19E-17 | 0.00015 | 66 |  |
| Appendicular lean mass | | rs35288270 | 4961278 | C | T | 0.134 | -0.033 | 0.0028 | 3.43E-32 | 0.00030 | 137 |  |
| Appendicular lean mass | | rs4748008 | 12935125 | C | T | 0.435 | -0.013 | 0.0019 | 8.95E-11 | 0.00010 | 43 |  |
| Appendicular lean mass | | rs332116 | 28926099 | T | C | 0.280 | -0.021 | 0.0021 | 2.89E-22 | 0.00021 | 96 |  |
| Appendicular lean mass | | rs10822117 | 52786701 | G | A | 0.237 | -0.018 | 0.0022 | 4.24E-15 | 0.00014 | 64 |  |
| Appendicular lean mass | | rs67527161 | 63781824 | C | T | 0.209 | -0.018 | 0.0023 | 5.79E-15 | 0.00014 | 63 |  |
| Appendicular lean mass | | rs10128333 | 64570038 | T | C | 0.168 | -0.015 | 0.0025 | 9.51E-09 | 0.00008 | 34 |  |
| Appendicular lean mass | | rs7095472 | 70399109 | G | A | 0.534 | 0.027 | 0.0019 | 7.66E-45 | 0.00044 | 197 |  |
| Appendicular lean mass | | rs117335233 | 79914330 | G | T | 0.053 | -0.024 | 0.0042 | 2.56E-08 | 0.00007 | 32 |  |
| Appendicular lean mass | | rs12773500 | 81232632 | T | C | 0.138 | 0.017 | 0.0028 | 5.05E-10 | 0.00008 | 37 |  |
| Appendicular lean mass | | rs11187838 | 96038686 | A | G | 0.435 | 0.039 | 0.0019 | 1.16E-94 | 0.00095 | 430 | body fat percentage |
| Appendicular lean mass | | rs2181834 | 102661251 | T | G | 0.550 | 0.025 | 0.0019 | 7.69E-41 | 0.00040 | 179 |  |
| Appendicular lean mass | | rs496783 | 116137961 | G | A | 0.465 | -0.012 | 0.0019 | 8.13E-11 | 0.00009 | 43 |  |
| Appendicular lean mass | | rs11198591 | 120515892 | A | G | 0.368 | 0.015 | 0.0020 | 4.91E-14 | 0.00012 | 55 |  |
| Appendicular lean mass | | rs2362487 | 126208402 | G | C | 0.247 | 0.015 | 0.0022 | 3.57E-12 | 0.00011 | 49 |  |
| Appendicular lean mass | | rs947099 | 31129883 | A | G | 0.354 | 0.012 | 0.0020 | 2.71E-09 | 0.00008 | 34 |  |
| Appendicular lean mass | | rs10824307 | 77185310 | C | G | 0.647 | -0.019 | 0.0020 | 1.79E-22 | 0.00021 | 94 |  |
| Appendicular lean mass | | rs664317 | 89812230 | C | A | 0.838 | -0.018 | 0.0026 | 4.50E-12 | 0.00010 | 46 |  |
| Appendicular lean mass | | rs2648725 | 93015079 | A | T | 0.213 | 0.017 | 0.0023 | 8.20E-13 | 0.00011 | 51 |  |
| Appendicular lean mass | | rs7893378 | 93634095 | A | G | 0.114 | 0.018 | 0.0031 | 2.36E-08 | 0.00007 | 32 |  |
| Appendicular lean mass | | rs291979 | 121129797 | A | G | 0.229 | 0.024 | 0.0023 | 6.76E-27 | 0.00025 | 111 |  |
| Appendicular lean mass | | rs11009928 | 35058712 | G | A | 0.255 | -0.015 | 0.0022 | 1.04E-11 | 0.00010 | 45 |  |
| Appendicular lean mass | | rs4752689 | 124131176 | A | G | 0.584 | 0.021 | 0.0019 | 1.36E-26 | 0.00026 | 116 |  |
| Appendicular lean mass | | rs11014285 | 25178864 | A | G | 0.165 | 0.034 | 0.0026 | 2.89E-40 | 0.00038 | 173 |  |
| Appendicular lean mass | | rs2490302 | 37702435 | A | T | 0.914 | 0.022 | 0.0034 | 6.24E-11 | 0.00009 | 42 |  |
| Appendicular lean mass | | rs11191208 | 103838497 | A | G | 0.206 | 0.015 | 0.0024 | 3.69E-10 | 0.00008 | 38 |  |
| Appendicular lean mass | | rs10749157 | 115780129 | C | T | 0.358 | 0.011 | 0.0020 | 1.24E-08 | 0.00007 | 32 |  |
| Appendicular lean mass | | rs2283200 | 2729340 | T | C | 0.056 | -0.028 | 0.0042 | 1.48E-11 | 0.00010 | 45 |  |
| Appendicular lean mass | | rs73413540 | 3090976 | T | C | 0.225 | -0.012 | 0.0023 | 4.42E-08 | 0.00006 | 29 |  |
| Appendicular lean mass | | rs985136 | 17497794 | G | C | 0.490 | 0.014 | 0.0020 | 2.96E-12 | 0.00011 | 48 |  |
| Appendicular lean mass | | rs4752829 | 47396654 | A | G | 0.286 | 0.026 | 0.0021 | 5.90E-36 | 0.00035 | 156 |  |
| Appendicular lean mass | | rs10796828 | 69490346 | G | T | 0.635 | 0.015 | 0.0020 | 5.77E-15 | 0.00013 | 59 | BMI |
| Appendicular lean mass | | rs7902 | 95565288 | G | A | 0.447 | 0.015 | 0.0019 | 5.22E-15 | 0.00014 | 61 |  |
| Appendicular lean mass | | rs11221657 | 129181358 | G | T | 0.135 | 0.018 | 0.0028 | 1.21E-10 | 0.00009 | 41 |  |
| Appendicular lean mass | | rs112873218 | 1960119 | T | C | 0.105 | 0.022 | 0.0031 | 4.02E-12 | 0.00011 | 49 |  |
| Appendicular lean mass | | rs10832963 | 18664241 | G | T | 0.745 | -0.020 | 0.0022 | 9.23E-21 | 0.00019 | 85 |  |
| Appendicular lean mass | | rs704660 | 30447998 | T | C | 0.410 | 0.015 | 0.0019 | 2.28E-15 | 0.00014 | 65 |  |
| Appendicular lean mass | | rs7107356 | 47676170 | G | A | 0.507 | 0.013 | 0.0019 | 1.86E-12 | 0.00011 | 49 | BMI |
| Appendicular lean mass | | rs11233117 | 69924352 | G | C | 0.454 | -0.018 | 0.0019 | 1.74E-20 | 0.00019 | 86 |  |
| Appendicular lean mass | | rs73006226 | 108072728 | A | C | 0.127 | -0.018 | 0.0029 | 2.23E-10 | 0.00009 | 39 |  |
| Appendicular lean mass | | rs545104 | 118591352 | C | T | 0.616 | 0.013 | 0.0020 | 8.08E-11 | 0.00009 | 40 |  |
| Appendicular lean mass | | rs56207600 | 126196537 | A | G | 0.111 | 0.019 | 0.0030 | 2.50E-10 | 0.00009 | 41 |  |
| Appendicular lean mass | | rs61878760 | 12807189 | A | G | 0.083 | 0.019 | 0.0034 | 3.74E-08 | 0.00007 | 31 |  |
| Appendicular lean mass | | rs7952436 | 67024534 | T | C | 0.082 | -0.045 | 0.0034 | 1.62E-39 | 0.00039 | 178 |  |
| Appendicular lean mass | | rs4244809 | 2164333 | A | G | 0.208 | -0.026 | 0.0023 | 5.55E-29 | 0.00029 | 130 |  |
| Appendicular lean mass | | rs1584011 | 27080527 | G | T | 0.356 | 0.016 | 0.0020 | 9.78E-16 | 0.00014 | 63 |  |
| Appendicular lean mass | | rs7941305 | 28652116 | C | T | 0.313 | -0.013 | 0.0021 | 6.01E-10 | 0.00008 | 38 |  |
| Appendicular lean mass | | rs11605297 | 58296806 | A | G | 0.233 | 0.015 | 0.0022 | 8.03E-11 | 0.00010 | 44 |  |
| Appendicular lean mass | | rs7129320 | 68388220 | A | G | 0.166 | -0.039 | 0.0025 | 7.29E-53 | 0.00054 | 242 |  |
| Appendicular lean mass | | rs34345560 | 69081998 | A | G | 0.195 | 0.022 | 0.0024 | 7.10E-20 | 0.00018 | 83 |  |
| Appendicular lean mass | | rs604723 | 100610546 | C | T | 0.725 | -0.017 | 0.0021 | 8.16E-15 | 0.00014 | 62 |  |
| Appendicular lean mass | | rs11042717 | 10303939 | C | T | 0.490 | -0.029 | 0.0019 | 4.04E-53 | 0.00052 | 233 | body fat percentage |
| Appendicular lean mass | | rs10657263 | 49690460 | G | C | 0.548 | -0.013 | 0.0019 | 7.98E-12 | 0.00010 | 47 |  |
| Appendicular lean mass | | rs4938359 | 117093560 | G | A | 0.202 | -0.016 | 0.0024 | 3.24E-11 | 0.00009 | 42 |  |
| Appendicular lean mass | | rs11217863 | 120293138 | A | G | 0.116 | -0.027 | 0.0030 | 1.06E-19 | 0.00018 | 80 |  |
| Appendicular lean mass | | rs772222 | 52356892 | G | A | 0.265 | 0.012 | 0.0021 | 1.55E-08 | 0.00007 | 33 |  |
| Appendicular lean mass | | rs1168768 | 66509650 | T | C | 0.975 | 0.033 | 0.0060 | 3.61E-08 | 0.00007 | 31 |  |
| Appendicular lean mass | | rs2089111 | 91180019 | G | C | 0.267 | -0.017 | 0.0022 | 1.73E-15 | 0.00014 | 61 |  |
| Appendicular lean mass | | rs4622329 | 102321935 | A | G | 0.350 | 0.015 | 0.0020 | 8.62E-14 | 0.00012 | 56 |  |
| Appendicular lean mass | | rs3764002 | 108618630 | T | C | 0.262 | 0.028 | 0.0021 | 4.47E-39 | 0.00039 | 178 | body fat percentage |
| Appendicular lean mass | | rs12423821 | 132650284 | C | T | 0.158 | 0.016 | 0.0027 | 1.17E-09 | 0.00008 | 36 |  |
| Appendicular lean mass | | rs7137546 | 577237 | T | A | 0.425 | 0.014 | 0.0019 | 9.71E-14 | 0.00012 | 56 |  |
| Appendicular lean mass | | rs67551338 | 3393100 | T | C | 0.062 | 0.058 | 0.0040 | 1.04E-47 | 0.00046 | 207 |  |
| Appendicular lean mass | | rs10845408 | 11880581 | T | C | 0.354 | 0.026 | 0.0020 | 3.25E-38 | 0.00036 | 163 |  |
| Appendicular lean mass | | rs17478946 | 24093062 | G | A | 0.300 | -0.019 | 0.0021 | 9.98E-21 | 0.00019 | 84 |  |
| Appendicular lean mass | | rs12230946 | 53498725 | A | G | 0.091 | 0.027 | 0.0033 | 1.45E-16 | 0.00015 | 67 |  |
| Appendicular lean mass | | rs9669278 | 66374587 | C | T | 0.518 | -0.050 | 0.0019 | 5.25E-151 | 0.00151 | 681 |  |
| Appendicular lean mass | | rs2229840 | 124826462 | T | C | 0.160 | 0.034 | 0.0026 | 3.02E-40 | 0.00038 | 172 |  |
| Appendicular lean mass | | rs7485647 | 131631133 | A | G | 0.844 | -0.026 | 0.0026 | 1.04E-23 | 0.00022 | 101 |  |
| Appendicular lean mass | | rs35756741 | 12868701 | T | C | 0.092 | -0.038 | 0.0033 | 5.80E-31 | 0.00029 | 131 |  |
| Appendicular lean mass | | rs6582398 | 42870444 | T | C | 0.600 | 0.014 | 0.0020 | 1.14E-12 | 0.00011 | 49 |  |
| Appendicular lean mass | | rs10748128 | 69827658 | T | G | 0.345 | 0.026 | 0.0020 | 6.77E-38 | 0.00036 | 163 |  |
| Appendicular lean mass | | rs11178643 | 71522437 | T | A | 0.359 | 0.011 | 0.0020 | 4.44E-08 | 0.00007 | 30 |  |
| Appendicular lean mass | | rs310796 | 77453226 | T | G | 0.681 | 0.014 | 0.0020 | 2.53E-12 | 0.00011 | 50 |  |
| Appendicular lean mass | | rs9634212 | 93993266 | A | C | 0.221 | 0.047 | 0.0023 | 8.59E-95 | 0.00093 | 419 |  |
| Appendicular lean mass | | rs7971536 | 102373788 | A | T | 0.495 | -0.019 | 0.0019 | 1.06E-24 | 0.00023 | 104 |  |
| Appendicular lean mass | | rs2454390 | 103255613 | C | T | 0.846 | -0.018 | 0.0026 | 1.74E-11 | 0.00010 | 46 |  |
| Appendicular lean mass | | rs11612462 | 104411368 | G | T | 0.170 | 0.015 | 0.0025 | 2.55E-09 | 0.00008 | 36 |  |
| Appendicular lean mass | | rs34338597 | 106301580 | G | A | 0.383 | -0.011 | 0.0019 | 7.88E-09 | 0.00008 | 35 |  |
| Appendicular lean mass | | rs3184504 | 111884608 | C | T | 0.517 | 0.018 | 0.0019 | 2.71E-22 | 0.00021 | 93 | BMI |
| Appendicular lean mass | | rs610694 | 121304826 | C | T | 0.490 | 0.014 | 0.0019 | 4.29E-13 | 0.00011 | 51 |  |
| Appendicular lean mass | | rs76895963 | 4384844 | G | T | 0.021 | 0.164 | 0.0073 | 8.22E-112 | 0.00112 | 504 | BMI |
| Appendicular lean mass | | rs11175919 | 66180277 | A | G | 0.026 | 0.035 | 0.0059 | 3.16E-09 | 0.00008 | 35 |  |
| Appendicular lean mass | | rs11068230 | 117349014 | G | C | 0.866 | 0.024 | 0.0028 | 9.67E-18 | 0.00016 | 72 |  |
| Appendicular lean mass | | rs2101017 | 122306857 | T | C | 0.870 | -0.022 | 0.0028 | 1.44E-15 | 0.00014 | 63 |  |
| Appendicular lean mass | | rs11060942 | 123434524 | A | G | 0.034 | -0.035 | 0.0052 | 7.49E-12 | 0.00010 | 46 |  |
| Appendicular lean mass | | rs28592876 | 123866429 | A | G | 0.205 | 0.030 | 0.0023 | 9.09E-38 | 0.00038 | 170 |  |
| Appendicular lean mass | | rs3782811 | 3339927 | A | C | 0.251 | -0.017 | 0.0022 | 3.96E-14 | 0.00012 | 56 |  |
| Appendicular lean mass | | rs61919240 | 8831954 | A | T | 0.324 | 0.014 | 0.0020 | 9.77E-12 | 0.00010 | 47 |  |
| Appendicular lean mass | | rs1444628 | 20563643 | T | C | 0.690 | 0.024 | 0.0020 | 6.85E-32 | 0.00032 | 144 |  |
| Appendicular lean mass | | rs11049704 | 28691701 | G | C | 0.293 | -0.018 | 0.0021 | 1.10E-18 | 0.00017 | 76 |  |
| Appendicular lean mass | | rs12831751 | 29520017 | C | A | 0.286 | 0.017 | 0.0021 | 1.57E-16 | 0.00015 | 67 |  |
| Appendicular lean mass | | rs12099669 | 46783653 | A | G | 0.696 | 0.033 | 0.0020 | 1.39E-58 | 0.00061 | 274 |  |
| Appendicular lean mass | | rs2071450 | 54428532 | T | C | 0.368 | -0.017 | 0.0020 | 8.85E-19 | 0.00017 | 76 | body fat percentage |
| Appendicular lean mass | | rs3782232 | 57116249 | A | G | 0.071 | -0.034 | 0.0037 | 2.41E-20 | 0.00019 | 84 |  |
| Appendicular lean mass | | rs7301341 | 94083105 | C | T | 0.327 | -0.026 | 0.0020 | 9.27E-37 | 0.00036 | 163 |  |
| Appendicular lean mass | | rs7321635 | 21472055 | C | A | 0.647 | -0.013 | 0.0020 | 2.58E-11 | 0.00010 | 44 |  |
| Appendicular lean mass | | rs3116602 | 51111355 | G | T | 0.215 | -0.061 | 0.0023 | 9.53E-155 | 0.00157 | 708 | waist hip ratio |
| Appendicular lean mass | | rs3818416 | 78474468 | C | A | 0.765 | 0.028 | 0.0022 | 2.01E-35 | 0.00036 | 161 |  |
| Appendicular lean mass | | rs61944841 | 27049616 | A | G | 0.414 | 0.025 | 0.0020 | 3.54E-37 | 0.00036 | 160 |  |
| Appendicular lean mass | | rs77013652 | 51142279 | G | T | 0.015 | 0.049 | 0.0081 | 1.50E-09 | 0.00008 | 37 |  |
| Appendicular lean mass | | rs7328187 | 74189974 | G | T | 0.498 | 0.012 | 0.0019 | 1.19E-09 | 0.00008 | 37 |  |
| Appendicular lean mass | | rs7320878 | 91994132 | A | G | 0.603 | -0.015 | 0.0019 | 1.34E-14 | 0.00014 | 62 |  |
| Appendicular lean mass | | rs144109601 | 50455500 | A | C | 0.042 | -0.028 | 0.0048 | 5.15E-09 | 0.00007 | 34 |  |
| Appendicular lean mass | | rs9590328 | 96448383 | G | A | 0.142 | 0.015 | 0.0027 | 2.02E-08 | 0.00007 | 32 |  |
| Appendicular lean mass | | rs78525785 | 111038331 | T | C | 0.620 | -0.017 | 0.0020 | 9.81E-18 | 0.00016 | 71 |  |
| Appendicular lean mass | | rs9594714 | 42800481 | T | G | 0.305 | 0.014 | 0.0021 | 2.65E-12 | 0.00010 | 47 |  |
| Appendicular lean mass | | rs9568031 | 48897520 | T | C | 0.706 | -0.012 | 0.0021 | 3.34E-08 | 0.00007 | 30 |  |
| Appendicular lean mass | | rs2812208 | 50707087 | C | G | 0.021 | 0.116 | 0.0066 | 5.51E-68 | 0.00068 | 307 |  |
| Appendicular lean mass | | rs8000973 | 100691367 | C | T | 0.533 | 0.013 | 0.0019 | 2.05E-12 | 0.00011 | 50 |  |
| Appendicular lean mass | | rs9525326 | 115075715 | G | A | 0.188 | -0.018 | 0.0024 | 3.62E-14 | 0.00013 | 59 |  |
| Appendicular lean mass | | rs532499 | 30165465 | C | T | 0.741 | -0.013 | 0.0022 | 4.90E-09 | 0.00007 | 33 |  |
| Appendicular lean mass | | rs9517483 | 99572712 | G | A | 0.699 | -0.018 | 0.0021 | 2.26E-18 | 0.00016 | 74 |  |
| Appendicular lean mass | | rs2296316 | 65520246 | C | T | 0.464 | -0.019 | 0.0019 | 1.59E-23 | 0.00023 | 102 |  |
| Appendicular lean mass | | rs113827862 | 89849527 | C | T | 0.061 | -0.024 | 0.0040 | 4.67E-09 | 0.00008 | 35 |  |
| Appendicular lean mass | | rs4900578 | 103926010 | T | A | 0.369 | -0.018 | 0.0020 | 1.76E-19 | 0.00017 | 78 |  |
| Appendicular lean mass | | rs56112295 | 105877057 | T | C | 0.226 | 0.015 | 0.0024 | 1.12E-10 | 0.00009 | 41 |  |
| Appendicular lean mass | | rs8019890 | 21538067 | A | C | 0.531 | 0.025 | 0.0019 | 1.96E-38 | 0.00038 | 173 |  |
| Appendicular lean mass | | rs8017006 | 42745052 | G | A | 0.328 | 0.012 | 0.0020 | 2.22E-09 | 0.00008 | 37 |  |
| Appendicular lean mass | | rs10483727 | 61072875 | C | T | 0.611 | -0.037 | 0.0019 | 6.73E-80 | 0.00083 | 375 |  |
| Appendicular lean mass | | rs8020095 | 67453858 | A | G | 0.150 | -0.015 | 0.0027 | 4.53E-08 | 0.00006 | 29 |  |
| Appendicular lean mass | | rs117068593 | 93118229 | T | C | 0.190 | 0.040 | 0.0024 | 8.83E-62 | 0.00063 | 282 | body fat percentage |
| Appendicular lean mass | | rs1190540 | 102897009 | G | A | 0.698 | 0.013 | 0.0021 | 1.62E-09 | 0.00008 | 35 |  |
| Appendicular lean mass | | rs17197114 | 21894526 | C | T | 0.177 | 0.018 | 0.0025 | 1.54E-12 | 0.00011 | 50 |  |
| Appendicular lean mass | | rs45528934 | 23793305 | T | C | 0.162 | 0.026 | 0.0026 | 1.97E-24 | 0.00023 | 102 |  |
| Appendicular lean mass | | rs28529055 | 92428216 | T | G | 0.437 | -0.015 | 0.0019 | 1.88E-14 | 0.00013 | 60 |  |
| Appendicular lean mass | | rs36226649 | 24835500 | C | T | 0.067 | 0.049 | 0.0038 | 3.05E-37 | 0.00036 | 163 |  |
| Appendicular lean mass | | rs28678024 | 25937161 | G | A | 0.285 | -0.012 | 0.0021 | 1.90E-08 | 0.00007 | 32 | BMI |
| Appendicular lean mass | | rs8904 | 35871217 | A | G | 0.363 | -0.016 | 0.0020 | 1.52E-15 | 0.00014 | 62 |  |
| Appendicular lean mass | | rs2070598 | 75360906 | A | G | 0.456 | 0.020 | 0.0019 | 6.36E-27 | 0.00026 | 115 | body fat percentage |
| Appendicular lean mass | | rs909220 | 75908780 | A | G | 0.444 | -0.015 | 0.0019 | 3.24E-15 | 0.00014 | 62 |  |
| Appendicular lean mass | | rs79066296 | 76391462 | T | C | 0.768 | -0.017 | 0.0022 | 6.11E-14 | 0.00013 | 59 |  |
| Appendicular lean mass | | rs12882130 | 103878774 | G | C | 0.384 | -0.020 | 0.0020 | 1.88E-24 | 0.00023 | 102 | BMI |
| Appendicular lean mass | | rs8018486 | 39818616 | G | A | 0.191 | -0.014 | 0.0024 | 1.18E-08 | 0.00007 | 33 |  |
| Appendicular lean mass | | rs7144307 | 69533837 | C | T | 0.385 | -0.012 | 0.0020 | 6.10E-10 | 0.00008 | 37 |  |
| Appendicular lean mass | | rs13316 | 93407301 | A | C | 0.430 | 0.012 | 0.0019 | 3.66E-09 | 0.00008 | 37 |  |
| Appendicular lean mass | | rs147233090 | 44028047 | T | C | 0.025 | -0.045 | 0.0061 | 3.95E-13 | 0.00012 | 53 |  |
| Appendicular lean mass | | rs4383083 | 63080442 | A | G | 0.647 | 0.011 | 0.0020 | 2.91E-08 | 0.00007 | 31 |  |
| Appendicular lean mass | | rs8042578 | 66992964 | G | C | 0.243 | 0.029 | 0.0022 | 2.29E-38 | 0.00038 | 170 |  |
| Appendicular lean mass | | rs990315 | 69578811 | C | T | 0.623 | -0.012 | 0.0020 | 5.07E-09 | 0.00007 | 33 |  |
| Appendicular lean mass | | rs74379684 | 94050205 | T | C | 0.075 | -0.027 | 0.0036 | 4.39E-14 | 0.00013 | 57 |  |
| Appendicular lean mass | | rs2871865 | 99194896 | G | C | 0.116 | -0.049 | 0.0030 | 3.40E-62 | 0.00060 | 270 |  |
| Appendicular lean mass | | rs17205463 | 62381413 | T | C | 0.448 | -0.026 | 0.0019 | 4.21E-43 | 0.00043 | 192 |  |
| Appendicular lean mass | | rs4965298 | 100802766 | T | C | 0.713 | -0.012 | 0.0021 | 1.81E-08 | 0.00007 | 32 |  |
| Appendicular lean mass | | rs11070842 | 51624185 | C | T | 0.163 | -0.015 | 0.0026 | 1.30E-08 | 0.00007 | 32 |  |
| Appendicular lean mass | | rs12907139 | 73521566 | A | G | 0.525 | -0.015 | 0.0019 | 4.89E-15 | 0.00014 | 61 |  |
| Appendicular lean mass | | rs5742915 | 74336633 | C | T | 0.461 | 0.025 | 0.0019 | 9.33E-39 | 0.00038 | 170 | BMI |
| Appendicular lean mass | | rs373736365 | 51360867 | G | T | 0.716 | 0.019 | 0.0022 | 4.53E-18 | 0.00016 | 74 |  |
| Appendicular lean mass | | rs11633371 | 89356832 | T | G | 0.476 | 0.022 | 0.0019 | 7.49E-30 | 0.00029 | 129 |  |
| Appendicular lean mass | | rs4932439 | 89401109 | G | A | 0.825 | -0.015 | 0.0025 | 1.43E-09 | 0.00008 | 36 |  |
| Appendicular lean mass | | rs2174008 | 38510456 | C | G | 0.498 | -0.019 | 0.0019 | 5.89E-24 | 0.00023 | 102 |  |
| Appendicular lean mass | | rs577289 | 40208911 | T | A | 0.281 | -0.013 | 0.0021 | 5.39E-09 | 0.00008 | 35 |  |
| Appendicular lean mass | | rs72726050 | 42270059 | C | T | 0.096 | -0.019 | 0.0034 | 1.84E-08 | 0.00007 | 32 |  |
| Appendicular lean mass | | rs12909863 | 75825822 | C | G | 0.251 | 0.019 | 0.0022 | 6.05E-18 | 0.00016 | 74 |  |
| Appendicular lean mass | | rs713467 | 84646473 | A | G | 0.435 | 0.015 | 0.0019 | 3.09E-14 | 0.00013 | 59 | body fat percentage |
| Appendicular lean mass | | rs11629593 | 96033696 | G | T | 0.616 | -0.011 | 0.0020 | 4.42E-08 | 0.00007 | 30 |  |
| Appendicular lean mass | | rs2663126 | 99563857 | A | G | 0.691 | -0.014 | 0.0021 | 1.36E-11 | 0.00010 | 44 |  |
| Appendicular lean mass | | rs116092985 | 2160973 | G | A | 0.096 | -0.040 | 0.0033 | 1.17E-34 | 0.00033 | 148 |  |
| Appendicular lean mass | | rs35811052 | 15128416 | G | A | 0.256 | -0.015 | 0.0022 | 8.84E-12 | 0.00010 | 45 |  |
| Appendicular lean mass | | rs72771070 | 19993750 | T | C | 0.283 | 0.015 | 0.0021 | 1.30E-12 | 0.00011 | 51 | BMI |
| Appendicular lean mass | | rs62033029 | 50107273 | A | G | 0.206 | -0.014 | 0.0023 | 1.73E-09 | 0.00008 | 38 |  |
| Appendicular lean mass | | rs72801843 | 53508802 | A | T | 0.302 | 0.031 | 0.0021 | 8.83E-52 | 0.00049 | 222 |  |
| Appendicular lean mass | | rs55872725 | 53809123 | T | C | 0.404 | 0.022 | 0.0019 | 1.46E-30 | 0.00030 | 137 | BMI |
| Appendicular lean mass | | rs4985445 | 69867835 | G | A | 0.457 | -0.018 | 0.0019 | 3.30E-20 | 0.00019 | 85 | BMI |
| Appendicular lean mass | | rs17818592 | 86088594 | C | T | 0.437 | -0.013 | 0.0019 | 1.23E-11 | 0.00010 | 46 |  |
| Appendicular lean mass | | rs8054549 | 86417234 | A | C | 0.449 | -0.025 | 0.0019 | 3.37E-39 | 0.00039 | 175 |  |
| Appendicular lean mass | | rs7185244 | 86546887 | C | T | 0.776 | -0.015 | 0.0023 | 1.40E-10 | 0.00009 | 41 |  |
| Appendicular lean mass | | rs12051245 | 783865 | C | T | 0.232 | 0.030 | 0.0022 | 2.56E-40 | 0.00041 | 185 |  |
| Appendicular lean mass | | rs143076454 | 921179 | A | G | 0.019 | -0.050 | 0.0070 | 1.06E-12 | 0.00011 | 51 |  |
| Appendicular lean mass | | rs246177 | 14380768 | T | C | 0.368 | 0.021 | 0.0020 | 2.04E-27 | 0.00025 | 114 |  |
| Appendicular lean mass | | rs116008080 | 67254841 | A | G | 0.024 | -0.042 | 0.0063 | 4.06E-11 | 0.00010 | 43 |  |
| Appendicular lean mass | | rs12926103 | 86371775 | A | G | 0.066 | 0.027 | 0.0038 | 9.60E-13 | 0.00011 | 51 |  |
| Appendicular lean mass | | rs77364196 | 88353016 | A | G | 0.053 | -0.033 | 0.0043 | 8.35E-15 | 0.00013 | 59 |  |
| Appendicular lean mass | | rs61528919 | 1004909 | C | T | 0.361 | 0.014 | 0.0020 | 3.33E-12 | 0.00011 | 49 |  |
| Appendicular lean mass | | rs35816944 | 1828030 | A | G | 0.007 | -0.109 | 0.0117 | 1.27E-20 | 0.00019 | 86 |  |
| Appendicular lean mass | | rs78457529 | 24950880 | T | C | 0.012 | -0.090 | 0.0088 | 1.22E-24 | 0.00023 | 106 |  |
| Appendicular lean mass | | rs4788218 | 30055750 | C | T | 0.401 | 0.028 | 0.0019 | 5.52E-46 | 0.00047 | 209 | BMI |
| Appendicular lean mass | | rs2240735 | 4027605 | T | C | 0.748 | 0.019 | 0.0022 | 3.99E-18 | 0.00016 | 74 | BMI |
| Appendicular lean mass | | rs77809369 | 9052448 | T | C | 0.064 | 0.024 | 0.0039 | 9.83E-10 | 0.00008 | 37 |  |
| Appendicular lean mass | | rs35268848 | 67927240 | A | C | 0.012 | 0.074 | 0.0101 | 2.83E-13 | 0.00012 | 53 |  |
| Appendicular lean mass | | rs62070319 | 89573216 | T | C | 0.443 | -0.018 | 0.0020 | 2.54E-20 | 0.00018 | 81 |  |
| Appendicular lean mass | | rs6502935 | 1650168 | T | C | 0.738 | -0.013 | 0.0022 | 6.31E-09 | 0.00007 | 32 |  |
| Appendicular lean mass | | rs113146332 | 42565977 | C | G | 0.038 | 0.031 | 0.0049 | 2.99E-10 | 0.00009 | 40 |  |
| Appendicular lean mass | | rs9894577 | 43223292 | A | G | 0.318 | -0.031 | 0.0020 | 1.40E-52 | 0.00053 | 240 |  |
| Appendicular lean mass | | rs2005172 | 61996255 | C | A | 0.640 | 0.048 | 0.0020 | 2.35E-128 | 0.00128 | 576 | body fat percentage |
| Appendicular lean mass | | rs28485212 | 63550026 | T | C | 0.149 | -0.019 | 0.0027 | 1.24E-12 | 0.00011 | 48 |  |
| Appendicular lean mass | | rs9890062 | 17434352 | A | G | 0.062 | 0.027 | 0.0039 | 1.20E-11 | 0.00010 | 47 |  |
| Appendicular lean mass | | rs117972846 | 26947476 | G | T | 0.029 | 0.034 | 0.0057 | 5.47E-09 | 0.00008 | 35 |  |
| Appendicular lean mass | | rs2289629 | 27959903 | A | G | 0.345 | -0.015 | 0.0020 | 8.02E-14 | 0.00012 | 55 | body fat percentage |
| Appendicular lean mass | | rs2019203 | 36908672 | A | C | 0.491 | 0.019 | 0.0019 | 1.84E-23 | 0.00022 | 99 |  |
| Appendicular lean mass | | rs9905385 | 59498250 | G | A | 0.671 | -0.034 | 0.0020 | 1.94E-63 | 0.00064 | 287 |  |
| Appendicular lean mass | | rs78766798 | 7517075 | C | T | 0.085 | 0.032 | 0.0035 | 2.61E-20 | 0.00018 | 83 |  |
| Appendicular lean mass | | rs78378222 | 7571752 | G | T | 0.012 | 0.138 | 0.0087 | 4.51E-56 | 0.00056 | 252 |  |
| Appendicular lean mass | | rs2112617 | 46977125 | A | G | 0.529 | -0.017 | 0.0019 | 1.07E-18 | 0.00017 | 77 |  |
| Appendicular lean mass | | rs2676298 | 62726707 | T | C | 0.853 | -0.027 | 0.0027 | 2.39E-23 | 0.00022 | 99 |  |
| Appendicular lean mass | | rs7220127 | 64545922 | C | T | 0.426 | -0.011 | 0.0019 | 4.60E-08 | 0.00007 | 31 |  |
| Appendicular lean mass | | rs36000545 | 79093822 | G | A | 0.396 | -0.022 | 0.0020 | 2.56E-29 | 0.00027 | 121 |  |
| Appendicular lean mass | | rs6505216 | 29206421 | T | G | 0.233 | -0.050 | 0.0023 | 1.83E-101 | 0.00104 | 469 |  |
| Appendicular lean mass | | rs57791149 | 54222307 | C | T | 0.402 | -0.017 | 0.0019 | 3.26E-19 | 0.00018 | 83 |  |
| Appendicular lean mass | | rs2521349 | 67503501 | A | G | 0.385 | 0.016 | 0.0019 | 2.01E-15 | 0.00015 | 67 |  |
| Appendicular lean mass | | rs173135 | 68172326 | T | C | 0.115 | -0.034 | 0.0030 | 3.25E-30 | 0.00029 | 129 |  |
| Appendicular lean mass | | rs12943867 | 79409710 | A | G | 0.337 | 0.018 | 0.0020 | 7.57E-20 | 0.00019 | 85 |  |
| Appendicular lean mass | | rs9898189 | 80480516 | G | C | 0.645 | -0.016 | 0.0021 | 1.88E-15 | 0.00013 | 60 |  |
| Appendicular lean mass | | rs57513571 | 2309130 | T | C | 0.200 | -0.019 | 0.0024 | 7.02E-16 | 0.00014 | 63 |  |
| Appendicular lean mass | | rs11867855 | 18262584 | T | G | 0.237 | -0.013 | 0.0022 | 2.93E-09 | 0.00008 | 36 |  |
| Appendicular lean mass | | rs4640244 | 21284223 | G | A | 0.399 | -0.020 | 0.0019 | 3.81E-25 | 0.00025 | 111 | BMI |
| Appendicular lean mass | | rs72829852 | 46633974 | T | C | 0.062 | 0.031 | 0.0039 | 3.74E-15 | 0.00014 | 63 |  |
| Appendicular lean mass | | rs2592208 | 67408873 | A | C | 0.513 | -0.012 | 0.0019 | 5.52E-11 | 0.00009 | 43 |  |
| Appendicular lean mass | | rs113232639 | 20715656 | A | G | 0.493 | 0.033 | 0.0019 | 4.79E-64 | 0.00066 | 296 |  |
| Appendicular lean mass | | rs8084413 | 22869123 | A | G | 0.469 | -0.013 | 0.0019 | 3.25E-11 | 0.00010 | 45 |  |
| Appendicular lean mass | | rs2978362 | 32959397 | T | C | 0.533 | 0.011 | 0.0019 | 2.85E-08 | 0.00007 | 31 |  |
| Appendicular lean mass | | rs74494415 | 74972138 | T | C | 0.040 | -0.042 | 0.0049 | 1.82E-17 | 0.00016 | 72 |  |
| Appendicular lean mass | | rs2347808 | 2750856 | A | G | 0.514 | -0.013 | 0.0019 | 5.79E-11 | 0.00010 | 43 |  |
| Appendicular lean mass | | rs33973388 | 46611842 | T | G | 0.435 | 0.025 | 0.0019 | 1.45E-38 | 0.00038 | 172 |  |
| Appendicular lean mass | | rs4940874 | 57105638 | G | A | 0.812 | 0.015 | 0.0024 | 1.24E-09 | 0.00008 | 38 |  |
| Appendicular lean mass | | rs1786263 | 13116432 | T | G | 0.606 | -0.019 | 0.0019 | 1.03E-22 | 0.00022 | 100 |  |
| Appendicular lean mass | | rs62103240 | 77650637 | A | G | 0.071 | 0.021 | 0.0037 | 1.40E-08 | 0.00007 | 33 |  |
| Appendicular lean mass | | rs4121583 | 125075 | T | C | 0.619 | 0.012 | 0.0020 | 4.62E-09 | 0.00008 | 35 |  |
| Appendicular lean mass | | rs568267 | 8799828 | T | C | 0.743 | 0.012 | 0.0022 | 2.23E-08 | 0.00007 | 31 |  |
| Appendicular lean mass | | rs35073631 | 22696964 | C | T | 0.433 | 0.011 | 0.0019 | 5.92E-09 | 0.00008 | 35 |  |
| Appendicular lean mass | | rs12962050 | 35179808 | A | G | 0.645 | 0.015 | 0.0020 | 1.52E-14 | 0.00013 | 59 |  |
| Appendicular lean mass | | rs7229520 | 46516468 | A | G | 0.662 | -0.022 | 0.0020 | 9.21E-29 | 0.00028 | 125 |  |
| Appendicular lean mass | | rs7228151 | 57181694 | C | T | 0.206 | -0.019 | 0.0023 | 3.19E-15 | 0.00014 | 65 |  |
| Appendicular lean mass | | rs9957318 | 33039106 | G | A | 0.348 | 0.019 | 0.0020 | 1.02E-20 | 0.00019 | 87 |  |
| Appendicular lean mass | | rs60389750 | 77182836 | T | C | 0.314 | -0.018 | 0.0021 | 1.06E-16 | 0.00015 | 69 |  |
| Appendicular lean mass | | rs79441499 | 7201704 | T | C | 0.406 | -0.014 | 0.0019 | 9.67E-13 | 0.00012 | 53 |  |
| Appendicular lean mass | | rs45474992 | 47724564 | T | C | 0.036 | -0.062 | 0.0051 | 2.17E-33 | 0.00032 | 146 |  |
| Appendicular lean mass | | rs10421750 | 50290604 | T | G | 0.312 | -0.015 | 0.0021 | 5.73E-12 | 0.00011 | 48 |  |
| Appendicular lean mass | | rs16989695 | 4505445 | A | G | 0.517 | -0.014 | 0.0019 | 1.93E-13 | 0.00012 | 54 |  |
| Appendicular lean mass | | rs12150907 | 4940630 | A | G | 0.198 | -0.022 | 0.0024 | 7.22E-20 | 0.00018 | 83 |  |
| Appendicular lean mass | | rs10948 | 10754905 | T | G | 0.664 | -0.025 | 0.0020 | 3.43E-36 | 0.00035 | 159 |  |
| Appendicular lean mass | | rs2607234 | 35563834 | G | A | 0.948 | -0.030 | 0.0043 | 2.00E-12 | 0.00011 | 49 |  |
| Appendicular lean mass | | rs117203652 | 49857801 | A | G | 0.030 | -0.035 | 0.0055 | 4.32E-10 | 0.00009 | 40 |  |
| Appendicular lean mass | | rs4807472 | 3448842 | C | T | 0.676 | -0.016 | 0.0020 | 8.18E-15 | 0.00014 | 62 |  |
| Appendicular lean mass | | rs12461874 | 17180358 | A | C | 0.278 | -0.018 | 0.0021 | 1.26E-17 | 0.00016 | 74 |  |
| Appendicular lean mass | | rs116919274 | 17359808 | A | G | 0.046 | 0.027 | 0.0046 | 3.52E-09 | 0.00008 | 35 |  |
| Appendicular lean mass | | rs111901094 | 19513570 | T | G | 0.182 | -0.025 | 0.0025 | 4.04E-24 | 0.00023 | 102 |  |
| Appendicular lean mass | | rs75702986 | 35566151 | A | G | 0.186 | -0.016 | 0.0025 | 3.14E-11 | 0.00009 | 43 |  |
| Appendicular lean mass | | rs4252548 | 55879672 | T | C | 0.022 | -0.075 | 0.0065 | 2.96E-31 | 0.00030 | 134 |  |
| Appendicular lean mass | | rs8112948 | 2175005 | A | T | 0.286 | -0.030 | 0.0022 | 4.24E-42 | 0.00040 | 182 |  |
| Appendicular lean mass | | rs350832 | 4069426 | A | G | 0.771 | -0.017 | 0.0023 | 3.44E-13 | 0.00011 | 51 | BMI |
| Appendicular lean mass | | rs11260035 | 7898957 | A | G | 0.276 | 0.015 | 0.0021 | 1.86E-12 | 0.00011 | 51 |  |
| Appendicular lean mass | | rs11672848 | 37570704 | T | C | 0.525 | -0.017 | 0.0019 | 7.73E-19 | 0.00018 | 81 |  |
| Appendicular lean mass | | rs2287821 | 33935102 | T | C | 0.509 | -0.015 | 0.0019 | 6.95E-16 | 0.00014 | 65 | BMI |
| Appendicular lean mass | | rs147110934 | 55993436 | T | G | 0.024 | -0.072 | 0.0062 | 9.39E-32 | 0.00030 | 136 |  |
| Appendicular lean mass | | rs6054390 | 6592094 | A | G | 0.628 | -0.019 | 0.0020 | 1.45E-21 | 0.00020 | 88 | BMI |
| Appendicular lean mass | | rs73125634 | 20069826 | T | G | 0.278 | -0.020 | 0.0021 | 5.11E-20 | 0.00019 | 86 |  |
| Appendicular lean mass | | rs12185775 | 20293769 | C | G | 0.117 | -0.017 | 0.0030 | 3.66E-08 | 0.00007 | 31 |  |
| Appendicular lean mass | | rs112021215 | 62867186 | C | T | 0.201 | -0.015 | 0.0025 | 5.99E-09 | 0.00008 | 35 |  |
| Appendicular lean mass | | rs4815952 | 6934897 | C | T | 0.522 | -0.016 | 0.0019 | 1.24E-16 | 0.00016 | 72 |  |
| Appendicular lean mass | | rs35963161 | 49210635 | A | G | 0.476 | -0.016 | 0.0019 | 7.46E-16 | 0.00015 | 68 |  |
| Appendicular lean mass | | rs77447813 | 50827041 | C | G | 0.087 | 0.022 | 0.0034 | 3.21E-11 | 0.00010 | 43 | BMI |
| Appendicular lean mass | | rs2236096 | 3266319 | C | T | 0.233 | 0.018 | 0.0023 | 1.29E-15 | 0.00014 | 61 |  |
| Appendicular lean mass | | rs6142059 | 32544327 | C | T | 0.493 | 0.012 | 0.0019 | 1.19E-09 | 0.00008 | 37 | BMI |
| Appendicular lean mass | | rs143384 | 34025756 | G | A | 0.404 | 0.073 | 0.0019 | 1.00E-200 | 0.00322 | 1456 | waist hip ratio |
| Appendicular lean mass | | rs80132799 | 62322896 | T | C | 0.068 | 0.023 | 0.0038 | 1.31E-09 | 0.00008 | 37 |  |
| Appendicular lean mass | | rs6054491 | 6709535 | G | C | 0.240 | -0.014 | 0.0022 | 2.15E-10 | 0.00009 | 42 |  |
| Appendicular lean mass | | rs684905 | 10472790 | T | C | 0.418 | -0.012 | 0.0019 | 7.10E-10 | 0.00009 | 39 |  |
| Appendicular lean mass | | rs6082354 | 21217976 | C | A | 0.668 | -0.024 | 0.0020 | 1.19E-32 | 0.00032 | 144 |  |
| Appendicular lean mass | | rs4287835 | 31457337 | C | T | 0.534 | 0.015 | 0.0019 | 9.94E-15 | 0.00013 | 60 |  |
| Appendicular lean mass | | rs1291114 | 35500850 | C | G | 0.890 | 0.017 | 0.0031 | 1.95E-08 | 0.00007 | 31 |  |
| Appendicular lean mass | | rs57696574 | 54884826 | C | A | 0.398 | 0.017 | 0.0020 | 4.50E-18 | 0.00017 | 75 |  |
| Appendicular lean mass | | rs34879158 | 32300634 | C | A | 0.263 | -0.036 | 0.0022 | 1.55E-63 | 0.00060 | 272 |  |
| Appendicular lean mass | | rs6028716 | 38547459 | A | G | 0.259 | -0.021 | 0.0022 | 4.58E-22 | 0.00020 | 91 |  |
| Appendicular lean mass | | rs6066122 | 45558573 | G | C | 0.763 | 0.013 | 0.0023 | 1.76E-08 | 0.00007 | 30 |  |
| Appendicular lean mass | | rs13037813 | 47750588 | C | T | 0.239 | 0.029 | 0.0022 | 1.71E-39 | 0.00039 | 176 |  |
| Appendicular lean mass | | rs73197345 | 36770120 | A | T | 0.137 | 0.021 | 0.0028 | 3.55E-14 | 0.00013 | 57 |  |
| Appendicular lean mass | | rs12483401 | 35443829 | C | T | 0.022 | -0.039 | 0.0067 | 9.22E-09 | 0.00007 | 33 |  |
| Appendicular lean mass | | rs2230033 | 39671476 | A | G | 0.564 | -0.027 | 0.0019 | 3.49E-43 | 0.00043 | 195 |  |
| Appendicular lean mass | | rs112153300 | 47547474 | A | G | 0.089 | 0.026 | 0.0034 | 7.05E-15 | 0.00013 | 59 |  |
| Appendicular lean mass | | rs2212926 | 38066883 | A | C | 0.211 | -0.022 | 0.0023 | 7.75E-21 | 0.00020 | 91 |  |
| Appendicular lean mass | | rs4818280 | 18114472 | T | C | 0.627 | -0.012 | 0.0020 | 2.84E-10 | 0.00009 | 38 |  |
| Appendicular lean mass | | rs9610447 | 20768891 | T | C | 0.747 | 0.015 | 0.0022 | 5.29E-12 | 0.00011 | 48 |  |
| Appendicular lean mass | | rs5753518 | 31631314 | A | G | 0.090 | 0.024 | 0.0033 | 5.04E-13 | 0.00012 | 54 |  |
| Appendicular lean mass | | rs7286917 | 39860868 | G | A | 0.746 | 0.017 | 0.0023 | 5.18E-14 | 0.00012 | 55 |  |
| Appendicular lean mass | | rs41311445 | 42070374 | C | A | 0.096 | -0.033 | 0.0032 | 4.73E-24 | 0.00023 | 105 |  |
| Appendicular lean mass | | rs5763821 | 30549071 | C | A | 0.391 | 0.019 | 0.0020 | 1.06E-21 | 0.00020 | 91 |  |
| Appendicular lean mass | | rs6000886 | 38176670 | C | T | 0.648 | 0.013 | 0.0020 | 5.88E-11 | 0.00010 | 43 |  |
| Appendicular lean mass | | rs8136517 | 46439433 | C | T | 0.065 | 0.027 | 0.0039 | 6.58E-12 | 0.00010 | 47 |  |
| Appendicular lean mass | | rs165849 | 19958669 | A | G | 0.698 | 0.016 | 0.0021 | 4.57E-14 | 0.00012 | 56 |  |
| Appendicular lean mass | | rs10453441 | 46363739 | G | A | 0.402 | -0.014 | 0.0020 | 9.10E-13 | 0.00011 | 48 |  |
| Appendicular lean mass | | rs28379706 | 50728062 | C | T | 0.393 | 0.011 | 0.0020 | 4.49E-09 | 0.00007 | 32 |  |
|  |

Abbreviation: SNP, single nucleotide polymorphism; EA, Effect allele; NEA, Non-effect allele; EAF, effect allele frequency; SE, standard error; BMI, body mass index.

*^a^ R^2^* was calculated using the following formula: (2×EAF×(1-EAF)×beta^2^)/[(2×EAF×(1-EAF)×beta^2^)+(2×EAF×(1-EAF)×N×SE^2^)], where EAF is the effect allele

frequency, beta is the estimated effect on urate. Ν is the sample size of the GWAS for the SNP-urate association and SE is the standard error of the estimated effect.

*^b^ F* statistic was calculated using the following formula: *R^2^*(N-2)/(1-*R^2^*), where *R^2^* is the proportion of variance in urate explained by each instrument and N is the sample size of the GWAS for the SNP-urate association.

^c^ SNPs associated with confounding factors were removed after searching Phenoscanner database.

Supplementary Table 6 Characteristics of SNPs used as genetic instruments for whole-body lean mass

| Exposure | | SNP | Position | EA | NEA | EAF | SNP-Exposure association | | | R^2 a^ | F-statistic ^b^ | Confounders ^c^ |
| --- | --- | --- | --- | --- | --- | --- | --- | --- | --- | --- | --- | --- |
|  | |  |  |  |  |  | Beta | SE | P value |  |  |  |
| whole-body lean mass | | rs4648626 | 2200390 | A | C | 0.457 | 0.008 | 0.001 | 9.10E-12 | 0.00010 | 46.51 |  |
| whole-body lean mass | | rs6665399 | 21553933 | C | T | 0.445 | 0.008 | 0.001 | 4.90E-11 | 0.00010 | 43.21 |  |
| whole-body lean mass | | rs67408364 | 39618556 | G | A | 0.214 | 0.014 | 0.002 | 6.00E-21 | 0.00019 | 88.16 | BMI |
| whole-body lean mass | | rs2885697 | 41544279 | T | G | 0.665 | -0.019 | 0.001 | 5.10E-47 | 0.00046 | 207.37 |  |
| whole-body lean mass | | rs892511 | 110078255 | G | C | 0.028 | 0.026 | 0.004 | 3.70E-12 | 0.00011 | 48.29 | BMI |
| whole-body lean mass | | rs7513326 | 159895536 | A | G | 0.508 | -0.007 | 0.001 | 2.50E-08 | 0.00007 | 31.05 |  |
| whole-body lean mass | | rs12729817 | 218971896 | G | A | 0.474 | -0.008 | 0.001 | 2.20E-11 | 0.00010 | 44.76 |  |
| whole-body lean mass | | rs2281175 | 1665702 | C | T | 0.405 | 0.011 | 0.001 | 6.30E-18 | 0.00016 | 74.42 | BMI |
| whole-body lean mass | | rs161799 | 8015560 | G | A | 0.344 | -0.008 | 0.001 | 9.00E-10 | 0.00008 | 37.52 |  |
| whole-body lean mass | | rs12041740 | 11236524 | A | G | 0.740 | -0.015 | 0.001 | 2.10E-25 | 0.00024 | 108.45 |  |
| whole-body lean mass | | rs926436 | 22563429 | A | G | 0.857 | 0.010 | 0.002 | 3.50E-08 | 0.00007 | 30.39 |  |
| whole-body lean mass | | rs55800172 | 32414767 | A | G | 0.064 | 0.018 | 0.003 | 5.70E-13 | 0.00011 | 51.96 |  |
| whole-body lean mass | | rs2568958 | 72765116 | A | G | 0.604 | 0.009 | 0.001 | 5.50E-14 | 0.00012 | 56.55 | BMI |
| whole-body lean mass | | rs3845344 | 75001480 | T | C | 0.391 | 0.008 | 0.001 | 3.10E-11 | 0.00010 | 44.11 | BMI |
| whole-body lean mass | | rs17363646 | 86823503 | G | A | 0.136 | 0.012 | 0.002 | 5.50E-12 | 0.00010 | 47.50 |  |
| whole-body lean mass | | rs12047986 | 155043812 | G | A | 0.522 | -0.008 | 0.001 | 5.10E-10 | 0.00008 | 38.64 |  |
| whole-body lean mass | | rs116817990 | 178989260 | G | A | 0.034 | -0.020 | 0.003 | 5.70E-09 | 0.00007 | 33.94 |  |
| whole-body lean mass | | rs12070699 | 190230408 | T | C | 0.563 | -0.009 | 0.001 | 1.50E-13 | 0.00012 | 54.53 | BMI |
| whole-body lean mass | | rs6693481 | 203766395 | C | T | 0.695 | -0.008 | 0.001 | 8.60E-10 | 0.00008 | 37.62 |  |
| whole-body lean mass | | rs12072845 | 214630757 | A | G | 0.395 | -0.014 | 0.001 | 2.80E-28 | 0.00027 | 121.65 |  |
| whole-body lean mass | | rs2789366 | 235508270 | A | G | 0.346 | -0.009 | 0.001 | 1.60E-12 | 0.00011 | 49.98 |  |
| whole-body lean mass | | rs284315 | 10734800 | G | A | 0.498 | -0.008 | 0.001 | 4.90E-10 | 0.00009 | 38.71 | BMI |
| whole-body lean mass | | rs2092322 | 22435723 | C | G | 0.537 | -0.010 | 0.001 | 1.50E-14 | 0.00013 | 59.13 |  |
| whole-body lean mass | | rs11578046 | 23425139 | A | G | 0.327 | -0.013 | 0.001 | 1.10E-22 | 0.00021 | 96.11 |  |
| whole-body lean mass | | rs4360494 | 38455891 | C | G | 0.554 | -0.010 | 0.001 | 2.10E-15 | 0.00014 | 62.94 |  |
| whole-body lean mass | | rs2104449 | 97082532 | T | G | 0.283 | 0.011 | 0.001 | 9.40E-15 | 0.00013 | 60.01 | BMI |
| whole-body lean mass | | rs527248 | 177875514 | G | A | 0.209 | 0.026 | 0.002 | 2.50E-63 | 0.00062 | 282.17 | BMI |
| whole-body lean mass | | rs12731187 | 202013757 | T | C | 0.358 | -0.008 | 0.001 | 2.50E-09 | 0.00008 | 35.52 | BMI |
| whole-body lean mass | | rs2970592 | 212220847 | A | G | 0.588 | 0.009 | 0.001 | 3.00E-13 | 0.00012 | 53.23 |  |
| whole-body lean mass | | rs35492502 | 217806224 | A | G | 0.296 | 0.010 | 0.001 | 2.10E-13 | 0.00012 | 53.93 |  |
| whole-body lean mass | | rs75786059 | 26449327 | G | A | 0.175 | 0.014 | 0.002 | 1.70E-18 | 0.00017 | 77.00 |  |
| whole-body lean mass | | rs11263853 | 36579648 | A | G | 0.810 | 0.009 | 0.002 | 4.80E-09 | 0.00008 | 34.25 |  |
| whole-body lean mass | | rs4926542 | 50263773 | T | C | 0.682 | -0.010 | 0.001 | 2.40E-13 | 0.00012 | 53.67 | BMI |
| whole-body lean mass | | rs12095997 | 51391845 | T | C | 0.089 | 0.023 | 0.002 | 2.70E-25 | 0.00024 | 107.98 |  |
| whole-body lean mass | | rs12140153 | 62579891 | T | G | 0.094 | -0.017 | 0.002 | 3.80E-15 | 0.00014 | 61.80 | BMI |
| whole-body lean mass | | rs17277008 | 172105162 | C | T | 0.313 | 0.014 | 0.001 | 4.60E-27 | 0.00026 | 116.08 |  |
| whole-body lean mass | | rs2678204 | 201800511 | G | T | 0.340 | 0.012 | 0.001 | 2.40E-20 | 0.00019 | 85.43 | BMI |
| whole-body lean mass | | rs11240565 | 205722958 | T | C | 0.398 | 0.013 | 0.001 | 8.80E-25 | 0.00023 | 105.64 |  |
| whole-body lean mass | | rs148662000 | 228721530 | T | G | 0.064 | -0.015 | 0.003 | 3.00E-09 | 0.00008 | 35.17 |  |
| whole-body lean mass | | rs6681795 | 56583274 | G | A | 0.228 | 0.015 | 0.001 | 1.60E-24 | 0.00023 | 104.48 |  |
| whole-body lean mass | | rs34517439 | 78450517 | A | C | 0.122 | 0.037 | 0.002 | 3.30E-84 | 0.00083 | 378.08 | BMI |
| whole-body lean mass | | rs77848106 | 107971673 | A | C | 0.296 | -0.009 | 0.001 | 3.90E-11 | 0.00010 | 43.67 | BMI |
| whole-body lean mass | | rs60804050 | 118870373 | A | G | 0.256 | -0.010 | 0.001 | 1.50E-12 | 0.00011 | 50.11 |  |
| whole-body lean mass | | rs11205354 | 150249101 | A | C | 0.444 | -0.008 | 0.001 | 3.70E-10 | 0.00009 | 39.25 |  |
| whole-body lean mass | | rs76798800 | 154994978 | T | G | 0.266 | 0.023 | 0.001 | 2.30E-59 | 0.00058 | 263.99 | BMI |
| whole-body lean mass | | rs77159542 | 170190337 | G | A | 0.066 | 0.014 | 0.002 | 8.60E-09 | 0.00007 | 33.14 |  |
| whole-body lean mass | | rs1040457 | 176802119 | T | C | 0.505 | 0.015 | 0.001 | 1.40E-30 | 0.00029 | 132.12 |  |
| whole-body lean mass | | rs2615075 | 225934295 | G | A | 0.376 | 0.010 | 0.001 | 2.00E-14 | 0.00013 | 58.53 |  |
| whole-body lean mass | | rs6743060 | 629510 | A | C | 0.828 | 0.034 | 0.002 | 1.10E-93 | 0.00093 | 421.54 | BMI |
| whole-body lean mass | | rs6721191 | 10190115 | G | A | 0.578 | -0.008 | 0.001 | 2.00E-09 | 0.00008 | 36.00 |  |
| whole-body lean mass | | rs1374370 | 85818273 | A | G | 0.305 | 0.010 | 0.001 | 3.50E-14 | 0.00013 | 57.44 |  |
| whole-body lean mass | | rs2140046 | 169706079 | C | T | 0.364 | -0.012 | 0.001 | 8.20E-20 | 0.00018 | 83.01 |  |
| whole-body lean mass | | rs116337081 | 183954625 | T | C | 0.070 | 0.014 | 0.002 | 3.60E-09 | 0.00008 | 34.83 |  |
| whole-body lean mass | | rs17443541 | 200402624 | C | T | 0.189 | -0.011 | 0.002 | 5.80E-12 | 0.00010 | 47.39 |  |
| whole-body lean mass | | rs11684531 | 219835489 | G | A | 0.133 | -0.011 | 0.002 | 6.60E-10 | 0.00008 | 38.14 |  |
| whole-body lean mass | | rs3116201 | 233074205 | A | G | 0.098 | -0.018 | 0.002 | 7.10E-18 | 0.00016 | 74.20 |  |
| whole-body lean mass | | rs3772051 | 241672904 | A | G | 0.237 | -0.009 | 0.001 | 5.90E-09 | 0.00007 | 33.85 |  |
| whole-body lean mass | | rs4676442 | 241740907 | C | T | 0.356 | 0.007 | 0.001 | 9.10E-09 | 0.00007 | 33.02 |  |
| whole-body lean mass | | rs1260326 | 27730940 | C | T | 0.604 | 0.019 | 0.001 | 1.10E-50 | 0.00049 | 224.19 |  |
| whole-body lean mass | | rs10172196 | 36780549 | A | G | 0.306 | 0.011 | 0.001 | 1.30E-15 | 0.00014 | 63.85 | body fat percentage |
| whole-body lean mass | | rs1805165 | 88874891 | A | C | 0.721 | -0.010 | 0.001 | 2.50E-13 | 0.00012 | 53.60 |  |
| whole-body lean mass | | rs71423263 | 144024781 | G | T | 0.141 | 0.012 | 0.002 | 1.50E-11 | 0.00010 | 45.53 | BMI |
| whole-body lean mass | | rs13392079 | 169119178 | C | T | 0.756 | -0.008 | 0.001 | 3.80E-09 | 0.00008 | 34.72 |  |
| whole-body lean mass | | rs72885917 | 172416376 | C | A | 0.247 | -0.020 | 0.001 | 2.20E-46 | 0.00045 | 204.45 |  |
| whole-body lean mass | | rs17400325 | 178565913 | C | T | 0.042 | 0.022 | 0.003 | 3.80E-12 | 0.00011 | 48.25 |  |
| whole-body lean mass | | rs13430869 | 218146818 | T | G | 0.742 | 0.014 | 0.001 | 1.40E-21 | 0.00020 | 91.08 |  |
| whole-body lean mass | | rs1478575 | 218278555 | A | T | 0.684 | 0.018 | 0.001 | 1.70E-39 | 0.00038 | 172.90 |  |
| whole-body lean mass | | rs1542224 | 223963874 | C | T | 0.719 | 0.013 | 0.001 | 2.00E-20 | 0.00019 | 85.79 |  |
| whole-body lean mass | | rs10202845 | 42575820 | G | A | 0.113 | -0.018 | 0.002 | 4.30E-19 | 0.00018 | 79.72 |  |
| whole-body lean mass | | rs2920974 | 55537126 | C | A | 0.560 | 0.007 | 0.001 | 1.10E-08 | 0.00007 | 32.68 |  |
| whole-body lean mass | | rs116211567 | 68425404 | C | T | 0.081 | -0.018 | 0.002 | 5.20E-15 | 0.00013 | 61.16 |  |
| whole-body lean mass | | rs11545482 | 70315987 | T | C | 0.020 | -0.032 | 0.004 | 4.40E-13 | 0.00012 | 52.45 |  |
| whole-body lean mass | | rs3771382 | 71559445 | G | C | 0.571 | -0.012 | 0.001 | 1.90E-21 | 0.00020 | 90.46 |  |
| whole-body lean mass | | rs6719296 | 103101108 | A | G | 0.495 | 0.009 | 0.001 | 1.10E-12 | 0.00011 | 50.57 |  |
| whole-body lean mass | | rs6747657 | 112003163 | A | G | 0.282 | 0.008 | 0.001 | 4.70E-09 | 0.00008 | 34.33 |  |
| whole-body lean mass | | rs10803955 | 183228114 | G | A | 0.508 | -0.011 | 0.001 | 6.40E-19 | 0.00017 | 78.95 |  |
| whole-body lean mass | | rs12694042 | 207029825 | T | C | 0.502 | -0.008 | 0.001 | 3.80E-11 | 0.00010 | 43.71 |  |
| whole-body lean mass | | rs17246129 | 227259964 | A | G | 0.305 | 0.013 | 0.001 | 1.40E-20 | 0.00019 | 86.50 |  |
| whole-body lean mass | | rs2197563 | 233687080 | A | G | 0.595 | 0.010 | 0.001 | 3.80E-16 | 0.00015 | 66.31 |  |
| whole-body lean mass | | rs10170971 | 242492779 | C | G | 0.502 | -0.009 | 0.001 | 2.60E-14 | 0.00013 | 58.01 |  |
| whole-body lean mass | | rs77165542 | 430975 | T | C | 0.035 | -0.058 | 0.003 | 6.60E-67 | 0.00066 | 298.60 | BMI |
| whole-body lean mass | | rs112544217 | 20222686 | T | C | 0.022 | -0.027 | 0.004 | 4.80E-10 | 0.00009 | 38.77 |  |
| whole-body lean mass | | rs12713004 | 23896049 | G | A | 0.725 | 0.017 | 0.001 | 3.90E-35 | 0.00034 | 152.98 |  |
| whole-body lean mass | | rs41458449 | 46929951 | C | G | 0.144 | -0.011 | 0.002 | 1.90E-09 | 0.00008 | 36.08 |  |
| whole-body lean mass | | rs6743107 | 100844781 | T | C | 0.275 | 0.008 | 0.001 | 1.30E-08 | 0.00007 | 32.29 | BMI |
| whole-body lean mass | | rs35651070 | 121613739 | A | G | 0.167 | -0.010 | 0.002 | 9.90E-09 | 0.00007 | 32.85 |  |
| whole-body lean mass | | rs10188231 | 142297493 | G | C | 0.186 | -0.010 | 0.002 | 2.70E-10 | 0.00009 | 39.88 | BMI |
| whole-body lean mass | | rs1064213 | 198950240 | A | G | 0.478 | 0.011 | 0.001 | 1.80E-20 | 0.00019 | 85.96 | BMI |
| whole-body lean mass | | rs1047891 | 211540507 | A | C | 0.316 | 0.017 | 0.001 | 6.00E-37 | 0.00035 | 161.26 | BMI |
| whole-body lean mass | | rs11689727 | 25458100 | A | C | 0.331 | -0.011 | 0.001 | 7.00E-17 | 0.00015 | 69.66 |  |
| whole-body lean mass | | rs115179432 | 33348679 | G | A | 0.072 | -0.022 | 0.002 | 4.50E-20 | 0.00019 | 84.20 |  |
| whole-body lean mass | | rs59985551 | 56106928 | T | C | 0.226 | -0.018 | 0.001 | 8.60E-36 | 0.00034 | 155.96 |  |
| whole-body lean mass | | rs752070 | 74824970 | G | A | 0.126 | 0.011 | 0.002 | 8.20E-10 | 0.00008 | 37.71 |  |
| whole-body lean mass | | rs4504126 | 33600582 | C | A | 0.027 | 0.025 | 0.004 | 4.30E-11 | 0.00010 | 43.45 |  |
| whole-body lean mass | | rs6779752 | 85663849 | A | G | 0.638 | -0.013 | 0.001 | 8.50E-23 | 0.00021 | 96.59 | BMI |
| whole-body lean mass | | rs843374 | 183997261 | T | A | 0.587 | -0.011 | 0.001 | 5.40E-19 | 0.00017 | 79.27 |  |
| whole-body lean mass | | rs62246311 | 9498143 | A | G | 0.102 | 0.013 | 0.002 | 2.20E-10 | 0.00009 | 40.31 | BMI |
| whole-body lean mass | | rs4677153 | 72413112 | G | C | 0.570 | -0.007 | 0.001 | 1.20E-08 | 0.00007 | 32.51 |  |
| whole-body lean mass | | rs4858940 | 88254820 | C | T | 0.886 | 0.016 | 0.002 | 1.20E-16 | 0.00015 | 68.61 | BMI |
| whole-body lean mass | | rs34693680 | 98665549 | T | C | 0.132 | 0.014 | 0.002 | 3.30E-15 | 0.00014 | 62.11 |  |
| whole-body lean mass | | rs11709402 | 131551027 | G | A | 0.279 | 0.010 | 0.001 | 7.10E-13 | 0.00011 | 51.53 | BMI |
| whole-body lean mass | | rs4635681 | 152310614 | G | A | 0.156 | 0.011 | 0.002 | 1.10E-10 | 0.00009 | 41.57 |  |
| whole-body lean mass | | rs357486 | 153885503 | C | T | 0.543 | 0.010 | 0.001 | 6.70E-16 | 0.00014 | 65.22 | BMI |
| whole-body lean mass | | rs13085472 | 171129859 | C | T | 0.666 | -0.008 | 0.001 | 4.10E-10 | 0.00009 | 39.08 | BMI |
| whole-body lean mass | | rs9882731 | 171906978 | C | G | 0.531 | -0.012 | 0.001 | 6.10E-20 | 0.00018 | 83.58 |  |
| whole-body lean mass | | rs73052033 | 185828465 | C | T | 0.185 | -0.015 | 0.002 | 5.20E-22 | 0.00020 | 92.99 | BMI |
| whole-body lean mass | | rs6772164 | 196078149 | A | C | 0.358 | 0.008 | 0.001 | 3.10E-10 | 0.00009 | 39.60 | BMI |
| whole-body lean mass | | rs2270894 | 9975386 | G | C | 0.203 | -0.017 | 0.002 | 1.00E-27 | 0.00026 | 119.03 |  |
| whole-body lean mass | | rs6781248 | 13726684 | A | G | 0.546 | 0.008 | 0.001 | 1.30E-09 | 0.00008 | 36.81 |  |
| whole-body lean mass | | rs7647657 | 38573746 | G | A | 0.386 | -0.011 | 0.001 | 9.10E-19 | 0.00017 | 78.25 |  |
| whole-body lean mass | | rs11925245 | 114183579 | G | A | 0.183 | -0.010 | 0.002 | 1.00E-09 | 0.00008 | 37.32 |  |
| whole-body lean mass | | rs1910466 | 147086268 | C | T | 0.498 | -0.008 | 0.001 | 9.40E-10 | 0.00008 | 37.45 |  |
| whole-body lean mass | | rs34345690 | 192445575 | G | A | 0.227 | 0.008 | 0.001 | 3.50E-08 | 0.00007 | 30.40 |  |
| whole-body lean mass | | rs434072 | 12916946 | G | T | 0.709 | -0.008 | 0.001 | 4.10E-08 | 0.00007 | 30.12 | BMI |
| whole-body lean mass | | rs2365363 | 20126635 | A | T | 0.504 | 0.007 | 0.001 | 4.40E-08 | 0.00007 | 29.96 |  |
| whole-body lean mass | | rs11712872 | 52835514 | A | G | 0.115 | 0.017 | 0.002 | 4.30E-18 | 0.00017 | 75.17 |  |
| whole-body lean mass | | rs77344209 | 55515277 | T | C | 0.252 | 0.010 | 0.001 | 1.20E-11 | 0.00010 | 46.00 |  |
| whole-body lean mass | | rs4974223 | 56602088 | T | C | 0.904 | 0.016 | 0.002 | 1.00E-14 | 0.00013 | 59.86 |  |
| whole-body lean mass | | rs485554 | 172137343 | C | G | 0.315 | 0.017 | 0.001 | 1.80E-37 | 0.00036 | 163.70 |  |
| whole-body lean mass | | rs73175572 | 185490184 | G | A | 0.112 | 0.028 | 0.002 | 6.10E-45 | 0.00043 | 197.87 |  |
| whole-body lean mass | | rs7619139 | 25110415 | A | T | 0.589 | 0.012 | 0.001 | 3.40E-21 | 0.00020 | 89.29 | BMI |
| whole-body lean mass | | rs6800021 | 50190346 | A | G | 0.425 | 0.014 | 0.001 | 1.60E-27 | 0.00026 | 118.12 | BMI |
| whole-body lean mass | | rs60385590 | 53630199 | A | C | 0.258 | 0.009 | 0.001 | 1.00E-10 | 0.00009 | 41.77 |  |
| whole-body lean mass | | rs10511111 | 80599757 | C | T | 0.309 | 0.009 | 0.001 | 7.90E-11 | 0.00009 | 42.28 |  |
| whole-body lean mass | | rs6762578 | 128992047 | A | G | 0.778 | 0.016 | 0.001 | 8.90E-26 | 0.00024 | 110.20 |  |
| whole-body lean mass | | rs9853018 | 141101961 | T | C | 0.443 | 0.030 | 0.001 | 6.30E-128 | 0.00127 | 578.96 | body fat percentage |
| whole-body lean mass | | rs111391498 | 1341553 | G | A | 0.047 | -0.026 | 0.003 | 6.70E-19 | 0.00017 | 78.84 |  |
| whole-body lean mass | | rs4132132 | 31001301 | C | T | 0.444 | 0.009 | 0.001 | 4.70E-12 | 0.00011 | 47.79 | BMI |
| whole-body lean mass | | rs10020631 | 69353863 | A | G | 0.248 | -0.008 | 0.001 | 8.20E-09 | 0.00007 | 33.24 |  |
| whole-body lean mass | | rs17011108 | 86800806 | C | T | 0.307 | 0.008 | 0.001 | 2.30E-08 | 0.00007 | 31.19 |  |
| whole-body lean mass | | rs1296328 | 137083193 | C | A | 0.559 | -0.009 | 0.001 | 4.50E-12 | 0.00011 | 47.87 | BMI |
| whole-body lean mass | | rs3990738 | 152295198 | G | A | 0.543 | -0.008 | 0.001 | 6.60E-10 | 0.00008 | 38.14 |  |
| whole-body lean mass | | rs981002 | 12881731 | A | T | 0.267 | -0.011 | 0.001 | 5.30E-14 | 0.00012 | 56.62 |  |
| whole-body lean mass | | rs1841738 | 88557753 | G | A | 0.518 | -0.011 | 0.001 | 3.20E-19 | 0.00018 | 80.31 |  |
| whole-body lean mass | | rs2101975 | 106216667 | G | A | 0.432 | -0.016 | 0.001 | 7.40E-39 | 0.00037 | 170.00 |  |
| whole-body lean mass | | rs1028197 | 124977659 | T | C | 0.225 | 0.008 | 0.001 | 4.10E-08 | 0.00007 | 30.09 |  |
| whole-body lean mass | | rs9985795 | 135213286 | C | T | 0.483 | -0.007 | 0.001 | 5.10E-09 | 0.00008 | 34.17 |  |
| whole-body lean mass | | rs34227797 | 159982551 | C | G | 0.310 | 0.008 | 0.001 | 9.30E-10 | 0.00008 | 37.47 |  |
| whole-body lean mass | | rs7683836 | 180167906 | A | G | 0.557 | -0.007 | 0.001 | 3.40E-08 | 0.00007 | 30.46 | BMI |
| whole-body lean mass | | rs798759 | 1730299 | G | A | 0.464 | -0.010 | 0.001 | 6.70E-15 | 0.00013 | 60.68 |  |
| whole-body lean mass | | rs2968669 | 4889866 | G | C | 0.526 | 0.007 | 0.001 | 8.60E-09 | 0.00007 | 33.13 |  |
| whole-body lean mass | | rs7671110 | 17874089 | T | C | 0.158 | -0.032 | 0.002 | 2.80E-78 | 0.00077 | 350.80 |  |
| whole-body lean mass | | rs10939792 | 18483405 | G | C | 0.320 | 0.008 | 0.001 | 3.20E-09 | 0.00008 | 35.08 | BMI |
| whole-body lean mass | | rs17556750 | 82155568 | A | C | 0.288 | 0.012 | 0.001 | 3.70E-18 | 0.00017 | 75.50 |  |
| whole-body lean mass | | rs6821305 | 122713863 | C | A | 0.399 | 0.013 | 0.001 | 3.30E-25 | 0.00024 | 107.58 |  |
| whole-body lean mass | | rs34848742 | 123828042 | G | T | 0.788 | -0.015 | 0.002 | 2.30E-22 | 0.00021 | 94.66 |  |
| whole-body lean mass | | rs16844418 | 3471754 | C | T | 0.143 | -0.012 | 0.002 | 1.90E-12 | 0.00011 | 49.61 |  |
| whole-body lean mass | | rs73213484 | 28489339 | T | A | 0.141 | -0.012 | 0.002 | 1.50E-11 | 0.00010 | 45.47 | BMI |
| whole-body lean mass | | rs10938397 | 45182527 | G | A | 0.434 | 0.012 | 0.001 | 1.70E-21 | 0.00020 | 90.61 | BMI |
| whole-body lean mass | | rs10434434 | 54271611 | C | A | 0.148 | -0.014 | 0.002 | 2.40E-15 | 0.00014 | 62.73 |  |
| whole-body lean mass | | rs13125082 | 57787000 | G | T | 0.278 | -0.008 | 0.001 | 3.40E-08 | 0.00007 | 30.48 |  |
| whole-body lean mass | | rs11937249 | 110755321 | T | G | 0.636 | -0.008 | 0.001 | 5.10E-10 | 0.00009 | 38.65 |  |
| whole-body lean mass | | rs4240326 | 145839264 | G | A | 0.550 | -0.021 | 0.001 | 3.70E-65 | 0.00064 | 290.60 |  |
| whole-body lean mass | | rs72703409 | 184225295 | C | G | 0.066 | -0.015 | 0.003 | 2.00E-09 | 0.00008 | 35.94 |  |
| whole-body lean mass | | rs10222924 | 49016761 | G | A | 0.699 | -0.010 | 0.001 | 2.00E-12 | 0.00011 | 49.50 |  |
| whole-body lean mass | | rs140493137 | 87666916 | A | G | 0.060 | 0.019 | 0.003 | 1.10E-13 | 0.00012 | 55.16 |  |
| whole-body lean mass | | rs111598585 | 171635471 | T | C | 0.209 | -0.009 | 0.002 | 2.00E-08 | 0.00007 | 31.47 |  |
| whole-body lean mass | | rs7706886 | 32722319 | A | G | 0.271 | 0.013 | 0.001 | 6.20E-19 | 0.00017 | 79.01 |  |
| whole-body lean mass | | rs12657771 | 36787962 | A | G | 0.439 | -0.013 | 0.001 | 9.40E-27 | 0.00025 | 114.65 |  |
| whole-body lean mass | | rs62372052 | 42724294 | G | A | 0.110 | 0.027 | 0.002 | 6.80E-41 | 0.00039 | 179.31 |  |
| whole-body lean mass | | rs4865956 | 54882505 | A | T | 0.697 | -0.012 | 0.001 | 7.30E-19 | 0.00017 | 78.69 |  |
| whole-body lean mass | | rs182224 | 67316442 | T | C | 0.090 | 0.013 | 0.002 | 4.60E-09 | 0.00008 | 34.35 |  |
| whole-body lean mass | | rs9800418 | 77641008 | C | T | 0.245 | 0.008 | 0.001 | 7.90E-09 | 0.00007 | 33.30 |  |
| whole-body lean mass | | rs7728690 | 88411214 | T | C | 0.386 | -0.011 | 0.001 | 5.00E-18 | 0.00016 | 74.86 |  |
| whole-body lean mass | | rs36695 | 127549187 | A | G | 0.504 | -0.009 | 0.001 | 2.30E-13 | 0.00012 | 53.73 |  |
| whole-body lean mass | | rs13185520 | 137807260 | A | G | 0.574 | -0.007 | 0.001 | 5.40E-09 | 0.00007 | 34.06 |  |
| whole-body lean mass | | rs3822742 | 139059017 | A | C | 0.371 | 0.013 | 0.001 | 1.60E-25 | 0.00024 | 109.01 | BMI |
| whole-body lean mass | | rs3853474 | 141816555 | T | C | 0.377 | -0.008 | 0.001 | 1.30E-09 | 0.00008 | 36.81 |  |
| whole-body lean mass | | rs6874142 | 172753555 | G | T | 0.114 | 0.018 | 0.002 | 1.40E-18 | 0.00017 | 77.46 |  |
| whole-body lean mass | | rs12188627 | 60720682 | G | A | 0.488 | -0.010 | 0.001 | 6.30E-17 | 0.00015 | 69.88 |  |
| whole-body lean mass | | rs141729694 | 87999371 | T | C | 0.074 | 0.019 | 0.002 | 4.70E-16 | 0.00014 | 65.92 | BMI |
| whole-body lean mass | | rs9327336 | 123990270 | C | T | 0.343 | 0.009 | 0.001 | 3.20E-11 | 0.00010 | 44.06 |  |
| whole-body lean mass | | rs4912650 | 142723937 | G | T | 0.253 | -0.009 | 0.001 | 3.80E-11 | 0.00010 | 43.72 |  |
| whole-body lean mass | | rs111365325 | 170865229 | T | C | 0.231 | -0.014 | 0.001 | 9.60E-23 | 0.00021 | 96.35 |  |
| whole-body lean mass | | rs2578557 | 5473063 | T | C | 0.642 | -0.009 | 0.001 | 2.90E-13 | 0.00012 | 53.30 |  |
| whole-body lean mass | | rs2307111 | 75003678 | C | T | 0.395 | -0.016 | 0.001 | 2.10E-38 | 0.00037 | 167.93 | BMI |
| whole-body lean mass | | rs6235 | 95728898 | G | C | 0.268 | 0.016 | 0.001 | 8.30E-30 | 0.00028 | 128.59 | BMI |
| whole-body lean mass | | rs2611732 | 95855576 | G | A | 0.602 | 0.008 | 0.001 | 9.30E-10 | 0.00008 | 37.46 | BMI |
| whole-body lean mass | | rs2952615 | 112138888 | C | G | 0.618 | -0.012 | 0.001 | 8.90E-21 | 0.00019 | 87.39 |  |
| whole-body lean mass | | rs1582931 | 122657199 | A | G | 0.473 | -0.019 | 0.001 | 8.70E-51 | 0.00049 | 224.65 | BMI |
| whole-body lean mass | | rs247008 | 131447104 | G | A | 0.670 | 0.013 | 0.001 | 3.60E-21 | 0.00020 | 89.17 |  |
| whole-body lean mass | | rs17115481 | 153358226 | A | G | 0.270 | -0.009 | 0.001 | 1.30E-10 | 0.00009 | 41.37 | BMI |
| whole-body lean mass | | rs254963 | 171224403 | G | A | 0.447 | -0.009 | 0.001 | 6.60E-12 | 0.00010 | 47.14 |  |
| whole-body lean mass | | rs7731023 | 36181627 | G | A | 0.574 | 0.009 | 0.001 | 7.80E-12 | 0.00010 | 46.82 |  |
| whole-body lean mass | | rs9291926 | 67599656 | G | T | 0.532 | -0.012 | 0.001 | 6.10E-22 | 0.00020 | 92.70 |  |
| whole-body lean mass | | rs365352 | 77401152 | A | G | 0.244 | -0.015 | 0.001 | 9.30E-25 | 0.00023 | 105.54 | BMI |
| whole-body lean mass | | rs244711 | 176509193 | T | C | 0.686 | 0.017 | 0.001 | 1.20E-31 | 0.00030 | 136.95 |  |
| whole-body lean mass | | rs9654451 | 299543 | T | C | 0.129 | 0.011 | 0.002 | 1.40E-09 | 0.00008 | 36.71 | body fat percentage |
| whole-body lean mass | | rs72754922 | 42547714 | A | G | 0.061 | -0.016 | 0.003 | 9.10E-10 | 0.00008 | 37.50 |  |
| whole-body lean mass | | rs695922 | 53385529 | G | A | 0.837 | -0.010 | 0.002 | 1.70E-09 | 0.00008 | 36.35 |  |
| whole-body lean mass | | rs505575 | 111256812 | C | T | 0.673 | -0.009 | 0.001 | 2.60E-11 | 0.00010 | 44.44 |  |
| whole-body lean mass | | rs31211 | 134363145 | A | G | 0.251 | -0.013 | 0.001 | 7.20E-20 | 0.00018 | 83.25 |  |
| whole-body lean mass | | rs2422054 | 161382474 | A | T | 0.631 | 0.008 | 0.001 | 5.10E-10 | 0.00008 | 38.63 |  |
| whole-body lean mass | | rs4282339 | 168256240 | A | G | 0.208 | -0.019 | 0.002 | 2.40E-35 | 0.00034 | 153.91 |  |
| whole-body lean mass | | rs55758152 | 171317318 | A | G | 0.326 | 0.010 | 0.001 | 2.80E-13 | 0.00012 | 53.31 |  |
| whole-body lean mass | | rs10498672 | 7797840 | G | C | 0.176 | 0.010 | 0.002 | 1.60E-10 | 0.00009 | 40.86 |  |
| whole-body lean mass | | rs115461836 | 19078931 | C | T | 0.046 | 0.021 | 0.003 | 4.20E-12 | 0.00011 | 48.03 |  |
| whole-body lean mass | | rs41271299 | 19839415 | T | C | 0.051 | 0.042 | 0.003 | 2.30E-50 | 0.00049 | 222.72 |  |
| whole-body lean mass | | rs35612982 | 20682622 | C | T | 0.178 | -0.009 | 0.002 | 1.10E-08 | 0.00007 | 32.67 | BMI |
| whole-body lean mass | | rs578366 | 81591034 | G | A | 0.420 | -0.010 | 0.001 | 3.50E-16 | 0.00015 | 66.48 |  |
| whole-body lean mass | | rs1179905 | 90322922 | G | A | 0.195 | 0.010 | 0.002 | 6.30E-11 | 0.00009 | 42.72 |  |
| whole-body lean mass | | rs6902789 | 105358192 | A | G | 0.368 | 0.011 | 0.001 | 1.10E-16 | 0.00015 | 68.81 |  |
| whole-body lean mass | | rs9491201 | 124934212 | T | C | 0.164 | 0.010 | 0.002 | 9.50E-09 | 0.00007 | 32.94 | BMI |
| whole-body lean mass | | rs9398171 | 108983527 | T | C | 0.711 | 0.021 | 0.001 | 1.80E-52 | 0.00051 | 232.39 | BMI |
| whole-body lean mass | | rs12213409 | 142610314 | G | T | 0.485 | -0.007 | 0.001 | 4.70E-08 | 0.00007 | 29.82 |  |
| whole-body lean mass | | rs6570509 | 142716286 | T | G | 0.287 | -0.014 | 0.001 | 3.90E-25 | 0.00024 | 107.26 |  |
| whole-body lean mass | | rs73013411 | 164126233 | A | C | 0.131 | 0.013 | 0.002 | 8.90E-13 | 0.00011 | 51.07 |  |
| whole-body lean mass | | rs76307059 | 169000888 | G | C | 0.046 | -0.019 | 0.003 | 3.10E-10 | 0.00009 | 39.60 |  |
| whole-body lean mass | | rs11243202 | 7719065 | C | T | 0.486 | 0.018 | 0.001 | 6.30E-49 | 0.00047 | 216.15 |  |
| whole-body lean mass | | rs9461246 | 26381660 | A | G | 0.118 | -0.018 | 0.002 | 1.50E-20 | 0.00019 | 86.31 | body fat percentage |
| whole-body lean mass | | rs2524137 | 31264582 | T | C | 0.696 | -0.021 | 0.001 | 2.30E-52 | 0.00051 | 231.87 |  |
| whole-body lean mass | | rs2744956 | 34618937 | C | T | 0.139 | 0.038 | 0.002 | 2.90E-99 | 0.00098 | 447.21 | BMI |
| whole-body lean mass | | rs12193797 | 41748352 | G | A | 0.133 | -0.019 | 0.002 | 2.90E-24 | 0.00023 | 103.30 | BMI |
| whole-body lean mass | | rs1418433 | 44752568 | A | G | 0.588 | -0.009 | 0.001 | 1.30E-11 | 0.00010 | 45.77 |  |
| whole-body lean mass | | rs240113 | 101058886 | A | G | 0.531 | -0.008 | 0.001 | 1.10E-10 | 0.00009 | 41.66 | BMI |
| whole-body lean mass | | rs9372837 | 126638124 | A | G | 0.471 | 0.015 | 0.001 | 6.80E-34 | 0.00032 | 147.30 | body fat percentage |
| whole-body lean mass | | rs7740107 | 130374461 | A | T | 0.736 | -0.029 | 0.001 | 1.10E-95 | 0.00095 | 430.70 |  |
| whole-body lean mass | | rs7755185 | 152339615 | G | A | 0.311 | 0.009 | 0.001 | 1.00E-10 | 0.00009 | 41.83 |  |
| whole-body lean mass | | rs9380508 | 35398689 | T | C | 0.775 | 0.012 | 0.001 | 1.40E-16 | 0.00015 | 68.29 |  |
| whole-body lean mass | | rs72892910 | 50816887 | T | G | 0.172 | 0.021 | 0.002 | 2.50E-36 | 0.00035 | 158.39 | BMI |
| whole-body lean mass | | rs1266874 | 51779638 | G | A | 0.350 | 0.008 | 0.001 | 1.10E-10 | 0.00009 | 41.62 | BMI |
| whole-body lean mass | | rs9480933 | 109671391 | G | C | 0.350 | -0.013 | 0.001 | 9.30E-24 | 0.00022 | 100.97 |  |
| whole-body lean mass | | rs603321 | 140256669 | G | A | 0.245 | -0.012 | 0.001 | 1.60E-15 | 0.00014 | 63.53 |  |
| whole-body lean mass | | rs10945540 | 158738975 | A | G | 0.665 | -0.009 | 0.001 | 1.10E-12 | 0.00011 | 50.61 |  |
| whole-body lean mass | | rs9365939 | 166336825 | G | A | 0.548 | -0.007 | 0.001 | 2.50E-09 | 0.00008 | 35.52 |  |
| whole-body lean mass | | rs62439025 | 166592164 | C | G | 0.152 | 0.011 | 0.002 | 1.80E-10 | 0.00009 | 40.64 |  |
| whole-body lean mass | | rs9379084 | 7231843 | A | G | 0.116 | -0.015 | 0.002 | 2.70E-14 | 0.00013 | 57.91 |  |
| whole-body lean mass | | rs2071286 | 32179896 | T | C | 0.182 | -0.021 | 0.002 | 4.20E-39 | 0.00038 | 171.12 |  |
| whole-body lean mass | | rs9394166 | 33751122 | C | G | 0.380 | 0.009 | 0.001 | 3.80E-12 | 0.00011 | 48.24 |  |
| whole-body lean mass | | rs12209223 | 76164589 | A | C | 0.101 | 0.015 | 0.002 | 2.60E-13 | 0.00012 | 53.53 |  |
| whole-body lean mass | | rs9350850 | 81050236 | C | T | 0.079 | 0.023 | 0.002 | 1.50E-24 | 0.00023 | 104.56 |  |
| whole-body lean mass | | rs12200061 | 83487443 | A | G | 0.405 | -0.007 | 0.001 | 3.50E-09 | 0.00008 | 34.90 |  |
| whole-body lean mass | | rs6900690 | 126223032 | A | G | 0.562 | 0.008 | 0.001 | 1.10E-10 | 0.00009 | 41.60 |  |
| whole-body lean mass | | rs3853252 | 152170247 | A | G | 0.455 | 0.015 | 0.001 | 1.70E-34 | 0.00033 | 149.98 |  |
| whole-body lean mass | | rs34776209 | 23513093 | T | C | 0.247 | -0.017 | 0.001 | 1.50E-32 | 0.00031 | 141.11 |  |
| whole-body lean mass | | rs4729085 | 76592670 | G | C | 0.868 | -0.016 | 0.002 | 2.60E-18 | 0.00017 | 76.14 | BMI |
| whole-body lean mass | | rs7780752 | 93241640 | C | T | 0.360 | 0.012 | 0.001 | 5.40E-22 | 0.00020 | 92.93 | BMI |
| whole-body lean mass | | rs12375196 | 103416541 | A | C | 0.424 | 0.009 | 0.001 | 6.50E-14 | 0.00012 | 56.21 | BMI |
| whole-body lean mass | | rs73158212 | 148383377 | G | A | 0.224 | -0.008 | 0.002 | 2.50E-08 | 0.00007 | 31.06 |  |
| whole-body lean mass | | rs114949263 | 150498245 | C | T | 0.112 | -0.013 | 0.002 | 2.50E-11 | 0.00010 | 44.53 |  |
| whole-body lean mass | | rs10282707 | 17911038 | T | C | 0.397 | 0.008 | 0.001 | 3.60E-10 | 0.00009 | 39.30 |  |
| whole-body lean mass | | rs2122823 | 25939161 | T | C | 0.211 | 0.010 | 0.002 | 2.00E-11 | 0.00010 | 44.96 |  |
| whole-body lean mass | | rs508347 | 28212824 | C | T | 0.703 | -0.012 | 0.001 | 7.80E-18 | 0.00016 | 74.00 |  |
| whole-body lean mass | | rs10950207 | 70105168 | T | C | 0.384 | 0.008 | 0.001 | 5.80E-11 | 0.00009 | 42.89 | BMI |
| whole-body lean mass | | rs13240065 | 73015369 | A | G | 0.128 | 0.018 | 0.002 | 2.90E-22 | 0.00021 | 94.16 |  |
| whole-body lean mass | | rs62621812 | 127015083 | A | G | 0.020 | 0.043 | 0.005 | 5.90E-21 | 0.00019 | 88.20 | BMI |
| whole-body lean mass | | rs12533548 | 148660579 | G | A | 0.651 | 0.008 | 0.001 | 4.60E-10 | 0.00009 | 38.83 |  |
| whole-body lean mass | | rs2533879 | 2859847 | A | G | 0.300 | -0.021 | 0.001 | 3.40E-52 | 0.00051 | 231.14 | body fat percentage |
| whole-body lean mass | | rs836511 | 6458319 | G | A | 0.199 | 0.010 | 0.002 | 2.00E-11 | 0.00010 | 45.01 | BMI |
| whole-body lean mass | | rs37964 | 8004981 | T | A | 0.466 | 0.008 | 0.001 | 1.30E-11 | 0.00010 | 45.79 |  |
| whole-body lean mass | | rs77760034 | 46606906 | A | T | 0.075 | -0.019 | 0.002 | 1.40E-15 | 0.00014 | 63.82 |  |
| whole-body lean mass | | rs10269774 | 92253972 | A | G | 0.326 | 0.026 | 0.001 | 4.70E-87 | 0.00086 | 391.15 |  |
| whole-body lean mass | | rs62466118 | 92716556 | A | G | 0.028 | -0.025 | 0.004 | 1.40E-10 | 0.00009 | 41.12 |  |
| whole-body lean mass | | rs62460525 | 44902676 | A | C | 0.070 | 0.014 | 0.002 | 1.70E-08 | 0.00007 | 31.82 |  |
| whole-body lean mass | | rs2740761 | 55255986 | T | C | 0.208 | 0.009 | 0.002 | 1.70E-08 | 0.00007 | 31.83 |  |
| whole-body lean mass | | rs6972291 | 55802063 | C | T | 0.176 | -0.011 | 0.002 | 7.70E-12 | 0.00010 | 46.84 |  |
| whole-body lean mass | | rs2293177 | 140244560 | T | C | 0.313 | 0.007 | 0.001 | 2.90E-08 | 0.00007 | 30.79 |  |
| whole-body lean mass | | rs6946415 | 150684548 | G | A | 0.627 | 0.019 | 0.001 | 6.30E-49 | 0.00047 | 216.13 | BMI |
| whole-body lean mass | | rs6946419 | 28768853 | C | T | 0.377 | -0.008 | 0.001 | 7.20E-10 | 0.00008 | 37.97 |  |
| whole-body lean mass | | rs2881198 | 46634506 | C | G | 0.531 | -0.012 | 0.001 | 7.80E-23 | 0.00021 | 96.76 |  |
| whole-body lean mass | | rs10899736 | 50546925 | A | G | 0.440 | -0.010 | 0.001 | 4.40E-16 | 0.00015 | 66.07 | BMI |
| whole-body lean mass | | rs236628 | 75023850 | T | G | 0.236 | 0.009 | 0.002 | 6.80E-10 | 0.00008 | 38.08 | BMI |
| whole-body lean mass | | rs10248298 | 121963813 | A | C | 0.366 | 0.011 | 0.001 | 8.50E-19 | 0.00017 | 78.38 | BMI |
| whole-body lean mass | | rs822549 | 148649180 | C | T | 0.267 | 0.012 | 0.001 | 1.40E-17 | 0.00016 | 72.89 |  |
| whole-body lean mass | | rs76364830 | 13372120 | A | G | 0.063 | -0.022 | 0.003 | 2.40E-18 | 0.00017 | 76.36 |  |
| whole-body lean mass | | rs4439140 | 23113908 | A | G | 0.418 | -0.008 | 0.001 | 4.70E-11 | 0.00010 | 43.30 |  |
| whole-body lean mass | | rs11997525 | 49401982 | A | T | 0.167 | 0.015 | 0.002 | 3.90E-20 | 0.00019 | 84.45 |  |
| whole-body lean mass | | rs72656010 | 57122215 | C | T | 0.132 | -0.037 | 0.002 | 2.80E-88 | 0.00087 | 396.74 |  |
| whole-body lean mass | | rs61729527 | 77761919 | T | C | 0.052 | -0.024 | 0.003 | 1.90E-17 | 0.00016 | 72.28 | BMI |
| whole-body lean mass | | rs7842996 | 78107140 | A | T | 0.284 | 0.019 | 0.001 | 7.70E-44 | 0.00042 | 192.83 |  |
| whole-body lean mass | | rs894360 | 135601194 | C | T | 0.366 | -0.019 | 0.001 | 5.70E-49 | 0.00048 | 216.33 |  |
| whole-body lean mass | | rs7843128 | 22473465 | C | T | 0.357 | -0.008 | 0.001 | 2.00E-10 | 0.00009 | 40.50 |  |
| whole-body lean mass | | rs2979649 | 6315856 | G | A | 0.174 | 0.010 | 0.002 | 3.60E-09 | 0.00008 | 34.81 |  |
| whole-body lean mass | | rs13264909 | 64702385 | T | A | 0.429 | -0.009 | 0.001 | 9.50E-12 | 0.00010 | 46.43 | BMI |
| whole-body lean mass | | rs16916881 | 95566270 | A | C | 0.235 | -0.010 | 0.001 | 9.20E-13 | 0.00011 | 51.02 | BMI |
| whole-body lean mass | | rs11782103 | 120451323 | A | T | 0.252 | -0.013 | 0.001 | 9.20E-20 | 0.00018 | 82.77 |  |
| whole-body lean mass | | rs62515437 | 57160328 | T | G | 0.225 | 0.019 | 0.001 | 1.40E-38 | 0.00037 | 168.68 |  |
| whole-body lean mass | | rs55674305 | 89435868 | A | G | 0.306 | -0.011 | 0.001 | 3.10E-16 | 0.00015 | 66.75 | BMI |
| whole-body lean mass | | rs2721938 | 116635611 | T | C | 0.600 | -0.012 | 0.001 | 1.30E-21 | 0.00020 | 91.24 |  |
| whole-body lean mass | | rs12156265 | 144848756 | A | G | 0.590 | 0.008 | 0.001 | 1.10E-10 | 0.00009 | 41.65 |  |
| whole-body lean mass | | rs13272451 | 8886183 | T | G | 0.439 | 0.009 | 0.001 | 8.80E-14 | 0.00012 | 55.62 | BMI |
| whole-body lean mass | | rs11785562 | 23391493 | A | G | 0.201 | 0.012 | 0.002 | 2.70E-14 | 0.00013 | 57.95 |  |
| whole-body lean mass | | rs3925 | 38281658 | A | G | 0.245 | -0.011 | 0.001 | 1.20E-13 | 0.00012 | 55.03 |  |
| whole-body lean mass | | rs17828687 | 73454767 | A | C | 0.573 | -0.009 | 0.001 | 6.80E-13 | 0.00011 | 51.59 | BMI |
| whole-body lean mass | | rs10283100 | 120596023 | G | A | 0.945 | 0.028 | 0.003 | 8.70E-25 | 0.00023 | 105.66 |  |
| whole-body lean mass | | rs13271368 | 126506140 | T | C | 0.237 | -0.015 | 0.001 | 2.00E-23 | 0.00022 | 99.44 |  |
| whole-body lean mass | | rs7815955 | 130719567 | T | A | 0.203 | -0.015 | 0.002 | 7.80E-23 | 0.00021 | 96.78 |  |
| whole-body lean mass | | rs6984820 | 144993324 | T | C | 0.422 | -0.010 | 0.001 | 8.50E-16 | 0.00014 | 64.74 |  |
| whole-body lean mass | | rs1412234 | 28410683 | C | T | 0.327 | 0.011 | 0.001 | 2.60E-16 | 0.00015 | 67.05 | BMI |
| whole-body lean mass | | rs10780905 | 90841066 | A | G | 0.588 | -0.010 | 0.001 | 8.40E-15 | 0.00013 | 60.24 |  |
| whole-body lean mass | | rs663344 | 98316331 | C | G | 0.181 | -0.015 | 0.002 | 3.70E-20 | 0.00019 | 84.58 |  |
| whole-body lean mass | | rs10119967 | 99268421 | C | A | 0.204 | 0.016 | 0.002 | 5.90E-24 | 0.00022 | 101.88 |  |
| whole-body lean mass | | rs7033487 | 119129257 | C | T | 0.198 | -0.023 | 0.002 | 4.50E-51 | 0.00050 | 225.99 |  |
| whole-body lean mass | | rs56141370 | 127056213 | T | C | 0.115 | 0.013 | 0.002 | 1.00E-10 | 0.00009 | 41.74 |  |
| whole-body lean mass | | rs13299559 | 128041828 | T | C | 0.450 | -0.010 | 0.001 | 5.20E-16 | 0.00014 | 65.72 |  |
| whole-body lean mass | | rs11794152 | 23345347 | G | A | 0.415 | 0.010 | 0.001 | 2.40E-16 | 0.00015 | 67.26 |  |
| whole-body lean mass | | rs12553221 | 37234008 | A | G | 0.362 | 0.007 | 0.001 | 2.20E-08 | 0.00007 | 31.31 | BMI |
| whole-body lean mass | | rs11142700 | 73757155 | C | T | 0.408 | -0.008 | 0.001 | 3.30E-10 | 0.00009 | 39.49 |  |
| whole-body lean mass | | rs28620532 | 98216876 | G | A | 0.349 | 0.018 | 0.001 | 6.40E-44 | 0.00042 | 193.19 |  |
| whole-body lean mass | | rs1056747 | 35690102 | G | A | 0.412 | -0.008 | 0.001 | 3.40E-11 | 0.00010 | 43.93 |  |
| whole-body lean mass | | rs10881583 | 137267439 | C | T | 0.241 | -0.009 | 0.001 | 3.40E-10 | 0.00009 | 39.43 |  |
| whole-body lean mass | | rs2274116 | 139094805 | T | C | 0.341 | -0.008 | 0.001 | 8.30E-10 | 0.00008 | 37.69 |  |
| whole-body lean mass | | rs1927635 | 16461905 | C | T | 0.354 | 0.009 | 0.001 | 5.00E-12 | 0.00010 | 47.69 |  |
| whole-body lean mass | | rs1928850 | 16765520 | T | A | 0.922 | 0.014 | 0.002 | 1.00E-09 | 0.00008 | 37.32 |  |
| whole-body lean mass | | rs968821 | 94187634 | C | G | 0.340 | -0.011 | 0.001 | 2.10E-17 | 0.00016 | 72.06 | BMI |
| whole-body lean mass | | rs10979612 | 111687379 | C | T | 0.073 | 0.019 | 0.002 | 4.90E-15 | 0.00013 | 61.28 |  |
| whole-body lean mass | | rs1008158 | 113828811 | G | A | 0.342 | 0.008 | 0.001 | 1.10E-10 | 0.00009 | 41.58 |  |
| whole-body lean mass | | rs7095768 | 53256505 | T | G | 0.263 | 0.008 | 0.001 | 2.70E-08 | 0.00007 | 30.89 |  |
| whole-body lean mass | | rs3740591 | 70287303 | T | C | 0.445 | 0.011 | 0.001 | 1.70E-19 | 0.00018 | 81.52 |  |
| whole-body lean mass | | rs1268947 | 121132631 | C | G | 0.126 | 0.012 | 0.002 | 8.90E-11 | 0.00009 | 42.06 |  |
| whole-body lean mass | | rs117543413 | 79543740 | T | C | 0.018 | -0.038 | 0.005 | 1.20E-15 | 0.00014 | 64.03 |  |
| whole-body lean mass | | rs11245450 | 126658075 | A | G | 0.422 | -0.010 | 0.001 | 7.00E-16 | 0.00014 | 65.15 | BMI |
| whole-body lean mass | | rs11014285 | 25178864 | A | G | 0.165 | 0.019 | 0.002 | 1.10E-29 | 0.00028 | 128.07 |  |
| whole-body lean mass | | rs572474 | 89791654 | G | A | 0.166 | 0.010 | 0.002 | 2.00E-09 | 0.00008 | 35.95 |  |
| whole-body lean mass | | rs7080472 | 96012950 | T | G | 0.425 | 0.013 | 0.001 | 5.20E-25 | 0.00023 | 106.70 | body fat percentage |
| whole-body lean mass | | rs2265309 | 104487871 | C | T | 0.517 | -0.012 | 0.001 | 1.50E-23 | 0.00022 | 99.97 |  |
| whole-body lean mass | | rs11593630 | 120491506 | T | C | 0.353 | -0.009 | 0.001 | 1.20E-11 | 0.00010 | 45.96 |  |
| whole-body lean mass | | rs6585827 | 124165615 | A | G | 0.471 | 0.011 | 0.001 | 3.80E-20 | 0.00019 | 84.51 |  |
| whole-body lean mass | | rs1421035 | 131136401 | T | C | 0.348 | 0.008 | 0.001 | 8.00E-09 | 0.00007 | 33.28 | BMI |
| whole-body lean mass | | rs1846221 | 4667302 | C | G | 0.175 | -0.010 | 0.002 | 9.70E-09 | 0.00007 | 32.91 |  |
| whole-body lean mass | | rs12764498 | 5042294 | C | T | 0.118 | -0.015 | 0.002 | 1.60E-15 | 0.00014 | 63.56 |  |
| whole-body lean mass | | rs2807742 | 28781367 | A | G | 0.771 | -0.009 | 0.001 | 8.60E-10 | 0.00008 | 37.62 |  |
| whole-body lean mass | | rs10995366 | 52772113 | A | G | 0.251 | -0.010 | 0.001 | 1.10E-11 | 0.00010 | 46.08 |  |
| whole-body lean mass | | rs11001399 | 77221744 | C | A | 0.559 | -0.011 | 0.001 | 5.20E-19 | 0.00017 | 79.36 |  |
| whole-body lean mass | | rs4980067 | 81136129 | A | C | 0.505 | -0.011 | 0.001 | 1.80E-19 | 0.00018 | 81.41 |  |
| whole-body lean mass | | rs7912286 | 114693230 | G | A | 0.602 | -0.009 | 0.001 | 1.30E-11 | 0.00010 | 45.81 |  |
| whole-body lean mass | | rs73601548 | 18549889 | T | C | 0.115 | 0.014 | 0.002 | 4.80E-13 | 0.00011 | 52.29 | BMI |
| whole-body lean mass | | rs12146350 | 22843111 | G | C | 0.602 | -0.008 | 0.001 | 2.30E-10 | 0.00009 | 40.22 |  |
| whole-body lean mass | | rs1657222 | 34837139 | A | G | 0.396 | 0.008 | 0.001 | 1.70E-10 | 0.00009 | 40.75 |  |
| whole-body lean mass | | rs224048 | 64491549 | A | G | 0.550 | -0.007 | 0.001 | 3.80E-09 | 0.00008 | 34.72 |  |
| whole-body lean mass | | rs10883560 | 102673707 | G | C | 0.429 | 0.017 | 0.001 | 1.50E-42 | 0.00041 | 186.87 | BMI |
| whole-body lean mass | | rs61878760 | 12807189 | A | G | 0.083 | 0.015 | 0.002 | 2.80E-11 | 0.00010 | 44.29 |  |
| whole-body lean mass | | rs112758380 | 14296329 | A | G | 0.057 | 0.017 | 0.003 | 2.10E-10 | 0.00009 | 40.38 |  |
| whole-body lean mass | | rs676105 | 30443688 | C | T | 0.304 | 0.014 | 0.001 | 7.80E-24 | 0.00022 | 101.34 | BMI |
| whole-body lean mass | | rs294382 | 30607515 | C | A | 0.720 | -0.008 | 0.001 | 4.00E-08 | 0.00007 | 30.14 |  |
| whole-body lean mass | | rs35251247 | 43878459 | A | G | 0.292 | 0.011 | 0.001 | 3.80E-15 | 0.00014 | 61.78 | BMI |
| whole-body lean mass | | rs7129320 | 68388220 | A | G | 0.166 | -0.023 | 0.002 | 1.10E-43 | 0.00042 | 192.06 |  |
| whole-body lean mass | | rs667515 | 69449076 | C | G | 0.386 | -0.010 | 0.001 | 5.90E-16 | 0.00014 | 65.47 | BMI |
| whole-body lean mass | | rs74048171 | 2093603 | A | C | 0.258 | -0.010 | 0.001 | 3.20E-13 | 0.00012 | 53.09 |  |
| whole-body lean mass | | rs11030112 | 27705188 | A | G | 0.319 | 0.017 | 0.001 | 3.60E-36 | 0.00035 | 157.72 | BMI |
| whole-body lean mass | | rs4268495 | 28645341 | C | G | 0.374 | -0.009 | 0.001 | 2.20E-11 | 0.00010 | 44.75 | BMI |
| whole-body lean mass | | rs34345560 | 69081998 | A | G | 0.195 | 0.009 | 0.002 | 1.20E-08 | 0.00007 | 32.43 |  |
| whole-body lean mass | | rs12795042 | 133658168 | C | A | 0.622 | 0.007 | 0.001 | 2.30E-08 | 0.00007 | 31.26 |  |
| whole-body lean mass | | rs11042725 | 10325325 | A | C | 0.488 | -0.012 | 0.001 | 3.10E-21 | 0.00020 | 89.49 | body fat percentage |
| whole-body lean mass | | rs1228024 | 47951353 | A | C | 0.661 | -0.011 | 0.001 | 2.10E-16 | 0.00015 | 67.52 |  |
| whole-body lean mass | | rs56207600 | 126196537 | A | G | 0.111 | 0.012 | 0.002 | 9.60E-10 | 0.00008 | 37.41 |  |
| whole-body lean mass | | rs35506085 | 2165576 | A | G | 0.185 | -0.020 | 0.002 | 8.10E-35 | 0.00033 | 151.50 |  |
| whole-body lean mass | | rs11021307 | 95501407 | T | C | 0.418 | 0.007 | 0.001 | 3.60E-08 | 0.00007 | 30.36 |  |
| whole-body lean mass | | rs6591 | 840363 | T | C | 0.544 | 0.008 | 0.001 | 1.90E-10 | 0.00009 | 40.56 | BMI |
| whole-body lean mass | | rs7952436 | 67024534 | T | C | 0.082 | -0.034 | 0.002 | 6.70E-50 | 0.00048 | 220.60 |  |
| whole-body lean mass | | rs744205 | 69929677 | A | G | 0.544 | 0.011 | 0.001 | 1.00E-17 | 0.00016 | 73.52 |  |
| whole-body lean mass | | rs1813212 | 89301382 | G | A | 0.446 | -0.009 | 0.001 | 6.70E-12 | 0.00010 | 47.10 |  |
| whole-body lean mass | | rs76693355 | 120292477 | C | T | 0.116 | -0.014 | 0.002 | 9.00E-14 | 0.00012 | 55.57 |  |
| whole-body lean mass | | rs2900208 | 11878464 | A | C | 0.354 | 0.014 | 0.001 | 4.90E-27 | 0.00025 | 115.94 |  |
| whole-body lean mass | | rs10843397 | 29529523 | T | C | 0.242 | 0.009 | 0.001 | 7.60E-11 | 0.00009 | 42.36 |  |
| whole-body lean mass | | rs11052457 | 33268956 | T | A | 0.038 | 0.023 | 0.003 | 2.10E-12 | 0.00011 | 49.36 |  |
| whole-body lean mass | | rs2897968 | 46763686 | A | G | 0.607 | 0.016 | 0.001 | 1.40E-34 | 0.00033 | 150.40 |  |
| whole-body lean mass | | rs3730071 | 49168798 | A | C | 0.030 | -0.023 | 0.004 | 3.20E-10 | 0.00009 | 39.57 | body fat percentage |
| whole-body lean mass | | rs3782232 | 57116249 | A | G | 0.071 | -0.020 | 0.002 | 4.40E-17 | 0.00016 | 70.59 |  |
| whole-body lean mass | | rs1351394 | 66351826 | C | T | 0.512 | -0.026 | 0.001 | 5.30E-96 | 0.00095 | 432.22 |  |
| whole-body lean mass | | rs1168768 | 66509650 | T | C | 0.975 | 0.022 | 0.004 | 2.60E-08 | 0.00007 | 31.01 |  |
| whole-body lean mass | | rs9634212 | 93993266 | A | C | 0.221 | 0.024 | 0.002 | 4.00E-56 | 0.00055 | 249.16 |  |
| whole-body lean mass | | rs7312646 | 102388920 | C | A | 0.521 | -0.011 | 0.001 | 9.20E-17 | 0.00015 | 69.13 |  |
| whole-body lean mass | | rs34825238 | 103328352 | T | G | 0.255 | 0.009 | 0.001 | 7.10E-10 | 0.00008 | 37.98 |  |
| whole-body lean mass | | rs2287163 | 107349294 | T | C | 0.374 | -0.010 | 0.001 | 8.40E-15 | 0.00013 | 60.23 |  |
| whole-body lean mass | | rs10773172 | 122939655 | A | G | 0.742 | 0.017 | 0.001 | 6.60E-35 | 0.00033 | 151.91 | BMI |
| whole-body lean mass | | rs12820906 | 123493123 | G | A | 0.247 | 0.012 | 0.001 | 3.90E-16 | 0.00015 | 66.29 |  |
| whole-body lean mass | | rs11613704 | 980925 | A | C | 0.200 | 0.016 | 0.002 | 2.10E-24 | 0.00023 | 103.92 | BMI |
| whole-body lean mass | | rs67551338 | 3393100 | T | C | 0.061 | 0.026 | 0.003 | 1.40E-22 | 0.00021 | 95.56 |  |
| whole-body lean mass | | rs76895963 | 4384844 | G | T | 0.021 | 0.095 | 0.005 | 2.80E-87 | 0.00086 | 392.18 | BMI |
| whole-body lean mass | | rs10770705 | 20857467 | C | A | 0.663 | -0.008 | 0.001 | 2.80E-10 | 0.00009 | 39.78 |  |
| whole-body lean mass | | rs7134283 | 24071748 | A | G | 0.283 | -0.012 | 0.001 | 1.20E-17 | 0.00016 | 73.19 |  |
| whole-body lean mass | | rs774214 | 56918566 | C | T | 0.663 | -0.009 | 0.001 | 7.20E-12 | 0.00010 | 46.98 |  |
| whole-body lean mass | | rs310796 | 77453226 | T | G | 0.681 | 0.011 | 0.001 | 2.10E-16 | 0.00015 | 67.50 |  |
| whole-body lean mass | | rs2229840 | 124826462 | T | C | 0.160 | 0.020 | 0.002 | 1.50E-33 | 0.00032 | 145.71 |  |
| whole-body lean mass | | rs10848288 | 131600054 | T | C | 0.304 | -0.008 | 0.001 | 1.90E-08 | 0.00007 | 31.63 |  |
| whole-body lean mass | | rs117451679 | 17245591 | G | A | 0.106 | 0.013 | 0.002 | 5.00E-10 | 0.00009 | 38.66 |  |
| whole-body lean mass | | rs7132908 | 50263148 | A | G | 0.384 | 0.016 | 0.001 | 2.90E-35 | 0.00034 | 153.59 | BMI |
| whole-body lean mass | | rs703593 | 103064247 | G | A | 0.482 | -0.011 | 0.001 | 4.10E-20 | 0.00019 | 84.38 |  |
| whole-body lean mass | | rs11065015 | 120520863 | T | C | 0.027 | -0.028 | 0.004 | 4.10E-13 | 0.00012 | 52.59 | body fat percentage |
| whole-body lean mass | | rs4980826 | 578349 | A | C | 0.396 | 0.008 | 0.001 | 9.30E-10 | 0.00008 | 37.46 |  |
| whole-body lean mass | | rs73129767 | 66131393 | A | C | 0.208 | -0.010 | 0.002 | 3.30E-11 | 0.00010 | 44.00 |  |
| whole-body lean mass | | rs7301341 | 94083105 | C | T | 0.327 | -0.010 | 0.001 | 9.30E-15 | 0.00013 | 60.04 |  |
| whole-body lean mass | | rs11833839 | 132560957 | T | C | 0.057 | 0.017 | 0.003 | 6.80E-10 | 0.00008 | 38.09 |  |
| whole-body lean mass | | rs35756741 | 12868701 | T | C | 0.092 | -0.016 | 0.002 | 1.60E-13 | 0.00012 | 54.44 |  |
| whole-body lean mass | | rs11049684 | 28665359 | T | C | 0.299 | -0.011 | 0.001 | 4.80E-15 | 0.00013 | 61.35 |  |
| whole-body lean mass | | rs1049193 | 53873844 | C | G | 0.171 | -0.013 | 0.002 | 4.60E-16 | 0.00014 | 65.96 |  |
| whole-body lean mass | | rs10748128 | 69827658 | T | G | 0.345 | 0.012 | 0.001 | 3.20E-21 | 0.00020 | 89.45 |  |
| whole-body lean mass | | rs12427047 | 90213070 | T | C | 0.243 | -0.010 | 0.001 | 2.10E-11 | 0.00010 | 44.91 | BMI |
| whole-body lean mass | | rs10777860 | 97792792 | A | G | 0.528 | -0.011 | 0.001 | 7.80E-18 | 0.00016 | 74.00 | BMI |
| whole-body lean mass | | rs11065979 | 112059557 | T | C | 0.438 | -0.013 | 0.001 | 4.30E-27 | 0.00026 | 116.21 |  |
| whole-body lean mass | | rs1971955 | 122420437 | G | A | 0.854 | -0.014 | 0.002 | 2.90E-15 | 0.00014 | 62.30 |  |
| whole-body lean mass | | rs11611651 | 133380790 | A | G | 0.088 | 0.017 | 0.002 | 4.30E-14 | 0.00013 | 57.04 |  |
| whole-body lean mass | | rs7994814 | 27054201 | T | C | 0.417 | 0.011 | 0.001 | 5.70E-18 | 0.00016 | 74.61 |  |
| whole-body lean mass | | rs1218826 | 27995760 | G | A | 0.666 | 0.009 | 0.001 | 5.00E-12 | 0.00010 | 47.69 | BMI |
| whole-body lean mass | | rs9317002 | 59175727 | A | C | 0.515 | 0.011 | 0.001 | 2.30E-17 | 0.00016 | 71.84 | BMI |
| whole-body lean mass | | rs10870597 | 114999636 | G | A | 0.235 | -0.010 | 0.001 | 1.00E-12 | 0.00011 | 50.81 |  |
| whole-body lean mass | | rs2225226 | 51127270 | T | C | 0.216 | -0.030 | 0.002 | 3.10E-86 | 0.00085 | 387.37 |  |
| whole-body lean mass | | rs7321045 | 97034410 | A | G | 0.449 | 0.009 | 0.001 | 1.30E-11 | 0.00010 | 45.83 | BMI |
| whole-body lean mass | | rs146851424 | 50377910 | C | A | 0.022 | 0.060 | 0.004 | 1.60E-43 | 0.00042 | 191.34 |  |
| whole-body lean mass | | rs9596810 | 54056553 | T | C | 0.435 | -0.009 | 0.001 | 4.70E-12 | 0.00011 | 47.81 | BMI |
| whole-body lean mass | | rs9540493 | 66205704 | G | A | 0.545 | -0.009 | 0.001 | 1.50E-12 | 0.00011 | 50.09 | BMI |
| whole-body lean mass | | rs1924936 | 78443297 | A | T | 0.774 | 0.015 | 0.001 | 7.80E-25 | 0.00023 | 105.88 |  |
| whole-body lean mass | | rs8002779 | 92015977 | A | G | 0.603 | -0.008 | 0.001 | 1.50E-09 | 0.00008 | 36.49 |  |
| whole-body lean mass | | rs7994783 | 41661922 | G | T | 0.302 | -0.008 | 0.001 | 4.30E-10 | 0.00009 | 38.96 |  |
| whole-body lean mass | | rs1176314 | 81221014 | G | T | 0.550 | -0.007 | 0.001 | 3.60E-09 | 0.00008 | 34.85 |  |
| whole-body lean mass | | rs9513510 | 99571922 | C | G | 0.699 | -0.011 | 0.001 | 1.00E-15 | 0.00014 | 64.41 |  |
| whole-body lean mass | | rs3783256 | 30107388 | C | T | 0.648 | -0.009 | 0.001 | 6.70E-13 | 0.00011 | 51.64 |  |
| whole-body lean mass | | rs6563808 | 40766662 | C | T | 0.734 | -0.009 | 0.001 | 1.70E-09 | 0.00008 | 36.33 | BMI |
| whole-body lean mass | | rs67141907 | 42805360 | T | C | 0.149 | 0.011 | 0.002 | 1.70E-10 | 0.00009 | 40.82 |  |
| whole-body lean mass | | rs3212260 | 24804088 | T | A | 0.258 | 0.013 | 0.001 | 3.60E-20 | 0.00019 | 84.64 |  |
| whole-body lean mass | | rs2296316 | 65520246 | C | T | 0.465 | -0.010 | 0.001 | 1.20E-16 | 0.00015 | 68.56 |  |
| whole-body lean mass | | rs6575340 | 94023972 | A | G | 0.636 | 0.008 | 0.001 | 3.60E-10 | 0.00009 | 39.30 | BMI |
| whole-body lean mass | | rs17197114 | 21894526 | C | T | 0.177 | 0.011 | 0.002 | 3.40E-11 | 0.00010 | 43.93 |  |
| whole-body lean mass | | rs12889702 | 42894143 | C | A | 0.313 | 0.009 | 0.001 | 6.40E-12 | 0.00010 | 47.21 |  |
| whole-body lean mass | | rs2884345 | 53323211 | A | G | 0.710 | -0.008 | 0.001 | 2.10E-08 | 0.00007 | 31.37 |  |
| whole-body lean mass | | rs7141420 | 79899454 | T | C | 0.516 | 0.013 | 0.001 | 1.20E-25 | 0.00024 | 109.55 | BMI |
| whole-body lean mass | | rs12879423 | 25927832 | G | A | 0.679 | 0.014 | 0.001 | 3.40E-26 | 0.00025 | 112.09 | BMI |
| whole-body lean mass | | rs8007644 | 35218831 | A | G | 0.388 | 0.008 | 0.001 | 3.40E-10 | 0.00009 | 39.41 |  |
| whole-body lean mass | | rs112957890 | 36220876 | G | A | 0.265 | 0.010 | 0.001 | 2.10E-11 | 0.00010 | 44.83 |  |
| whole-body lean mass | | rs112560164 | 93112924 | A | G | 0.191 | 0.013 | 0.002 | 1.30E-16 | 0.00015 | 68.48 | body fat percentage |
| whole-body lean mass | | rs11160601 | 101186641 | T | C | 0.092 | 0.016 | 0.002 | 6.50E-13 | 0.00011 | 51.68 | BMI |
| whole-body lean mass | | rs61992671 | 101531854 | G | A | 0.492 | -0.011 | 0.001 | 1.40E-16 | 0.00015 | 68.25 | BMI |
| whole-body lean mass | | rs9783665 | 103271158 | A | T | 0.653 | -0.007 | 0.001 | 1.30E-08 | 0.00007 | 32.31 | BMI |
| whole-body lean mass | | rs36100359 | 21578007 | A | G | 0.139 | -0.012 | 0.002 | 1.40E-10 | 0.00009 | 41.19 |  |
| whole-body lean mass | | rs9788443 | 23878279 | C | T | 0.048 | 0.019 | 0.003 | 5.80E-11 | 0.00009 | 42.87 |  |
| whole-body lean mass | | rs4899012 | 61003889 | C | G | 0.607 | -0.021 | 0.001 | 1.10E-63 | 0.00062 | 283.80 |  |
| whole-body lean mass | | rs7150606 | 68413076 | T | G | 0.142 | -0.010 | 0.002 | 3.40E-08 | 0.00007 | 30.46 |  |
| whole-body lean mass | | rs2332175 | 70345411 | A | G | 0.547 | -0.008 | 0.001 | 9.60E-10 | 0.00008 | 37.41 |  |
| whole-body lean mass | | rs4906203 | 102928991 | T | C | 0.234 | -0.009 | 0.001 | 2.60E-10 | 0.00009 | 39.97 |  |
| whole-body lean mass | | rs56130943 | 105906522 | C | A | 0.233 | 0.009 | 0.002 | 2.90E-09 | 0.00008 | 35.28 |  |
| whole-body lean mass | | rs11855017 | 42096146 | A | C | 0.182 | 0.013 | 0.002 | 4.80E-16 | 0.00014 | 65.87 | BMI |
| whole-body lean mass | | rs113978196 | 75916965 | A | G | 0.251 | -0.008 | 0.001 | 1.80E-08 | 0.00007 | 31.75 |  |
| whole-body lean mass | | rs62621400 | 101718239 | G | C | 0.058 | -0.020 | 0.003 | 9.80E-14 | 0.00012 | 55.41 |  |
| whole-body lean mass | | rs2663125 | 99563891 | C | T | 0.691 | -0.009 | 0.001 | 3.20E-11 | 0.00010 | 44.02 |  |
| whole-body lean mass | | rs11071182 | 55644676 | G | A | 0.870 | 0.013 | 0.002 | 6.30E-13 | 0.00011 | 51.74 |  |
| whole-body lean mass | | rs338361 | 68171116 | C | T | 0.706 | 0.009 | 0.001 | 3.90E-12 | 0.00011 | 48.15 | BMI |
| whole-body lean mass | | rs12906197 | 38492199 | T | C | 0.422 | -0.011 | 0.001 | 9.40E-18 | 0.00016 | 73.63 |  |
| whole-body lean mass | | rs76875574 | 40376098 | A | C | 0.054 | -0.016 | 0.003 | 2.70E-08 | 0.00007 | 30.94 |  |
| whole-body lean mass | | rs6493534 | 52379736 | C | T | 0.580 | 0.007 | 0.001 | 3.60E-08 | 0.00007 | 30.35 | BMI |
| whole-body lean mass | | rs933807 | 62274940 | C | G | 0.453 | -0.012 | 0.001 | 7.70E-21 | 0.00019 | 87.68 |  |
| whole-body lean mass | | rs3809569 | 66999828 | G | A | 0.243 | 0.014 | 0.001 | 2.10E-22 | 0.00021 | 94.79 |  |
| whole-body lean mass | | rs1521624 | 79412399 | A | C | 0.471 | -0.009 | 0.001 | 4.00E-13 | 0.00012 | 52.66 | BMI |
| whole-body lean mass | | rs16942324 | 89383854 | A | C | 0.027 | -0.037 | 0.004 | 2.20E-22 | 0.00021 | 94.67 |  |
| whole-body lean mass | | rs1573891 | 99186488 | C | G | 0.158 | -0.022 | 0.002 | 7.50E-38 | 0.00036 | 165.40 |  |
| whole-body lean mass | | rs35874463 | 67457698 | G | A | 0.058 | 0.018 | 0.003 | 9.70E-12 | 0.00010 | 46.39 |  |
| whole-body lean mass | | rs5742915 | 74336633 | C | T | 0.460 | 0.009 | 0.001 | 3.20E-14 | 0.00013 | 57.63 | BMI |
| whole-body lean mass | | rs12907384 | 86276000 | C | T | 0.532 | -0.012 | 0.001 | 4.70E-22 | 0.00020 | 93.20 |  |
| whole-body lean mass | | rs11648796 | 792190 | G | A | 0.232 | 0.015 | 0.002 | 1.40E-23 | 0.00022 | 100.11 |  |
| whole-body lean mass | | rs71385734 | 2160503 | G | T | 0.170 | -0.023 | 0.002 | 1.20E-43 | 0.00042 | 191.94 | BMI |
| whole-body lean mass | | rs876672 | 81571600 | G | A | 0.085 | -0.013 | 0.002 | 3.20E-09 | 0.00008 | 35.05 |  |
| whole-body lean mass | | rs7188009 | 81660642 | A | G | 0.404 | 0.009 | 0.001 | 7.00E-12 | 0.00010 | 47.03 |  |
| whole-body lean mass | | rs2531991 | 4023553 | A | G | 0.747 | 0.016 | 0.001 | 2.30E-30 | 0.00029 | 131.11 | BMI |
| whole-body lean mass | | rs55727637 | 15145252 | T | C | 0.305 | -0.012 | 0.001 | 6.10E-20 | 0.00018 | 83.60 | BMI |
| whole-body lean mass | | rs2726036 | 28347140 | C | A | 0.398 | 0.012 | 0.001 | 8.00E-21 | 0.00019 | 87.61 | BMI |
| whole-body lean mass | | rs72801843 | 53508802 | A | T | 0.301 | 0.016 | 0.001 | 3.90E-33 | 0.00032 | 143.83 |  |
| whole-body lean mass | | rs71393968 | 67847341 | A | G | 0.046 | 0.021 | 0.003 | 6.90E-13 | 0.00011 | 51.58 |  |
| whole-body lean mass | | rs8059189 | 86417349 | A | G | 0.404 | -0.012 | 0.001 | 4.40E-20 | 0.00019 | 84.24 |  |
| whole-body lean mass | | rs7192870 | 14397436 | C | T | 0.469 | -0.009 | 0.001 | 2.00E-12 | 0.00011 | 49.44 |  |
| whole-body lean mass | | rs56094641 | 53806453 | G | A | 0.405 | 0.036 | 0.001 | 1.00E-178 | 0.00178 | 812.49 | BMI |
| whole-body lean mass | | rs34017457 | 67166731 | A | G | 0.007 | 0.049 | 0.007 | 6.90E-11 | 0.00009 | 42.55 |  |
| whole-body lean mass | | rs76513770 | 72505534 | C | T | 0.128 | -0.016 | 0.002 | 5.10E-18 | 0.00016 | 74.83 |  |
| whole-body lean mass | | rs4887925 | 73784416 | C | G | 0.413 | -0.008 | 0.001 | 1.20E-09 | 0.00008 | 36.89 |  |
| whole-body lean mass | | rs9925273 | 375900 | G | A | 0.181 | -0.012 | 0.002 | 2.70E-13 | 0.00012 | 53.39 |  |
| whole-body lean mass | | rs9938120 | 19977733 | T | C | 0.144 | -0.018 | 0.002 | 9.40E-25 | 0.00023 | 105.53 | BMI |
| whole-body lean mass | | rs4788218 | 30055750 | C | T | 0.401 | 0.022 | 0.001 | 3.60E-66 | 0.00065 | 295.21 | BMI |
| whole-body lean mass | | rs6500249 | 49778147 | G | A | 0.735 | -0.008 | 0.001 | 6.40E-09 | 0.00007 | 33.71 |  |
| whole-body lean mass | | rs10775348 | 88806348 | G | A | 0.704 | 0.013 | 0.001 | 2.00E-21 | 0.00020 | 90.38 |  |
| whole-body lean mass | | rs25849 | 2265071 | G | C | 0.289 | 0.017 | 0.001 | 1.30E-34 | 0.00033 | 150.51 |  |
| whole-body lean mass | | rs3751866 | 84771304 | C | T | 0.247 | -0.008 | 0.001 | 1.90E-08 | 0.00007 | 31.64 |  |
| whole-body lean mass | | rs10775406 | 46197755 | G | A | 0.760 | -0.010 | 0.001 | 4.70E-11 | 0.00010 | 43.29 |  |
| whole-body lean mass | | rs7503332 | 63563513 | G | T | 0.482 | 0.008 | 0.001 | 9.40E-10 | 0.00008 | 37.45 |  |
| whole-body lean mass | | rs2252909 | 2278609 | T | C | 0.657 | -0.007 | 0.001 | 2.10E-08 | 0.00007 | 31.40 |  |
| whole-body lean mass | | rs2521349 | 67503501 | A | G | 0.385 | 0.008 | 0.001 | 1.60E-09 | 0.00008 | 36.42 |  |
| whole-body lean mass | | rs114177791 | 78555512 | T | G | 0.214 | 0.010 | 0.002 | 2.70E-11 | 0.00010 | 44.41 | BMI |
| whole-body lean mass | | rs36000545 | 79093822 | G | A | 0.396 | -0.017 | 0.001 | 8.20E-38 | 0.00036 | 165.23 |  |
| whole-body lean mass | | rs55831773 | 7559037 | T | C | 0.199 | -0.015 | 0.002 | 4.10E-20 | 0.00019 | 84.37 |  |
| whole-body lean mass | | rs78378222 | 7571752 | G | T | 0.012 | 0.082 | 0.006 | 7.40E-46 | 0.00044 | 202.05 |  |
| whole-body lean mass | | rs4795318 | 36949053 | T | C | 0.520 | 0.009 | 0.001 | 7.10E-13 | 0.00011 | 51.51 |  |
| whole-body lean mass | | rs6416914 | 39262850 | G | A | 0.695 | -0.009 | 0.001 | 6.50E-11 | 0.00009 | 42.67 | BMI |
| whole-body lean mass | | rs67560975 | 47142930 | C | T | 0.116 | 0.013 | 0.002 | 3.30E-11 | 0.00010 | 43.97 | BMI |
| whole-body lean mass | | rs77093479 | 79429575 | G | C | 0.165 | -0.010 | 0.002 | 6.90E-10 | 0.00008 | 38.05 |  |
| whole-body lean mass | | rs9898189 | 80480516 | G | C | 0.644 | -0.008 | 0.001 | 2.10E-09 | 0.00008 | 35.92 |  |
| whole-body lean mass | | rs3110496 | 27917771 | G | A | 0.686 | 0.009 | 0.001 | 5.00E-11 | 0.00009 | 43.19 |  |
| whole-body lean mass | | rs7223535 | 29211667 | A | G | 0.270 | -0.025 | 0.001 | 1.00E-71 | 0.00070 | 320.72 |  |
| whole-body lean mass | | rs2015561 | 38593294 | G | A | 0.424 | -0.012 | 0.001 | 1.90E-20 | 0.00019 | 85.93 |  |
| whole-body lean mass | | rs8067974 | 43232566 | G | A | 0.329 | -0.014 | 0.001 | 1.90E-25 | 0.00024 | 108.65 |  |
| whole-body lean mass | | rs34055910 | 64460104 | G | A | 0.379 | -0.008 | 0.001 | 5.10E-09 | 0.00008 | 34.16 |  |
| whole-body lean mass | | rs4968799 | 68158831 | T | A | 0.112 | -0.018 | 0.002 | 7.30E-21 | 0.00019 | 87.78 |  |
| whole-body lean mass | | rs4525525 | 1866892 | T | G | 0.264 | -0.009 | 0.001 | 5.90E-11 | 0.00009 | 42.86 | BMI |
| whole-body lean mass | | rs8074074 | 17407816 | T | C | 0.755 | 0.009 | 0.001 | 6.80E-11 | 0.00009 | 42.59 |  |
| whole-body lean mass | | rs9892365 | 59491384 | G | A | 0.670 | -0.016 | 0.001 | 8.90E-33 | 0.00031 | 142.18 |  |
| whole-body lean mass | | rs2005172 | 61996255 | C | A | 0.640 | 0.023 | 0.001 | 1.00E-66 | 0.00065 | 297.69 | body fat percentage |
| whole-body lean mass | | rs236587 | 68203915 | C | T | 0.737 | -0.008 | 0.001 | 6.20E-09 | 0.00007 | 33.77 |  |
| whole-body lean mass | | rs498685 | 13088673 | C | T | 0.594 | -0.007 | 0.001 | 2.20E-08 | 0.00007 | 31.31 |  |
| whole-body lean mass | | rs8091374 | 71904385 | A | G | 0.161 | -0.010 | 0.002 | 1.10E-08 | 0.00007 | 32.73 | BMI |
| whole-body lean mass | | rs4800670 | 23415263 | C | G | 0.386 | -0.007 | 0.001 | 2.30E-08 | 0.00007 | 31.26 |  |
| whole-body lean mass | | rs1941697 | 31251276 | A | G | 0.449 | 0.008 | 0.001 | 1.00E-09 | 0.00008 | 37.31 | BMI |
| whole-body lean mass | | rs33973388 | 46611842 | T | G | 0.435 | 0.012 | 0.001 | 9.70E-23 | 0.00021 | 96.34 |  |
| whole-body lean mass | | rs9960619 | 33040095 | T | C | 0.344 | 0.010 | 0.001 | 8.70E-14 | 0.00012 | 55.65 |  |
| whole-body lean mass | | rs9951619 | 56882326 | G | T | 0.767 | 0.012 | 0.001 | 4.00E-17 | 0.00016 | 70.78 | BMI |
| whole-body lean mass | | rs12967135 | 57849023 | A | G | 0.233 | 0.043 | 0.001 | 2.30E-190 | 0.00190 | 866.08 | BMI |
| whole-body lean mass | | rs8088739 | 60767088 | C | T | 0.244 | 0.008 | 0.001 | 1.30E-08 | 0.00007 | 32.29 |  |
| whole-body lean mass | | rs74494415 | 74972138 | T | C | 0.040 | -0.025 | 0.003 | 4.60E-15 | 0.00014 | 61.43 |  |
| whole-body lean mass | | rs11663903 | 32762592 | A | G | 0.429 | 0.008 | 0.001 | 2.00E-09 | 0.00008 | 35.99 |  |
| whole-body lean mass | | rs7229520 | 46516468 | A | G | 0.662 | -0.010 | 0.001 | 2.00E-15 | 0.00014 | 63.02 |  |
| whole-body lean mass | | rs57126421 | 2656989 | G | A | 0.238 | -0.011 | 0.001 | 3.80E-13 | 0.00012 | 52.77 |  |
| whole-body lean mass | | rs4369779 | 20735408 | C | T | 0.789 | 0.026 | 0.002 | 2.60E-65 | 0.00064 | 291.33 |  |
| whole-body lean mass | | rs55854145 | 45928049 | C | A | 0.055 | -0.015 | 0.003 | 2.40E-08 | 0.00007 | 31.13 |  |
| whole-body lean mass | | rs17066856 | 58049656 | C | T | 0.092 | -0.021 | 0.002 | 5.60E-22 | 0.00020 | 92.87 | BMI |
| whole-body lean mass | | rs2602713 | 4910021 | C | A | 0.440 | 0.011 | 0.001 | 1.20E-18 | 0.00017 | 77.64 |  |
| whole-body lean mass | | rs284662 | 41932275 | C | T | 0.620 | -0.009 | 0.001 | 2.20E-13 | 0.00012 | 53.83 |  |
| whole-body lean mass | | rs12981554 | 3416668 | G | A | 0.542 | -0.009 | 0.001 | 8.90E-12 | 0.00010 | 46.56 |  |
| whole-body lean mass | | rs7245985 | 30710410 | G | T | 0.208 | -0.011 | 0.002 | 1.50E-12 | 0.00011 | 50.10 |  |
| whole-body lean mass | | rs29946 | 34299865 | C | T | 0.557 | 0.007 | 0.001 | 4.50E-09 | 0.00008 | 34.39 |  |
| whole-body lean mass | | rs281385 | 49217261 | G | A | 0.879 | -0.012 | 0.002 | 1.70E-10 | 0.00009 | 40.79 |  |
| whole-body lean mass | | rs147110934 | 55993436 | T | G | 0.024 | -0.032 | 0.004 | 5.60E-15 | 0.00013 | 61.03 |  |
| whole-body lean mass | | rs10432304 | 4458242 | T | A | 0.560 | 0.007 | 0.001 | 4.80E-09 | 0.00008 | 34.26 |  |
| whole-body lean mass | | rs58857770 | 7208110 | G | C | 0.408 | -0.010 | 0.001 | 7.60E-14 | 0.00012 | 55.90 |  |
| whole-body lean mass | | rs34831515 | 17275777 | T | C | 0.232 | -0.008 | 0.001 | 1.30E-08 | 0.00007 | 32.34 |  |
| whole-body lean mass | | rs111640872 | 30290357 | C | G | 0.331 | 0.014 | 0.001 | 1.10E-27 | 0.00026 | 118.90 | BMI |
| whole-body lean mass | | rs3810291 | 47569003 | A | G | 0.675 | 0.017 | 0.001 | 2.40E-37 | 0.00036 | 163.08 | BMI |
| whole-body lean mass | | rs11880992 | 2176403 | A | G | 0.408 | 0.014 | 0.001 | 4.60E-28 | 0.00027 | 120.61 |  |
| whole-body lean mass | | rs62621197 | 8670147 | T | C | 0.037 | -0.033 | 0.003 | 1.40E-21 | 0.00020 | 91.01 | body fat percentage |
| whole-body lean mass | | rs73004967 | 19717056 | G | A | 0.069 | -0.017 | 0.002 | 1.30E-11 | 0.00010 | 45.79 |  |
| whole-body lean mass | | rs35050648 | 46991243 | T | G | 0.236 | 0.008 | 0.001 | 1.60E-08 | 0.00007 | 31.98 |  |
| whole-body lean mass | | rs2163832 | 10745764 | C | T | 0.660 | -0.013 | 0.001 | 6.40E-24 | 0.00022 | 101.72 |  |
| whole-body lean mass | | rs1407031 | 6542634 | T | C | 0.397 | -0.008 | 0.001 | 1.20E-10 | 0.00009 | 41.42 |  |
| whole-body lean mass | | rs6514066 | 44500794 | G | T | 0.624 | 0.007 | 0.001 | 2.50E-08 | 0.00007 | 31.07 |  |
| whole-body lean mass | | rs6026578 | 57463472 | G | C | 0.625 | -0.010 | 0.001 | 6.80E-14 | 0.00012 | 56.11 |  |
| whole-body lean mass | | rs2427320 | 60976723 | G | A | 0.232 | -0.008 | 0.001 | 2.50E-08 | 0.00007 | 31.10 |  |
| whole-body lean mass | | rs116165844 | 62610556 | T | G | 0.137 | -0.011 | 0.002 | 4.30E-09 | 0.00008 | 34.47 |  |
| whole-body lean mass | | rs6085658 | 6685377 | T | C | 0.403 | -0.009 | 0.001 | 1.60E-11 | 0.00010 | 45.35 |  |
| whole-body lean mass | | rs143384 | 34025756 | G | A | 0.404 | 0.035 | 0.001 | 1.50E-171 | 0.00171 | 779.56 | waist hip ratio |
| whole-body lean mass | | rs34879158 | 32300634 | C | A | 0.263 | -0.022 | 0.001 | 2.20E-52 | 0.00051 | 231.98 |  |
| whole-body lean mass | | rs237738 | 47904920 | C | T | 0.823 | 0.009 | 0.002 | 7.30E-09 | 0.00007 | 33.46 |  |
| whole-body lean mass | | rs6081869 | 20060745 | G | T | 0.386 | -0.010 | 0.001 | 4.40E-15 | 0.00014 | 61.50 |  |
| whole-body lean mass | | rs2252720 | 21223663 | T | C | 0.674 | -0.014 | 0.001 | 4.60E-24 | 0.00023 | 102.39 |  |
| whole-body lean mass | | rs6142059 | 32544327 | C | T | 0.493 | 0.010 | 0.001 | 1.10E-14 | 0.00013 | 59.76 | BMI |
| whole-body lean mass | | rs13043303 | 51091385 | A | G | 0.183 | -0.019 | 0.002 | 6.20E-31 | 0.00029 | 133.73 | BMI |
| whole-body lean mass | | rs73619441 | 61564901 | G | T | 0.144 | -0.013 | 0.002 | 1.50E-12 | 0.00011 | 50.07 | BMI |
| whole-body lean mass | | rs1780672 | 35755818 | C | T | 0.811 | 0.013 | 0.002 | 2.20E-15 | 0.00014 | 62.85 |  |
| whole-body lean mass | | rs4819021 | 46466927 | C | T | 0.477 | -0.008 | 0.001 | 6.40E-10 | 0.00008 | 38.20 | BMI |
| whole-body lean mass | | rs9976812 | 39690245 | G | C | 0.564 | -0.014 | 0.001 | 6.10E-27 | 0.00025 | 115.50 |  |
| whole-body lean mass | | rs7280982 | 37468223 | A | G | 0.221 | -0.009 | 0.002 | 4.20E-09 | 0.00008 | 34.52 |  |
| whole-body lean mass | | rs1043801 | 47552527 | A | G | 0.058 | 0.015 | 0.003 | 1.80E-08 | 0.00007 | 31.70 |  |
| whole-body lean mass | | rs73189390 | 17383170 | A | G | 0.184 | -0.009 | 0.002 | 2.30E-08 | 0.00007 | 31.26 |  |
| whole-body lean mass | | rs35665085 | 17625915 | A | G | 0.056 | -0.017 | 0.003 | 9.20E-10 | 0.00008 | 37.48 |  |
| whole-body lean mass | | rs4821940 | 40659573 | C | T | 0.550 | -0.007 | 0.001 | 2.50E-08 | 0.00007 | 31.08 | BMI |
| whole-body lean mass | | rs41311445 | 42070374 | C | A | 0.096 | -0.024 | 0.002 | 1.60E-30 | 0.00029 | 131.92 |  |
| whole-body lean mass | | rs5771118 | 50714289 | C | T | 0.742 | 0.009 | 0.001 | 1.80E-09 | 0.00008 | 36.22 |  |
| whole-body lean mass | | rs165656 | 19948863 | C | G | 0.517 | 0.008 | 0.001 | 8.10E-11 | 0.00009 | 42.23 |  |
| whole-body lean mass | | rs5753630 | 31861950 | G | A | 0.439 | 0.007 | 0.001 | 6.70E-09 | 0.00007 | 33.63 |  |
| whole-body lean mass | | rs118173451 | 28356600 | C | T | 0.016 | -0.029 | 0.005 | 6.30E-09 | 0.00007 | 33.74 |  |
| whole-body lean mass | | rs113619763 | 46399175 | T | A | 0.062 | 0.017 | 0.003 | 8.00E-11 | 0.00009 | 42.26 |  |
| whole-body lean mass | | rs5752989 | 30365780 | A | G | 0.571 | -0.010 | 0.001 | 2.90E-15 | 0.00014 | 62.32 |  |
|  |

Abbreviation: SNP, single nucleotide polymorphism; EA, Effect allele; NEA, Non-effect allele; EAF, effect allele frequency; SE, standard error; BMI, body mass index.

*^a^ R^2^* was calculated using the following formula: (2×EAF×(1-EAF)×beta^2^)/[(2×EAF×(1-EAF)×beta^2^)+(2×EAF×(1-EAF)×N×SE^2^)], where EAF is the effect allele

frequency, beta is the estimated effect on urate. Ν is the sample size of the GWAS for the SNP-urate association and SE is the standard error of the estimated effect.

*^b^ F* statistic was calculated using the following formula: *R^2^*(N-2)/(1-*R^2^*), where *R^2^* is the proportion of variance in urate explained by each instrument and N is the sample size of the GWAS for the SNP-urate association.

^c^ SNPs associated with confounding factors were removed after searching Phenoscanner database.

Supplementary Table 7 Characteristics of SNPs used as genetic instruments for right-leg FFM

| Exposure | SNP | | Position | EA | NEA | EAF | SNP-Exposure association | | | R^2 a^ | F-statistic ^b^ | Confounders ^c^ |
| --- | --- | --- | --- | --- | --- | --- | --- | --- | --- | --- | --- | --- |
|  |  | |  |  |  |  | Beta | SE | P value |  |  |  |
| Left-leg FFM | rs1356803 | | 32131163 | G | C | 0.546 | 0.009 | 0.001 | 1.70E-11 | 1.0E-04 | 45.26 |  |
| Left-leg FFM | rs4653016 | | 33776441 | A | C | 0.683 | 0.014 | 0.001 | 5.60E-23 | 2.1E-04 | 97.41 | BMI |
| Left-leg FFM | rs2298200 | | 49361400 | T | C | 0.183 | 0.010 | 0.002 | 7.80E-10 | 8.3E-05 | 37.80 | BMI |
| Left-leg FFM | rs2568958 | | 72765116 | A | G | 0.604 | 0.011 | 0.001 | 1.10E-15 | 1.4E-04 | 64.31 | BMI |
| Left-leg FFM | rs3845344 | | 75001480 | T | C | 0.391 | 0.010 | 0.001 | 2.20E-13 | 1.2E-04 | 53.81 | BMI |
| Left-leg FFM | rs34517439 | | 78450517 | A | C | 0.122 | 0.038 | 0.002 | 1.60E-80 | 7.9E-04 | 361.12 | BMI |
| Left-leg FFM | rs2181375 | | 96940119 | G | A | 0.596 | 0.010 | 0.001 | 1.10E-13 | 1.2E-04 | 55.23 | BMI |
| Left-leg FFM | rs655598 | | 190287713 | A | G | 0.563 | -0.011 | 0.001 | 2.10E-16 | 1.5E-04 | 67.50 | BMI |
| Left-leg FFM | rs2807339 | | 22578063 | C | T | 0.758 | 0.009 | 0.002 | 6.20E-09 | 7.4E-05 | 33.77 |  |
| Left-leg FFM | rs11578046 | | 23425139 | A | G | 0.327 | -0.012 | 0.001 | 1.10E-18 | 1.7E-04 | 77.92 |  |
| Left-leg FFM | rs6669139 | | 56583110 | T | C | 0.209 | 0.016 | 0.002 | 2.40E-23 | 2.2E-04 | 99.08 |  |
| Left-leg FFM | rs10923769 | | 119734551 | G | C | 0.145 | 0.010 | 0.002 | 4.80E-08 | 6.5E-05 | 29.78 |  |
| Left-leg FFM | rs112685832 | | 155822629 | A | C | 0.115 | 0.015 | 0.002 | 1.10E-12 | 1.1E-04 | 50.69 | BMI |
| Left-leg FFM | rs7546249 | | 176792690 | A | T | 0.727 | 0.013 | 0.001 | 6.00E-19 | 1.7E-04 | 79.08 |  |
| Left-leg FFM | rs2678204 | | 201800511 | G | T | 0.340 | 0.013 | 0.001 | 5.40E-21 | 1.9E-04 | 88.37 | BMI |
| Left-leg FFM | rs35492502 | | 217806224 | A | G | 0.296 | 0.010 | 0.001 | 1.70E-12 | 1.1E-04 | 49.75 |  |
| Left-leg FFM | rs2281175 | | 1665702 | C | T | 0.405 | 0.011 | 0.001 | 6.50E-15 | 1.3E-04 | 60.73 | BMI |
| Left-leg FFM | rs212521 | | 21580345 | C | T | 0.601 | 0.007 | 0.001 | 4.90E-08 | 6.5E-05 | 29.74 |  |
| Left-leg FFM | rs17363646 | | 86823503 | G | A | 0.136 | 0.012 | 0.002 | 1.70E-10 | 9.0E-05 | 40.77 |  |
| Left-leg FFM | rs72697614 | | 107514107 | A | C | 0.320 | 0.008 | 0.001 | 3.50E-09 | 7.7E-05 | 34.91 | BMI |
| Left-leg FFM | rs543874 | | 177889480 | G | A | 0.205 | 0.030 | 0.002 | 1.40E-75 | 7.4E-04 | 338.48 | BMI |
| Left-leg FFM | rs55800172 | | 32414767 | A | G | 0.064 | 0.018 | 0.003 | 3.40E-11 | 9.7E-05 | 43.90 |  |
| Left-leg FFM | rs28605759 | | 38399816 | A | G | 0.454 | -0.009 | 0.001 | 3.90E-11 | 9.6E-05 | 43.67 |  |
| Left-leg FFM | rs17491275 | | 39672545 | G | T | 0.160 | 0.019 | 0.002 | 6.70E-27 | 2.5E-04 | 115.31 | BMI |
| Left-leg FFM | rs12140153 | | 62579891 | T | G | 0.094 | -0.020 | 0.002 | 9.40E-18 | 1.6E-04 | 73.64 | BMI |
| Left-leg FFM | rs3009872 | | 66411400 | C | T | 0.435 | -0.007 | 0.001 | 2.10E-08 | 6.9E-05 | 31.41 | BMI |
| Left-leg FFM | rs7550987 | | 96338074 | T | G | 0.385 | 0.008 | 0.001 | 3.20E-10 | 8.7E-05 | 39.54 | BMI |
| Left-leg FFM | rs76798800 | | 154994978 | T | G | 0.266 | 0.023 | 0.001 | 5.80E-54 | 5.3E-04 | 239.21 | BMI |
| Left-leg FFM | rs12731187 | | 202013757 | T | C | 0.358 | -0.008 | 0.001 | 4.30E-09 | 7.6E-05 | 34.49 | BMI |
| Left-leg FFM | rs6675441 | | 214659762 | A | G | 0.234 | -0.012 | 0.002 | 1.40E-13 | 1.2E-04 | 54.74 |  |
| Left-leg FFM | rs2615075 | | 225934295 | G | A | 0.376 | 0.010 | 0.001 | 1.60E-13 | 1.2E-04 | 54.45 |  |
| Left-leg FFM | rs2789370 | | 235503256 | G | A | 0.346 | -0.009 | 0.001 | 1.40E-11 | 1.0E-04 | 45.69 |  |
| Left-leg FFM | rs149229890 | | 2253864 | T | G | 0.011 | 0.039 | 0.006 | 7.50E-10 | 8.3E-05 | 37.88 |  |
| Left-leg FFM | rs67373773 | | 2710377 | A | G | 0.439 | -0.008 | 0.001 | 2.80E-09 | 7.8E-05 | 35.31 | BMI |
| Left-leg FFM | rs1205593 | | 11252716 | C | T | 0.759 | -0.015 | 0.002 | 1.20E-23 | 2.2E-04 | 100.55 | BMI |
| Left-leg FFM | rs66766977 | | 22431092 | C | T | 0.539 | -0.010 | 0.001 | 1.90E-14 | 1.3E-04 | 58.67 |  |
| Left-leg FFM | rs475390 | | 41570459 | A | G | 0.776 | -0.018 | 0.002 | 3.20E-30 | 2.9E-04 | 130.49 |  |
| Left-leg FFM | rs12095997 | | 51391845 | T | C | 0.089 | 0.020 | 0.002 | 2.50E-19 | 1.8E-04 | 80.77 |  |
| Left-leg FFM | rs77848106 | | 107971673 | A | C | 0.296 | -0.010 | 0.001 | 5.10E-12 | 1.0E-04 | 47.65 | BMI |
| Left-leg FFM | rs17024393 | | 110154688 | C | T | 0.026 | 0.037 | 0.004 | 2.50E-19 | 1.8E-04 | 80.80 | BMI |
| Left-leg FFM | rs11205354 | | 150249101 | A | C | 0.444 | -0.009 | 0.001 | 4.40E-11 | 9.5E-05 | 43.42 |  |
| Left-leg FFM | rs60077625 | | 172098794 | A | G | 0.314 | 0.012 | 0.001 | 2.60E-17 | 1.6E-04 | 71.65 |  |
| Left-leg FFM | rs12123505 | | 203791049 | G | C | 0.719 | -0.008 | 0.001 | 6.70E-09 | 7.4E-05 | 33.63 |  |
| Left-leg FFM | rs823118 | | 205723572 | T | C | 0.548 | -0.013 | 0.001 | 3.80E-24 | 2.3E-04 | 102.77 | BMI |
| Left-leg FFM | rs6684205 | | 218609702 | G | A | 0.287 | 0.014 | 0.001 | 5.90E-23 | 2.1E-04 | 97.31 |  |
| Left-leg FFM | rs13007086 | | 630034 | T | A | 0.828 | 0.037 | 0.002 | 1.20E-103 | 1.0E-03 | 467.38 | BMI |
| Left-leg FFM | rs11695471 | | 25457708 | A | T | 0.331 | -0.010 | 0.001 | 2.50E-12 | 1.1E-04 | 49.03 |  |
| Left-leg FFM | rs2075171 | | 58688907 | A | G | 0.238 | -0.010 | 0.002 | 3.10E-10 | 8.7E-05 | 39.63 | BMI |
| Left-leg FFM | rs6711568 | | 59291172 | G | T | 0.702 | -0.012 | 0.001 | 1.40E-17 | 1.6E-04 | 72.86 | BMI |
| Left-leg FFM | rs752070 | | 74824970 | G | A | 0.126 | 0.012 | 0.002 | 6.90E-10 | 8.4E-05 | 38.04 |  |
| Left-leg FFM | rs1837367 | | 111874551 | A | G | 0.483 | 0.010 | 0.001 | 2.00E-13 | 1.2E-04 | 54.02 |  |
| Left-leg FFM | rs1064213 | | 198950240 | A | G | 0.478 | 0.010 | 0.001 | 6.30E-16 | 1.4E-04 | 65.33 | BMI |
| Left-leg FFM | rs1542224 | | 223963874 | C | T | 0.719 | 0.014 | 0.001 | 4.60E-21 | 1.9E-04 | 88.70 |  |
| Left-leg FFM | rs3116201 | | 233074205 | A | G | 0.098 | -0.015 | 0.002 | 5.30E-12 | 1.0E-04 | 47.57 |  |
| Left-leg FFM | rs2197563 | | 233687080 | A | G | 0.595 | 0.010 | 0.001 | 1.40E-13 | 1.2E-04 | 54.70 |  |
| Left-leg FFM | rs12713004 | | 23896049 | G | A | 0.725 | 0.014 | 0.001 | 3.00E-21 | 2.0E-04 | 89.53 |  |
| Left-leg FFM | rs10202845 | | 42575820 | G | A | 0.113 | -0.016 | 0.002 | 1.70E-14 | 1.3E-04 | 58.88 |  |
| Left-leg FFM | rs10188231 | | 142297493 | G | C | 0.186 | -0.011 | 0.002 | 3.10E-11 | 9.7E-05 | 44.13 | BMI |
| Left-leg FFM | rs77165542 | | 430975 | T | C | 0.035 | -0.067 | 0.004 | 7.40E-79 | 7.8E-04 | 353.47 | BMI |
| Left-leg FFM | rs1374370 | | 85818273 | A | G | 0.305 | 0.009 | 0.001 | 2.40E-10 | 8.8E-05 | 40.09 |  |
| Left-leg FFM | rs11680549 | | 88926348 | C | G | 0.280 | 0.009 | 0.001 | 4.00E-09 | 7.6E-05 | 34.61 |  |
| Left-leg FFM | rs6430010 | | 143972554 | A | T | 0.142 | 0.013 | 0.002 | 6.30E-13 | 1.1E-04 | 51.75 | BMI |
| Left-leg FFM | rs2140046 | | 169706079 | C | T | 0.364 | -0.010 | 0.001 | 3.90E-13 | 1.2E-04 | 52.71 |  |
| Left-leg FFM | rs72885917 | | 172416376 | C | A | 0.247 | -0.020 | 0.002 | 1.00E-40 | 3.9E-04 | 178.56 |  |
| Left-leg FFM | rs6745626 | | 181436641 | T | C | 0.590 | 0.008 | 0.001 | 6.80E-09 | 7.4E-05 | 33.59 | BMI |
| Left-leg FFM | rs10803955 | | 183228114 | G | A | 0.508 | -0.009 | 0.001 | 1.80E-12 | 1.1E-04 | 49.66 |  |
| Left-leg FFM | rs1260326 | | 27730940 | C | T | 0.604 | 0.017 | 0.001 | 2.00E-36 | 3.5E-04 | 158.83 |  |
| Left-leg FFM | rs115179432 | | 33348679 | G | A | 0.072 | -0.018 | 0.003 | 3.00E-13 | 1.2E-04 | 53.20 |  |
| Left-leg FFM | rs56254146 | | 100822430 | A | G | 0.136 | 0.015 | 0.002 | 5.50E-15 | 1.3E-04 | 61.08 | BMI |
| Left-leg FFM | rs1047891 | | 211540507 | A | C | 0.316 | 0.016 | 0.001 | 6.00E-30 | 2.8E-04 | 129.23 | BMI |
| Left-leg FFM | rs13430869 | | 218146818 | T | G | 0.742 | 0.012 | 0.001 | 3.30E-15 | 1.4E-04 | 62.09 |  |
| Left-leg FFM | rs17246129 | | 227259964 | A | G | 0.305 | 0.009 | 0.001 | 3.50E-10 | 8.7E-05 | 39.39 |  |
| Left-leg FFM | rs4663211 | | 236756688 | G | T | 0.766 | -0.010 | 0.002 | 2.90E-10 | 8.7E-05 | 39.73 | BMI |
| Left-leg FFM | rs9295 | | 36777825 | A | G | 0.296 | 0.013 | 0.001 | 1.70E-19 | 1.8E-04 | 81.60 |  |
| Left-leg FFM | rs59985551 | | 56106928 | T | C | 0.226 | -0.019 | 0.002 | 3.90E-34 | 3.3E-04 | 148.41 |  |
| Left-leg FFM | rs11545482 | | 70315987 | T | C | 0.020 | -0.029 | 0.005 | 2.50E-10 | 8.8E-05 | 40.00 |  |
| Left-leg FFM | rs4852777 | | 71534161 | C | G | 0.595 | -0.010 | 0.001 | 1.20E-14 | 1.3E-04 | 59.56 |  |
| Left-leg FFM | rs58584712 | | 112249583 | A | G | 0.211 | 0.011 | 0.002 | 7.10E-12 | 1.0E-04 | 47.00 |  |
| Left-leg FFM | rs17443541 | | 200402624 | C | T | 0.189 | -0.010 | 0.002 | 3.20E-09 | 7.7E-05 | 35.06 |  |
| Left-leg FFM | rs1447498 | | 205388729 | G | A | 0.770 | -0.010 | 0.002 | 7.60E-10 | 8.3E-05 | 37.86 | BMI |
| Left-leg FFM | rs1478575 | | 218278555 | A | T | 0.684 | 0.017 | 0.001 | 1.80E-33 | 3.2E-04 | 145.32 |  |
| Left-leg FFM | rs7601000 | | 242610773 | A | T | 0.767 | -0.013 | 0.002 | 3.80E-16 | 1.5E-04 | 66.34 |  |
| Left-leg FFM | rs2270894 | | 9975386 | G | C | 0.203 | -0.017 | 0.002 | 8.60E-25 | 2.3E-04 | 105.69 |  |
| Left-leg FFM | rs2569993 | | 12926096 | C | T | 0.320 | 0.009 | 0.001 | 1.80E-11 | 9.9E-05 | 45.14 | BMI |
| Left-leg FFM | rs7619139 | | 25110415 | A | T | 0.589 | 0.013 | 0.001 | 2.00E-23 | 2.2E-04 | 99.49 | BMI |
| Left-leg FFM | rs117206167 | | 33260151 | T | C | 0.066 | -0.018 | 0.003 | 4.10E-12 | 1.1E-04 | 48.08 |  |
| Left-leg FFM | rs2735559 | | 88106618 | A | G | 0.114 | -0.017 | 0.002 | 3.00E-17 | 1.6E-04 | 71.33 | BMI |
| Left-leg FFM | rs2016469 | | 108023965 | A | G | 0.372 | 0.008 | 0.001 | 5.60E-10 | 8.5E-05 | 38.47 | BMI |
| Left-leg FFM | rs7624428 | | 131516596 | A | T | 0.269 | 0.013 | 0.001 | 2.00E-17 | 1.6E-04 | 72.18 | BMI |
| Left-leg FFM | rs724016 | | 141105570 | G | A | 0.444 | 0.028 | 0.001 | 1.40E-101 | 1.0E-03 | 457.87 |  |
| Left-leg FFM | rs5396 | | 170744815 | C | T | 0.278 | 0.010 | 0.001 | 2.60E-12 | 1.1E-04 | 48.98 | BMI |
| Left-leg FFM | rs62246311 | | 9498143 | A | G | 0.102 | 0.015 | 0.002 | 1.30E-12 | 1.1E-04 | 50.39 | BMI |
| Left-leg FFM | rs9843653 | | 49920571 | C | T | 0.512 | 0.014 | 0.001 | 6.30E-28 | 2.6E-04 | 120.01 | BMI |
| Left-leg FFM | rs56038322 | | 69925128 | A | G | 0.311 | 0.008 | 0.001 | 3.40E-08 | 6.7E-05 | 30.45 | BMI |
| Left-leg FFM | rs6779752 | | 85663849 | A | G | 0.638 | -0.012 | 0.001 | 1.00E-17 | 1.6E-04 | 73.46 | BMI |
| Left-leg FFM | rs34693680 | | 98665549 | T | C | 0.132 | 0.013 | 0.002 | 1.60E-11 | 1.0E-04 | 45.37 |  |
| Left-leg FFM | rs1910466 | | 147086268 | C | T | 0.498 | -0.008 | 0.001 | 2.70E-09 | 7.8E-05 | 35.41 |  |
| Left-leg FFM | rs357486 | | 153885503 | C | T | 0.543 | 0.011 | 0.001 | 9.40E-18 | 1.6E-04 | 73.63 | BMI |
| Left-leg FFM | rs73175572 | | 185490184 | G | A | 0.112 | 0.026 | 0.002 | 6.40E-37 | 3.5E-04 | 161.12 |  |
| Left-leg FFM | rs73052033 | | 185828465 | C | T | 0.185 | -0.017 | 0.002 | 3.40E-24 | 2.3E-04 | 102.97 | BMI |
| Left-leg FFM | rs6772164 | | 196078149 | A | C | 0.358 | 0.009 | 0.001 | 7.20E-11 | 9.3E-05 | 42.46 | BMI |
| Left-leg FFM | rs1911073 | | 30051533 | G | A | 0.347 | 0.008 | 0.001 | 1.90E-09 | 7.9E-05 | 36.04 |  |
| Left-leg FFM | rs13059004 | | 38601237 | C | A | 0.478 | 0.007 | 0.001 | 2.00E-08 | 6.9E-05 | 31.47 |  |
| Left-leg FFM | rs754635 | | 42305131 | G | C | 0.887 | 0.014 | 0.002 | 1.20E-11 | 1.0E-04 | 46.05 | BMI |
| Left-leg FFM | rs6762851 | | 56686329 | C | T | 0.357 | -0.011 | 0.001 | 9.80E-16 | 1.4E-04 | 64.48 |  |
| Left-leg FFM | rs4635681 | | 152310614 | G | A | 0.156 | 0.011 | 0.002 | 1.20E-09 | 8.1E-05 | 36.96 |  |
| Left-leg FFM | rs36012032 | | 52814709 | A | C | 0.091 | 0.018 | 0.002 | 6.40E-16 | 1.4E-04 | 65.32 |  |
| Left-leg FFM | rs7641177 | | 61183158 | C | A | 0.374 | -0.009 | 0.001 | 1.10E-10 | 9.1E-05 | 41.59 | BMI |
| Left-leg FFM | rs6766472 | | 80651423 | C | A | 0.297 | 0.010 | 0.001 | 3.80E-12 | 1.1E-04 | 48.20 |  |
| Left-leg FFM | rs12631813 | | 171126134 | G | C | 0.503 | -0.008 | 0.001 | 4.10E-09 | 7.6E-05 | 34.58 |  |
| Left-leg FFM | rs7652177 | | 171969077 | G | C | 0.505 | 0.013 | 0.001 | 2.70E-23 | 2.2E-04 | 98.85 |  |
| Left-leg FFM | rs512692 | | 172170842 | T | A | 0.285 | 0.013 | 0.001 | 3.60E-20 | 1.9E-04 | 84.61 |  |
| Left-leg FFM | rs4974072 | | 41222586 | A | G | 0.490 | 0.011 | 0.001 | 1.00E-17 | 1.6E-04 | 73.42 |  |
| Left-leg FFM | rs2306272 | | 66434643 | C | T | 0.287 | 0.008 | 0.001 | 6.70E-09 | 7.4E-05 | 33.62 | BMI |
| Left-leg FFM | rs76267866 | | 70540347 | T | A | 0.206 | 0.010 | 0.002 | 1.30E-09 | 8.1E-05 | 36.82 | BMI |
| Left-leg FFM | rs6762578 | | 128992047 | A | G | 0.778 | 0.015 | 0.002 | 1.30E-20 | 1.9E-04 | 86.65 |  |
| Left-leg FFM | rs843374 | | 183997261 | T | A | 0.587 | -0.012 | 0.001 | 2.30E-20 | 1.9E-04 | 85.52 |  |
| Left-leg FFM | rs10488836 | | 2160894 | T | C | 0.053 | -0.016 | 0.003 | 4.00E-08 | 6.6E-05 | 30.14 |  |
| Left-leg FFM | rs4527444 | | 30842780 | G | A | 0.541 | 0.009 | 0.001 | 2.30E-12 | 1.1E-04 | 49.17 | BMI |
| Left-leg FFM | rs34028346 | | 54368694 | A | G | 0.142 | 0.012 | 0.002 | 4.20E-10 | 8.6E-05 | 39.04 |  |
| Left-leg FFM | rs34772064 | | 55495948 | G | T | 0.556 | -0.008 | 0.001 | 3.20E-09 | 7.7E-05 | 35.06 | BMI |
| Left-leg FFM | rs1443536 | | 82174165 | G | A | 0.305 | 0.011 | 0.001 | 2.20E-15 | 1.4E-04 | 62.84 |  |
| Left-leg FFM | rs11097755 | | 102709308 | C | T | 0.443 | 0.010 | 0.001 | 2.50E-13 | 1.2E-04 | 53.57 | BMI |
| Left-leg FFM | rs6840229 | | 115064611 | A | G | 0.438 | -0.008 | 0.001 | 2.30E-10 | 8.8E-05 | 40.16 | BMI |
| Left-leg FFM | rs4240326 | | 145839264 | G | A | 0.550 | -0.020 | 0.001 | 9.80E-55 | 5.3E-04 | 242.77 |  |
| Left-leg FFM | rs34627176 | | 1006200 | A | G | 0.214 | -0.012 | 0.002 | 8.80E-14 | 1.2E-04 | 55.63 |  |
| Left-leg FFM | rs1477890 | | 18511738 | G | A | 0.493 | 0.010 | 0.001 | 9.00E-14 | 1.2E-04 | 55.58 | BMI |
| Left-leg FFM | rs2231142 | | 89052323 | T | G | 0.113 | -0.015 | 0.002 | 6.40E-13 | 1.1E-04 | 51.73 | BMI |
| Left-leg FFM | rs34848742 | | 123828042 | G | T | 0.788 | -0.014 | 0.002 | 1.50E-18 | 1.7E-04 | 77.30 |  |
| Left-leg FFM | rs139919928 | | 134724499 | A | G | 0.027 | 0.023 | 0.004 | 1.20E-08 | 7.1E-05 | 32.52 |  |
| Left-leg FFM | rs10938398 | | 45186139 | A | G | 0.434 | 0.015 | 0.001 | 8.40E-30 | 2.8E-04 | 128.57 | BMI |
| Left-leg FFM | rs6821305 | | 122713863 | C | A | 0.399 | 0.013 | 0.001 | 5.30E-22 | 2.0E-04 | 92.98 |  |
| Left-leg FFM | rs55920177 | | 171609715 | T | A | 0.129 | -0.011 | 0.002 | 9.70E-09 | 7.2E-05 | 32.91 | BMI |
| Left-leg FFM | rs2858019 | | 3493345 | G | A | 0.275 | -0.008 | 0.001 | 9.10E-09 | 7.3E-05 | 33.03 |  |
| Left-leg FFM | rs2102278 | | 52818664 | G | A | 0.323 | 0.011 | 0.001 | 3.40E-14 | 1.3E-04 | 57.47 | BMI |
| Left-leg FFM | rs2647268 | | 106211443 | G | A | 0.384 | -0.015 | 0.001 | 2.40E-30 | 2.9E-04 | 131.04 |  |
| Left-leg FFM | rs1296328 | | 137083193 | C | A | 0.559 | -0.011 | 0.001 | 6.90E-16 | 1.4E-04 | 65.16 | BMI |
| Left-leg FFM | rs395980 | | 177430072 | G | T | 0.263 | -0.009 | 0.001 | 2.40E-10 | 8.8E-05 | 40.07 |  |
| Left-leg FFM | rs7680647 | | 1750487 | C | T | 0.356 | 0.011 | 0.001 | 3.10E-15 | 1.4E-04 | 62.21 |  |
| Left-leg FFM | rs7671110 | | 17874089 | T | C | 0.158 | -0.027 | 0.002 | 1.10E-50 | 4.9E-04 | 224.11 |  |
| Left-leg FFM | rs11945720 | | 28247163 | A | G | 0.839 | 0.010 | 0.002 | 4.70E-08 | 6.6E-05 | 29.84 |  |
| Left-leg FFM | rs17010957 | | 86719165 | C | T | 0.148 | -0.013 | 0.002 | 1.20E-11 | 1.0E-04 | 46.01 |  |
| Left-leg FFM | rs72659403 | | 90825002 | G | A | 0.078 | -0.013 | 0.002 | 3.40E-08 | 6.7E-05 | 30.46 |  |
| Left-leg FFM | rs3990738 | | 152295198 | G | A | 0.543 | -0.008 | 0.001 | 3.70E-09 | 7.6E-05 | 34.76 |  |
| Left-leg FFM | rs10058393 | | 277617 | T | C | 0.129 | 0.012 | 0.002 | 1.20E-10 | 9.1E-05 | 41.52 |  |
| Left-leg FFM | rs12519532 | | 42829303 | A | G | 0.283 | 0.013 | 0.001 | 1.70E-20 | 1.9E-04 | 86.12 |  |
| Left-leg FFM | rs2578490 | | 92389236 | G | A | 0.642 | 0.008 | 0.001 | 7.00E-10 | 8.4E-05 | 38.03 | BMI |
| Left-leg FFM | rs62365719 | | 95862694 | C | T | 0.398 | -0.009 | 0.001 | 1.70E-11 | 1.0E-04 | 45.30 | BMI |
| Left-leg FFM | rs2952615 | | 112138888 | C | G | 0.618 | -0.011 | 0.001 | 1.20E-17 | 1.6E-04 | 73.15 |  |
| Left-leg FFM | rs62378779 | | 122649290 | T | C | 0.300 | -0.012 | 0.001 | 1.30E-15 | 1.4E-04 | 63.98 |  |
| Left-leg FFM | rs153758 | | 171209065 | C | G | 0.431 | -0.007 | 0.001 | 1.30E-08 | 7.1E-05 | 32.28 |  |
| Left-leg FFM | rs252937 | | 5497900 | G | C | 0.662 | -0.010 | 0.001 | 2.70E-12 | 1.1E-04 | 48.87 |  |
| Left-leg FFM | rs695922 | | 53385529 | G | A | 0.837 | -0.011 | 0.002 | 2.20E-09 | 7.9E-05 | 35.79 |  |
| Left-leg FFM | rs505575 | | 111256812 | C | T | 0.673 | -0.009 | 0.001 | 1.60E-11 | 1.0E-04 | 45.37 |  |
| Left-leg FFM | rs1835009 | | 122661217 | T | C | 0.236 | -0.015 | 0.002 | 1.10E-23 | 2.2E-04 | 100.61 | BMI |
| Left-leg FFM | rs9327336 | | 123990270 | C | T | 0.343 | 0.008 | 0.001 | 1.90E-08 | 7.0E-05 | 31.62 |  |
| Left-leg FFM | rs6860245 | | 127367998 | C | G | 0.248 | 0.025 | 0.002 | 1.10E-59 | 5.8E-04 | 265.49 |  |
| Left-leg FFM | rs157577 | | 131563571 | C | G | 0.722 | 0.015 | 0.001 | 2.20E-25 | 2.4E-04 | 108.37 |  |
| Left-leg FFM | rs7701443 | | 142792650 | G | A | 0.411 | -0.008 | 0.001 | 1.20E-08 | 7.1E-05 | 32.43 |  |
| Left-leg FFM | rs4282339 | | 168256240 | A | G | 0.208 | -0.016 | 0.002 | 1.10E-23 | 2.2E-04 | 100.60 |  |
| Left-leg FFM | rs32736 | | 3462896 | G | T | 0.348 | 0.008 | 0.001 | 3.50E-08 | 6.7E-05 | 30.40 |  |
| Left-leg FFM | rs6450346 | | 55014771 | C | T | 0.701 | -0.009 | 0.001 | 7.80E-11 | 9.3E-05 | 42.30 |  |
| Left-leg FFM | rs6888717 | | 158363581 | A | G | 0.811 | -0.009 | 0.002 | 2.00E-08 | 6.9E-05 | 31.52 |  |
| Left-leg FFM | rs2422054 | | 161382474 | A | T | 0.631 | 0.008 | 0.001 | 4.00E-09 | 7.6E-05 | 34.61 |  |
| Left-leg FFM | rs6874142 | | 172753555 | G | T | 0.114 | 0.017 | 0.002 | 1.90E-14 | 1.3E-04 | 58.63 |  |
| Left-leg FFM | rs13180309 | | 36790001 | G | A | 0.445 | -0.014 | 0.001 | 1.50E-25 | 2.4E-04 | 109.18 |  |
| Left-leg FFM | rs840812 | | 86970418 | T | C | 0.020 | 0.027 | 0.005 | 6.90E-09 | 7.4E-05 | 33.55 | BMI |
| Left-leg FFM | rs141729694 | | 87999371 | T | C | 0.074 | 0.023 | 0.002 | 8.30E-21 | 1.9E-04 | 87.53 | BMI |
| Left-leg FFM | rs33967909 | | 137603293 | A | G | 0.216 | 0.013 | 0.002 | 6.80E-16 | 1.4E-04 | 65.19 |  |
| Left-leg FFM | rs17115481 | | 153358226 | A | G | 0.270 | -0.009 | 0.001 | 7.00E-10 | 8.4E-05 | 38.01 | BMI |
| Left-leg FFM | rs446382 | | 176517461 | G | T | 0.726 | 0.010 | 0.001 | 2.50E-12 | 1.1E-04 | 49.01 |  |
| Left-leg FFM | rs7706886 | | 32722319 | A | G | 0.271 | 0.011 | 0.001 | 2.20E-14 | 1.3E-04 | 58.39 |  |
| Left-leg FFM | rs7709645 | | 60731458 | C | G | 0.493 | -0.011 | 0.001 | 4.90E-17 | 1.5E-04 | 70.38 |  |
| Left-leg FFM | rs11738728 | | 64238731 | A | G | 0.358 | -0.008 | 0.001 | 2.60E-08 | 6.8E-05 | 31.02 |  |
| Left-leg FFM | rs6873192 | | 67598184 | G | A | 0.517 | -0.011 | 0.001 | 2.50E-16 | 1.5E-04 | 67.14 |  |
| Left-leg FFM | rs2307111 | | 75003678 | C | T | 0.395 | -0.018 | 0.001 | 3.30E-42 | 4.1E-04 | 185.36 | BMI |
| Left-leg FFM | rs365352 | | 77401152 | A | G | 0.244 | -0.016 | 0.002 | 3.90E-26 | 2.5E-04 | 111.82 | BMI |
| Left-leg FFM | rs6235 | | 95728898 | G | C | 0.268 | 0.016 | 0.001 | 2.20E-28 | 2.7E-04 | 122.06 | BMI |
| Left-leg FFM | rs3822742 | | 139059017 | A | C | 0.371 | 0.015 | 0.001 | 4.60E-27 | 2.6E-04 | 116.05 | BMI |
| Left-leg FFM | rs59738707 | | 141814420 | A | G | 0.128 | -0.011 | 0.002 | 2.70E-08 | 6.8E-05 | 30.95 |  |
| Left-leg FFM | rs4073717 | | 170864021 | T | G | 0.202 | -0.016 | 0.002 | 1.80E-22 | 2.1E-04 | 95.09 |  |
| Left-leg FFM | rs55758152 | | 171317318 | A | G | 0.326 | 0.008 | 0.001 | 2.00E-08 | 6.9E-05 | 31.45 |  |
| Left-leg FFM | rs11243202 | | 7719065 | C | T | 0.486 | 0.015 | 0.001 | 1.60E-30 | 2.9E-04 | 131.84 |  |
| Left-leg FFM | rs62396185 | | 26180634 | C | G | 0.260 | -0.022 | 0.001 | 2.00E-48 | 4.7E-04 | 213.87 | BMI |
| Left-leg FFM | rs9344126 | | 81907559 | C | T | 0.514 | -0.009 | 0.001 | 6.20E-12 | 1.0E-04 | 47.26 |  |
| Left-leg FFM | rs1369869 | | 83366282 | T | C | 0.439 | 0.009 | 0.001 | 6.40E-11 | 9.4E-05 | 42.70 | BMI |
| Left-leg FFM | rs6570509 | | 142716286 | T | G | 0.287 | -0.013 | 0.001 | 2.00E-18 | 1.7E-04 | 76.65 |  |
| Left-leg FFM | rs394487 | | 160778639 | T | C | 0.278 | 0.010 | 0.001 | 1.20E-12 | 1.1E-04 | 50.41 | BMI |
| Left-leg FFM | rs12199246 | | 41999809 | A | G | 0.087 | 0.013 | 0.002 | 4.40E-08 | 6.6E-05 | 29.98 |  |
| Left-leg FFM | rs10457469 | | 126083658 | A | G | 0.523 | 0.013 | 0.001 | 5.90E-25 | 2.3E-04 | 106.44 | BMI |
| Left-leg FFM | rs7740107 | | 130374461 | A | T | 0.736 | -0.026 | 0.001 | 3.30E-67 | 6.6E-04 | 299.99 |  |
| Left-leg FFM | rs62425398 | | 166416028 | A | C | 0.106 | 0.014 | 0.002 | 2.70E-11 | 9.8E-05 | 44.35 |  |
| Left-leg FFM | rs76307059 | | 169000888 | G | C | 0.046 | -0.018 | 0.003 | 1.30E-08 | 7.1E-05 | 32.28 |  |
| Left-leg FFM | rs12213070 | | 12131542 | A | G | 0.346 | -0.009 | 0.001 | 7.50E-12 | 1.0E-04 | 46.90 |  |
| Left-leg FFM | rs41271299 | | 19839415 | T | C | 0.051 | 0.036 | 0.003 | 8.80E-34 | 3.2E-04 | 146.76 | BMI |
| Left-leg FFM | rs16891847 | | 39260119 | C | T | 0.185 | 0.009 | 0.002 | 3.20E-08 | 6.7E-05 | 30.58 |  |
| Left-leg FFM | rs1266876 | | 51781580 | T | C | 0.350 | 0.010 | 0.001 | 7.40E-14 | 1.2E-04 | 55.97 | BMI |
| Left-leg FFM | rs3805885 | | 80996751 | C | G | 0.455 | -0.011 | 0.001 | 1.90E-17 | 1.6E-04 | 72.20 |  |
| Left-leg FFM | rs3736984 | | 90408589 | G | A | 0.172 | 0.011 | 0.002 | 2.10E-10 | 8.9E-05 | 40.40 |  |
| Left-leg FFM | rs9391253 | | 105367616 | T | A | 0.320 | 0.011 | 0.001 | 1.20E-15 | 1.4E-04 | 64.09 |  |
| Left-leg FFM | rs11756675 | | 120067932 | G | A | 0.257 | 0.008 | 0.001 | 4.80E-08 | 6.6E-05 | 29.81 |  |
| Left-leg FFM | rs687694 | | 153472227 | G | T | 0.495 | -0.008 | 0.001 | 1.20E-09 | 8.1E-05 | 37.02 |  |
| Left-leg FFM | rs4235910 | | 169344450 | C | A | 0.515 | 0.008 | 0.001 | 1.50E-09 | 8.0E-05 | 36.57 |  |
| Left-leg FFM | rs3129962 | | 32379383 | A | G | 0.129 | 0.024 | 0.002 | 1.20E-34 | 3.3E-04 | 150.68 |  |
| Left-leg FFM | rs9272554 | | 32607026 | G | A | 0.227 | 0.009 | 0.002 | 2.30E-09 | 7.9E-05 | 35.71 |  |
| Left-leg FFM | rs114056237 | | 41877671 | A | G | 0.012 | -0.063 | 0.006 | 2.40E-27 | 2.6E-04 | 117.38 |  |
| Left-leg FFM | rs1418433 | | 44752568 | A | G | 0.588 | -0.008 | 0.001 | 1.30E-09 | 8.1E-05 | 36.75 |  |
| Left-leg FFM | rs9388498 | | 126873423 | T | G | 0.186 | 0.014 | 0.002 | 5.30E-17 | 1.5E-04 | 70.21 |  |
| Left-leg FFM | rs599004 | | 140439740 | T | C | 0.281 | -0.009 | 0.001 | 2.40E-10 | 8.8E-05 | 40.08 |  |
| Left-leg FFM | rs3853252 | | 152170247 | A | G | 0.455 | 0.015 | 0.001 | 8.30E-29 | 2.7E-04 | 124.02 |  |
| Left-leg FFM | rs12216497 | | 19028623 | T | C | 0.561 | -0.010 | 0.001 | 3.00E-13 | 1.2E-04 | 53.19 |  |
| Left-leg FFM | rs3131014 | | 31116627 | A | G | 0.236 | -0.018 | 0.002 | 9.00E-31 | 2.9E-04 | 133.00 |  |
| Left-leg FFM | rs2744956 | | 34618937 | C | T | 0.139 | 0.039 | 0.002 | 6.90E-96 | 9.5E-04 | 431.71 | BMI |
| Left-leg FFM | rs4715207 | | 50809278 | T | C | 0.180 | 0.023 | 0.002 | 1.40E-42 | 4.1E-04 | 187.03 | BMI |
| Left-leg FFM | rs79589376 | | 90294244 | T | A | 0.127 | 0.011 | 0.002 | 4.70E-08 | 6.6E-05 | 29.83 |  |
| Left-leg FFM | rs17789218 | | 100600097 | C | T | 0.245 | 0.011 | 0.002 | 2.80E-12 | 1.1E-04 | 48.83 |  |
| Left-leg FFM | rs9398171 | | 108983527 | T | C | 0.711 | 0.018 | 0.001 | 2.30E-37 | 3.6E-04 | 163.18 | BMI |
| Left-leg FFM | rs11153171 | | 109653825 | T | C | 0.356 | -0.013 | 0.001 | 3.80E-21 | 2.0E-04 | 89.07 |  |
| Left-leg FFM | rs7755185 | | 152339615 | G | A | 0.311 | 0.009 | 0.001 | 1.20E-09 | 8.1E-05 | 36.92 |  |
| Left-leg FFM | rs37964 | | 8004981 | T | A | 0.466 | 0.008 | 0.001 | 5.00E-09 | 7.5E-05 | 34.20 |  |
| Left-leg FFM | rs11761240 | | 67647531 | C | T | 0.313 | -0.008 | 0.001 | 1.20E-08 | 7.1E-05 | 32.43 |  |
| Left-leg FFM | rs2866719 | | 70106061 | T | C | 0.369 | 0.009 | 0.001 | 6.90E-12 | 1.0E-04 | 47.06 | BMI |
| Left-leg FFM | rs10953083 | | 92657034 | A | C | 0.446 | 0.008 | 0.001 | 9.10E-09 | 7.3E-05 | 33.03 |  |
| Left-leg FFM | rs10256522 | | 139734373 | T | C | 0.375 | 0.007 | 0.001 | 3.00E-08 | 6.8E-05 | 30.72 |  |
| Left-leg FFM | rs34776209 | | 23513093 | T | C | 0.247 | -0.015 | 0.002 | 1.70E-23 | 2.2E-04 | 99.78 |  |
| Left-leg FFM | rs56304870 | | 44863439 | A | T | 0.070 | 0.014 | 0.003 | 1.30E-08 | 7.1E-05 | 32.30 |  |
| Left-leg FFM | rs3823674 | | 50571996 | T | C | 0.428 | -0.011 | 0.001 | 5.90E-16 | 1.4E-04 | 65.49 | BMI |
| Left-leg FFM | rs16868443 | | 93206264 | C | G | 0.360 | 0.013 | 0.001 | 2.80E-21 | 2.0E-04 | 89.69 | BMI |
| Left-leg FFM | rs2396625 | | 113028634 | A | T | 0.421 | -0.011 | 0.001 | 1.90E-15 | 1.4E-04 | 63.12 | BMI |
| Left-leg FFM | rs62621812 | | 127015083 | A | G | 0.020 | 0.041 | 0.005 | 9.10E-18 | 1.6E-04 | 73.71 | BMI |
| Left-leg FFM | rs111636812 | | 148364845 | G | T | 0.224 | -0.009 | 0.002 | 1.10E-08 | 7.2E-05 | 32.59 |  |
| Left-leg FFM | rs822549 | | 148649180 | C | T | 0.267 | 0.010 | 0.001 | 2.10E-12 | 1.1E-04 | 49.40 |  |
| Left-leg FFM | rs6946415 | | 150684548 | G | A | 0.627 | 0.018 | 0.001 | 2.00E-42 | 4.1E-04 | 186.33 | BMI |
| Left-leg FFM | rs10899768 | | 55824205 | G | A | 0.164 | -0.010 | 0.002 | 1.70E-08 | 7.0E-05 | 31.83 |  |
| Left-leg FFM | rs13244614 | | 72973854 | A | C | 0.283 | 0.010 | 0.001 | 3.10E-12 | 1.1E-04 | 48.61 |  |
| Left-leg FFM | rs4729099 | | 76638755 | A | C | 0.826 | -0.017 | 0.002 | 4.80E-24 | 2.2E-04 | 102.27 |  |
| Left-leg FFM | rs10269774 | | 92253972 | A | G | 0.326 | 0.023 | 0.001 | 1.40E-63 | 6.2E-04 | 283.32 |  |
| Left-leg FFM | rs114949263 | | 150498245 | C | T | 0.112 | -0.013 | 0.002 | 5.00E-10 | 8.5E-05 | 38.68 |  |
| Left-leg FFM | rs2533879 | | 2859847 | A | G | 0.300 | -0.022 | 0.001 | 2.00E-53 | 5.2E-04 | 236.79 |  |
| Left-leg FFM | rs520161 | | 28210660 | C | T | 0.703 | -0.009 | 0.001 | 5.70E-10 | 8.4E-05 | 38.42 |  |
| Left-leg FFM | rs12700901 | | 28783171 | A | C | 0.406 | -0.008 | 0.001 | 2.80E-09 | 7.8E-05 | 35.30 |  |
| Left-leg FFM | rs77760034 | | 46606906 | A | T | 0.075 | -0.017 | 0.003 | 7.80E-12 | 1.0E-04 | 46.81 |  |
| Left-leg FFM | rs2881198 | | 46634506 | C | G | 0.530 | -0.012 | 0.001 | 5.30E-19 | 1.7E-04 | 79.32 | BMI |
| Left-leg FFM | rs1443749 | | 121960438 | T | C | 0.366 | 0.012 | 0.001 | 4.10E-18 | 1.7E-04 | 75.26 | BMI |
| Left-leg FFM | rs836511 | | 6458319 | G | A | 0.199 | 0.011 | 0.002 | 2.40E-11 | 9.8E-05 | 44.61 |  |
| Left-leg FFM | rs2740761 | | 55255986 | T | C | 0.208 | 0.009 | 0.002 | 4.50E-08 | 6.6E-05 | 29.92 |  |
| Left-leg FFM | rs56383938 | | 74097622 | G | A | 0.087 | -0.016 | 0.002 | 8.80E-13 | 1.1E-04 | 51.10 | BMI |
| Left-leg FFM | rs1910252 | | 49407362 | T | C | 0.168 | 0.013 | 0.002 | 1.20E-13 | 1.2E-04 | 55.09 |  |
| Left-leg FFM | rs72656010 | | 57122215 | C | T | 0.132 | -0.033 | 0.002 | 6.00E-65 | 6.4E-04 | 289.63 |  |
| Left-leg FFM | rs61729527 | | 77761919 | T | C | 0.052 | -0.023 | 0.003 | 1.60E-15 | 1.4E-04 | 63.55 | BMI |
| Left-leg FFM | rs7815955 | | 130719567 | T | A | 0.203 | -0.013 | 0.002 | 7.90E-16 | 1.4E-04 | 64.89 |  |
| Left-leg FFM | rs2277138 | | 135622640 | C | T | 0.394 | -0.017 | 0.001 | 3.10E-36 | 3.5E-04 | 157.99 |  |
| Left-leg FFM | rs13264909 | | 64702385 | T | A | 0.429 | -0.009 | 0.001 | 1.30E-12 | 1.1E-04 | 50.33 | BMI |
| Left-leg FFM | rs11782103 | | 120451323 | A | T | 0.252 | -0.012 | 0.001 | 1.30E-14 | 1.3E-04 | 59.32 |  |
| Left-leg FFM | rs11777007 | | 10801857 | T | C | 0.547 | -0.011 | 0.001 | 4.50E-18 | 1.7E-04 | 75.09 | BMI |
| Left-leg FFM | rs13282247 | | 19625464 | T | C | 0.305 | -0.008 | 0.001 | 6.80E-09 | 7.4E-05 | 33.58 |  |
| Left-leg FFM | rs10958683 | | 38274193 | G | C | 0.229 | -0.011 | 0.002 | 7.40E-12 | 1.0E-04 | 46.92 |  |
| Left-leg FFM | rs4735766 | | 78099782 | T | G | 0.285 | 0.017 | 0.001 | 1.10E-32 | 3.1E-04 | 141.83 |  |
| Left-leg FFM | rs16916881 | | 95566270 | A | C | 0.235 | -0.010 | 0.002 | 2.40E-10 | 8.8E-05 | 40.10 | BMI |
| Left-leg FFM | rs112875651 | | 126506694 | A | G | 0.391 | 0.014 | 0.001 | 1.20E-26 | 2.5E-04 | 114.22 | BMI |
| Left-leg FFM | rs2280940 | | 23159320 | T | C | 0.690 | 0.009 | 0.001 | 3.20E-11 | 9.7E-05 | 44.07 |  |
| Left-leg FFM | rs62515438 | | 57161608 | G | T | 0.228 | 0.017 | 0.002 | 1.90E-28 | 2.7E-04 | 122.38 |  |
| Left-leg FFM | rs1431663 | | 73460688 | C | A | 0.572 | -0.010 | 0.001 | 4.60E-14 | 1.3E-04 | 56.89 |  |
| Left-leg FFM | rs7832926 | | 23355620 | C | A | 0.326 | 0.008 | 0.001 | 8.10E-09 | 7.3E-05 | 33.25 |  |
| Left-leg FFM | rs1982441 | | 28021769 | T | G | 0.130 | 0.012 | 0.002 | 9.90E-10 | 8.2E-05 | 37.34 | BMI |
| Left-leg FFM | rs1504797 | | 89434405 | C | T | 0.306 | -0.012 | 0.001 | 4.50E-16 | 1.5E-04 | 65.99 | BMI |
| Left-leg FFM | rs2737218 | | 116631278 | C | T | 0.208 | -0.016 | 0.002 | 1.80E-24 | 2.3E-04 | 104.23 | BMI |
| Left-leg FFM | rs117169657 | | 145508782 | T | C | 0.136 | 0.012 | 0.002 | 3.10E-10 | 8.7E-05 | 39.60 |  |
| Left-leg FFM | rs7042372 | | 6959840 | G | A | 0.335 | -0.009 | 0.001 | 6.80E-10 | 8.4E-05 | 38.08 | BMI |
| Left-leg FFM | rs11794152 | | 23345347 | G | A | 0.415 | 0.011 | 0.001 | 3.40E-16 | 1.5E-04 | 66.57 |  |
| Left-leg FFM | rs1243873 | | 35686595 | T | C | 0.462 | -0.009 | 0.001 | 2.00E-11 | 9.9E-05 | 44.93 |  |
| Left-leg FFM | rs10780905 | | 90841066 | A | G | 0.588 | -0.008 | 0.001 | 2.30E-10 | 8.8E-05 | 40.22 |  |
| Left-leg FFM | rs28457693 | | 98217348 | G | A | 0.107 | 0.025 | 0.002 | 6.10E-32 | 3.0E-04 | 138.36 |  |
| Left-leg FFM | rs11516134 | | 140282285 | C | T | 0.221 | -0.011 | 0.002 | 5.00E-12 | 1.0E-04 | 47.69 |  |
| Left-leg FFM | rs12001437 | | 34074476 | C | T | 0.368 | 0.007 | 0.001 | 3.60E-08 | 6.7E-05 | 30.37 | BMI |
| Left-leg FFM | rs1308512 | | 87279298 | G | A | 0.805 | 0.010 | 0.002 | 1.30E-09 | 8.1E-05 | 36.79 |  |
| Left-leg FFM | rs111821073 | | 99084793 | T | C | 0.157 | 0.016 | 0.002 | 5.10E-18 | 1.6E-04 | 74.86 |  |
| Left-leg FFM | rs72733810 | | 73797181 | G | A | 0.202 | 0.010 | 0.002 | 2.30E-09 | 7.8E-05 | 35.66 |  |
| Left-leg FFM | rs2482357 | | 94179978 | A | G | 0.432 | -0.010 | 0.001 | 2.30E-15 | 1.4E-04 | 62.78 | BMI |
| Left-leg FFM | rs7033487 | | 119129257 | C | T | 0.198 | -0.021 | 0.002 | 2.10E-38 | 3.7E-04 | 167.97 |  |
| Left-leg FFM | rs35990522 | | 119274481 | A | T | 0.075 | 0.016 | 0.002 | 1.30E-10 | 9.1E-05 | 41.29 |  |
| Left-leg FFM | rs1147345 | | 132212511 | T | A | 0.526 | -0.007 | 0.001 | 2.90E-08 | 6.8E-05 | 30.75 |  |
| Left-leg FFM | rs1412234 | | 28410683 | C | T | 0.327 | 0.013 | 0.001 | 2.20E-20 | 1.9E-04 | 85.59 | BMI |
| Left-leg FFM | rs12238669 | | 128011403 | G | A | 0.469 | -0.010 | 0.001 | 1.60E-14 | 1.3E-04 | 58.99 | BMI |
| Left-leg FFM | rs1330199 | | 27760946 | T | G | 0.483 | -0.008 | 0.001 | 6.80E-09 | 7.4E-05 | 33.60 | BMI |
| Left-leg FFM | rs10979612 | | 111687379 | C | T | 0.073 | 0.018 | 0.003 | 1.70E-13 | 1.2E-04 | 54.36 |  |
| Left-leg FFM | rs7080472 | | 96012950 | T | G | 0.425 | 0.012 | 0.001 | 5.00E-19 | 1.7E-04 | 79.42 |  |
| Left-leg FFM | rs10886477 | | 121175524 | A | G | 0.128 | 0.012 | 0.002 | 6.80E-10 | 8.4E-05 | 38.08 |  |
| Left-leg FFM | rs12764498 | | 5042294 | C | T | 0.118 | -0.013 | 0.002 | 6.50E-11 | 9.4E-05 | 42.68 |  |
| Left-leg FFM | rs73601548 | | 18549889 | T | C | 0.115 | 0.014 | 0.002 | 7.10E-12 | 1.0E-04 | 47.00 | BMI |
| Left-leg FFM | rs68156080 | | 63781673 | G | A | 0.274 | -0.010 | 0.001 | 1.10E-11 | 1.0E-04 | 46.19 |  |
| Left-leg FFM | rs224143 | | 64477836 | A | G | 0.600 | -0.009 | 0.001 | 4.20E-12 | 1.1E-04 | 48.03 |  |
| Left-leg FFM | rs7100187 | | 66666847 | A | G | 0.505 | -0.007 | 0.001 | 2.10E-08 | 6.9E-05 | 31.38 |  |
| Left-leg FFM | rs10887571 | | 88030441 | T | C | 0.448 | 0.008 | 0.001 | 1.10E-09 | 8.2E-05 | 37.19 | BMI |
| Left-leg FFM | rs117944670 | | 98645695 | C | T | 0.053 | -0.016 | 0.003 | 1.90E-08 | 6.9E-05 | 31.59 |  |
| Left-leg FFM | rs6585827 | | 124165615 | A | G | 0.471 | 0.012 | 0.001 | 4.10E-19 | 1.8E-04 | 79.84 |  |
| Left-leg FFM | rs2666773 | | 22123768 | T | A | 0.711 | -0.009 | 0.001 | 2.90E-09 | 7.8E-05 | 35.26 | BMI |
| Left-leg FFM | rs11014285 | | 25178864 | A | G | 0.165 | 0.018 | 0.002 | 3.90E-23 | 2.2E-04 | 98.15 |  |
| Left-leg FFM | rs1657222 | | 34837139 | A | G | 0.396 | 0.009 | 0.001 | 6.70E-11 | 9.4E-05 | 42.60 |  |
| Left-leg FFM | rs10995366 | | 52772113 | A | G | 0.251 | -0.009 | 0.002 | 6.70E-10 | 8.4E-05 | 38.10 |  |
| Left-leg FFM | rs4255484 | | 77215583 | G | C | 0.559 | -0.009 | 0.001 | 6.50E-12 | 1.0E-04 | 47.16 |  |
| Left-leg FFM | rs1316312 | | 81110417 | C | G | 0.500 | -0.010 | 0.001 | 5.30E-15 | 1.3E-04 | 61.15 |  |
| Left-leg FFM | rs10883560 | | 102673707 | G | C | 0.429 | 0.017 | 0.001 | 4.80E-37 | 3.6E-04 | 161.71 | BMI |
| Left-leg FFM | rs11245450 | | 126658075 | A | G | 0.422 | -0.011 | 0.001 | 1.90E-16 | 1.5E-04 | 67.75 | BMI |
| Left-leg FFM | rs58790726 | | 70240379 | C | G | 0.429 | 0.010 | 0.001 | 1.20E-13 | 1.2E-04 | 55.00 |  |
| Left-leg FFM | rs117543413 | | 79543740 | T | C | 0.018 | -0.040 | 0.005 | 9.70E-16 | 1.4E-04 | 64.48 |  |
| Left-leg FFM | rs7912286 | | 114693230 | G | A | 0.602 | -0.009 | 0.001 | 2.90E-11 | 9.7E-05 | 44.21 |  |
| Left-leg FFM | rs7916178 | | 131144249 | T | C | 0.347 | 0.008 | 0.001 | 4.20E-08 | 6.6E-05 | 30.04 | BMI |
| Left-leg FFM | rs11191515 | | 104776527 | A | G | 0.078 | 0.025 | 0.002 | 4.70E-24 | 2.2E-04 | 102.35 | BMI |
| Left-leg FFM | rs7077087 | | 115954458 | G | A | 0.246 | 0.010 | 0.002 | 1.60E-11 | 1.0E-04 | 45.44 |  |
| Left-leg FFM | rs6591 | | 840363 | T | C | 0.544 | 0.010 | 0.001 | 5.60E-14 | 1.2E-04 | 56.50 | BMI |
| Left-leg FFM | rs676105 | | 30443688 | C | T | 0.304 | 0.013 | 0.001 | 1.50E-20 | 1.9E-04 | 86.34 | BMI |
| Left-leg FFM | rs2293576 | | 47434986 | A | G | 0.332 | -0.012 | 0.001 | 9.20E-18 | 1.6E-04 | 73.67 | BMI |
| Left-leg FFM | rs7129320 | | 68388220 | A | G | 0.166 | -0.021 | 0.002 | 1.40E-34 | 3.3E-04 | 150.46 |  |
| Left-leg FFM | rs1813212 | | 89301382 | G | A | 0.446 | -0.009 | 0.001 | 2.20E-12 | 1.1E-04 | 49.30 |  |
| Left-leg FFM | rs7933085 | | 130796248 | G | A | 0.508 | 0.008 | 0.001 | 3.50E-10 | 8.7E-05 | 39.38 | BMI |
| Left-leg FFM | rs11042321 | | 9399731 | A | G | 0.408 | 0.010 | 0.001 | 4.30E-13 | 1.2E-04 | 52.49 |  |
| Left-leg FFM | rs1789167 | | 69486244 | G | A | 0.653 | 0.012 | 0.001 | 2.30E-18 | 1.7E-04 | 76.40 | BMI |
| Left-leg FFM | rs744205 | | 69929677 | A | G | 0.544 | 0.011 | 0.001 | 1.30E-17 | 1.6E-04 | 72.94 |  |
| Left-leg FFM | rs7932719 | | 87449071 | T | A | 0.244 | 0.008 | 0.002 | 4.60E-08 | 6.6E-05 | 29.88 |  |
| Left-leg FFM | rs1869912 | | 93186433 | T | G | 0.420 | 0.007 | 0.001 | 2.40E-08 | 6.8E-05 | 31.12 |  |
| Left-leg FFM | rs11217863 | | 120293138 | A | G | 0.116 | -0.016 | 0.002 | 1.80E-14 | 1.3E-04 | 58.70 |  |
| Left-leg FFM | rs74749286 | | 14322359 | A | G | 0.108 | 0.014 | 0.002 | 1.10E-10 | 9.2E-05 | 41.68 |  |
| Left-leg FFM | rs35186585 | | 43878534 | C | A | 0.231 | 0.012 | 0.002 | 2.30E-14 | 1.3E-04 | 58.25 | BMI |
| Left-leg FFM | rs7952436 | | 67024534 | T | C | 0.082 | -0.030 | 0.002 | 5.00E-36 | 3.5E-04 | 157.03 |  |
| Left-leg FFM | rs66462629 | | 70373289 | A | C | 0.116 | -0.011 | 0.002 | 2.80E-08 | 6.8E-05 | 30.87 |  |
| Left-leg FFM | rs74048171 | | 2093603 | A | C | 0.258 | -0.011 | 0.001 | 1.90E-14 | 1.3E-04 | 58.63 |  |
| Left-leg FFM | rs35506085 | | 2165576 | A | G | 0.185 | -0.017 | 0.002 | 3.30E-24 | 2.3E-04 | 103.01 |  |
| Left-leg FFM | rs11030112 | | 27705188 | A | G | 0.319 | 0.018 | 0.001 | 2.00E-38 | 3.7E-04 | 168.06 | BMI |
| Left-leg FFM | rs573455 | | 117267884 | G | A | 0.534 | 0.007 | 0.001 | 4.20E-08 | 6.6E-05 | 30.08 |  |
| Left-leg FFM | rs7111235 | | 133658661 | C | T | 0.494 | 0.008 | 0.001 | 2.10E-10 | 8.9E-05 | 40.41 |  |
| Left-leg FFM | rs12364470 | | 134601012 | G | T | 0.165 | 0.011 | 0.002 | 2.60E-10 | 8.8E-05 | 39.94 | BMI |
| Left-leg FFM | rs10845408 | | 11880581 | T | C | 0.353 | 0.010 | 0.001 | 1.70E-13 | 1.2E-04 | 54.30 |  |
| Left-leg FFM | rs11052457 | | 33268956 | T | A | 0.038 | 0.024 | 0.003 | 4.30E-12 | 1.1E-04 | 48.00 |  |
| Left-leg FFM | rs2241960 | | 46580618 | G | A | 0.274 | 0.008 | 0.001 | 2.40E-08 | 6.8E-05 | 31.15 |  |
| Left-leg FFM | rs7959830 | | 66347368 | T | G | 0.413 | -0.024 | 0.001 | 1.90E-76 | 7.5E-04 | 342.38 |  |
| Left-leg FFM | rs2369463 | | 77432523 | C | T | 0.170 | -0.010 | 0.002 | 1.40E-08 | 7.1E-05 | 32.20 |  |
| Left-leg FFM | rs310796 | | 77453226 | T | G | 0.681 | 0.011 | 0.001 | 2.10E-15 | 1.4E-04 | 62.99 |  |
| Left-leg FFM | rs2041895 | | 107350088 | G | C | 0.434 | -0.009 | 0.001 | 9.00E-11 | 9.2E-05 | 42.04 |  |
| Left-leg FFM | rs11065979 | | 112059557 | T | C | 0.438 | -0.012 | 0.001 | 7.90E-20 | 1.8E-04 | 83.07 |  |
| Left-leg FFM | rs11058226 | | 122938958 | C | T | 0.742 | 0.019 | 0.001 | 5.00E-39 | 3.8E-04 | 170.78 | BMI |
| Left-leg FFM | rs11060406 | | 123339117 | T | C | 0.037 | -0.028 | 0.004 | 1.20E-15 | 1.4E-04 | 64.01 |  |
| Left-leg FFM | rs2291256 | | 133393323 | T | C | 0.088 | 0.017 | 0.002 | 6.00E-14 | 1.2E-04 | 56.39 |  |
| Left-leg FFM | rs4980826 | | 578349 | A | C | 0.396 | 0.008 | 0.001 | 8.50E-09 | 7.3E-05 | 33.17 |  |
| Left-leg FFM | rs12099669 | | 46783653 | A | G | 0.696 | 0.015 | 0.001 | 3.20E-26 | 2.5E-04 | 112.22 |  |
| Left-leg FFM | rs7132908 | | 50263148 | A | G | 0.384 | 0.019 | 0.001 | 9.60E-47 | 4.5E-04 | 206.12 | BMI |
| Left-leg FFM | rs2277339 | | 57146069 | G | T | 0.104 | -0.017 | 0.002 | 7.30E-15 | 1.3E-04 | 60.52 | BMI |
| Left-leg FFM | rs58035271 | | 66127644 | C | G | 0.243 | -0.010 | 0.002 | 1.80E-11 | 9.9E-05 | 45.19 |  |
| Left-leg FFM | rs146714063 | | 97498661 | A | G | 0.158 | 0.010 | 0.002 | 1.70E-08 | 7.0E-05 | 31.84 |  |
| Left-leg FFM | rs7971536 | | 102373788 | A | T | 0.494 | -0.010 | 0.001 | 1.40E-15 | 1.4E-04 | 63.73 |  |
| Left-leg FFM | rs78812993 | | 103175170 | C | G | 0.052 | -0.022 | 0.003 | 8.50E-14 | 1.2E-04 | 55.69 |  |
| Left-leg FFM | rs67551338 | | 3393100 | T | C | 0.061 | 0.024 | 0.003 | 8.60E-19 | 1.7E-04 | 78.35 |  |
| Left-leg FFM | rs76895963 | | 4384844 | G | T | 0.021 | 0.095 | 0.005 | 9.60E-80 | 7.9E-04 | 357.55 | BMI |
| Left-leg FFM | rs774214 | | 56918566 | C | T | 0.663 | -0.010 | 0.001 | 9.70E-13 | 1.1E-04 | 50.91 |  |
| Left-leg FFM | rs2241212 | | 109939641 | A | T | 0.505 | 0.008 | 0.001 | 6.20E-10 | 8.4E-05 | 38.26 |  |
| Left-leg FFM | rs11065015 | | 120520863 | T | C | 0.027 | -0.030 | 0.004 | 1.30E-13 | 1.2E-04 | 54.89 |  |
| Left-leg FFM | rs7487292 | | 122614387 | G | T | 0.447 | 0.011 | 0.001 | 4.60E-16 | 1.5E-04 | 65.98 | BMI |
| Left-leg FFM | rs55726687 | | 991306 | A | G | 0.210 | 0.016 | 0.002 | 7.60E-24 | 2.2E-04 | 101.38 | BMI |
| Left-leg FFM | rs117451679 | | 17245591 | G | A | 0.106 | 0.013 | 0.002 | 4.90E-10 | 8.5E-05 | 38.71 |  |
| Left-leg FFM | rs11049386 | | 28320536 | A | T | 0.290 | -0.009 | 0.001 | 2.80E-09 | 7.8E-05 | 35.33 |  |
| Left-leg FFM | rs1049193 | | 53873844 | C | G | 0.171 | -0.015 | 0.002 | 5.60E-18 | 1.6E-04 | 74.66 |  |
| Left-leg FFM | rs12813149 | | 90142637 | A | G | 0.243 | -0.011 | 0.002 | 1.90E-13 | 1.2E-04 | 54.06 | BMI |
| Left-leg FFM | rs2287547 | | 108924341 | C | T | 0.168 | 0.010 | 0.002 | 1.80E-08 | 7.0E-05 | 31.66 |  |
| Left-leg FFM | rs529736 | | 122384457 | C | A | 0.833 | -0.010 | 0.002 | 2.10E-09 | 7.9E-05 | 35.85 |  |
| Left-leg FFM | rs7969505 | | 12827900 | C | G | 0.114 | -0.013 | 0.002 | 3.50E-10 | 8.7E-05 | 39.38 |  |
| Left-leg FFM | rs7134283 | | 24071748 | A | G | 0.283 | -0.011 | 0.001 | 4.00E-14 | 1.3E-04 | 57.14 |  |
| Left-leg FFM | rs10843397 | | 29529523 | T | C | 0.242 | 0.009 | 0.002 | 6.80E-09 | 7.4E-05 | 33.58 |  |
| Left-leg FFM | rs12230050 | | 93990553 | G | A | 0.261 | 0.019 | 0.001 | 2.50E-36 | 3.5E-04 | 158.45 |  |
| Left-leg FFM | rs2229840 | | 124826462 | T | C | 0.160 | 0.021 | 0.002 | 1.90E-31 | 3.0E-04 | 136.12 |  |
| Left-leg FFM | rs1218822 | | 28011963 | A | G | 0.661 | 0.011 | 0.001 | 1.70E-14 | 1.3E-04 | 58.80 | BMI |
| Left-leg FFM | rs9549099 | | 40766349 | C | T | 0.265 | 0.010 | 0.001 | 3.80E-12 | 1.1E-04 | 48.20 | BMI |
| Left-leg FFM | rs9317002 | | 59175727 | A | C | 0.515 | 0.011 | 0.001 | 3.10E-18 | 1.7E-04 | 75.84 | BMI |
| Left-leg FFM | rs7985813 | | 99592595 | A | G | 0.735 | -0.009 | 0.001 | 5.40E-09 | 7.5E-05 | 34.03 |  |
| Left-leg FFM | rs7994814 | | 27054201 | T | C | 0.417 | 0.012 | 0.001 | 4.40E-20 | 1.9E-04 | 84.22 |  |
| Left-leg FFM | rs9540493 | | 66205704 | G | A | 0.545 | -0.010 | 0.001 | 1.10E-13 | 1.2E-04 | 55.21 | BMI |
| Left-leg FFM | rs11616283 | | 21493853 | C | T | 0.140 | 0.011 | 0.002 | 9.80E-09 | 7.2E-05 | 32.89 |  |
| Left-leg FFM | rs2225226 | | 51127270 | T | C | 0.216 | -0.029 | 0.002 | 3.90E-73 | 7.2E-04 | 327.20 |  |
| Left-leg FFM | rs1924936 | | 78443297 | A | T | 0.774 | 0.016 | 0.002 | 9.00E-26 | 2.4E-04 | 110.17 |  |
| Left-leg FFM | rs7994573 | | 115008388 | C | T | 0.235 | -0.010 | 0.002 | 5.00E-11 | 9.5E-05 | 43.17 |  |
| Left-leg FFM | rs1373273 | | 53973351 | A | C | 0.571 | -0.007 | 0.001 | 3.90E-08 | 6.6E-05 | 30.19 |  |
| Left-leg FFM | rs532499 | | 30165465 | C | T | 0.741 | -0.008 | 0.001 | 1.60E-08 | 7.0E-05 | 31.93 |  |
| Left-leg FFM | rs9532631 | | 41449737 | T | G | 0.198 | -0.011 | 0.002 | 1.00E-10 | 9.2E-05 | 41.82 |  |
| Left-leg FFM | rs146851424 | | 50377910 | C | A | 0.022 | 0.057 | 0.005 | 1.20E-36 | 3.5E-04 | 159.87 |  |
| Left-leg FFM | rs61950323 | | 54070046 | T | C | 0.058 | 0.020 | 0.003 | 3.20E-12 | 1.1E-04 | 48.56 | BMI |
| Left-leg FFM | rs9513143 | | 97085570 | G | A | 0.542 | 0.010 | 0.001 | 1.60E-13 | 1.2E-04 | 54.50 | BMI |
| Left-leg FFM | rs36100359 | | 21578007 | A | G | 0.139 | -0.011 | 0.002 | 1.80E-09 | 7.9E-05 | 36.13 |  |
| Left-leg FFM | rs12432051 | | 94082464 | T | A | 0.692 | 0.011 | 0.001 | 7.10E-15 | 1.3E-04 | 60.56 | BMI |
| Left-leg FFM | rs11627567 | | 103851775 | T | C | 0.215 | -0.011 | 0.002 | 8.50E-12 | 1.0E-04 | 46.65 | BMI |
| Left-leg FFM | rs17197114 | | 21894526 | C | T | 0.177 | 0.011 | 0.002 | 3.60E-10 | 8.6E-05 | 39.30 |  |
| Left-leg FFM | rs112957890 | | 36220876 | G | A | 0.265 | 0.010 | 0.001 | 5.90E-12 | 1.0E-04 | 47.38 |  |
| Left-leg FFM | rs117034105 | | 53531085 | C | T | 0.049 | 0.019 | 0.003 | 3.90E-09 | 7.6E-05 | 34.69 |  |
| Left-leg FFM | rs17105272 | | 77529783 | T | C | 0.329 | 0.009 | 0.001 | 1.80E-11 | 9.9E-05 | 45.14 |  |
| Left-leg FFM | rs148740466 | | 89016257 | A | C | 0.016 | -0.029 | 0.005 | 1.40E-08 | 7.1E-05 | 32.23 |  |
| Left-leg FFM | rs12879423 | | 25927832 | G | A | 0.679 | 0.015 | 0.001 | 1.50E-26 | 2.5E-04 | 113.69 | BMI |
| Left-leg FFM | rs12889702 | | 42894143 | C | A | 0.313 | 0.011 | 0.001 | 6.90E-14 | 1.2E-04 | 56.08 |  |
| Left-leg FFM | rs2296316 | | 65520246 | C | T | 0.465 | -0.008 | 0.001 | 1.10E-08 | 7.2E-05 | 32.70 |  |
| Left-leg FFM | rs1286058 | | 91458523 | A | T | 0.704 | 0.010 | 0.001 | 6.80E-12 | 1.0E-04 | 47.08 | BMI |
| Left-leg FFM | rs112097633 | | 93131683 | A | G | 0.077 | 0.015 | 0.002 | 5.40E-10 | 8.5E-05 | 38.52 |  |
| Left-leg FFM | rs61992671 | | 101531854 | G | A | 0.492 | -0.012 | 0.001 | 7.10E-18 | 1.6E-04 | 74.20 | BMI |
| Left-leg FFM | rs12880432 | | 103267500 | C | G | 0.647 | -0.008 | 0.001 | 9.20E-09 | 7.3E-05 | 33.01 | BMI |
| Left-leg FFM | rs3212260 | | 24804088 | T | A | 0.258 | 0.012 | 0.001 | 1.80E-15 | 1.4E-04 | 63.23 |  |
| Left-leg FFM | rs8007644 | | 35218831 | A | G | 0.388 | 0.008 | 0.001 | 3.00E-09 | 7.7E-05 | 35.19 |  |
| Left-leg FFM | rs4899012 | | 61003889 | C | G | 0.607 | -0.019 | 0.001 | 4.00E-45 | 4.4E-04 | 198.72 |  |
| Left-leg FFM | rs7141420 | | 79899454 | T | C | 0.516 | 0.014 | 0.001 | 5.40E-28 | 2.6E-04 | 120.30 | BMI |
| Left-leg FFM | rs9788443 | | 23878279 | C | T | 0.048 | 0.017 | 0.003 | 2.10E-08 | 6.9E-05 | 31.42 |  |
| Left-leg FFM | rs12147845 | | 101144596 | T | C | 0.116 | 0.013 | 0.002 | 1.80E-10 | 8.9E-05 | 40.63 | BMI |
| Left-leg FFM | rs12906197 | | 38492199 | T | C | 0.422 | -0.010 | 0.001 | 1.60E-13 | 1.2E-04 | 54.49 |  |
| Left-leg FFM | rs55887408 | | 52073816 | A | G | 0.450 | -0.008 | 0.001 | 1.20E-09 | 8.1E-05 | 37.02 | BMI |
| Left-leg FFM | rs4143843 | | 62373121 | T | C | 0.448 | -0.010 | 0.001 | 1.30E-13 | 1.2E-04 | 54.80 | BMI |
| Left-leg FFM | rs9920235 | | 79384021 | T | C | 0.399 | -0.009 | 0.001 | 3.40E-12 | 1.1E-04 | 48.44 | BMI |
| Left-leg FFM | rs11638457 | | 81012407 | G | C | 0.444 | 0.010 | 0.001 | 5.80E-15 | 1.3E-04 | 60.98 | BMI |
| Left-leg FFM | rs11630112 | | 86120246 | C | T | 0.268 | 0.009 | 0.001 | 9.20E-10 | 8.2E-05 | 37.49 |  |
| Left-leg FFM | rs11855017 | | 42096146 | A | C | 0.182 | 0.013 | 0.002 | 3.60E-14 | 1.3E-04 | 57.40 | BMI |
| Left-leg FFM | rs2414910 | | 66976149 | T | G | 0.788 | -0.010 | 0.002 | 4.40E-10 | 8.6E-05 | 38.94 |  |
| Left-leg FFM | rs8040399 | | 89432795 | G | C | 0.588 | -0.008 | 0.001 | 3.20E-10 | 8.7E-05 | 39.54 |  |
| Left-leg FFM | rs1573891 | | 99186488 | C | G | 0.158 | -0.019 | 0.002 | 4.80E-25 | 2.3E-04 | 106.86 |  |
| Left-leg FFM | rs11071182 | | 55644676 | G | A | 0.870 | 0.015 | 0.002 | 3.60E-14 | 1.3E-04 | 57.36 |  |
| Left-leg FFM | rs8030456 | | 68076856 | T | C | 0.226 | -0.014 | 0.002 | 2.90E-19 | 1.8E-04 | 80.50 | BMI |
| Left-leg FFM | rs62621400 | | 101718239 | G | C | 0.058 | -0.021 | 0.003 | 2.50E-13 | 1.2E-04 | 53.57 |  |
| Left-leg FFM | rs2663125 | | 99563891 | C | T | 0.691 | -0.009 | 0.001 | 3.30E-11 | 9.7E-05 | 44.02 |  |
| Left-leg FFM | rs13333747 | | 2175373 | C | T | 0.183 | -0.018 | 0.002 | 4.80E-27 | 2.5E-04 | 115.97 | BMI |
| Left-leg FFM | rs2539999 | | 2266173 | C | T | 0.287 | 0.015 | 0.001 | 7.20E-24 | 2.2E-04 | 101.48 |  |
| Left-leg FFM | rs35467921 | | 30048553 | T | C | 0.400 | 0.021 | 0.001 | 1.60E-57 | 5.6E-04 | 255.57 | BMI |
| Left-leg FFM | rs7188009 | | 81660642 | A | G | 0.404 | 0.008 | 0.001 | 2.00E-09 | 7.9E-05 | 35.93 |  |
| Left-leg FFM | rs12926311 | | 406427 | C | G | 0.354 | -0.009 | 0.001 | 5.90E-11 | 9.4E-05 | 42.85 | BMI |
| Left-leg FFM | rs9926458 | | 4872628 | A | G | 0.379 | 0.009 | 0.001 | 1.80E-11 | 9.9E-05 | 45.23 | BMI |
| Left-leg FFM | rs11648796 | | 792190 | G | A | 0.232 | 0.014 | 0.002 | 8.30E-19 | 1.7E-04 | 78.42 |  |
| Left-leg FFM | rs2238435 | | 4014282 | G | C | 0.614 | 0.019 | 0.001 | 2.10E-44 | 4.3E-04 | 195.45 | BMI |
| Left-leg FFM | rs4985124 | | 15125441 | G | T | 0.297 | -0.012 | 0.001 | 4.20E-17 | 1.6E-04 | 70.69 |  |
| Left-leg FFM | rs34017457 | | 67166731 | A | G | 0.007 | 0.050 | 0.008 | 1.60E-10 | 9.0E-05 | 40.89 |  |
| Left-leg FFM | rs71393968 | | 67847341 | A | G | 0.046 | 0.020 | 0.003 | 5.20E-11 | 9.5E-05 | 43.09 |  |
| Left-leg FFM | rs72801843 | | 53508802 | A | T | 0.301 | 0.017 | 0.001 | 1.70E-32 | 3.1E-04 | 140.86 |  |
| Left-leg FFM | rs8059189 | | 86417349 | A | G | 0.404 | -0.011 | 0.001 | 8.40E-15 | 1.3E-04 | 60.25 |  |
| Left-leg FFM | rs10775348 | | 88806348 | G | A | 0.704 | 0.012 | 0.001 | 1.10E-16 | 1.5E-04 | 68.87 |  |
| Left-leg FFM | rs72771080 | | 20021383 | T | C | 0.212 | 0.014 | 0.002 | 6.30E-18 | 1.6E-04 | 74.43 | BMI |
| Left-leg FFM | rs35626515 | | 28649651 | A | C | 0.405 | 0.017 | 0.001 | 2.40E-36 | 3.5E-04 | 158.48 | BMI |
| Left-leg FFM | rs56094641 | | 53806453 | G | A | 0.405 | 0.042 | 0.001 | 1.00E-200 | 2.2E-03 | 1004.93 | BMI |
| Left-leg FFM | rs76513770 | | 72505534 | C | T | 0.128 | -0.017 | 0.002 | 1.50E-17 | 1.6E-04 | 72.67 |  |
| Left-leg FFM | rs55831773 | | 7559037 | T | C | 0.199 | -0.015 | 0.002 | 2.50E-19 | 1.8E-04 | 80.79 |  |
| Left-leg FFM | rs1242510 | | 17371405 | T | C | 0.801 | 0.010 | 0.002 | 6.00E-09 | 7.4E-05 | 33.82 |  |
| Left-leg FFM | rs72833620 | | 47056753 | C | A | 0.096 | 0.018 | 0.002 | 1.70E-15 | 1.4E-04 | 63.34 | BMI |
| Left-leg FFM | rs2005172 | | 61996255 | C | A | 0.640 | 0.019 | 0.001 | 5.40E-45 | 4.4E-04 | 198.11 |  |
| Left-leg FFM | rs34055910 | | 64460104 | G | A | 0.379 | -0.007 | 0.001 | 4.60E-08 | 6.6E-05 | 29.87 |  |
| Left-leg FFM | rs2592209 | | 67408931 | C | G | 0.513 | -0.007 | 0.001 | 2.40E-08 | 6.8E-05 | 31.15 |  |
| Left-leg FFM | rs4968799 | | 68158831 | T | A | 0.112 | -0.017 | 0.002 | 1.90E-16 | 1.5E-04 | 67.69 |  |
| Left-leg FFM | rs236587 | | 68203915 | C | T | 0.737 | -0.008 | 0.001 | 4.70E-08 | 6.6E-05 | 29.82 |  |
| Left-leg FFM | rs2252909 | | 2278609 | T | C | 0.657 | -0.008 | 0.001 | 2.00E-08 | 6.9E-05 | 31.53 |  |
| Left-leg FFM | rs2301652 | | 15928584 | G | T | 0.543 | 0.009 | 0.001 | 1.30E-11 | 1.0E-04 | 45.75 | BMI |
| Left-leg FFM | rs3110496 | | 27917771 | G | A | 0.686 | 0.008 | 0.001 | 6.80E-09 | 7.4E-05 | 33.59 |  |
| Left-leg FFM | rs6505044 | | 54258240 | C | A | 0.462 | -0.008 | 0.001 | 8.20E-09 | 7.3E-05 | 33.22 |  |
| Left-leg FFM | rs757608 | | 59497277 | G | A | 0.670 | -0.012 | 0.001 | 2.30E-18 | 1.7E-04 | 76.41 | BMI |
| Left-leg FFM | rs4525525 | | 1866892 | T | G | 0.264 | -0.011 | 0.001 | 4.50E-13 | 1.2E-04 | 52.40 | BMI |
| Left-leg FFM | rs28613067 | | 46781635 | A | G | 0.278 | 0.009 | 0.001 | 2.10E-10 | 8.9E-05 | 40.35 |  |
| Left-leg FFM | rs12946718 | | 77777125 | A | C | 0.226 | -0.009 | 0.002 | 4.80E-09 | 7.5E-05 | 34.26 |  |
| Left-leg FFM | rs36000545 | | 79093822 | G | A | 0.396 | -0.016 | 0.001 | 5.90E-32 | 3.0E-04 | 138.41 |  |
| Left-leg FFM | rs78378222 | | 7571752 | G | T | 0.012 | 0.077 | 0.006 | 6.70E-37 | 3.5E-04 | 161.03 |  |
| Left-leg FFM | rs2314338 | | 38344485 | C | T | 0.269 | 0.011 | 0.001 | 7.50E-13 | 1.1E-04 | 51.42 |  |
| Left-leg FFM | rs62070648 | | 29210595 | A | G | 0.269 | -0.022 | 0.001 | 8.20E-50 | 4.8E-04 | 220.20 |  |
| Left-leg FFM | rs12951408 | | 36913807 | C | T | 0.559 | 0.011 | 0.001 | 1.20E-16 | 1.5E-04 | 68.69 |  |
| Left-leg FFM | rs6416914 | | 39262850 | G | A | 0.695 | -0.009 | 0.001 | 4.50E-11 | 9.5E-05 | 43.39 | BMI |
| Left-leg FFM | rs9889839 | | 78270679 | G | A | 0.455 | 0.007 | 0.001 | 2.30E-08 | 6.9E-05 | 31.25 |  |
| Left-leg FFM | rs7220294 | | 78627753 | C | T | 0.233 | -0.011 | 0.002 | 2.00E-12 | 1.1E-04 | 49.49 | BMI |
| Left-leg FFM | rs4392169 | | 20724931 | T | A | 0.785 | 0.020 | 0.002 | 1.30E-36 | 3.5E-04 | 159.75 |  |
| Left-leg FFM | rs9957318 | | 33039106 | G | A | 0.348 | 0.008 | 0.001 | 2.00E-09 | 7.9E-05 | 35.93 |  |
| Left-leg FFM | rs2276190 | | 63430340 | A | G | 0.277 | 0.009 | 0.001 | 4.70E-10 | 8.5E-05 | 38.79 | BMI |
| Left-leg FFM | rs57126421 | | 2656989 | G | A | 0.238 | -0.011 | 0.002 | 4.80E-13 | 1.1E-04 | 52.29 |  |
| Left-leg FFM | rs55854145 | | 45928049 | C | A | 0.055 | -0.016 | 0.003 | 1.40E-08 | 7.1E-05 | 32.24 |  |
| Left-leg FFM | rs33973388 | | 46611842 | T | G | 0.435 | 0.010 | 0.001 | 6.30E-14 | 1.2E-04 | 56.27 |  |
| Left-leg FFM | rs6567160 | | 57829135 | C | T | 0.233 | 0.045 | 0.002 | 2.30E-185 | 1.9E-03 | 843.09 | BMI |
| Left-leg FFM | rs1941697 | | 31251276 | A | G | 0.449 | 0.008 | 0.001 | 1.80E-09 | 8.0E-05 | 36.20 | BMI |
| Left-leg FFM | rs4552110 | | 57183948 | T | A | 0.158 | -0.011 | 0.002 | 8.80E-10 | 8.3E-05 | 37.58 |  |
| Left-leg FFM | rs751894 | | 60756884 | T | C | 0.206 | 0.009 | 0.002 | 1.30E-08 | 7.1E-05 | 32.31 |  |
| Left-leg FFM | rs74494415 | | 74972138 | T | C | 0.040 | -0.023 | 0.003 | 6.40E-12 | 1.0E-04 | 47.21 |  |
| Left-leg FFM | rs11663903 | | 32762592 | A | G | 0.429 | 0.008 | 0.001 | 1.20E-08 | 7.1E-05 | 32.46 |  |
| Left-leg FFM | rs7229520 | | 46516468 | A | G | 0.662 | -0.010 | 0.001 | 2.10E-13 | 1.2E-04 | 53.93 |  |
| Left-leg FFM | rs1517037 | | 56878274 | T | C | 0.188 | -0.014 | 0.002 | 8.30E-17 | 1.5E-04 | 69.34 |  |
| Left-leg FFM | rs57636386 | | 58048295 | C | T | 0.084 | -0.025 | 0.002 | 2.50E-25 | 2.4E-04 | 108.11 | BMI |
| Left-leg FFM | rs2163832 | | 10745764 | C | T | 0.660 | -0.012 | 0.001 | 1.30E-19 | 1.8E-04 | 82.08 |  |
| Left-leg FFM | rs73004967 | | 19717056 | G | A | 0.069 | -0.021 | 0.003 | 5.30E-16 | 1.4E-04 | 65.66 |  |
| Left-leg FFM | rs284662 | | 41932275 | C | T | 0.620 | -0.009 | 0.001 | 1.00E-10 | 9.2E-05 | 41.81 |  |
| Left-leg FFM | rs35050648 | | 46991243 | T | G | 0.236 | 0.009 | 0.002 | 4.50E-09 | 7.6E-05 | 34.39 |  |
| Left-leg FFM | rs11880992 | | 2176403 | A | G | 0.408 | 0.012 | 0.001 | 6.10E-20 | 1.8E-04 | 83.57 |  |
| Left-leg FFM | rs58857770 | | 7208110 | G | C | 0.408 | -0.008 | 0.001 | 5.80E-10 | 8.4E-05 | 38.38 |  |
| Left-leg FFM | rs147110934 | | 55993436 | T | G | 0.024 | -0.028 | 0.004 | 2.50E-11 | 9.8E-05 | 44.51 |  |
| Left-leg FFM | rs7253519 | | 1986520 | A | G | 0.119 | -0.013 | 0.002 | 1.00E-10 | 9.2E-05 | 41.76 | BMI |
| Left-leg FFM | rs62621197 | | 8670147 | T | C | 0.037 | -0.034 | 0.004 | 1.70E-21 | 2.0E-04 | 90.66 |  |
| Left-leg FFM | rs3810291 | | 47569003 | A | G | 0.675 | 0.018 | 0.001 | 2.10E-39 | 3.8E-04 | 172.50 | BMI |
| Left-leg FFM | rs4807473 | | 3448869 | G | A | 0.642 | -0.010 | 0.001 | 1.60E-14 | 1.3E-04 | 58.99 |  |
| Left-leg FFM | rs4808845 | | 18812024 | G | A | 0.400 | -0.009 | 0.001 | 4.30E-11 | 9.6E-05 | 43.46 | BMI |
| Left-leg FFM | rs111640872 | | 30290357 | C | G | 0.331 | 0.016 | 0.001 | 2.70E-29 | 2.8E-04 | 126.27 | BMI |
| Left-leg FFM | rs2252720 | | 21223663 | T | C | 0.674 | -0.010 | 0.001 | 4.70E-13 | 1.2E-04 | 52.33 |  |
| Left-leg FFM | rs34879158 | | 32300634 | C | A | 0.263 | -0.020 | 0.001 | 6.40E-41 | 3.9E-04 | 179.46 |  |
| Left-leg FFM | rs73619441 | | 61564901 | G | T | 0.144 | -0.012 | 0.002 | 9.50E-11 | 9.2E-05 | 41.93 | BMI |
| Left-leg FFM | rs143384 | | 34025756 | G | A | 0.404 | 0.032 | 0.001 | 1.20E-131 | 1.3E-03 | 595.98 |  |
| Left-leg FFM | rs6051425 | | 2815318 | G | C | 0.510 | -0.008 | 0.001 | 1.00E-09 | 8.2E-05 | 37.25 |  |
| Left-leg FFM | rs16996657 | | 15816236 | C | T | 0.128 | 0.012 | 0.002 | 8.40E-10 | 8.3E-05 | 37.66 | BMI |
| Left-leg FFM | rs6136938 | | 20058992 | A | G | 0.436 | -0.008 | 0.001 | 2.30E-10 | 8.8E-05 | 40.18 |  |
| Left-leg FFM | rs6142059 | | 32544327 | C | T | 0.492 | 0.010 | 0.001 | 1.40E-13 | 1.2E-04 | 54.69 | BMI |
| Left-leg FFM | rs1291066 | | 35790918 | C | G | 0.809 | 0.012 | 0.002 | 1.10E-12 | 1.1E-04 | 50.59 |  |
| Left-leg FFM | rs6026578 | | 57463472 | G | C | 0.625 | -0.010 | 0.001 | 5.90E-14 | 1.2E-04 | 56.41 |  |
| Left-leg FFM | rs2007022 | | 3237674 | A | C | 0.231 | 0.009 | 0.002 | 2.20E-08 | 6.9E-05 | 31.35 |  |
| Left-leg FFM | rs6124249 | | 39175029 | C | T | 0.316 | 0.009 | 0.001 | 5.20E-10 | 8.5E-05 | 38.60 |  |
| Left-leg FFM | rs13043303 | | 51091385 | A | G | 0.183 | -0.019 | 0.002 | 3.00E-28 | 2.7E-04 | 121.49 | BMI |
| Left-leg FFM | rs9976812 | | 39690245 | G | C | 0.564 | -0.013 | 0.001 | 6.80E-23 | 2.1E-04 | 97.05 |  |
| Left-leg FFM | rs73189390 | | 17383170 | A | G | 0.184 | -0.010 | 0.002 | 6.50E-09 | 7.4E-05 | 33.67 |  |
| Left-leg FFM | rs4819021 | | 46466927 | C | T | 0.477 | -0.009 | 0.001 | 1.30E-12 | 1.1E-04 | 50.30 | BMI |
| Left-leg FFM | rs165656 | | 19948863 | C | G | 0.517 | 0.008 | 0.001 | 9.50E-10 | 8.2E-05 | 37.42 |  |
| Left-leg FFM | rs5752989 | | 30365780 | A | G | 0.571 | -0.009 | 0.001 | 6.10E-12 | 1.0E-04 | 47.29 |  |
| Left-leg FFM | rs41311445 | | 42070374 | C | A | 0.096 | -0.022 | 0.002 | 3.30E-22 | 2.1E-04 | 93.88 |  |
| Left-leg FFM | rs113619763 | | 46399175 | T | A | 0.062 | 0.016 | 0.003 | 7.10E-09 | 7.4E-05 | 33.51 |  |
| Left-leg FFM | rs5771118 | | 50714289 | C | T | 0.742 | 0.009 | 0.002 | 6.50E-10 | 8.4E-05 | 38.15 |  |
| Left-leg FFM | rs5762873 | | 29265512 | A | T | 0.045 | -0.018 | 0.003 | 2.20E-08 | 6.9E-05 | 31.32 |  |
|  | |

Abbreviation: SNP, single nucleotide polymorphism; EA, Effect allele; NEA, Non-effect allele; EAF, effect allele frequency; SE, standard error; BMI, body mass index.

*^a^ R^2^* was calculated using the following formula: (2×EAF×(1-EAF)×beta^2^)/[(2×EAF×(1-EAF)×beta^2^)+(2×EAF×(1-EAF)×N×SE^2^)], where EAF is the effect allele

frequency, beta is the estimated effect on urate. Ν is the sample size of the GWAS for the SNP-urate association and SE is the standard error of the estimated effect.

*^b^ F* statistic was calculated using the following formula: *R^2^*(N-2)/(1-*R^2^*), where *R^2^* is the proportion of variance in urate explained by each instrument and N is the sample size of the GWAS for the SNP-urate association.

^c^ SNPs associated with confounding factors were removed after searching Phenoscanner database.

Supplementary Table 8 Characteristics of SNPs used as genetic instruments for left-leg FFM

| Exposure | SNP | | Position | EA | NEA | EAF | SNP-Exposure association | | | R^2 a^ | F-statistic ^b^ | Confounders ^c^ |
| --- | --- | --- | --- | --- | --- | --- | --- | --- | --- | --- | --- | --- |
|  |  | |  |  |  |  | Beta | SE | P value |  |  |  |
| Left-leg FFM | rs1356803 | | 32131163 | G | C | 0.546 | 0.009 | 0.001 | 1.70E-11 | 1.0E-04 | 45.26 |  |
| Left-leg FFM | rs4653016 | | 33776441 | A | C | 0.683 | 0.014 | 0.001 | 5.60E-23 | 2.1E-04 | 97.41 | BMI |
| Left-leg FFM | rs2298200 | | 49361400 | T | C | 0.183 | 0.010 | 0.002 | 7.80E-10 | 8.3E-05 | 37.80 | BMI |
| Left-leg FFM | rs2568958 | | 72765116 | A | G | 0.604 | 0.011 | 0.001 | 1.10E-15 | 1.4E-04 | 64.31 | BMI |
| Left-leg FFM | rs3845344 | | 75001480 | T | C | 0.391 | 0.010 | 0.001 | 2.20E-13 | 1.2E-04 | 53.81 | BMI |
| Left-leg FFM | rs34517439 | | 78450517 | A | C | 0.122 | 0.038 | 0.002 | 1.60E-80 | 7.9E-04 | 361.12 | BMI |
| Left-leg FFM | rs2181375 | | 96940119 | G | A | 0.596 | 0.010 | 0.001 | 1.10E-13 | 1.2E-04 | 55.23 | BMI |
| Left-leg FFM | rs655598 | | 190287713 | A | G | 0.563 | -0.011 | 0.001 | 2.10E-16 | 1.5E-04 | 67.50 | BMI |
| Left-leg FFM | rs2807339 | | 22578063 | C | T | 0.758 | 0.009 | 0.002 | 6.20E-09 | 7.4E-05 | 33.77 |  |
| Left-leg FFM | rs11578046 | | 23425139 | A | G | 0.327 | -0.012 | 0.001 | 1.10E-18 | 1.7E-04 | 77.92 |  |
| Left-leg FFM | rs6669139 | | 56583110 | T | C | 0.209 | 0.016 | 0.002 | 2.40E-23 | 2.2E-04 | 99.08 |  |
| Left-leg FFM | rs10923769 | | 119734551 | G | C | 0.145 | 0.010 | 0.002 | 4.80E-08 | 6.5E-05 | 29.78 |  |
| Left-leg FFM | rs112685832 | | 155822629 | A | C | 0.115 | 0.015 | 0.002 | 1.10E-12 | 1.1E-04 | 50.69 | BMI |
| Left-leg FFM | rs7546249 | | 176792690 | A | T | 0.727 | 0.013 | 0.001 | 6.00E-19 | 1.7E-04 | 79.08 |  |
| Left-leg FFM | rs2678204 | | 201800511 | G | T | 0.340 | 0.013 | 0.001 | 5.40E-21 | 1.9E-04 | 88.37 | BMI |
| Left-leg FFM | rs35492502 | | 217806224 | A | G | 0.296 | 0.010 | 0.001 | 1.70E-12 | 1.1E-04 | 49.75 |  |
| Left-leg FFM | rs2281175 | | 1665702 | C | T | 0.405 | 0.011 | 0.001 | 6.50E-15 | 1.3E-04 | 60.73 | BMI |
| Left-leg FFM | rs212521 | | 21580345 | C | T | 0.601 | 0.007 | 0.001 | 4.90E-08 | 6.5E-05 | 29.74 |  |
| Left-leg FFM | rs17363646 | | 86823503 | G | A | 0.136 | 0.012 | 0.002 | 1.70E-10 | 9.0E-05 | 40.77 |  |
| Left-leg FFM | rs72697614 | | 107514107 | A | C | 0.320 | 0.008 | 0.001 | 3.50E-09 | 7.7E-05 | 34.91 | BMI |
| Left-leg FFM | rs543874 | | 177889480 | G | A | 0.205 | 0.030 | 0.002 | 1.40E-75 | 7.4E-04 | 338.48 | BMI |
| Left-leg FFM | rs55800172 | | 32414767 | A | G | 0.064 | 0.018 | 0.003 | 3.40E-11 | 9.7E-05 | 43.90 |  |
| Left-leg FFM | rs28605759 | | 38399816 | A | G | 0.454 | -0.009 | 0.001 | 3.90E-11 | 9.6E-05 | 43.67 |  |
| Left-leg FFM | rs17491275 | | 39672545 | G | T | 0.160 | 0.019 | 0.002 | 6.70E-27 | 2.5E-04 | 115.31 | BMI |
| Left-leg FFM | rs12140153 | | 62579891 | T | G | 0.094 | -0.020 | 0.002 | 9.40E-18 | 1.6E-04 | 73.64 | BMI |
| Left-leg FFM | rs3009872 | | 66411400 | C | T | 0.435 | -0.007 | 0.001 | 2.10E-08 | 6.9E-05 | 31.41 | BMI |
| Left-leg FFM | rs7550987 | | 96338074 | T | G | 0.385 | 0.008 | 0.001 | 3.20E-10 | 8.7E-05 | 39.54 | BMI |
| Left-leg FFM | rs76798800 | | 154994978 | T | G | 0.266 | 0.023 | 0.001 | 5.80E-54 | 5.3E-04 | 239.21 | BMI |
| Left-leg FFM | rs12731187 | | 202013757 | T | C | 0.358 | -0.008 | 0.001 | 4.30E-09 | 7.6E-05 | 34.49 | BMI |
| Left-leg FFM | rs6675441 | | 214659762 | A | G | 0.234 | -0.012 | 0.002 | 1.40E-13 | 1.2E-04 | 54.74 |  |
| Left-leg FFM | rs2615075 | | 225934295 | G | A | 0.376 | 0.010 | 0.001 | 1.60E-13 | 1.2E-04 | 54.45 |  |
| Left-leg FFM | rs2789370 | | 235503256 | G | A | 0.346 | -0.009 | 0.001 | 1.40E-11 | 1.0E-04 | 45.69 |  |
| Left-leg FFM | rs149229890 | | 2253864 | T | G | 0.011 | 0.039 | 0.006 | 7.50E-10 | 8.3E-05 | 37.88 |  |
| Left-leg FFM | rs67373773 | | 2710377 | A | G | 0.439 | -0.008 | 0.001 | 2.80E-09 | 7.8E-05 | 35.31 | BMI |
| Left-leg FFM | rs1205593 | | 11252716 | C | T | 0.759 | -0.015 | 0.002 | 1.20E-23 | 2.2E-04 | 100.55 | BMI |
| Left-leg FFM | rs66766977 | | 22431092 | C | T | 0.539 | -0.010 | 0.001 | 1.90E-14 | 1.3E-04 | 58.67 |  |
| Left-leg FFM | rs475390 | | 41570459 | A | G | 0.776 | -0.018 | 0.002 | 3.20E-30 | 2.9E-04 | 130.49 |  |
| Left-leg FFM | rs12095997 | | 51391845 | T | C | 0.089 | 0.020 | 0.002 | 2.50E-19 | 1.8E-04 | 80.77 |  |
| Left-leg FFM | rs77848106 | | 107971673 | A | C | 0.296 | -0.010 | 0.001 | 5.10E-12 | 1.0E-04 | 47.65 | BMI |
| Left-leg FFM | rs17024393 | | 110154688 | C | T | 0.026 | 0.037 | 0.004 | 2.50E-19 | 1.8E-04 | 80.80 | BMI |
| Left-leg FFM | rs11205354 | | 150249101 | A | C | 0.444 | -0.009 | 0.001 | 4.40E-11 | 9.5E-05 | 43.42 |  |
| Left-leg FFM | rs60077625 | | 172098794 | A | G | 0.314 | 0.012 | 0.001 | 2.60E-17 | 1.6E-04 | 71.65 |  |
| Left-leg FFM | rs12123505 | | 203791049 | G | C | 0.719 | -0.008 | 0.001 | 6.70E-09 | 7.4E-05 | 33.63 |  |
| Left-leg FFM | rs823118 | | 205723572 | T | C | 0.548 | -0.013 | 0.001 | 3.80E-24 | 2.3E-04 | 102.77 | BMI |
| Left-leg FFM | rs6684205 | | 218609702 | G | A | 0.287 | 0.014 | 0.001 | 5.90E-23 | 2.1E-04 | 97.31 |  |
| Left-leg FFM | rs13007086 | | 630034 | T | A | 0.828 | 0.037 | 0.002 | 1.20E-103 | 1.0E-03 | 467.38 | BMI |
| Left-leg FFM | rs11695471 | | 25457708 | A | T | 0.331 | -0.010 | 0.001 | 2.50E-12 | 1.1E-04 | 49.03 |  |
| Left-leg FFM | rs2075171 | | 58688907 | A | G | 0.238 | -0.010 | 0.002 | 3.10E-10 | 8.7E-05 | 39.63 | BMI |
| Left-leg FFM | rs6711568 | | 59291172 | G | T | 0.702 | -0.012 | 0.001 | 1.40E-17 | 1.6E-04 | 72.86 | BMI |
| Left-leg FFM | rs752070 | | 74824970 | G | A | 0.126 | 0.012 | 0.002 | 6.90E-10 | 8.4E-05 | 38.04 |  |
| Left-leg FFM | rs1837367 | | 111874551 | A | G | 0.483 | 0.010 | 0.001 | 2.00E-13 | 1.2E-04 | 54.02 |  |
| Left-leg FFM | rs1064213 | | 198950240 | A | G | 0.478 | 0.010 | 0.001 | 6.30E-16 | 1.4E-04 | 65.33 | BMI |
| Left-leg FFM | rs1542224 | | 223963874 | C | T | 0.719 | 0.014 | 0.001 | 4.60E-21 | 1.9E-04 | 88.70 |  |
| Left-leg FFM | rs3116201 | | 233074205 | A | G | 0.098 | -0.015 | 0.002 | 5.30E-12 | 1.0E-04 | 47.57 |  |
| Left-leg FFM | rs2197563 | | 233687080 | A | G | 0.595 | 0.010 | 0.001 | 1.40E-13 | 1.2E-04 | 54.70 |  |
| Left-leg FFM | rs12713004 | | 23896049 | G | A | 0.725 | 0.014 | 0.001 | 3.00E-21 | 2.0E-04 | 89.53 |  |
| Left-leg FFM | rs10202845 | | 42575820 | G | A | 0.113 | -0.016 | 0.002 | 1.70E-14 | 1.3E-04 | 58.88 |  |
| Left-leg FFM | rs10188231 | | 142297493 | G | C | 0.186 | -0.011 | 0.002 | 3.10E-11 | 9.7E-05 | 44.13 | BMI |
| Left-leg FFM | rs77165542 | | 430975 | T | C | 0.035 | -0.067 | 0.004 | 7.40E-79 | 7.8E-04 | 353.47 | BMI |
| Left-leg FFM | rs1374370 | | 85818273 | A | G | 0.305 | 0.009 | 0.001 | 2.40E-10 | 8.8E-05 | 40.09 |  |
| Left-leg FFM | rs11680549 | | 88926348 | C | G | 0.280 | 0.009 | 0.001 | 4.00E-09 | 7.6E-05 | 34.61 |  |
| Left-leg FFM | rs6430010 | | 143972554 | A | T | 0.142 | 0.013 | 0.002 | 6.30E-13 | 1.1E-04 | 51.75 | BMI |
| Left-leg FFM | rs2140046 | | 169706079 | C | T | 0.364 | -0.010 | 0.001 | 3.90E-13 | 1.2E-04 | 52.71 |  |
| Left-leg FFM | rs72885917 | | 172416376 | C | A | 0.247 | -0.020 | 0.002 | 1.00E-40 | 3.9E-04 | 178.56 |  |
| Left-leg FFM | rs6745626 | | 181436641 | T | C | 0.590 | 0.008 | 0.001 | 6.80E-09 | 7.4E-05 | 33.59 | BMI |
| Left-leg FFM | rs10803955 | | 183228114 | G | A | 0.508 | -0.009 | 0.001 | 1.80E-12 | 1.1E-04 | 49.66 |  |
| Left-leg FFM | rs1260326 | | 27730940 | C | T | 0.604 | 0.017 | 0.001 | 2.00E-36 | 3.5E-04 | 158.83 |  |
| Left-leg FFM | rs115179432 | | 33348679 | G | A | 0.072 | -0.018 | 0.003 | 3.00E-13 | 1.2E-04 | 53.20 |  |
| Left-leg FFM | rs56254146 | | 100822430 | A | G | 0.136 | 0.015 | 0.002 | 5.50E-15 | 1.3E-04 | 61.08 | BMI |
| Left-leg FFM | rs1047891 | | 211540507 | A | C | 0.316 | 0.016 | 0.001 | 6.00E-30 | 2.8E-04 | 129.23 | BMI |
| Left-leg FFM | rs13430869 | | 218146818 | T | G | 0.742 | 0.012 | 0.001 | 3.30E-15 | 1.4E-04 | 62.09 |  |
| Left-leg FFM | rs17246129 | | 227259964 | A | G | 0.305 | 0.009 | 0.001 | 3.50E-10 | 8.7E-05 | 39.39 |  |
| Left-leg FFM | rs4663211 | | 236756688 | G | T | 0.766 | -0.010 | 0.002 | 2.90E-10 | 8.7E-05 | 39.73 | BMI |
| Left-leg FFM | rs9295 | | 36777825 | A | G | 0.296 | 0.013 | 0.001 | 1.70E-19 | 1.8E-04 | 81.60 |  |
| Left-leg FFM | rs59985551 | | 56106928 | T | C | 0.226 | -0.019 | 0.002 | 3.90E-34 | 3.3E-04 | 148.41 |  |
| Left-leg FFM | rs11545482 | | 70315987 | T | C | 0.020 | -0.029 | 0.005 | 2.50E-10 | 8.8E-05 | 40.00 |  |
| Left-leg FFM | rs4852777 | | 71534161 | C | G | 0.595 | -0.010 | 0.001 | 1.20E-14 | 1.3E-04 | 59.56 |  |
| Left-leg FFM | rs58584712 | | 112249583 | A | G | 0.211 | 0.011 | 0.002 | 7.10E-12 | 1.0E-04 | 47.00 |  |
| Left-leg FFM | rs17443541 | | 200402624 | C | T | 0.189 | -0.010 | 0.002 | 3.20E-09 | 7.7E-05 | 35.06 |  |
| Left-leg FFM | rs1447498 | | 205388729 | G | A | 0.770 | -0.010 | 0.002 | 7.60E-10 | 8.3E-05 | 37.86 | BMI |
| Left-leg FFM | rs1478575 | | 218278555 | A | T | 0.684 | 0.017 | 0.001 | 1.80E-33 | 3.2E-04 | 145.32 |  |
| Left-leg FFM | rs7601000 | | 242610773 | A | T | 0.767 | -0.013 | 0.002 | 3.80E-16 | 1.5E-04 | 66.34 |  |
| Left-leg FFM | rs2270894 | | 9975386 | G | C | 0.203 | -0.017 | 0.002 | 8.60E-25 | 2.3E-04 | 105.69 |  |
| Left-leg FFM | rs2569993 | | 12926096 | C | T | 0.320 | 0.009 | 0.001 | 1.80E-11 | 9.9E-05 | 45.14 | BMI |
| Left-leg FFM | rs7619139 | | 25110415 | A | T | 0.589 | 0.013 | 0.001 | 2.00E-23 | 2.2E-04 | 99.49 | BMI |
| Left-leg FFM | rs117206167 | | 33260151 | T | C | 0.066 | -0.018 | 0.003 | 4.10E-12 | 1.1E-04 | 48.08 |  |
| Left-leg FFM | rs2735559 | | 88106618 | A | G | 0.114 | -0.017 | 0.002 | 3.00E-17 | 1.6E-04 | 71.33 | BMI |
| Left-leg FFM | rs2016469 | | 108023965 | A | G | 0.372 | 0.008 | 0.001 | 5.60E-10 | 8.5E-05 | 38.47 | BMI |
| Left-leg FFM | rs7624428 | | 131516596 | A | T | 0.269 | 0.013 | 0.001 | 2.00E-17 | 1.6E-04 | 72.18 | BMI |
| Left-leg FFM | rs724016 | | 141105570 | G | A | 0.444 | 0.028 | 0.001 | 1.40E-101 | 1.0E-03 | 457.87 |  |
| Left-leg FFM | rs5396 | | 170744815 | C | T | 0.278 | 0.010 | 0.001 | 2.60E-12 | 1.1E-04 | 48.98 | BMI |
| Left-leg FFM | rs62246311 | | 9498143 | A | G | 0.102 | 0.015 | 0.002 | 1.30E-12 | 1.1E-04 | 50.39 | BMI |
| Left-leg FFM | rs9843653 | | 49920571 | C | T | 0.512 | 0.014 | 0.001 | 6.30E-28 | 2.6E-04 | 120.01 | BMI |
| Left-leg FFM | rs56038322 | | 69925128 | A | G | 0.311 | 0.008 | 0.001 | 3.40E-08 | 6.7E-05 | 30.45 | BMI |
| Left-leg FFM | rs6779752 | | 85663849 | A | G | 0.638 | -0.012 | 0.001 | 1.00E-17 | 1.6E-04 | 73.46 | BMI |
| Left-leg FFM | rs34693680 | | 98665549 | T | C | 0.132 | 0.013 | 0.002 | 1.60E-11 | 1.0E-04 | 45.37 |  |
| Left-leg FFM | rs1910466 | | 147086268 | C | T | 0.498 | -0.008 | 0.001 | 2.70E-09 | 7.8E-05 | 35.41 |  |
| Left-leg FFM | rs357486 | | 153885503 | C | T | 0.543 | 0.011 | 0.001 | 9.40E-18 | 1.6E-04 | 73.63 | BMI |
| Left-leg FFM | rs73175572 | | 185490184 | G | A | 0.112 | 0.026 | 0.002 | 6.40E-37 | 3.5E-04 | 161.12 |  |
| Left-leg FFM | rs73052033 | | 185828465 | C | T | 0.185 | -0.017 | 0.002 | 3.40E-24 | 2.3E-04 | 102.97 | BMI |
| Left-leg FFM | rs6772164 | | 196078149 | A | C | 0.358 | 0.009 | 0.001 | 7.20E-11 | 9.3E-05 | 42.46 | BMI |
| Left-leg FFM | rs1911073 | | 30051533 | G | A | 0.347 | 0.008 | 0.001 | 1.90E-09 | 7.9E-05 | 36.04 |  |
| Left-leg FFM | rs13059004 | | 38601237 | C | A | 0.478 | 0.007 | 0.001 | 2.00E-08 | 6.9E-05 | 31.47 |  |
| Left-leg FFM | rs754635 | | 42305131 | G | C | 0.887 | 0.014 | 0.002 | 1.20E-11 | 1.0E-04 | 46.05 | BMI |
| Left-leg FFM | rs6762851 | | 56686329 | C | T | 0.357 | -0.011 | 0.001 | 9.80E-16 | 1.4E-04 | 64.48 |  |
| Left-leg FFM | rs4635681 | | 152310614 | G | A | 0.156 | 0.011 | 0.002 | 1.20E-09 | 8.1E-05 | 36.96 |  |
| Left-leg FFM | rs36012032 | | 52814709 | A | C | 0.091 | 0.018 | 0.002 | 6.40E-16 | 1.4E-04 | 65.32 |  |
| Left-leg FFM | rs7641177 | | 61183158 | C | A | 0.374 | -0.009 | 0.001 | 1.10E-10 | 9.1E-05 | 41.59 | BMI |
| Left-leg FFM | rs6766472 | | 80651423 | C | A | 0.297 | 0.010 | 0.001 | 3.80E-12 | 1.1E-04 | 48.20 |  |
| Left-leg FFM | rs12631813 | | 171126134 | G | C | 0.503 | -0.008 | 0.001 | 4.10E-09 | 7.6E-05 | 34.58 |  |
| Left-leg FFM | rs7652177 | | 171969077 | G | C | 0.505 | 0.013 | 0.001 | 2.70E-23 | 2.2E-04 | 98.85 |  |
| Left-leg FFM | rs512692 | | 172170842 | T | A | 0.285 | 0.013 | 0.001 | 3.60E-20 | 1.9E-04 | 84.61 |  |
| Left-leg FFM | rs4974072 | | 41222586 | A | G | 0.490 | 0.011 | 0.001 | 1.00E-17 | 1.6E-04 | 73.42 |  |
| Left-leg FFM | rs2306272 | | 66434643 | C | T | 0.287 | 0.008 | 0.001 | 6.70E-09 | 7.4E-05 | 33.62 | BMI |
| Left-leg FFM | rs76267866 | | 70540347 | T | A | 0.206 | 0.010 | 0.002 | 1.30E-09 | 8.1E-05 | 36.82 | BMI |
| Left-leg FFM | rs6762578 | | 128992047 | A | G | 0.778 | 0.015 | 0.002 | 1.30E-20 | 1.9E-04 | 86.65 |  |
| Left-leg FFM | rs843374 | | 183997261 | T | A | 0.587 | -0.012 | 0.001 | 2.30E-20 | 1.9E-04 | 85.52 |  |
| Left-leg FFM | rs10488836 | | 2160894 | T | C | 0.053 | -0.016 | 0.003 | 4.00E-08 | 6.6E-05 | 30.14 |  |
| Left-leg FFM | rs4527444 | | 30842780 | G | A | 0.541 | 0.009 | 0.001 | 2.30E-12 | 1.1E-04 | 49.17 | BMI |
| Left-leg FFM | rs34028346 | | 54368694 | A | G | 0.142 | 0.012 | 0.002 | 4.20E-10 | 8.6E-05 | 39.04 |  |
| Left-leg FFM | rs34772064 | | 55495948 | G | T | 0.556 | -0.008 | 0.001 | 3.20E-09 | 7.7E-05 | 35.06 | BMI |
| Left-leg FFM | rs1443536 | | 82174165 | G | A | 0.305 | 0.011 | 0.001 | 2.20E-15 | 1.4E-04 | 62.84 |  |
| Left-leg FFM | rs11097755 | | 102709308 | C | T | 0.443 | 0.010 | 0.001 | 2.50E-13 | 1.2E-04 | 53.57 | BMI |
| Left-leg FFM | rs6840229 | | 115064611 | A | G | 0.438 | -0.008 | 0.001 | 2.30E-10 | 8.8E-05 | 40.16 | BMI |
| Left-leg FFM | rs4240326 | | 145839264 | G | A | 0.550 | -0.020 | 0.001 | 9.80E-55 | 5.3E-04 | 242.77 |  |
| Left-leg FFM | rs34627176 | | 1006200 | A | G | 0.214 | -0.012 | 0.002 | 8.80E-14 | 1.2E-04 | 55.63 |  |
| Left-leg FFM | rs1477890 | | 18511738 | G | A | 0.493 | 0.010 | 0.001 | 9.00E-14 | 1.2E-04 | 55.58 | BMI |
| Left-leg FFM | rs2231142 | | 89052323 | T | G | 0.113 | -0.015 | 0.002 | 6.40E-13 | 1.1E-04 | 51.73 | BMI |
| Left-leg FFM | rs34848742 | | 123828042 | G | T | 0.788 | -0.014 | 0.002 | 1.50E-18 | 1.7E-04 | 77.30 |  |
| Left-leg FFM | rs139919928 | | 134724499 | A | G | 0.027 | 0.023 | 0.004 | 1.20E-08 | 7.1E-05 | 32.52 |  |
| Left-leg FFM | rs10938398 | | 45186139 | A | G | 0.434 | 0.015 | 0.001 | 8.40E-30 | 2.8E-04 | 128.57 | BMI |
| Left-leg FFM | rs6821305 | | 122713863 | C | A | 0.399 | 0.013 | 0.001 | 5.30E-22 | 2.0E-04 | 92.98 |  |
| Left-leg FFM | rs55920177 | | 171609715 | T | A | 0.129 | -0.011 | 0.002 | 9.70E-09 | 7.2E-05 | 32.91 | BMI |
| Left-leg FFM | rs2858019 | | 3493345 | G | A | 0.275 | -0.008 | 0.001 | 9.10E-09 | 7.3E-05 | 33.03 |  |
| Left-leg FFM | rs2102278 | | 52818664 | G | A | 0.323 | 0.011 | 0.001 | 3.40E-14 | 1.3E-04 | 57.47 | BMI |
| Left-leg FFM | rs2647268 | | 106211443 | G | A | 0.384 | -0.015 | 0.001 | 2.40E-30 | 2.9E-04 | 131.04 |  |
| Left-leg FFM | rs1296328 | | 137083193 | C | A | 0.559 | -0.011 | 0.001 | 6.90E-16 | 1.4E-04 | 65.16 | BMI |
| Left-leg FFM | rs395980 | | 177430072 | G | T | 0.263 | -0.009 | 0.001 | 2.40E-10 | 8.8E-05 | 40.07 |  |
| Left-leg FFM | rs7680647 | | 1750487 | C | T | 0.356 | 0.011 | 0.001 | 3.10E-15 | 1.4E-04 | 62.21 |  |
| Left-leg FFM | rs7671110 | | 17874089 | T | C | 0.158 | -0.027 | 0.002 | 1.10E-50 | 4.9E-04 | 224.11 |  |
| Left-leg FFM | rs11945720 | | 28247163 | A | G | 0.839 | 0.010 | 0.002 | 4.70E-08 | 6.6E-05 | 29.84 |  |
| Left-leg FFM | rs17010957 | | 86719165 | C | T | 0.148 | -0.013 | 0.002 | 1.20E-11 | 1.0E-04 | 46.01 |  |
| Left-leg FFM | rs72659403 | | 90825002 | G | A | 0.078 | -0.013 | 0.002 | 3.40E-08 | 6.7E-05 | 30.46 |  |
| Left-leg FFM | rs3990738 | | 152295198 | G | A | 0.543 | -0.008 | 0.001 | 3.70E-09 | 7.6E-05 | 34.76 |  |
| Left-leg FFM | rs10058393 | | 277617 | T | C | 0.129 | 0.012 | 0.002 | 1.20E-10 | 9.1E-05 | 41.52 |  |
| Left-leg FFM | rs12519532 | | 42829303 | A | G | 0.283 | 0.013 | 0.001 | 1.70E-20 | 1.9E-04 | 86.12 |  |
| Left-leg FFM | rs2578490 | | 92389236 | G | A | 0.642 | 0.008 | 0.001 | 7.00E-10 | 8.4E-05 | 38.03 | BMI |
| Left-leg FFM | rs62365719 | | 95862694 | C | T | 0.398 | -0.009 | 0.001 | 1.70E-11 | 1.0E-04 | 45.30 | BMI |
| Left-leg FFM | rs2952615 | | 112138888 | C | G | 0.618 | -0.011 | 0.001 | 1.20E-17 | 1.6E-04 | 73.15 |  |
| Left-leg FFM | rs62378779 | | 122649290 | T | C | 0.300 | -0.012 | 0.001 | 1.30E-15 | 1.4E-04 | 63.98 |  |
| Left-leg FFM | rs153758 | | 171209065 | C | G | 0.431 | -0.007 | 0.001 | 1.30E-08 | 7.1E-05 | 32.28 |  |
| Left-leg FFM | rs252937 | | 5497900 | G | C | 0.662 | -0.010 | 0.001 | 2.70E-12 | 1.1E-04 | 48.87 |  |
| Left-leg FFM | rs695922 | | 53385529 | G | A | 0.837 | -0.011 | 0.002 | 2.20E-09 | 7.9E-05 | 35.79 |  |
| Left-leg FFM | rs505575 | | 111256812 | C | T | 0.673 | -0.009 | 0.001 | 1.60E-11 | 1.0E-04 | 45.37 |  |
| Left-leg FFM | rs1835009 | | 122661217 | T | C | 0.236 | -0.015 | 0.002 | 1.10E-23 | 2.2E-04 | 100.61 | BMI |
| Left-leg FFM | rs9327336 | | 123990270 | C | T | 0.343 | 0.008 | 0.001 | 1.90E-08 | 7.0E-05 | 31.62 |  |
| Left-leg FFM | rs6860245 | | 127367998 | C | G | 0.248 | 0.025 | 0.002 | 1.10E-59 | 5.8E-04 | 265.49 |  |
| Left-leg FFM | rs157577 | | 131563571 | C | G | 0.722 | 0.015 | 0.001 | 2.20E-25 | 2.4E-04 | 108.37 |  |
| Left-leg FFM | rs7701443 | | 142792650 | G | A | 0.411 | -0.008 | 0.001 | 1.20E-08 | 7.1E-05 | 32.43 |  |
| Left-leg FFM | rs4282339 | | 168256240 | A | G | 0.208 | -0.016 | 0.002 | 1.10E-23 | 2.2E-04 | 100.60 |  |
| Left-leg FFM | rs32736 | | 3462896 | G | T | 0.348 | 0.008 | 0.001 | 3.50E-08 | 6.7E-05 | 30.40 |  |
| Left-leg FFM | rs6450346 | | 55014771 | C | T | 0.701 | -0.009 | 0.001 | 7.80E-11 | 9.3E-05 | 42.30 |  |
| Left-leg FFM | rs6888717 | | 158363581 | A | G | 0.811 | -0.009 | 0.002 | 2.00E-08 | 6.9E-05 | 31.52 |  |
| Left-leg FFM | rs2422054 | | 161382474 | A | T | 0.631 | 0.008 | 0.001 | 4.00E-09 | 7.6E-05 | 34.61 |  |
| Left-leg FFM | rs6874142 | | 172753555 | G | T | 0.114 | 0.017 | 0.002 | 1.90E-14 | 1.3E-04 | 58.63 |  |
| Left-leg FFM | rs13180309 | | 36790001 | G | A | 0.445 | -0.014 | 0.001 | 1.50E-25 | 2.4E-04 | 109.18 |  |
| Left-leg FFM | rs840812 | | 86970418 | T | C | 0.020 | 0.027 | 0.005 | 6.90E-09 | 7.4E-05 | 33.55 | BMI |
| Left-leg FFM | rs141729694 | | 87999371 | T | C | 0.074 | 0.023 | 0.002 | 8.30E-21 | 1.9E-04 | 87.53 | BMI |
| Left-leg FFM | rs33967909 | | 137603293 | A | G | 0.216 | 0.013 | 0.002 | 6.80E-16 | 1.4E-04 | 65.19 |  |
| Left-leg FFM | rs17115481 | | 153358226 | A | G | 0.270 | -0.009 | 0.001 | 7.00E-10 | 8.4E-05 | 38.01 | BMI |
| Left-leg FFM | rs446382 | | 176517461 | G | T | 0.726 | 0.010 | 0.001 | 2.50E-12 | 1.1E-04 | 49.01 |  |
| Left-leg FFM | rs7706886 | | 32722319 | A | G | 0.271 | 0.011 | 0.001 | 2.20E-14 | 1.3E-04 | 58.39 |  |
| Left-leg FFM | rs7709645 | | 60731458 | C | G | 0.493 | -0.011 | 0.001 | 4.90E-17 | 1.5E-04 | 70.38 |  |
| Left-leg FFM | rs11738728 | | 64238731 | A | G | 0.358 | -0.008 | 0.001 | 2.60E-08 | 6.8E-05 | 31.02 |  |
| Left-leg FFM | rs6873192 | | 67598184 | G | A | 0.517 | -0.011 | 0.001 | 2.50E-16 | 1.5E-04 | 67.14 |  |
| Left-leg FFM | rs2307111 | | 75003678 | C | T | 0.395 | -0.018 | 0.001 | 3.30E-42 | 4.1E-04 | 185.36 | BMI |
| Left-leg FFM | rs365352 | | 77401152 | A | G | 0.244 | -0.016 | 0.002 | 3.90E-26 | 2.5E-04 | 111.82 | BMI |
| Left-leg FFM | rs6235 | | 95728898 | G | C | 0.268 | 0.016 | 0.001 | 2.20E-28 | 2.7E-04 | 122.06 | BMI |
| Left-leg FFM | rs3822742 | | 139059017 | A | C | 0.371 | 0.015 | 0.001 | 4.60E-27 | 2.6E-04 | 116.05 | BMI |
| Left-leg FFM | rs59738707 | | 141814420 | A | G | 0.128 | -0.011 | 0.002 | 2.70E-08 | 6.8E-05 | 30.95 |  |
| Left-leg FFM | rs4073717 | | 170864021 | T | G | 0.202 | -0.016 | 0.002 | 1.80E-22 | 2.1E-04 | 95.09 |  |
| Left-leg FFM | rs55758152 | | 171317318 | A | G | 0.326 | 0.008 | 0.001 | 2.00E-08 | 6.9E-05 | 31.45 |  |
| Left-leg FFM | rs11243202 | | 7719065 | C | T | 0.486 | 0.015 | 0.001 | 1.60E-30 | 2.9E-04 | 131.84 |  |
| Left-leg FFM | rs62396185 | | 26180634 | C | G | 0.260 | -0.022 | 0.001 | 2.00E-48 | 4.7E-04 | 213.87 | BMI |
| Left-leg FFM | rs9344126 | | 81907559 | C | T | 0.514 | -0.009 | 0.001 | 6.20E-12 | 1.0E-04 | 47.26 |  |
| Left-leg FFM | rs1369869 | | 83366282 | T | C | 0.439 | 0.009 | 0.001 | 6.40E-11 | 9.4E-05 | 42.70 | BMI |
| Left-leg FFM | rs6570509 | | 142716286 | T | G | 0.287 | -0.013 | 0.001 | 2.00E-18 | 1.7E-04 | 76.65 |  |
| Left-leg FFM | rs394487 | | 160778639 | T | C | 0.278 | 0.010 | 0.001 | 1.20E-12 | 1.1E-04 | 50.41 | BMI |
| Left-leg FFM | rs12199246 | | 41999809 | A | G | 0.087 | 0.013 | 0.002 | 4.40E-08 | 6.6E-05 | 29.98 |  |
| Left-leg FFM | rs10457469 | | 126083658 | A | G | 0.523 | 0.013 | 0.001 | 5.90E-25 | 2.3E-04 | 106.44 | BMI |
| Left-leg FFM | rs7740107 | | 130374461 | A | T | 0.736 | -0.026 | 0.001 | 3.30E-67 | 6.6E-04 | 299.99 |  |
| Left-leg FFM | rs62425398 | | 166416028 | A | C | 0.106 | 0.014 | 0.002 | 2.70E-11 | 9.8E-05 | 44.35 |  |
| Left-leg FFM | rs76307059 | | 169000888 | G | C | 0.046 | -0.018 | 0.003 | 1.30E-08 | 7.1E-05 | 32.28 |  |
| Left-leg FFM | rs12213070 | | 12131542 | A | G | 0.346 | -0.009 | 0.001 | 7.50E-12 | 1.0E-04 | 46.90 |  |
| Left-leg FFM | rs41271299 | | 19839415 | T | C | 0.051 | 0.036 | 0.003 | 8.80E-34 | 3.2E-04 | 146.76 | BMI |
| Left-leg FFM | rs16891847 | | 39260119 | C | T | 0.185 | 0.009 | 0.002 | 3.20E-08 | 6.7E-05 | 30.58 |  |
| Left-leg FFM | rs1266876 | | 51781580 | T | C | 0.350 | 0.010 | 0.001 | 7.40E-14 | 1.2E-04 | 55.97 | BMI |
| Left-leg FFM | rs3805885 | | 80996751 | C | G | 0.455 | -0.011 | 0.001 | 1.90E-17 | 1.6E-04 | 72.20 |  |
| Left-leg FFM | rs3736984 | | 90408589 | G | A | 0.172 | 0.011 | 0.002 | 2.10E-10 | 8.9E-05 | 40.40 |  |
| Left-leg FFM | rs9391253 | | 105367616 | T | A | 0.320 | 0.011 | 0.001 | 1.20E-15 | 1.4E-04 | 64.09 |  |
| Left-leg FFM | rs11756675 | | 120067932 | G | A | 0.257 | 0.008 | 0.001 | 4.80E-08 | 6.6E-05 | 29.81 |  |
| Left-leg FFM | rs687694 | | 153472227 | G | T | 0.495 | -0.008 | 0.001 | 1.20E-09 | 8.1E-05 | 37.02 |  |
| Left-leg FFM | rs4235910 | | 169344450 | C | A | 0.515 | 0.008 | 0.001 | 1.50E-09 | 8.0E-05 | 36.57 |  |
| Left-leg FFM | rs3129962 | | 32379383 | A | G | 0.129 | 0.024 | 0.002 | 1.20E-34 | 3.3E-04 | 150.68 |  |
| Left-leg FFM | rs9272554 | | 32607026 | G | A | 0.227 | 0.009 | 0.002 | 2.30E-09 | 7.9E-05 | 35.71 |  |
| Left-leg FFM | rs114056237 | | 41877671 | A | G | 0.012 | -0.063 | 0.006 | 2.40E-27 | 2.6E-04 | 117.38 |  |
| Left-leg FFM | rs1418433 | | 44752568 | A | G | 0.588 | -0.008 | 0.001 | 1.30E-09 | 8.1E-05 | 36.75 |  |
| Left-leg FFM | rs9388498 | | 126873423 | T | G | 0.186 | 0.014 | 0.002 | 5.30E-17 | 1.5E-04 | 70.21 |  |
| Left-leg FFM | rs599004 | | 140439740 | T | C | 0.281 | -0.009 | 0.001 | 2.40E-10 | 8.8E-05 | 40.08 |  |
| Left-leg FFM | rs3853252 | | 152170247 | A | G | 0.455 | 0.015 | 0.001 | 8.30E-29 | 2.7E-04 | 124.02 |  |
| Left-leg FFM | rs12216497 | | 19028623 | T | C | 0.561 | -0.010 | 0.001 | 3.00E-13 | 1.2E-04 | 53.19 |  |
| Left-leg FFM | rs3131014 | | 31116627 | A | G | 0.236 | -0.018 | 0.002 | 9.00E-31 | 2.9E-04 | 133.00 |  |
| Left-leg FFM | rs2744956 | | 34618937 | C | T | 0.139 | 0.039 | 0.002 | 6.90E-96 | 9.5E-04 | 431.71 | BMI |
| Left-leg FFM | rs4715207 | | 50809278 | T | C | 0.180 | 0.023 | 0.002 | 1.40E-42 | 4.1E-04 | 187.03 | BMI |
| Left-leg FFM | rs79589376 | | 90294244 | T | A | 0.127 | 0.011 | 0.002 | 4.70E-08 | 6.6E-05 | 29.83 |  |
| Left-leg FFM | rs17789218 | | 100600097 | C | T | 0.245 | 0.011 | 0.002 | 2.80E-12 | 1.1E-04 | 48.83 |  |
| Left-leg FFM | rs9398171 | | 108983527 | T | C | 0.711 | 0.018 | 0.001 | 2.30E-37 | 3.6E-04 | 163.18 | BMI |
| Left-leg FFM | rs11153171 | | 109653825 | T | C | 0.356 | -0.013 | 0.001 | 3.80E-21 | 2.0E-04 | 89.07 |  |
| Left-leg FFM | rs7755185 | | 152339615 | G | A | 0.311 | 0.009 | 0.001 | 1.20E-09 | 8.1E-05 | 36.92 |  |
| Left-leg FFM | rs37964 | | 8004981 | T | A | 0.466 | 0.008 | 0.001 | 5.00E-09 | 7.5E-05 | 34.20 |  |
| Left-leg FFM | rs11761240 | | 67647531 | C | T | 0.313 | -0.008 | 0.001 | 1.20E-08 | 7.1E-05 | 32.43 |  |
| Left-leg FFM | rs2866719 | | 70106061 | T | C | 0.369 | 0.009 | 0.001 | 6.90E-12 | 1.0E-04 | 47.06 | BMI |
| Left-leg FFM | rs10953083 | | 92657034 | A | C | 0.446 | 0.008 | 0.001 | 9.10E-09 | 7.3E-05 | 33.03 |  |
| Left-leg FFM | rs10256522 | | 139734373 | T | C | 0.375 | 0.007 | 0.001 | 3.00E-08 | 6.8E-05 | 30.72 |  |
| Left-leg FFM | rs34776209 | | 23513093 | T | C | 0.247 | -0.015 | 0.002 | 1.70E-23 | 2.2E-04 | 99.78 |  |
| Left-leg FFM | rs56304870 | | 44863439 | A | T | 0.070 | 0.014 | 0.003 | 1.30E-08 | 7.1E-05 | 32.30 |  |
| Left-leg FFM | rs3823674 | | 50571996 | T | C | 0.428 | -0.011 | 0.001 | 5.90E-16 | 1.4E-04 | 65.49 | BMI |
| Left-leg FFM | rs16868443 | | 93206264 | C | G | 0.360 | 0.013 | 0.001 | 2.80E-21 | 2.0E-04 | 89.69 | BMI |
| Left-leg FFM | rs2396625 | | 113028634 | A | T | 0.421 | -0.011 | 0.001 | 1.90E-15 | 1.4E-04 | 63.12 | BMI |
| Left-leg FFM | rs62621812 | | 127015083 | A | G | 0.020 | 0.041 | 0.005 | 9.10E-18 | 1.6E-04 | 73.71 | BMI |
| Left-leg FFM | rs111636812 | | 148364845 | G | T | 0.224 | -0.009 | 0.002 | 1.10E-08 | 7.2E-05 | 32.59 |  |
| Left-leg FFM | rs822549 | | 148649180 | C | T | 0.267 | 0.010 | 0.001 | 2.10E-12 | 1.1E-04 | 49.40 |  |
| Left-leg FFM | rs6946415 | | 150684548 | G | A | 0.627 | 0.018 | 0.001 | 2.00E-42 | 4.1E-04 | 186.33 | BMI |
| Left-leg FFM | rs10899768 | | 55824205 | G | A | 0.164 | -0.010 | 0.002 | 1.70E-08 | 7.0E-05 | 31.83 |  |
| Left-leg FFM | rs13244614 | | 72973854 | A | C | 0.283 | 0.010 | 0.001 | 3.10E-12 | 1.1E-04 | 48.61 |  |
| Left-leg FFM | rs4729099 | | 76638755 | A | C | 0.826 | -0.017 | 0.002 | 4.80E-24 | 2.2E-04 | 102.27 |  |
| Left-leg FFM | rs10269774 | | 92253972 | A | G | 0.326 | 0.023 | 0.001 | 1.40E-63 | 6.2E-04 | 283.32 |  |
| Left-leg FFM | rs114949263 | | 150498245 | C | T | 0.112 | -0.013 | 0.002 | 5.00E-10 | 8.5E-05 | 38.68 |  |
| Left-leg FFM | rs2533879 | | 2859847 | A | G | 0.300 | -0.022 | 0.001 | 2.00E-53 | 5.2E-04 | 236.79 |  |
| Left-leg FFM | rs520161 | | 28210660 | C | T | 0.703 | -0.009 | 0.001 | 5.70E-10 | 8.4E-05 | 38.42 |  |
| Left-leg FFM | rs12700901 | | 28783171 | A | C | 0.406 | -0.008 | 0.001 | 2.80E-09 | 7.8E-05 | 35.30 |  |
| Left-leg FFM | rs77760034 | | 46606906 | A | T | 0.075 | -0.017 | 0.003 | 7.80E-12 | 1.0E-04 | 46.81 |  |
| Left-leg FFM | rs2881198 | | 46634506 | C | G | 0.530 | -0.012 | 0.001 | 5.30E-19 | 1.7E-04 | 79.32 | BMI |
| Left-leg FFM | rs1443749 | | 121960438 | T | C | 0.366 | 0.012 | 0.001 | 4.10E-18 | 1.7E-04 | 75.26 | BMI |
| Left-leg FFM | rs836511 | | 6458319 | G | A | 0.199 | 0.011 | 0.002 | 2.40E-11 | 9.8E-05 | 44.61 |  |
| Left-leg FFM | rs2740761 | | 55255986 | T | C | 0.208 | 0.009 | 0.002 | 4.50E-08 | 6.6E-05 | 29.92 |  |
| Left-leg FFM | rs56383938 | | 74097622 | G | A | 0.087 | -0.016 | 0.002 | 8.80E-13 | 1.1E-04 | 51.10 | BMI |
| Left-leg FFM | rs1910252 | | 49407362 | T | C | 0.168 | 0.013 | 0.002 | 1.20E-13 | 1.2E-04 | 55.09 |  |
| Left-leg FFM | rs72656010 | | 57122215 | C | T | 0.132 | -0.033 | 0.002 | 6.00E-65 | 6.4E-04 | 289.63 |  |
| Left-leg FFM | rs61729527 | | 77761919 | T | C | 0.052 | -0.023 | 0.003 | 1.60E-15 | 1.4E-04 | 63.55 | BMI |
| Left-leg FFM | rs7815955 | | 130719567 | T | A | 0.203 | -0.013 | 0.002 | 7.90E-16 | 1.4E-04 | 64.89 |  |
| Left-leg FFM | rs2277138 | | 135622640 | C | T | 0.394 | -0.017 | 0.001 | 3.10E-36 | 3.5E-04 | 157.99 |  |
| Left-leg FFM | rs13264909 | | 64702385 | T | A | 0.429 | -0.009 | 0.001 | 1.30E-12 | 1.1E-04 | 50.33 | BMI |
| Left-leg FFM | rs11782103 | | 120451323 | A | T | 0.252 | -0.012 | 0.001 | 1.30E-14 | 1.3E-04 | 59.32 |  |
| Left-leg FFM | rs11777007 | | 10801857 | T | C | 0.547 | -0.011 | 0.001 | 4.50E-18 | 1.7E-04 | 75.09 | BMI |
| Left-leg FFM | rs13282247 | | 19625464 | T | C | 0.305 | -0.008 | 0.001 | 6.80E-09 | 7.4E-05 | 33.58 |  |
| Left-leg FFM | rs10958683 | | 38274193 | G | C | 0.229 | -0.011 | 0.002 | 7.40E-12 | 1.0E-04 | 46.92 |  |
| Left-leg FFM | rs4735766 | | 78099782 | T | G | 0.285 | 0.017 | 0.001 | 1.10E-32 | 3.1E-04 | 141.83 |  |
| Left-leg FFM | rs16916881 | | 95566270 | A | C | 0.235 | -0.010 | 0.002 | 2.40E-10 | 8.8E-05 | 40.10 | BMI |
| Left-leg FFM | rs112875651 | | 126506694 | A | G | 0.391 | 0.014 | 0.001 | 1.20E-26 | 2.5E-04 | 114.22 | BMI |
| Left-leg FFM | rs2280940 | | 23159320 | T | C | 0.690 | 0.009 | 0.001 | 3.20E-11 | 9.7E-05 | 44.07 |  |
| Left-leg FFM | rs62515438 | | 57161608 | G | T | 0.228 | 0.017 | 0.002 | 1.90E-28 | 2.7E-04 | 122.38 |  |
| Left-leg FFM | rs1431663 | | 73460688 | C | A | 0.572 | -0.010 | 0.001 | 4.60E-14 | 1.3E-04 | 56.89 |  |
| Left-leg FFM | rs7832926 | | 23355620 | C | A | 0.326 | 0.008 | 0.001 | 8.10E-09 | 7.3E-05 | 33.25 |  |
| Left-leg FFM | rs1982441 | | 28021769 | T | G | 0.130 | 0.012 | 0.002 | 9.90E-10 | 8.2E-05 | 37.34 | BMI |
| Left-leg FFM | rs1504797 | | 89434405 | C | T | 0.306 | -0.012 | 0.001 | 4.50E-16 | 1.5E-04 | 65.99 | BMI |
| Left-leg FFM | rs2737218 | | 116631278 | C | T | 0.208 | -0.016 | 0.002 | 1.80E-24 | 2.3E-04 | 104.23 | BMI |
| Left-leg FFM | rs117169657 | | 145508782 | T | C | 0.136 | 0.012 | 0.002 | 3.10E-10 | 8.7E-05 | 39.60 |  |
| Left-leg FFM | rs7042372 | | 6959840 | G | A | 0.335 | -0.009 | 0.001 | 6.80E-10 | 8.4E-05 | 38.08 | BMI |
| Left-leg FFM | rs11794152 | | 23345347 | G | A | 0.415 | 0.011 | 0.001 | 3.40E-16 | 1.5E-04 | 66.57 |  |
| Left-leg FFM | rs1243873 | | 35686595 | T | C | 0.462 | -0.009 | 0.001 | 2.00E-11 | 9.9E-05 | 44.93 |  |
| Left-leg FFM | rs10780905 | | 90841066 | A | G | 0.588 | -0.008 | 0.001 | 2.30E-10 | 8.8E-05 | 40.22 |  |
| Left-leg FFM | rs28457693 | | 98217348 | G | A | 0.107 | 0.025 | 0.002 | 6.10E-32 | 3.0E-04 | 138.36 |  |
| Left-leg FFM | rs11516134 | | 140282285 | C | T | 0.221 | -0.011 | 0.002 | 5.00E-12 | 1.0E-04 | 47.69 |  |
| Left-leg FFM | rs12001437 | | 34074476 | C | T | 0.368 | 0.007 | 0.001 | 3.60E-08 | 6.7E-05 | 30.37 | BMI |
| Left-leg FFM | rs1308512 | | 87279298 | G | A | 0.805 | 0.010 | 0.002 | 1.30E-09 | 8.1E-05 | 36.79 |  |
| Left-leg FFM | rs111821073 | | 99084793 | T | C | 0.157 | 0.016 | 0.002 | 5.10E-18 | 1.6E-04 | 74.86 |  |
| Left-leg FFM | rs72733810 | | 73797181 | G | A | 0.202 | 0.010 | 0.002 | 2.30E-09 | 7.8E-05 | 35.66 |  |
| Left-leg FFM | rs2482357 | | 94179978 | A | G | 0.432 | -0.010 | 0.001 | 2.30E-15 | 1.4E-04 | 62.78 | BMI |
| Left-leg FFM | rs7033487 | | 119129257 | C | T | 0.198 | -0.021 | 0.002 | 2.10E-38 | 3.7E-04 | 167.97 |  |
| Left-leg FFM | rs35990522 | | 119274481 | A | T | 0.075 | 0.016 | 0.002 | 1.30E-10 | 9.1E-05 | 41.29 |  |
| Left-leg FFM | rs1147345 | | 132212511 | T | A | 0.526 | -0.007 | 0.001 | 2.90E-08 | 6.8E-05 | 30.75 |  |
| Left-leg FFM | rs1412234 | | 28410683 | C | T | 0.327 | 0.013 | 0.001 | 2.20E-20 | 1.9E-04 | 85.59 | BMI |
| Left-leg FFM | rs12238669 | | 128011403 | G | A | 0.469 | -0.010 | 0.001 | 1.60E-14 | 1.3E-04 | 58.99 | BMI |
| Left-leg FFM | rs1330199 | | 27760946 | T | G | 0.483 | -0.008 | 0.001 | 6.80E-09 | 7.4E-05 | 33.60 | BMI |
| Left-leg FFM | rs10979612 | | 111687379 | C | T | 0.073 | 0.018 | 0.003 | 1.70E-13 | 1.2E-04 | 54.36 |  |
| Left-leg FFM | rs7080472 | | 96012950 | T | G | 0.425 | 0.012 | 0.001 | 5.00E-19 | 1.7E-04 | 79.42 |  |
| Left-leg FFM | rs10886477 | | 121175524 | A | G | 0.128 | 0.012 | 0.002 | 6.80E-10 | 8.4E-05 | 38.08 |  |
| Left-leg FFM | rs12764498 | | 5042294 | C | T | 0.118 | -0.013 | 0.002 | 6.50E-11 | 9.4E-05 | 42.68 |  |
| Left-leg FFM | rs73601548 | | 18549889 | T | C | 0.115 | 0.014 | 0.002 | 7.10E-12 | 1.0E-04 | 47.00 | BMI |
| Left-leg FFM | rs68156080 | | 63781673 | G | A | 0.274 | -0.010 | 0.001 | 1.10E-11 | 1.0E-04 | 46.19 |  |
| Left-leg FFM | rs224143 | | 64477836 | A | G | 0.600 | -0.009 | 0.001 | 4.20E-12 | 1.1E-04 | 48.03 |  |
| Left-leg FFM | rs7100187 | | 66666847 | A | G | 0.505 | -0.007 | 0.001 | 2.10E-08 | 6.9E-05 | 31.38 |  |
| Left-leg FFM | rs10887571 | | 88030441 | T | C | 0.448 | 0.008 | 0.001 | 1.10E-09 | 8.2E-05 | 37.19 | BMI |
| Left-leg FFM | rs117944670 | | 98645695 | C | T | 0.053 | -0.016 | 0.003 | 1.90E-08 | 6.9E-05 | 31.59 |  |
| Left-leg FFM | rs6585827 | | 124165615 | A | G | 0.471 | 0.012 | 0.001 | 4.10E-19 | 1.8E-04 | 79.84 |  |
| Left-leg FFM | rs2666773 | | 22123768 | T | A | 0.711 | -0.009 | 0.001 | 2.90E-09 | 7.8E-05 | 35.26 | BMI |
| Left-leg FFM | rs11014285 | | 25178864 | A | G | 0.165 | 0.018 | 0.002 | 3.90E-23 | 2.2E-04 | 98.15 |  |
| Left-leg FFM | rs1657222 | | 34837139 | A | G | 0.396 | 0.009 | 0.001 | 6.70E-11 | 9.4E-05 | 42.60 |  |
| Left-leg FFM | rs10995366 | | 52772113 | A | G | 0.251 | -0.009 | 0.002 | 6.70E-10 | 8.4E-05 | 38.10 |  |
| Left-leg FFM | rs4255484 | | 77215583 | G | C | 0.559 | -0.009 | 0.001 | 6.50E-12 | 1.0E-04 | 47.16 |  |
| Left-leg FFM | rs1316312 | | 81110417 | C | G | 0.500 | -0.010 | 0.001 | 5.30E-15 | 1.3E-04 | 61.15 |  |
| Left-leg FFM | rs10883560 | | 102673707 | G | C | 0.429 | 0.017 | 0.001 | 4.80E-37 | 3.6E-04 | 161.71 | BMI |
| Left-leg FFM | rs11245450 | | 126658075 | A | G | 0.422 | -0.011 | 0.001 | 1.90E-16 | 1.5E-04 | 67.75 | BMI |
| Left-leg FFM | rs58790726 | | 70240379 | C | G | 0.429 | 0.010 | 0.001 | 1.20E-13 | 1.2E-04 | 55.00 |  |
| Left-leg FFM | rs117543413 | | 79543740 | T | C | 0.018 | -0.040 | 0.005 | 9.70E-16 | 1.4E-04 | 64.48 |  |
| Left-leg FFM | rs7912286 | | 114693230 | G | A | 0.602 | -0.009 | 0.001 | 2.90E-11 | 9.7E-05 | 44.21 |  |
| Left-leg FFM | rs7916178 | | 131144249 | T | C | 0.347 | 0.008 | 0.001 | 4.20E-08 | 6.6E-05 | 30.04 | BMI |
| Left-leg FFM | rs11191515 | | 104776527 | A | G | 0.078 | 0.025 | 0.002 | 4.70E-24 | 2.2E-04 | 102.35 | BMI |
| Left-leg FFM | rs7077087 | | 115954458 | G | A | 0.246 | 0.010 | 0.002 | 1.60E-11 | 1.0E-04 | 45.44 |  |
| Left-leg FFM | rs6591 | | 840363 | T | C | 0.544 | 0.010 | 0.001 | 5.60E-14 | 1.2E-04 | 56.50 | BMI |
| Left-leg FFM | rs676105 | | 30443688 | C | T | 0.304 | 0.013 | 0.001 | 1.50E-20 | 1.9E-04 | 86.34 | BMI |
| Left-leg FFM | rs2293576 | | 47434986 | A | G | 0.332 | -0.012 | 0.001 | 9.20E-18 | 1.6E-04 | 73.67 | BMI |
| Left-leg FFM | rs7129320 | | 68388220 | A | G | 0.166 | -0.021 | 0.002 | 1.40E-34 | 3.3E-04 | 150.46 |  |
| Left-leg FFM | rs1813212 | | 89301382 | G | A | 0.446 | -0.009 | 0.001 | 2.20E-12 | 1.1E-04 | 49.30 |  |
| Left-leg FFM | rs7933085 | | 130796248 | G | A | 0.508 | 0.008 | 0.001 | 3.50E-10 | 8.7E-05 | 39.38 | BMI |
| Left-leg FFM | rs11042321 | | 9399731 | A | G | 0.408 | 0.010 | 0.001 | 4.30E-13 | 1.2E-04 | 52.49 |  |
| Left-leg FFM | rs1789167 | | 69486244 | G | A | 0.653 | 0.012 | 0.001 | 2.30E-18 | 1.7E-04 | 76.40 | BMI |
| Left-leg FFM | rs744205 | | 69929677 | A | G | 0.544 | 0.011 | 0.001 | 1.30E-17 | 1.6E-04 | 72.94 |  |
| Left-leg FFM | rs7932719 | | 87449071 | T | A | 0.244 | 0.008 | 0.002 | 4.60E-08 | 6.6E-05 | 29.88 |  |
| Left-leg FFM | rs1869912 | | 93186433 | T | G | 0.420 | 0.007 | 0.001 | 2.40E-08 | 6.8E-05 | 31.12 |  |
| Left-leg FFM | rs11217863 | | 120293138 | A | G | 0.116 | -0.016 | 0.002 | 1.80E-14 | 1.3E-04 | 58.70 |  |
| Left-leg FFM | rs74749286 | | 14322359 | A | G | 0.108 | 0.014 | 0.002 | 1.10E-10 | 9.2E-05 | 41.68 |  |
| Left-leg FFM | rs35186585 | | 43878534 | C | A | 0.231 | 0.012 | 0.002 | 2.30E-14 | 1.3E-04 | 58.25 | BMI |
| Left-leg FFM | rs7952436 | | 67024534 | T | C | 0.082 | -0.030 | 0.002 | 5.00E-36 | 3.5E-04 | 157.03 |  |
| Left-leg FFM | rs66462629 | | 70373289 | A | C | 0.116 | -0.011 | 0.002 | 2.80E-08 | 6.8E-05 | 30.87 |  |
| Left-leg FFM | rs74048171 | | 2093603 | A | C | 0.258 | -0.011 | 0.001 | 1.90E-14 | 1.3E-04 | 58.63 |  |
| Left-leg FFM | rs35506085 | | 2165576 | A | G | 0.185 | -0.017 | 0.002 | 3.30E-24 | 2.3E-04 | 103.01 |  |
| Left-leg FFM | rs11030112 | | 27705188 | A | G | 0.319 | 0.018 | 0.001 | 2.00E-38 | 3.7E-04 | 168.06 | BMI |
| Left-leg FFM | rs573455 | | 117267884 | G | A | 0.534 | 0.007 | 0.001 | 4.20E-08 | 6.6E-05 | 30.08 |  |
| Left-leg FFM | rs7111235 | | 133658661 | C | T | 0.494 | 0.008 | 0.001 | 2.10E-10 | 8.9E-05 | 40.41 |  |
| Left-leg FFM | rs12364470 | | 134601012 | G | T | 0.165 | 0.011 | 0.002 | 2.60E-10 | 8.8E-05 | 39.94 | BMI |
| Left-leg FFM | rs10845408 | | 11880581 | T | C | 0.353 | 0.010 | 0.001 | 1.70E-13 | 1.2E-04 | 54.30 |  |
| Left-leg FFM | rs11052457 | | 33268956 | T | A | 0.038 | 0.024 | 0.003 | 4.30E-12 | 1.1E-04 | 48.00 |  |
| Left-leg FFM | rs2241960 | | 46580618 | G | A | 0.274 | 0.008 | 0.001 | 2.40E-08 | 6.8E-05 | 31.15 |  |
| Left-leg FFM | rs7959830 | | 66347368 | T | G | 0.413 | -0.024 | 0.001 | 1.90E-76 | 7.5E-04 | 342.38 |  |
| Left-leg FFM | rs2369463 | | 77432523 | C | T | 0.170 | -0.010 | 0.002 | 1.40E-08 | 7.1E-05 | 32.20 |  |
| Left-leg FFM | rs310796 | | 77453226 | T | G | 0.681 | 0.011 | 0.001 | 2.10E-15 | 1.4E-04 | 62.99 |  |
| Left-leg FFM | rs2041895 | | 107350088 | G | C | 0.434 | -0.009 | 0.001 | 9.00E-11 | 9.2E-05 | 42.04 |  |
| Left-leg FFM | rs11065979 | | 112059557 | T | C | 0.438 | -0.012 | 0.001 | 7.90E-20 | 1.8E-04 | 83.07 |  |
| Left-leg FFM | rs11058226 | | 122938958 | C | T | 0.742 | 0.019 | 0.001 | 5.00E-39 | 3.8E-04 | 170.78 | BMI |
| Left-leg FFM | rs11060406 | | 123339117 | T | C | 0.037 | -0.028 | 0.004 | 1.20E-15 | 1.4E-04 | 64.01 |  |
| Left-leg FFM | rs2291256 | | 133393323 | T | C | 0.088 | 0.017 | 0.002 | 6.00E-14 | 1.2E-04 | 56.39 |  |
| Left-leg FFM | rs4980826 | | 578349 | A | C | 0.396 | 0.008 | 0.001 | 8.50E-09 | 7.3E-05 | 33.17 |  |
| Left-leg FFM | rs12099669 | | 46783653 | A | G | 0.696 | 0.015 | 0.001 | 3.20E-26 | 2.5E-04 | 112.22 |  |
| Left-leg FFM | rs7132908 | | 50263148 | A | G | 0.384 | 0.019 | 0.001 | 9.60E-47 | 4.5E-04 | 206.12 | BMI |
| Left-leg FFM | rs2277339 | | 57146069 | G | T | 0.104 | -0.017 | 0.002 | 7.30E-15 | 1.3E-04 | 60.52 | BMI |
| Left-leg FFM | rs58035271 | | 66127644 | C | G | 0.243 | -0.010 | 0.002 | 1.80E-11 | 9.9E-05 | 45.19 |  |
| Left-leg FFM | rs146714063 | | 97498661 | A | G | 0.158 | 0.010 | 0.002 | 1.70E-08 | 7.0E-05 | 31.84 |  |
| Left-leg FFM | rs7971536 | | 102373788 | A | T | 0.494 | -0.010 | 0.001 | 1.40E-15 | 1.4E-04 | 63.73 |  |
| Left-leg FFM | rs78812993 | | 103175170 | C | G | 0.052 | -0.022 | 0.003 | 8.50E-14 | 1.2E-04 | 55.69 |  |
| Left-leg FFM | rs67551338 | | 3393100 | T | C | 0.061 | 0.024 | 0.003 | 8.60E-19 | 1.7E-04 | 78.35 |  |
| Left-leg FFM | rs76895963 | | 4384844 | G | T | 0.021 | 0.095 | 0.005 | 9.60E-80 | 7.9E-04 | 357.55 | BMI |
| Left-leg FFM | rs774214 | | 56918566 | C | T | 0.663 | -0.010 | 0.001 | 9.70E-13 | 1.1E-04 | 50.91 |  |
| Left-leg FFM | rs2241212 | | 109939641 | A | T | 0.505 | 0.008 | 0.001 | 6.20E-10 | 8.4E-05 | 38.26 |  |
| Left-leg FFM | rs11065015 | | 120520863 | T | C | 0.027 | -0.030 | 0.004 | 1.30E-13 | 1.2E-04 | 54.89 |  |
| Left-leg FFM | rs7487292 | | 122614387 | G | T | 0.447 | 0.011 | 0.001 | 4.60E-16 | 1.5E-04 | 65.98 | BMI |
| Left-leg FFM | rs55726687 | | 991306 | A | G | 0.210 | 0.016 | 0.002 | 7.60E-24 | 2.2E-04 | 101.38 | BMI |
| Left-leg FFM | rs117451679 | | 17245591 | G | A | 0.106 | 0.013 | 0.002 | 4.90E-10 | 8.5E-05 | 38.71 |  |
| Left-leg FFM | rs11049386 | | 28320536 | A | T | 0.290 | -0.009 | 0.001 | 2.80E-09 | 7.8E-05 | 35.33 |  |
| Left-leg FFM | rs1049193 | | 53873844 | C | G | 0.171 | -0.015 | 0.002 | 5.60E-18 | 1.6E-04 | 74.66 |  |
| Left-leg FFM | rs12813149 | | 90142637 | A | G | 0.243 | -0.011 | 0.002 | 1.90E-13 | 1.2E-04 | 54.06 | BMI |
| Left-leg FFM | rs2287547 | | 108924341 | C | T | 0.168 | 0.010 | 0.002 | 1.80E-08 | 7.0E-05 | 31.66 |  |
| Left-leg FFM | rs529736 | | 122384457 | C | A | 0.833 | -0.010 | 0.002 | 2.10E-09 | 7.9E-05 | 35.85 |  |
| Left-leg FFM | rs7969505 | | 12827900 | C | G | 0.114 | -0.013 | 0.002 | 3.50E-10 | 8.7E-05 | 39.38 |  |
| Left-leg FFM | rs7134283 | | 24071748 | A | G | 0.283 | -0.011 | 0.001 | 4.00E-14 | 1.3E-04 | 57.14 |  |
| Left-leg FFM | rs10843397 | | 29529523 | T | C | 0.242 | 0.009 | 0.002 | 6.80E-09 | 7.4E-05 | 33.58 |  |
| Left-leg FFM | rs12230050 | | 93990553 | G | A | 0.261 | 0.019 | 0.001 | 2.50E-36 | 3.5E-04 | 158.45 |  |
| Left-leg FFM | rs2229840 | | 124826462 | T | C | 0.160 | 0.021 | 0.002 | 1.90E-31 | 3.0E-04 | 136.12 |  |
| Left-leg FFM | rs1218822 | | 28011963 | A | G | 0.661 | 0.011 | 0.001 | 1.70E-14 | 1.3E-04 | 58.80 | BMI |
| Left-leg FFM | rs9549099 | | 40766349 | C | T | 0.265 | 0.010 | 0.001 | 3.80E-12 | 1.1E-04 | 48.20 | BMI |
| Left-leg FFM | rs9317002 | | 59175727 | A | C | 0.515 | 0.011 | 0.001 | 3.10E-18 | 1.7E-04 | 75.84 | BMI |
| Left-leg FFM | rs7985813 | | 99592595 | A | G | 0.735 | -0.009 | 0.001 | 5.40E-09 | 7.5E-05 | 34.03 |  |
| Left-leg FFM | rs7994814 | | 27054201 | T | C | 0.417 | 0.012 | 0.001 | 4.40E-20 | 1.9E-04 | 84.22 |  |
| Left-leg FFM | rs9540493 | | 66205704 | G | A | 0.545 | -0.010 | 0.001 | 1.10E-13 | 1.2E-04 | 55.21 | BMI |
| Left-leg FFM | rs11616283 | | 21493853 | C | T | 0.140 | 0.011 | 0.002 | 9.80E-09 | 7.2E-05 | 32.89 |  |
| Left-leg FFM | rs2225226 | | 51127270 | T | C | 0.216 | -0.029 | 0.002 | 3.90E-73 | 7.2E-04 | 327.20 |  |
| Left-leg FFM | rs1924936 | | 78443297 | A | T | 0.774 | 0.016 | 0.002 | 9.00E-26 | 2.4E-04 | 110.17 |  |
| Left-leg FFM | rs7994573 | | 115008388 | C | T | 0.235 | -0.010 | 0.002 | 5.00E-11 | 9.5E-05 | 43.17 |  |
| Left-leg FFM | rs1373273 | | 53973351 | A | C | 0.571 | -0.007 | 0.001 | 3.90E-08 | 6.6E-05 | 30.19 |  |
| Left-leg FFM | rs532499 | | 30165465 | C | T | 0.741 | -0.008 | 0.001 | 1.60E-08 | 7.0E-05 | 31.93 |  |
| Left-leg FFM | rs9532631 | | 41449737 | T | G | 0.198 | -0.011 | 0.002 | 1.00E-10 | 9.2E-05 | 41.82 |  |
| Left-leg FFM | rs146851424 | | 50377910 | C | A | 0.022 | 0.057 | 0.005 | 1.20E-36 | 3.5E-04 | 159.87 |  |
| Left-leg FFM | rs61950323 | | 54070046 | T | C | 0.058 | 0.020 | 0.003 | 3.20E-12 | 1.1E-04 | 48.56 | BMI |
| Left-leg FFM | rs9513143 | | 97085570 | G | A | 0.542 | 0.010 | 0.001 | 1.60E-13 | 1.2E-04 | 54.50 | BMI |
| Left-leg FFM | rs36100359 | | 21578007 | A | G | 0.139 | -0.011 | 0.002 | 1.80E-09 | 7.9E-05 | 36.13 |  |
| Left-leg FFM | rs12432051 | | 94082464 | T | A | 0.692 | 0.011 | 0.001 | 7.10E-15 | 1.3E-04 | 60.56 | BMI |
| Left-leg FFM | rs11627567 | | 103851775 | T | C | 0.215 | -0.011 | 0.002 | 8.50E-12 | 1.0E-04 | 46.65 | BMI |
| Left-leg FFM | rs17197114 | | 21894526 | C | T | 0.177 | 0.011 | 0.002 | 3.60E-10 | 8.6E-05 | 39.30 |  |
| Left-leg FFM | rs112957890 | | 36220876 | G | A | 0.265 | 0.010 | 0.001 | 5.90E-12 | 1.0E-04 | 47.38 |  |
| Left-leg FFM | rs117034105 | | 53531085 | C | T | 0.049 | 0.019 | 0.003 | 3.90E-09 | 7.6E-05 | 34.69 |  |
| Left-leg FFM | rs17105272 | | 77529783 | T | C | 0.329 | 0.009 | 0.001 | 1.80E-11 | 9.9E-05 | 45.14 |  |
| Left-leg FFM | rs148740466 | | 89016257 | A | C | 0.016 | -0.029 | 0.005 | 1.40E-08 | 7.1E-05 | 32.23 |  |
| Left-leg FFM | rs12879423 | | 25927832 | G | A | 0.679 | 0.015 | 0.001 | 1.50E-26 | 2.5E-04 | 113.69 | BMI |
| Left-leg FFM | rs12889702 | | 42894143 | C | A | 0.313 | 0.011 | 0.001 | 6.90E-14 | 1.2E-04 | 56.08 |  |
| Left-leg FFM | rs2296316 | | 65520246 | C | T | 0.465 | -0.008 | 0.001 | 1.10E-08 | 7.2E-05 | 32.70 |  |
| Left-leg FFM | rs1286058 | | 91458523 | A | T | 0.704 | 0.010 | 0.001 | 6.80E-12 | 1.0E-04 | 47.08 | BMI |
| Left-leg FFM | rs112097633 | | 93131683 | A | G | 0.077 | 0.015 | 0.002 | 5.40E-10 | 8.5E-05 | 38.52 |  |
| Left-leg FFM | rs61992671 | | 101531854 | G | A | 0.492 | -0.012 | 0.001 | 7.10E-18 | 1.6E-04 | 74.20 | BMI |
| Left-leg FFM | rs12880432 | | 103267500 | C | G | 0.647 | -0.008 | 0.001 | 9.20E-09 | 7.3E-05 | 33.01 | BMI |
| Left-leg FFM | rs3212260 | | 24804088 | T | A | 0.258 | 0.012 | 0.001 | 1.80E-15 | 1.4E-04 | 63.23 |  |
| Left-leg FFM | rs8007644 | | 35218831 | A | G | 0.388 | 0.008 | 0.001 | 3.00E-09 | 7.7E-05 | 35.19 |  |
| Left-leg FFM | rs4899012 | | 61003889 | C | G | 0.607 | -0.019 | 0.001 | 4.00E-45 | 4.4E-04 | 198.72 |  |
| Left-leg FFM | rs7141420 | | 79899454 | T | C | 0.516 | 0.014 | 0.001 | 5.40E-28 | 2.6E-04 | 120.30 | BMI |
| Left-leg FFM | rs9788443 | | 23878279 | C | T | 0.048 | 0.017 | 0.003 | 2.10E-08 | 6.9E-05 | 31.42 |  |
| Left-leg FFM | rs12147845 | | 101144596 | T | C | 0.116 | 0.013 | 0.002 | 1.80E-10 | 8.9E-05 | 40.63 | BMI |
| Left-leg FFM | rs12906197 | | 38492199 | T | C | 0.422 | -0.010 | 0.001 | 1.60E-13 | 1.2E-04 | 54.49 |  |
| Left-leg FFM | rs55887408 | | 52073816 | A | G | 0.450 | -0.008 | 0.001 | 1.20E-09 | 8.1E-05 | 37.02 | BMI |
| Left-leg FFM | rs4143843 | | 62373121 | T | C | 0.448 | -0.010 | 0.001 | 1.30E-13 | 1.2E-04 | 54.80 | BMI |
| Left-leg FFM | rs9920235 | | 79384021 | T | C | 0.399 | -0.009 | 0.001 | 3.40E-12 | 1.1E-04 | 48.44 | BMI |
| Left-leg FFM | rs11638457 | | 81012407 | G | C | 0.444 | 0.010 | 0.001 | 5.80E-15 | 1.3E-04 | 60.98 | BMI |
| Left-leg FFM | rs11630112 | | 86120246 | C | T | 0.268 | 0.009 | 0.001 | 9.20E-10 | 8.2E-05 | 37.49 |  |
| Left-leg FFM | rs11855017 | | 42096146 | A | C | 0.182 | 0.013 | 0.002 | 3.60E-14 | 1.3E-04 | 57.40 | BMI |
| Left-leg FFM | rs2414910 | | 66976149 | T | G | 0.788 | -0.010 | 0.002 | 4.40E-10 | 8.6E-05 | 38.94 |  |
| Left-leg FFM | rs8040399 | | 89432795 | G | C | 0.588 | -0.008 | 0.001 | 3.20E-10 | 8.7E-05 | 39.54 |  |
| Left-leg FFM | rs1573891 | | 99186488 | C | G | 0.158 | -0.019 | 0.002 | 4.80E-25 | 2.3E-04 | 106.86 |  |
| Left-leg FFM | rs11071182 | | 55644676 | G | A | 0.870 | 0.015 | 0.002 | 3.60E-14 | 1.3E-04 | 57.36 |  |
| Left-leg FFM | rs8030456 | | 68076856 | T | C | 0.226 | -0.014 | 0.002 | 2.90E-19 | 1.8E-04 | 80.50 | BMI |
| Left-leg FFM | rs62621400 | | 101718239 | G | C | 0.058 | -0.021 | 0.003 | 2.50E-13 | 1.2E-04 | 53.57 |  |
| Left-leg FFM | rs2663125 | | 99563891 | C | T | 0.691 | -0.009 | 0.001 | 3.30E-11 | 9.7E-05 | 44.02 |  |
| Left-leg FFM | rs13333747 | | 2175373 | C | T | 0.183 | -0.018 | 0.002 | 4.80E-27 | 2.5E-04 | 115.97 | BMI |
| Left-leg FFM | rs2539999 | | 2266173 | C | T | 0.287 | 0.015 | 0.001 | 7.20E-24 | 2.2E-04 | 101.48 |  |
| Left-leg FFM | rs35467921 | | 30048553 | T | C | 0.400 | 0.021 | 0.001 | 1.60E-57 | 5.6E-04 | 255.57 | BMI |
| Left-leg FFM | rs7188009 | | 81660642 | A | G | 0.404 | 0.008 | 0.001 | 2.00E-09 | 7.9E-05 | 35.93 |  |
| Left-leg FFM | rs12926311 | | 406427 | C | G | 0.354 | -0.009 | 0.001 | 5.90E-11 | 9.4E-05 | 42.85 | BMI |
| Left-leg FFM | rs9926458 | | 4872628 | A | G | 0.379 | 0.009 | 0.001 | 1.80E-11 | 9.9E-05 | 45.23 | BMI |
| Left-leg FFM | rs11648796 | | 792190 | G | A | 0.232 | 0.014 | 0.002 | 8.30E-19 | 1.7E-04 | 78.42 |  |
| Left-leg FFM | rs2238435 | | 4014282 | G | C | 0.614 | 0.019 | 0.001 | 2.10E-44 | 4.3E-04 | 195.45 | BMI |
| Left-leg FFM | rs4985124 | | 15125441 | G | T | 0.297 | -0.012 | 0.001 | 4.20E-17 | 1.6E-04 | 70.69 |  |
| Left-leg FFM | rs34017457 | | 67166731 | A | G | 0.007 | 0.050 | 0.008 | 1.60E-10 | 9.0E-05 | 40.89 |  |
| Left-leg FFM | rs71393968 | | 67847341 | A | G | 0.046 | 0.020 | 0.003 | 5.20E-11 | 9.5E-05 | 43.09 |  |
| Left-leg FFM | rs72801843 | | 53508802 | A | T | 0.301 | 0.017 | 0.001 | 1.70E-32 | 3.1E-04 | 140.86 |  |
| Left-leg FFM | rs8059189 | | 86417349 | A | G | 0.404 | -0.011 | 0.001 | 8.40E-15 | 1.3E-04 | 60.25 |  |
| Left-leg FFM | rs10775348 | | 88806348 | G | A | 0.704 | 0.012 | 0.001 | 1.10E-16 | 1.5E-04 | 68.87 |  |
| Left-leg FFM | rs72771080 | | 20021383 | T | C | 0.212 | 0.014 | 0.002 | 6.30E-18 | 1.6E-04 | 74.43 | BMI |
| Left-leg FFM | rs35626515 | | 28649651 | A | C | 0.405 | 0.017 | 0.001 | 2.40E-36 | 3.5E-04 | 158.48 | BMI |
| Left-leg FFM | rs56094641 | | 53806453 | G | A | 0.405 | 0.042 | 0.001 | 1.00E-200 | 2.2E-03 | 1004.93 | BMI |
| Left-leg FFM | rs76513770 | | 72505534 | C | T | 0.128 | -0.017 | 0.002 | 1.50E-17 | 1.6E-04 | 72.67 |  |
| Left-leg FFM | rs55831773 | | 7559037 | T | C | 0.199 | -0.015 | 0.002 | 2.50E-19 | 1.8E-04 | 80.79 |  |
| Left-leg FFM | rs1242510 | | 17371405 | T | C | 0.801 | 0.010 | 0.002 | 6.00E-09 | 7.4E-05 | 33.82 |  |
| Left-leg FFM | rs72833620 | | 47056753 | C | A | 0.096 | 0.018 | 0.002 | 1.70E-15 | 1.4E-04 | 63.34 | BMI |
| Left-leg FFM | rs2005172 | | 61996255 | C | A | 0.640 | 0.019 | 0.001 | 5.40E-45 | 4.4E-04 | 198.11 |  |
| Left-leg FFM | rs34055910 | | 64460104 | G | A | 0.379 | -0.007 | 0.001 | 4.60E-08 | 6.6E-05 | 29.87 |  |
| Left-leg FFM | rs2592209 | | 67408931 | C | G | 0.513 | -0.007 | 0.001 | 2.40E-08 | 6.8E-05 | 31.15 |  |
| Left-leg FFM | rs4968799 | | 68158831 | T | A | 0.112 | -0.017 | 0.002 | 1.90E-16 | 1.5E-04 | 67.69 |  |
| Left-leg FFM | rs236587 | | 68203915 | C | T | 0.737 | -0.008 | 0.001 | 4.70E-08 | 6.6E-05 | 29.82 |  |
| Left-leg FFM | rs2252909 | | 2278609 | T | C | 0.657 | -0.008 | 0.001 | 2.00E-08 | 6.9E-05 | 31.53 |  |
| Left-leg FFM | rs2301652 | | 15928584 | G | T | 0.543 | 0.009 | 0.001 | 1.30E-11 | 1.0E-04 | 45.75 | BMI |
| Left-leg FFM | rs3110496 | | 27917771 | G | A | 0.686 | 0.008 | 0.001 | 6.80E-09 | 7.4E-05 | 33.59 |  |
| Left-leg FFM | rs6505044 | | 54258240 | C | A | 0.462 | -0.008 | 0.001 | 8.20E-09 | 7.3E-05 | 33.22 |  |
| Left-leg FFM | rs757608 | | 59497277 | G | A | 0.670 | -0.012 | 0.001 | 2.30E-18 | 1.7E-04 | 76.41 | BMI |
| Left-leg FFM | rs4525525 | | 1866892 | T | G | 0.264 | -0.011 | 0.001 | 4.50E-13 | 1.2E-04 | 52.40 | BMI |
| Left-leg FFM | rs28613067 | | 46781635 | A | G | 0.278 | 0.009 | 0.001 | 2.10E-10 | 8.9E-05 | 40.35 |  |
| Left-leg FFM | rs12946718 | | 77777125 | A | C | 0.226 | -0.009 | 0.002 | 4.80E-09 | 7.5E-05 | 34.26 |  |
| Left-leg FFM | rs36000545 | | 79093822 | G | A | 0.396 | -0.016 | 0.001 | 5.90E-32 | 3.0E-04 | 138.41 |  |
| Left-leg FFM | rs78378222 | | 7571752 | G | T | 0.012 | 0.077 | 0.006 | 6.70E-37 | 3.5E-04 | 161.03 |  |
| Left-leg FFM | rs2314338 | | 38344485 | C | T | 0.269 | 0.011 | 0.001 | 7.50E-13 | 1.1E-04 | 51.42 |  |
| Left-leg FFM | rs62070648 | | 29210595 | A | G | 0.269 | -0.022 | 0.001 | 8.20E-50 | 4.8E-04 | 220.20 |  |
| Left-leg FFM | rs12951408 | | 36913807 | C | T | 0.559 | 0.011 | 0.001 | 1.20E-16 | 1.5E-04 | 68.69 |  |
| Left-leg FFM | rs6416914 | | 39262850 | G | A | 0.695 | -0.009 | 0.001 | 4.50E-11 | 9.5E-05 | 43.39 | BMI |
| Left-leg FFM | rs9889839 | | 78270679 | G | A | 0.455 | 0.007 | 0.001 | 2.30E-08 | 6.9E-05 | 31.25 |  |
| Left-leg FFM | rs7220294 | | 78627753 | C | T | 0.233 | -0.011 | 0.002 | 2.00E-12 | 1.1E-04 | 49.49 | BMI |
| Left-leg FFM | rs4392169 | | 20724931 | T | A | 0.785 | 0.020 | 0.002 | 1.30E-36 | 3.5E-04 | 159.75 |  |
| Left-leg FFM | rs9957318 | | 33039106 | G | A | 0.348 | 0.008 | 0.001 | 2.00E-09 | 7.9E-05 | 35.93 |  |
| Left-leg FFM | rs2276190 | | 63430340 | A | G | 0.277 | 0.009 | 0.001 | 4.70E-10 | 8.5E-05 | 38.79 | BMI |
| Left-leg FFM | rs57126421 | | 2656989 | G | A | 0.238 | -0.011 | 0.002 | 4.80E-13 | 1.1E-04 | 52.29 |  |
| Left-leg FFM | rs55854145 | | 45928049 | C | A | 0.055 | -0.016 | 0.003 | 1.40E-08 | 7.1E-05 | 32.24 |  |
| Left-leg FFM | rs33973388 | | 46611842 | T | G | 0.435 | 0.010 | 0.001 | 6.30E-14 | 1.2E-04 | 56.27 |  |
| Left-leg FFM | rs6567160 | | 57829135 | C | T | 0.233 | 0.045 | 0.002 | 2.30E-185 | 1.9E-03 | 843.09 | BMI |
| Left-leg FFM | rs1941697 | | 31251276 | A | G | 0.449 | 0.008 | 0.001 | 1.80E-09 | 8.0E-05 | 36.20 | BMI |
| Left-leg FFM | rs4552110 | | 57183948 | T | A | 0.158 | -0.011 | 0.002 | 8.80E-10 | 8.3E-05 | 37.58 |  |
| Left-leg FFM | rs751894 | | 60756884 | T | C | 0.206 | 0.009 | 0.002 | 1.30E-08 | 7.1E-05 | 32.31 |  |
| Left-leg FFM | rs74494415 | | 74972138 | T | C | 0.040 | -0.023 | 0.003 | 6.40E-12 | 1.0E-04 | 47.21 |  |
| Left-leg FFM | rs11663903 | | 32762592 | A | G | 0.429 | 0.008 | 0.001 | 1.20E-08 | 7.1E-05 | 32.46 |  |
| Left-leg FFM | rs7229520 | | 46516468 | A | G | 0.662 | -0.010 | 0.001 | 2.10E-13 | 1.2E-04 | 53.93 |  |
| Left-leg FFM | rs1517037 | | 56878274 | T | C | 0.188 | -0.014 | 0.002 | 8.30E-17 | 1.5E-04 | 69.34 |  |
| Left-leg FFM | rs57636386 | | 58048295 | C | T | 0.084 | -0.025 | 0.002 | 2.50E-25 | 2.4E-04 | 108.11 | BMI |
| Left-leg FFM | rs2163832 | | 10745764 | C | T | 0.660 | -0.012 | 0.001 | 1.30E-19 | 1.8E-04 | 82.08 |  |
| Left-leg FFM | rs73004967 | | 19717056 | G | A | 0.069 | -0.021 | 0.003 | 5.30E-16 | 1.4E-04 | 65.66 |  |
| Left-leg FFM | rs284662 | | 41932275 | C | T | 0.620 | -0.009 | 0.001 | 1.00E-10 | 9.2E-05 | 41.81 |  |
| Left-leg FFM | rs35050648 | | 46991243 | T | G | 0.236 | 0.009 | 0.002 | 4.50E-09 | 7.6E-05 | 34.39 |  |
| Left-leg FFM | rs11880992 | | 2176403 | A | G | 0.408 | 0.012 | 0.001 | 6.10E-20 | 1.8E-04 | 83.57 |  |
| Left-leg FFM | rs58857770 | | 7208110 | G | C | 0.408 | -0.008 | 0.001 | 5.80E-10 | 8.4E-05 | 38.38 |  |
| Left-leg FFM | rs147110934 | | 55993436 | T | G | 0.024 | -0.028 | 0.004 | 2.50E-11 | 9.8E-05 | 44.51 |  |
| Left-leg FFM | rs7253519 | | 1986520 | A | G | 0.119 | -0.013 | 0.002 | 1.00E-10 | 9.2E-05 | 41.76 | BMI |
| Left-leg FFM | rs62621197 | | 8670147 | T | C | 0.037 | -0.034 | 0.004 | 1.70E-21 | 2.0E-04 | 90.66 |  |
| Left-leg FFM | rs3810291 | | 47569003 | A | G | 0.675 | 0.018 | 0.001 | 2.10E-39 | 3.8E-04 | 172.50 | BMI |
| Left-leg FFM | rs4807473 | | 3448869 | G | A | 0.642 | -0.010 | 0.001 | 1.60E-14 | 1.3E-04 | 58.99 |  |
| Left-leg FFM | rs4808845 | | 18812024 | G | A | 0.400 | -0.009 | 0.001 | 4.30E-11 | 9.6E-05 | 43.46 | BMI |
| Left-leg FFM | rs111640872 | | 30290357 | C | G | 0.331 | 0.016 | 0.001 | 2.70E-29 | 2.8E-04 | 126.27 | BMI |
| Left-leg FFM | rs2252720 | | 21223663 | T | C | 0.674 | -0.010 | 0.001 | 4.70E-13 | 1.2E-04 | 52.33 |  |
| Left-leg FFM | rs34879158 | | 32300634 | C | A | 0.263 | -0.020 | 0.001 | 6.40E-41 | 3.9E-04 | 179.46 |  |
| Left-leg FFM | rs73619441 | | 61564901 | G | T | 0.144 | -0.012 | 0.002 | 9.50E-11 | 9.2E-05 | 41.93 | BMI |
| Left-leg FFM | rs143384 | | 34025756 | G | A | 0.404 | 0.032 | 0.001 | 1.20E-131 | 1.3E-03 | 595.98 |  |
| Left-leg FFM | rs6051425 | | 2815318 | G | C | 0.510 | -0.008 | 0.001 | 1.00E-09 | 8.2E-05 | 37.25 |  |
| Left-leg FFM | rs16996657 | | 15816236 | C | T | 0.128 | 0.012 | 0.002 | 8.40E-10 | 8.3E-05 | 37.66 | BMI |
| Left-leg FFM | rs6136938 | | 20058992 | A | G | 0.436 | -0.008 | 0.001 | 2.30E-10 | 8.8E-05 | 40.18 |  |
| Left-leg FFM | rs6142059 | | 32544327 | C | T | 0.492 | 0.010 | 0.001 | 1.40E-13 | 1.2E-04 | 54.69 | BMI |
| Left-leg FFM | rs1291066 | | 35790918 | C | G | 0.809 | 0.012 | 0.002 | 1.10E-12 | 1.1E-04 | 50.59 |  |
| Left-leg FFM | rs6026578 | | 57463472 | G | C | 0.625 | -0.010 | 0.001 | 5.90E-14 | 1.2E-04 | 56.41 |  |
| Left-leg FFM | rs2007022 | | 3237674 | A | C | 0.231 | 0.009 | 0.002 | 2.20E-08 | 6.9E-05 | 31.35 |  |
| Left-leg FFM | rs6124249 | | 39175029 | C | T | 0.316 | 0.009 | 0.001 | 5.20E-10 | 8.5E-05 | 38.60 |  |
| Left-leg FFM | rs13043303 | | 51091385 | A | G | 0.183 | -0.019 | 0.002 | 3.00E-28 | 2.7E-04 | 121.49 | BMI |
| Left-leg FFM | rs9976812 | | 39690245 | G | C | 0.564 | -0.013 | 0.001 | 6.80E-23 | 2.1E-04 | 97.05 |  |
| Left-leg FFM | rs73189390 | | 17383170 | A | G | 0.184 | -0.010 | 0.002 | 6.50E-09 | 7.4E-05 | 33.67 |  |
| Left-leg FFM | rs4819021 | | 46466927 | C | T | 0.477 | -0.009 | 0.001 | 1.30E-12 | 1.1E-04 | 50.30 | BMI |
| Left-leg FFM | rs165656 | | 19948863 | C | G | 0.517 | 0.008 | 0.001 | 9.50E-10 | 8.2E-05 | 37.42 |  |
| Left-leg FFM | rs5752989 | | 30365780 | A | G | 0.571 | -0.009 | 0.001 | 6.10E-12 | 1.0E-04 | 47.29 |  |
| Left-leg FFM | rs41311445 | | 42070374 | C | A | 0.096 | -0.022 | 0.002 | 3.30E-22 | 2.1E-04 | 93.88 |  |
| Left-leg FFM | rs113619763 | | 46399175 | T | A | 0.062 | 0.016 | 0.003 | 7.10E-09 | 7.4E-05 | 33.51 |  |
| Left-leg FFM | rs5771118 | | 50714289 | C | T | 0.742 | 0.009 | 0.002 | 6.50E-10 | 8.4E-05 | 38.15 |  |
| Left-leg FFM | rs5762873 | | 29265512 | A | T | 0.045 | -0.018 | 0.003 | 2.20E-08 | 6.9E-05 | 31.32 |  |
|  | |

Abbreviation: SNP, single nucleotide polymorphism; EA, Effect allele; NEA, Non-effect allele; EAF, effect allele frequency; SE, standard error; BMI, body mass index.

*^a^ R^2^* was calculated using the following formula: (2×EAF×(1-EAF)×beta^2^)/[(2×EAF×(1-EAF)×beta^2^)+(2×EAF×(1-EAF)×N×SE^2^)], where EAF is the effect allele

frequency, beta is the estimated effect on urate. Ν is the sample size of the GWAS for the SNP-urate association and SE is the standard error of the estimated effect.

*^b^ F* statistic was calculated using the following formula: *R^2^*(N-2)/(1-*R^2^*), where *R^2^* is the proportion of variance in urate explained by each instrument and N is the sample size of the GWAS for the SNP-urate association.

^c^ SNPs associated with confounding factors were removed after searching Phenoscanner database.

Supplementary Table 9 Characteristics of SNPs used as genetic instruments for right-arm FFM

| Exposure | SNP | | Position | EA | NEA | EAF | SNP-Exposure association | | | R^2 a^ | F-statistic ^b^ | Confounders ^c^ |
| --- | --- | --- | --- | --- | --- | --- | --- | --- | --- | --- | --- | --- |
|  |  | |  |  |  |  | Beta | SE | P value |  |  |  |
| Right-arm FFM | rs226250 | | 8022171 | T | C | 0.406 | -0.008 | 0.001 | 2.90E-11 | 9.7E-05 | 44.21 |  |
| Right-arm FFM | rs7542242 | | 22477493 | T | C | 0.329 | -0.009 | 0.001 | 7.00E-11 | 9.4E-05 | 42.53 |  |
| Right-arm FFM | rs2783712 | | 26429344 | C | A | 0.174 | 0.014 | 0.002 | 2.10E-17 | 1.6E-04 | 72.05 |  |
| Right-arm FFM | rs652112 | | 32345980 | C | T | 0.105 | 0.017 | 0.002 | 8.00E-18 | 1.6E-04 | 73.95 |  |
| Right-arm FFM | rs71647469 | | 65366509 | T | C | 0.013 | 0.036 | 0.006 | 3.60E-10 | 8.6E-05 | 39.30 |  |
| Right-arm FFM | rs34517439 | | 78450517 | A | C | 0.122 | 0.034 | 0.002 | 6.30E-70 | 6.9E-04 | 312.48 | BMI |
| Right-arm FFM | rs60804050 | | 118870373 | A | G | 0.256 | -0.011 | 0.001 | 7.60E-14 | 1.2E-04 | 55.92 |  |
| Right-arm FFM | rs76798800 | | 154994978 | T | G | 0.266 | 0.021 | 0.001 | 3.70E-49 | 4.8E-04 | 217.19 | BMI |
| Right-arm FFM | rs7513326 | | 159895536 | A | G | 0.508 | -0.007 | 0.001 | 1.70E-08 | 7.0E-05 | 31.80 |  |
| Right-arm FFM | rs1022523 | | 176788394 | A | G | 0.727 | 0.016 | 0.001 | 1.40E-29 | 2.8E-04 | 127.56 |  |
| Right-arm FFM | rs527248 | | 177875514 | G | A | 0.209 | 0.022 | 0.002 | 4.40E-49 | 4.8E-04 | 216.86 | BMI |
| Right-arm FFM | rs10920678 | | 190239907 | G | A | 0.563 | -0.008 | 0.001 | 1.20E-11 | 1.0E-04 | 45.90 | BMI |
| Right-arm FFM | rs12354311 | | 224640099 | C | A | 0.306 | -0.007 | 0.001 | 4.60E-08 | 6.6E-05 | 29.89 |  |
| Right-arm FFM | rs74598702 | | 228622065 | T | C | 0.093 | -0.016 | 0.002 | 1.70E-13 | 1.2E-04 | 54.38 |  |
| Right-arm FFM | rs72660086 | | 39571992 | G | T | 0.211 | 0.011 | 0.002 | 2.50E-13 | 1.2E-04 | 53.57 | BMI |
| Right-arm FFM | rs12731454 | | 97067652 | G | A | 0.309 | 0.011 | 0.001 | 2.40E-15 | 1.4E-04 | 62.68 | BMI |
| Right-arm FFM | rs17277008 | | 172105162 | C | T | 0.313 | 0.014 | 0.001 | 8.50E-26 | 2.4E-04 | 110.29 |  |
| Right-arm FFM | rs3850625 | | 201016296 | A | G | 0.118 | -0.019 | 0.002 | 5.50E-23 | 2.1E-04 | 97.47 |  |
| Right-arm FFM | rs12048049 | | 218597297 | G | C | 0.287 | 0.012 | 0.001 | 7.20E-20 | 1.8E-04 | 83.26 |  |
| Right-arm FFM | rs903908 | | 2202967 | C | T | 0.509 | 0.008 | 0.001 | 1.70E-11 | 1.0E-04 | 45.26 |  |
| Right-arm FFM | rs10798945 | | 33858873 | T | C | 0.751 | 0.012 | 0.001 | 8.40E-17 | 1.5E-04 | 69.32 |  |
| Right-arm FFM | rs272815 | | 36648011 | C | T | 0.580 | 0.008 | 0.001 | 1.80E-09 | 8.0E-05 | 36.13 |  |
| Right-arm FFM | rs12095997 | | 51391845 | T | C | 0.089 | 0.019 | 0.002 | 1.30E-18 | 1.7E-04 | 77.49 |  |
| Right-arm FFM | rs17363646 | | 86823503 | G | A | 0.136 | 0.012 | 0.002 | 1.60E-11 | 1.0E-04 | 45.35 |  |
| Right-arm FFM | rs903678 | | 201809918 | A | G | 0.337 | 0.012 | 0.001 | 7.00E-19 | 1.7E-04 | 78.78 | BMI |
| Right-arm FFM | rs823118 | | 205723572 | T | C | 0.548 | -0.012 | 0.001 | 1.40E-23 | 2.2E-04 | 100.12 | BMI |
| Right-arm FFM | rs3790731 | | 217795891 | A | T | 0.397 | 0.009 | 0.001 | 2.50E-12 | 1.1E-04 | 49.06 |  |
| Right-arm FFM | rs6659660 | | 225962333 | G | A | 0.682 | 0.008 | 0.001 | 1.30E-09 | 8.1E-05 | 36.76 |  |
| Right-arm FFM | rs2885697 | | 41544279 | T | G | 0.665 | -0.018 | 0.001 | 6.40E-44 | 4.2E-04 | 193.20 |  |
| Right-arm FFM | rs12140153 | | 62579891 | T | G | 0.094 | -0.015 | 0.002 | 2.90E-12 | 1.1E-04 | 48.76 | BMI |
| Right-arm FFM | rs6690871 | | 74977277 | G | A | 0.407 | 0.008 | 0.001 | 9.60E-10 | 8.2E-05 | 37.40 | BMI |
| Right-arm FFM | rs77848106 | | 107971673 | A | C | 0.296 | -0.008 | 0.001 | 5.20E-09 | 7.5E-05 | 34.12 | BMI |
| Right-arm FFM | rs3879448 | | 109891423 | C | G | 0.710 | -0.009 | 0.001 | 3.70E-10 | 8.6E-05 | 39.28 |  |
| Right-arm FFM | rs7535501 | | 176462695 | T | C | 0.944 | -0.015 | 0.003 | 3.00E-08 | 6.8E-05 | 30.72 |  |
| Right-arm FFM | rs2789366 | | 235508270 | A | G | 0.346 | -0.008 | 0.001 | 6.40E-11 | 9.4E-05 | 42.68 |  |
| Right-arm FFM | rs2295363 | | 1850428 | G | A | 0.548 | 0.010 | 0.001 | 4.80E-15 | 1.3E-04 | 61.35 | BMI |
| Right-arm FFM | rs67373773 | | 2710377 | A | G | 0.439 | -0.007 | 0.001 | 3.80E-09 | 7.6E-05 | 34.71 | BMI |
| Right-arm FFM | rs1205593 | | 11252716 | C | T | 0.759 | -0.014 | 0.001 | 1.90E-22 | 2.1E-04 | 95.01 | BMI |
| Right-arm FFM | rs11590433 | | 21505827 | G | A | 0.649 | 0.009 | 0.001 | 9.10E-12 | 1.0E-04 | 46.51 |  |
| Right-arm FFM | rs28605759 | | 38399816 | A | G | 0.454 | -0.007 | 0.001 | 1.20E-08 | 7.2E-05 | 32.54 |  |
| Right-arm FFM | rs4926542 | | 50263773 | T | C | 0.682 | -0.009 | 0.001 | 1.20E-12 | 1.1E-04 | 50.44 | BMI |
| Right-arm FFM | rs6669139 | | 56583110 | T | C | 0.209 | 0.013 | 0.002 | 6.70E-19 | 1.7E-04 | 78.84 |  |
| Right-arm FFM | rs12726084 | | 72649154 | T | C | 0.204 | -0.013 | 0.002 | 6.50E-17 | 1.5E-04 | 69.81 | BMI |
| Right-arm FFM | rs72697614 | | 107514107 | A | C | 0.320 | 0.008 | 0.001 | 6.60E-10 | 8.4E-05 | 38.13 | BMI |
| Right-arm FFM | rs10749659 | | 151033979 | T | C | 0.772 | -0.010 | 0.001 | 3.50E-11 | 9.6E-05 | 43.85 | BMI |
| Right-arm FFM | rs6675441 | | 214659762 | A | G | 0.234 | -0.011 | 0.001 | 1.10E-14 | 1.3E-04 | 59.73 |  |
| Right-arm FFM | rs59985551 | | 56106928 | T | C | 0.226 | -0.014 | 0.001 | 1.50E-20 | 1.9E-04 | 86.33 |  |
| Right-arm FFM | rs6733029 | | 68434157 | C | T | 0.393 | -0.011 | 0.001 | 3.60E-17 | 1.6E-04 | 70.96 |  |
| Right-arm FFM | rs1528450 | | 181517642 | C | T | 0.597 | 0.008 | 0.001 | 3.50E-10 | 8.7E-05 | 39.39 | BMI |
| Right-arm FFM | rs11684531 | | 219835489 | G | A | 0.133 | -0.011 | 0.002 | 5.80E-10 | 8.4E-05 | 38.39 |  |
| Right-arm FFM | rs17246129 | | 227259964 | A | G | 0.305 | 0.012 | 0.001 | 2.10E-18 | 1.7E-04 | 76.55 |  |
| Right-arm FFM | rs3116201 | | 233074205 | A | G | 0.098 | -0.018 | 0.002 | 3.00E-17 | 1.6E-04 | 71.33 |  |
| Right-arm FFM | rs6721191 | | 10190115 | G | A | 0.578 | -0.008 | 0.001 | 7.20E-11 | 9.3E-05 | 42.47 |  |
| Right-arm FFM | rs4671197 | | 67952811 | C | T | 0.688 | -0.007 | 0.001 | 3.50E-08 | 6.7E-05 | 30.39 |  |
| Right-arm FFM | rs2871392 | | 101716400 | C | T | 0.325 | -0.007 | 0.001 | 4.10E-08 | 6.6E-05 | 30.09 |  |
| Right-arm FFM | rs13392139 | | 121578767 | G | A | 0.066 | -0.014 | 0.003 | 8.40E-09 | 7.3E-05 | 33.17 |  |
| Right-arm FFM | rs10931008 | | 183238919 | C | T | 0.343 | -0.011 | 0.001 | 3.30E-17 | 1.6E-04 | 71.14 |  |
| Right-arm FFM | rs1047891 | | 211540507 | A | C | 0.316 | 0.016 | 0.001 | 3.40E-32 | 3.1E-04 | 139.54 | BMI |
| Right-arm FFM | rs13430869 | | 218146818 | T | G | 0.742 | 0.013 | 0.001 | 3.00E-19 | 1.8E-04 | 80.46 |  |
| Right-arm FFM | rs1542224 | | 223963874 | C | T | 0.719 | 0.011 | 0.001 | 4.00E-16 | 1.5E-04 | 66.23 |  |
| Right-arm FFM | rs77165542 | | 430975 | T | C | 0.035 | -0.053 | 0.003 | 7.70E-55 | 5.3E-04 | 243.24 | BMI |
| Right-arm FFM | rs112544217 | | 20222686 | T | C | 0.022 | -0.024 | 0.004 | 3.80E-08 | 6.7E-05 | 30.23 |  |
| Right-arm FFM | rs343954 | | 44999709 | C | T | 0.172 | 0.011 | 0.002 | 3.60E-12 | 1.1E-04 | 48.32 |  |
| Right-arm FFM | rs17049820 | | 59139507 | C | T | 0.113 | -0.011 | 0.002 | 3.90E-08 | 6.6E-05 | 30.21 | BMI |
| Right-arm FFM | rs7581335 | | 143958864 | T | A | 0.141 | 0.011 | 0.002 | 4.90E-10 | 8.5E-05 | 38.73 | BMI |
| Right-arm FFM | rs12694042 | | 207029825 | T | C | 0.502 | -0.009 | 0.001 | 3.90E-13 | 1.2E-04 | 52.69 |  |
| Right-arm FFM | rs1478575 | | 218278555 | A | T | 0.684 | 0.015 | 0.001 | 2.40E-30 | 2.9E-04 | 131.03 |  |
| Right-arm FFM | rs1260326 | | 27730940 | C | T | 0.604 | 0.018 | 0.001 | 9.70E-48 | 4.6E-04 | 210.70 |  |
| Right-arm FFM | rs115179432 | | 33348679 | G | A | 0.072 | -0.021 | 0.002 | 6.80E-19 | 1.7E-04 | 78.83 |  |
| Right-arm FFM | rs6543706 | | 33559710 | T | G | 0.090 | -0.014 | 0.002 | 1.10E-10 | 9.2E-05 | 41.65 |  |
| Right-arm FFM | rs6711568 | | 59291172 | G | T | 0.702 | -0.010 | 0.001 | 2.20E-13 | 1.2E-04 | 53.82 | BMI |
| Right-arm FFM | rs1374370 | | 85818273 | A | G | 0.305 | 0.010 | 0.001 | 5.10E-13 | 1.1E-04 | 52.17 |  |
| Right-arm FFM | rs13026283 | | 100842638 | T | C | 0.393 | -0.007 | 0.001 | 4.80E-08 | 6.6E-05 | 29.79 | BMI |
| Right-arm FFM | rs6719296 | | 103101108 | A | G | 0.495 | 0.008 | 0.001 | 4.00E-11 | 9.6E-05 | 43.59 |  |
| Right-arm FFM | rs6747657 | | 112003163 | A | G | 0.282 | 0.008 | 0.001 | 3.60E-08 | 6.7E-05 | 30.34 |  |
| Right-arm FFM | rs62160072 | | 128912223 | T | C | 0.199 | 0.009 | 0.002 | 7.70E-09 | 7.3E-05 | 33.35 |  |
| Right-arm FFM | rs13392079 | | 169119178 | C | T | 0.756 | -0.009 | 0.001 | 1.80E-09 | 8.0E-05 | 36.15 |  |
| Right-arm FFM | rs72885917 | | 172416376 | C | A | 0.247 | -0.018 | 0.001 | 1.00E-36 | 3.5E-04 | 160.15 |  |
| Right-arm FFM | rs1064213 | | 198950240 | A | G | 0.478 | 0.012 | 0.001 | 1.80E-23 | 2.2E-04 | 99.65 | BMI |
| Right-arm FFM | rs17443541 | | 200402624 | C | T | 0.189 | -0.011 | 0.002 | 3.70E-12 | 1.1E-04 | 48.27 |  |
| Right-arm FFM | rs6435153 | | 203292039 | T | C | 0.101 | -0.011 | 0.002 | 4.50E-08 | 6.6E-05 | 29.93 |  |
| Right-arm FFM | rs2197563 | | 233687080 | A | G | 0.595 | 0.010 | 0.001 | 8.80E-15 | 1.3E-04 | 60.15 |  |
| Right-arm FFM | rs10170971 | | 242492779 | C | G | 0.502 | -0.009 | 0.001 | 3.70E-12 | 1.1E-04 | 48.29 |  |
| Right-arm FFM | rs12995666 | | 629959 | C | T | 0.828 | 0.032 | 0.002 | 2.30E-83 | 8.2E-04 | 374.16 | BMI |
| Right-arm FFM | rs12713004 | | 23896049 | G | A | 0.725 | 0.016 | 0.001 | 1.70E-32 | 3.1E-04 | 140.94 |  |
| Right-arm FFM | rs11689727 | | 25458100 | A | C | 0.331 | -0.010 | 0.001 | 3.40E-14 | 1.3E-04 | 57.47 |  |
| Right-arm FFM | rs2372604 | | 36820390 | T | G | 0.380 | 0.008 | 0.001 | 4.00E-10 | 8.6E-05 | 39.10 | body fat percentage |
| Right-arm FFM | rs10170082 | | 42623452 | C | T | 0.130 | -0.015 | 0.002 | 1.80E-16 | 1.5E-04 | 67.79 |  |
| Right-arm FFM | rs4852257 | | 71678520 | G | T | 0.576 | -0.010 | 0.001 | 7.90E-16 | 1.4E-04 | 64.89 |  |
| Right-arm FFM | rs13001657 | | 88946611 | A | G | 0.294 | 0.010 | 0.001 | 9.90E-13 | 1.1E-04 | 50.86 |  |
| Right-arm FFM | rs74684058 | | 145616054 | T | C | 0.070 | 0.016 | 0.002 | 1.30E-10 | 9.1E-05 | 41.33 | BMI |
| Right-arm FFM | rs2140046 | | 169706079 | C | T | 0.364 | -0.011 | 0.001 | 2.70E-18 | 1.7E-04 | 76.07 |  |
| Right-arm FFM | rs2270894 | | 9975386 | G | C | 0.203 | -0.014 | 0.002 | 1.20E-19 | 1.8E-04 | 82.30 |  |
| Right-arm FFM | rs1470571 | | 77565844 | T | C | 0.715 | -0.008 | 0.001 | 6.60E-09 | 7.4E-05 | 33.64 |  |
| Right-arm FFM | rs2875907 | | 85518580 | G | A | 0.647 | -0.013 | 0.001 | 7.20E-25 | 2.3E-04 | 106.04 | BMI |
| Right-arm FFM | rs4858940 | | 88254820 | C | T | 0.886 | 0.016 | 0.002 | 5.20E-16 | 1.4E-04 | 65.71 | BMI |
| Right-arm FFM | rs6762578 | | 128992047 | A | G | 0.778 | 0.014 | 0.001 | 5.10E-22 | 2.0E-04 | 93.05 |  |
| Right-arm FFM | rs4407387 | | 152331086 | C | G | 0.343 | 0.008 | 0.001 | 4.70E-10 | 8.5E-05 | 38.80 |  |
| Right-arm FFM | rs2569993 | | 12926096 | C | T | 0.320 | 0.008 | 0.001 | 4.10E-09 | 7.6E-05 | 34.58 | BMI |
| Right-arm FFM | rs7619139 | | 25110415 | A | T | 0.589 | 0.010 | 0.001 | 2.40E-15 | 1.4E-04 | 62.67 | BMI |
| Right-arm FFM | rs7633464 | | 98715823 | A | G | 0.478 | 0.010 | 0.001 | 5.50E-15 | 1.3E-04 | 61.06 |  |
| Right-arm FFM | rs357486 | | 153885503 | C | T | 0.543 | 0.010 | 0.001 | 4.80E-15 | 1.3E-04 | 61.34 | BMI |
| Right-arm FFM | rs13097641 | | 13825666 | G | A | 0.359 | -0.007 | 0.001 | 9.20E-09 | 7.3E-05 | 33.00 |  |
| Right-arm FFM | rs6800021 | | 50190346 | A | G | 0.425 | 0.015 | 0.001 | 8.80E-32 | 3.0E-04 | 137.63 | BMI |
| Right-arm FFM | rs11717749 | | 52844733 | T | C | 0.115 | 0.015 | 0.002 | 2.40E-14 | 1.3E-04 | 58.16 | waist hip ratio |
| Right-arm FFM | rs17056859 | | 56535107 | A | G | 0.683 | 0.008 | 0.001 | 2.60E-09 | 7.8E-05 | 35.44 |  |
| Right-arm FFM | rs11925245 | | 114183579 | G | A | 0.183 | -0.009 | 0.002 | 1.10E-08 | 7.2E-05 | 32.72 |  |
| Right-arm FFM | rs724016 | | 141105570 | G | A | 0.444 | 0.027 | 0.001 | 6.00E-103 | 1.0E-03 | 464.16 | body fat percentage |
| Right-arm FFM | rs509035 | | 172163449 | A | G | 0.316 | 0.016 | 0.001 | 3.30E-34 | 3.3E-04 | 148.70 |  |
| Right-arm FFM | rs10084690 | | 33591384 | C | A | 0.035 | 0.021 | 0.003 | 2.50E-10 | 8.8E-05 | 39.99 |  |
| Right-arm FFM | rs3749387 | | 38496193 | C | G | 0.530 | 0.013 | 0.001 | 8.90E-24 | 2.2E-04 | 101.05 |  |
| Right-arm FFM | rs4974223 | | 56602088 | T | C | 0.904 | 0.015 | 0.002 | 3.90E-12 | 1.1E-04 | 48.19 |  |
| Right-arm FFM | rs75451531 | | 61187422 | T | C | 0.147 | -0.012 | 0.002 | 9.80E-12 | 1.0E-04 | 46.36 | BMI |
| Right-arm FFM | rs4677151 | | 72394027 | A | G | 0.505 | -0.008 | 0.001 | 9.10E-10 | 8.3E-05 | 37.51 |  |
| Right-arm FFM | rs1394046 | | 147059025 | T | A | 0.753 | -0.009 | 0.001 | 2.20E-10 | 8.9E-05 | 40.24 |  |
| Right-arm FFM | rs843374 | | 183997261 | T | A | 0.587 | -0.010 | 0.001 | 1.30E-14 | 1.3E-04 | 59.42 |  |
| Right-arm FFM | rs73175572 | | 185490184 | G | A | 0.112 | 0.025 | 0.002 | 1.80E-37 | 3.6E-04 | 163.66 |  |
| Right-arm FFM | rs11709402 | | 131551027 | G | A | 0.279 | 0.009 | 0.001 | 2.90E-11 | 9.7E-05 | 44.23 | BMI |
| Right-arm FFM | rs10936684 | | 171130793 | A | G | 0.660 | -0.008 | 0.001 | 9.00E-10 | 8.3E-05 | 37.54 | BMI |
| Right-arm FFM | rs9790159 | | 171908032 | G | A | 0.530 | -0.011 | 0.001 | 4.60E-17 | 1.5E-04 | 70.48 |  |
| Right-arm FFM | rs73052033 | | 185828465 | C | T | 0.185 | -0.014 | 0.002 | 2.10E-19 | 1.8E-04 | 81.10 | BMI |
| Right-arm FFM | rs6772164 | | 196078149 | A | C | 0.358 | 0.009 | 0.001 | 6.50E-12 | 1.0E-04 | 47.17 | BMI |
| Right-arm FFM | rs62325220 | | 54295227 | C | G | 0.149 | -0.014 | 0.002 | 5.90E-16 | 1.4E-04 | 65.46 |  |
| Right-arm FFM | rs10020631 | | 69353863 | A | G | 0.248 | -0.008 | 0.001 | 4.70E-08 | 6.6E-05 | 29.85 |  |
| Right-arm FFM | rs140493137 | | 87666916 | A | G | 0.060 | 0.019 | 0.003 | 3.80E-13 | 1.2E-04 | 52.75 |  |
| Right-arm FFM | rs2101975 | | 106216667 | G | A | 0.432 | -0.014 | 0.001 | 1.20E-27 | 2.6E-04 | 118.72 |  |
| Right-arm FFM | rs12498374 | | 111584419 | T | C | 0.205 | 0.009 | 0.002 | 1.70E-08 | 7.0E-05 | 31.82 |  |
| Right-arm FFM | rs1296328 | | 137083193 | C | A | 0.559 | -0.009 | 0.001 | 8.10E-12 | 1.0E-04 | 46.75 | BMI |
| Right-arm FFM | rs981002 | | 12881731 | A | T | 0.267 | -0.010 | 0.001 | 1.30E-12 | 1.1E-04 | 50.28 |  |
| Right-arm FFM | rs1472852 | | 17910236 | A | C | 0.159 | -0.029 | 0.002 | 2.30E-68 | 6.7E-04 | 305.30 | body fat percentage |
| Right-arm FFM | rs17556750 | | 82155568 | A | C | 0.288 | 0.012 | 0.001 | 9.20E-18 | 1.6E-04 | 73.67 |  |
| Right-arm FFM | rs1841738 | | 88557753 | G | A | 0.518 | -0.011 | 0.001 | 2.90E-19 | 1.8E-04 | 80.53 |  |
| Right-arm FFM | rs6824633 | | 111019965 | T | C | 0.353 | 0.007 | 0.001 | 1.80E-08 | 7.0E-05 | 31.66 |  |
| Right-arm FFM | rs11098675 | | 123832776 | G | A | 0.806 | -0.015 | 0.002 | 3.30E-21 | 2.0E-04 | 89.35 |  |
| Right-arm FFM | rs9985795 | | 135213286 | C | T | 0.483 | -0.008 | 0.001 | 1.30E-09 | 8.1E-05 | 36.79 |  |
| Right-arm FFM | rs111391498 | | 1341553 | G | A | 0.047 | -0.023 | 0.003 | 5.00E-16 | 1.4E-04 | 65.78 |  |
| Right-arm FFM | rs10222924 | | 49016761 | G | A | 0.699 | -0.010 | 0.001 | 1.10E-12 | 1.1E-04 | 50.61 |  |
| Right-arm FFM | rs11097755 | | 102709308 | C | T | 0.443 | 0.007 | 0.001 | 2.00E-09 | 7.9E-05 | 35.97 | BMI |
| Right-arm FFM | rs72703409 | | 184225295 | C | G | 0.066 | -0.014 | 0.002 | 2.90E-08 | 6.8E-05 | 30.79 |  |
| Right-arm FFM | rs73213484 | | 28489339 | T | A | 0.141 | -0.012 | 0.002 | 3.50E-11 | 9.7E-05 | 43.88 | BMI |
| Right-arm FFM | rs7669124 | | 31019816 | A | G | 0.366 | 0.009 | 0.001 | 4.10E-12 | 1.1E-04 | 48.07 | BMI |
| Right-arm FFM | rs10938398 | | 45186139 | A | G | 0.434 | 0.011 | 0.001 | 5.00E-20 | 1.8E-04 | 83.97 | BMI |
| Right-arm FFM | rs35339719 | | 57759537 | A | G | 0.280 | -0.008 | 0.001 | 2.30E-09 | 7.9E-05 | 35.73 |  |
| Right-arm FFM | rs4240326 | | 145839264 | G | A | 0.550 | -0.019 | 0.001 | 2.00E-55 | 5.4E-04 | 245.92 |  |
| Right-arm FFM | rs798759 | | 1730299 | G | A | 0.464 | -0.008 | 0.001 | 6.50E-12 | 1.0E-04 | 47.18 |  |
| Right-arm FFM | rs4426714 | | 4934599 | C | G | 0.378 | 0.009 | 0.001 | 2.20E-11 | 9.8E-05 | 44.77 |  |
| Right-arm FFM | rs6821305 | | 122713863 | C | A | 0.399 | 0.011 | 0.001 | 1.20E-19 | 1.8E-04 | 82.28 |  |
| Right-arm FFM | rs36089326 | | 160028812 | T | A | 0.327 | 0.008 | 0.001 | 6.00E-10 | 8.4E-05 | 38.32 |  |
| Right-arm FFM | rs11739036 | | 32723478 | A | G | 0.343 | -0.010 | 0.001 | 7.70E-15 | 1.3E-04 | 60.42 |  |
| Right-arm FFM | rs2307111 | | 75003678 | C | T | 0.395 | -0.016 | 0.001 | 6.10E-38 | 3.6E-04 | 165.79 | BMI |
| Right-arm FFM | rs9327336 | | 123990270 | C | T | 0.343 | 0.009 | 0.001 | 2.00E-11 | 9.9E-05 | 45.00 |  |
| Right-arm FFM | rs31210 | | 134361020 | A | G | 0.253 | -0.012 | 0.001 | 2.40E-16 | 1.5E-04 | 67.24 |  |
| Right-arm FFM | rs1990657 | | 171220503 | C | T | 0.428 | -0.008 | 0.001 | 6.00E-11 | 9.4E-05 | 42.82 |  |
| Right-arm FFM | rs6874142 | | 172753555 | G | T | 0.114 | 0.015 | 0.002 | 5.10E-13 | 1.1E-04 | 52.15 |  |
| Right-arm FFM | rs10058393 | | 277617 | T | C | 0.129 | 0.011 | 0.002 | 5.30E-09 | 7.5E-05 | 34.07 | body fat percentage |
| Right-arm FFM | rs12657771 | | 36787962 | A | G | 0.439 | -0.012 | 0.001 | 7.70E-22 | 2.0E-04 | 92.24 |  |
| Right-arm FFM | rs9291926 | | 67599656 | G | T | 0.532 | -0.011 | 0.001 | 7.80E-19 | 1.7E-04 | 78.56 |  |
| Right-arm FFM | rs10515237 | | 95751549 | G | A | 0.282 | 0.012 | 0.001 | 2.00E-19 | 1.8E-04 | 81.20 | BMI |
| Right-arm FFM | rs2952615 | | 112138888 | C | G | 0.618 | -0.011 | 0.001 | 1.80E-19 | 1.8E-04 | 81.44 |  |
| Right-arm FFM | rs1582931 | | 122657199 | A | G | 0.473 | -0.017 | 0.001 | 1.10E-40 | 3.9E-04 | 178.43 | BMI |
| Right-arm FFM | rs115053453 | | 130570345 | G | A | 0.161 | -0.012 | 0.002 | 3.90E-13 | 1.2E-04 | 52.68 |  |
| Right-arm FFM | rs2422054 | | 161382474 | A | T | 0.631 | 0.008 | 0.001 | 1.60E-10 | 9.0E-05 | 40.85 |  |
| Right-arm FFM | rs244711 | | 176509193 | T | C | 0.686 | 0.016 | 0.001 | 6.20E-28 | 2.6E-04 | 120.04 |  |
| Right-arm FFM | rs62372052 | | 42724294 | G | A | 0.110 | 0.025 | 0.002 | 1.00E-37 | 3.6E-04 | 164.77 |  |
| Right-arm FFM | rs12188627 | | 60720682 | G | A | 0.488 | -0.009 | 0.001 | 7.10E-14 | 1.2E-04 | 56.04 |  |
| Right-arm FFM | rs61749613 | | 82815170 | G | A | 0.041 | 0.027 | 0.003 | 7.90E-19 | 1.7E-04 | 78.53 | body fat percentage |
| Right-arm FFM | rs6898801 | | 111250615 | G | A | 0.658 | -0.008 | 0.001 | 1.10E-10 | 9.1E-05 | 41.55 |  |
| Right-arm FFM | rs3822742 | | 139059017 | A | C | 0.371 | 0.012 | 0.001 | 9.40E-20 | 1.8E-04 | 82.74 | BMI |
| Right-arm FFM | rs55758152 | | 171317318 | A | G | 0.326 | 0.009 | 0.001 | 1.90E-11 | 9.9E-05 | 45.03 |  |
| Right-arm FFM | rs10062079 | | 39393733 | A | G | 0.425 | 0.008 | 0.001 | 1.00E-09 | 8.2E-05 | 37.30 |  |
| Right-arm FFM | rs6450346 | | 55014771 | C | T | 0.701 | -0.011 | 0.001 | 9.00E-16 | 1.4E-04 | 64.64 |  |
| Right-arm FFM | rs9800418 | | 77641008 | C | T | 0.245 | 0.009 | 0.001 | 1.00E-09 | 8.2E-05 | 37.24 |  |
| Right-arm FFM | rs35267052 | | 87949118 | G | T | 0.103 | 0.014 | 0.002 | 5.20E-12 | 1.0E-04 | 47.62 | BMI |
| Right-arm FFM | rs7708460 | | 134579904 | A | G | 0.436 | -0.007 | 0.001 | 1.10E-08 | 7.2E-05 | 32.71 |  |
| Right-arm FFM | rs17115481 | | 153358226 | A | G | 0.270 | -0.008 | 0.001 | 1.30E-09 | 8.1E-05 | 36.87 | BMI |
| Right-arm FFM | rs252938 | | 5496480 | T | C | 0.649 | -0.009 | 0.001 | 1.50E-12 | 1.1E-04 | 50.10 |  |
| Right-arm FFM | rs10041126 | | 52813119 | C | A | 0.603 | 0.007 | 0.001 | 6.40E-09 | 7.4E-05 | 33.72 |  |
| Right-arm FFM | rs365352 | | 77401152 | A | G | 0.244 | -0.013 | 0.001 | 1.80E-20 | 1.9E-04 | 86.02 | BMI |
| Right-arm FFM | rs7442885 | | 87682877 | G | C | 0.214 | -0.016 | 0.002 | 7.10E-28 | 2.6E-04 | 119.77 | BMI |
| Right-arm FFM | rs6897407 | | 137798795 | C | G | 0.578 | -0.007 | 0.001 | 3.40E-09 | 7.7E-05 | 34.96 |  |
| Right-arm FFM | rs4282339 | | 168256240 | A | G | 0.208 | -0.018 | 0.002 | 1.70E-32 | 3.1E-04 | 140.86 |  |
| Right-arm FFM | rs111365325 | | 170865229 | T | C | 0.231 | -0.013 | 0.001 | 7.50E-19 | 1.7E-04 | 78.62 |  |
| Right-arm FFM | rs9379084 | | 7231843 | A | G | 0.116 | -0.016 | 0.002 | 1.50E-16 | 1.5E-04 | 68.14 |  |
| Right-arm FFM | rs2814993 | | 34618893 | A | G | 0.140 | 0.035 | 0.002 | 8.20E-87 | 8.6E-04 | 390.02 | BMI |
| Right-arm FFM | rs2395792 | | 41852187 | G | T | 0.956 | -0.027 | 0.003 | 5.80E-19 | 1.7E-04 | 79.14 |  |
| Right-arm FFM | rs114056237 | | 41877671 | A | G | 0.012 | -0.053 | 0.006 | 2.90E-21 | 2.0E-04 | 89.58 |  |
| Right-arm FFM | rs9350850 | | 81050236 | C | T | 0.080 | 0.022 | 0.002 | 9.30E-22 | 2.0E-04 | 91.87 |  |
| Right-arm FFM | rs10943915 | | 83617970 | T | A | 0.297 | 0.008 | 0.001 | 6.60E-09 | 7.4E-05 | 33.65 |  |
| Right-arm FFM | rs9372414 | | 97764299 | T | C | 0.348 | -0.007 | 0.001 | 3.20E-08 | 6.7E-05 | 30.59 | BMI |
| Right-arm FFM | rs7755185 | | 152339615 | G | A | 0.311 | 0.008 | 0.001 | 2.10E-09 | 7.9E-05 | 35.90 |  |
| Right-arm FFM | rs9380508 | | 35398689 | T | C | 0.775 | 0.011 | 0.001 | 5.80E-14 | 1.2E-04 | 56.43 |  |
| Right-arm FFM | rs72892910 | | 50816887 | T | G | 0.172 | 0.020 | 0.002 | 3.40E-33 | 3.2E-04 | 144.07 | BMI |
| Right-arm FFM | rs5019542 | | 51738352 | T | C | 0.636 | -0.007 | 0.001 | 1.50E-08 | 7.0E-05 | 32.00 | BMI |
| Right-arm FFM | rs180963 | | 101127213 | C | T | 0.512 | -0.008 | 0.001 | 1.80E-11 | 9.9E-05 | 45.18 |  |
| Right-arm FFM | rs9388446 | | 126064920 | A | T | 0.516 | 0.013 | 0.001 | 2.40E-27 | 2.6E-04 | 117.32 | BMI |
| Right-arm FFM | rs394487 | | 160778639 | T | C | 0.278 | 0.009 | 0.001 | 2.10E-11 | 9.9E-05 | 44.88 | BMI |
| Right-arm FFM | rs12216497 | | 19028623 | T | C | 0.561 | -0.011 | 0.001 | 5.10E-19 | 1.7E-04 | 79.38 |  |
| Right-arm FFM | rs62396185 | | 26180634 | C | G | 0.260 | -0.020 | 0.001 | 5.10E-47 | 4.6E-04 | 207.38 | BMI |
| Right-arm FFM | rs1418433 | | 44752568 | A | G | 0.588 | -0.008 | 0.001 | 1.30E-09 | 8.1E-05 | 36.78 |  |
| Right-arm FFM | rs578366 | | 81591034 | G | A | 0.420 | -0.010 | 0.001 | 4.10E-16 | 1.5E-04 | 66.21 |  |
| Right-arm FFM | rs9388498 | | 126873423 | T | G | 0.186 | 0.016 | 0.002 | 7.50E-23 | 2.1E-04 | 96.84 |  |
| Right-arm FFM | rs7740107 | | 130374461 | A | T | 0.736 | -0.028 | 0.001 | 6.50E-87 | 8.6E-04 | 390.50 |  |
| Right-arm FFM | rs603321 | | 140256669 | G | A | 0.244 | -0.013 | 0.001 | 8.00E-19 | 1.7E-04 | 78.50 |  |
| Right-arm FFM | rs2457982 | | 166313684 | A | G | 0.279 | 0.009 | 0.001 | 4.20E-11 | 9.6E-05 | 43.52 |  |
| Right-arm FFM | rs11243202 | | 7719065 | C | T | 0.486 | 0.018 | 0.001 | 2.50E-47 | 4.6E-04 | 208.82 |  |
| Right-arm FFM | rs7766034 | | 19753061 | T | C | 0.426 | -0.008 | 0.001 | 1.00E-10 | 9.2E-05 | 41.82 |  |
| Right-arm FFM | rs12175489 | | 31377587 | A | G | 0.119 | -0.014 | 0.002 | 1.40E-12 | 1.1E-04 | 50.16 |  |
| Right-arm FFM | rs62405860 | | 33459829 | C | T | 0.244 | 0.009 | 0.001 | 3.90E-11 | 9.6E-05 | 43.65 | BMI |
| Right-arm FFM | rs12211255 | | 76188330 | A | C | 0.104 | 0.014 | 0.002 | 2.40E-12 | 1.1E-04 | 49.13 |  |
| Right-arm FFM | rs17069647 | | 108917688 | A | T | 0.094 | -0.012 | 0.002 | 1.80E-08 | 7.0E-05 | 31.71 |  |
| Right-arm FFM | rs6570509 | | 142716286 | T | G | 0.287 | -0.013 | 0.001 | 1.50E-20 | 1.9E-04 | 86.40 |  |
| Right-arm FFM | rs3853252 | | 152170247 | A | G | 0.455 | 0.014 | 0.001 | 4.50E-28 | 2.7E-04 | 120.66 |  |
| Right-arm FFM | rs56017587 | | 18698092 | C | T | 0.137 | 0.011 | 0.002 | 5.30E-09 | 7.5E-05 | 34.07 |  |
| Right-arm FFM | rs41271299 | | 19839415 | T | C | 0.051 | 0.038 | 0.003 | 1.40E-41 | 4.0E-04 | 182.40 |  |
| Right-arm FFM | rs3130481 | | 31839756 | C | G | 0.513 | -0.015 | 0.001 | 2.20E-35 | 3.4E-04 | 154.14 |  |
| Right-arm FFM | rs4946936 | | 109003321 | C | T | 0.713 | 0.021 | 0.001 | 1.40E-50 | 4.9E-04 | 223.72 | BMI |
| Right-arm FFM | rs11153171 | | 109653825 | T | C | 0.356 | -0.012 | 0.001 | 6.80E-20 | 1.8E-04 | 83.37 |  |
| Right-arm FFM | rs6923431 | | 129824754 | C | A | 0.734 | 0.008 | 0.001 | 2.60E-08 | 6.8E-05 | 30.99 |  |
| Right-arm FFM | rs687694 | | 153472227 | G | T | 0.495 | -0.008 | 0.001 | 2.50E-10 | 8.8E-05 | 40.04 |  |
| Right-arm FFM | rs9457777 | | 160352921 | C | T | 0.228 | 0.010 | 0.001 | 8.50E-11 | 9.3E-05 | 42.14 |  |
| Right-arm FFM | rs3099282 | | 166584842 | G | A | 0.208 | -0.009 | 0.002 | 1.40E-09 | 8.1E-05 | 36.67 |  |
| Right-arm FFM | rs34776209 | | 23513093 | T | C | 0.247 | -0.015 | 0.001 | 4.10E-25 | 2.4E-04 | 107.17 |  |
| Right-arm FFM | rs3778934 | | 39445385 | C | A | 0.341 | -0.008 | 0.001 | 1.20E-09 | 8.1E-05 | 36.94 | BMI |
| Right-arm FFM | rs74876583 | | 50602539 | G | T | 0.117 | 0.016 | 0.002 | 1.80E-17 | 1.6E-04 | 72.38 |  |
| Right-arm FFM | rs10260993 | | 55884295 | G | T | 0.196 | -0.009 | 0.002 | 4.80E-08 | 6.6E-05 | 29.80 |  |
| Right-arm FFM | rs4285416 | | 92471899 | A | G | 0.166 | 0.010 | 0.002 | 4.50E-09 | 7.6E-05 | 34.41 |  |
| Right-arm FFM | rs7781021 | | 139715866 | C | T | 0.209 | 0.009 | 0.002 | 1.20E-09 | 8.1E-05 | 37.00 |  |
| Right-arm FFM | rs58687622 | | 6494765 | A | G | 0.141 | 0.011 | 0.002 | 4.60E-10 | 8.5E-05 | 38.83 |  |
| Right-arm FFM | rs7805694 | | 20437005 | T | C | 0.585 | 0.007 | 0.001 | 1.60E-08 | 7.0E-05 | 31.94 |  |
| Right-arm FFM | rs723149 | | 46577056 | G | A | 0.563 | -0.010 | 0.001 | 1.50E-16 | 1.5E-04 | 68.12 |  |
| Right-arm FFM | rs10269774 | | 92253972 | A | G | 0.326 | 0.023 | 0.001 | 1.30E-67 | 6.6E-04 | 301.89 |  |
| Right-arm FFM | rs1701827 | | 113040467 | G | A | 0.270 | -0.008 | 0.001 | 2.00E-08 | 6.9E-05 | 31.45 | BMI |
| Right-arm FFM | rs1403987 | | 38111941 | G | A | 0.654 | 0.008 | 0.001 | 1.80E-10 | 8.9E-05 | 40.66 |  |
| Right-arm FFM | rs13240065 | | 73015369 | A | G | 0.128 | 0.020 | 0.002 | 2.10E-26 | 2.5E-04 | 113.08 |  |
| Right-arm FFM | rs236628 | | 75023850 | T | G | 0.236 | 0.009 | 0.001 | 3.60E-10 | 8.6E-05 | 39.30 | BMI |
| Right-arm FFM | rs11525873 | | 138817193 | C | T | 0.098 | -0.014 | 0.002 | 3.40E-11 | 9.7E-05 | 43.94 | BMI |
| Right-arm FFM | rs115946508 | | 150497496 | A | C | 0.111 | -0.012 | 0.002 | 6.00E-10 | 8.4E-05 | 38.31 |  |
| Right-arm FFM | rs4725984 | | 150668514 | C | T | 0.642 | 0.018 | 0.001 | 1.10E-41 | 4.0E-04 | 182.89 | BMI |
| Right-arm FFM | rs6951209 | | 1273821 | T | A | 0.200 | -0.011 | 0.002 | 1.10E-11 | 1.0E-04 | 46.18 | BMI |
| Right-arm FFM | rs10242866 | | 17920613 | T | C | 0.399 | 0.008 | 0.001 | 2.20E-10 | 8.9E-05 | 40.31 |  |
| Right-arm FFM | rs508347 | | 28212824 | C | T | 0.703 | -0.011 | 0.001 | 4.90E-17 | 1.5E-04 | 70.39 |  |
| Right-arm FFM | rs7788672 | | 93139177 | C | G | 0.476 | 0.011 | 0.001 | 4.30E-18 | 1.7E-04 | 75.20 | BMI |
| Right-arm FFM | rs10243988 | | 99528761 | A | G | 0.195 | -0.009 | 0.002 | 2.50E-08 | 6.8E-05 | 31.04 |  |
| Right-arm FFM | rs39328 | | 103444978 | T | C | 0.425 | 0.009 | 0.001 | 8.70E-14 | 1.2E-04 | 55.64 | BMI |
| Right-arm FFM | rs10248298 | | 121963813 | A | C | 0.366 | 0.010 | 0.001 | 1.90E-14 | 1.3E-04 | 58.64 | BMI |
| Right-arm FFM | rs62621812 | | 127015083 | A | G | 0.020 | 0.040 | 0.005 | 1.80E-18 | 1.7E-04 | 76.87 | BMI |
| Right-arm FFM | rs1182207 | | 2810746 | A | G | 0.293 | -0.018 | 0.001 | 5.30E-39 | 3.8E-04 | 170.66 | body fat percentage |
| Right-arm FFM | rs10950207 | | 70105168 | T | C | 0.384 | 0.008 | 0.001 | 3.30E-09 | 7.7E-05 | 34.99 | BMI |
| Right-arm FFM | rs4729085 | | 76592670 | G | C | 0.868 | -0.015 | 0.002 | 1.50E-15 | 1.4E-04 | 63.58 | BMI |
| Right-arm FFM | rs822551 | | 148650375 | G | A | 0.812 | 0.011 | 0.002 | 3.00E-12 | 1.1E-04 | 48.72 |  |
| Right-arm FFM | rs17828687 | | 73454767 | A | C | 0.573 | -0.010 | 0.001 | 1.40E-14 | 1.3E-04 | 59.20 | BMI |
| Right-arm FFM | rs11997525 | | 49401982 | A | T | 0.167 | 0.015 | 0.002 | 5.20E-19 | 1.7E-04 | 79.36 |  |
| Right-arm FFM | rs62515438 | | 57161608 | G | T | 0.228 | 0.018 | 0.001 | 2.00E-34 | 3.3E-04 | 149.68 |  |
| Right-arm FFM | rs61729527 | | 77761919 | T | C | 0.052 | -0.020 | 0.003 | 5.10E-13 | 1.1E-04 | 52.16 | BMI |
| Right-arm FFM | rs7842996 | | 78107140 | A | T | 0.284 | 0.017 | 0.001 | 3.00E-34 | 3.3E-04 | 148.93 |  |
| Right-arm FFM | rs55674305 | | 89435868 | A | G | 0.306 | -0.010 | 0.001 | 7.50E-13 | 1.1E-04 | 51.40 | BMI |
| Right-arm FFM | rs12680965 | | 95566297 | G | T | 0.235 | -0.011 | 0.001 | 2.10E-14 | 1.3E-04 | 58.39 |  |
| Right-arm FFM | rs3925 | | 38281658 | A | G | 0.245 | -0.009 | 0.001 | 1.00E-09 | 8.2E-05 | 37.28 |  |
| Right-arm FFM | rs72656010 | | 57122215 | C | T | 0.132 | -0.032 | 0.002 | 8.50E-69 | 6.8E-04 | 307.29 |  |
| Right-arm FFM | rs11778491 | | 120451362 | C | G | 0.252 | -0.011 | 0.001 | 8.40E-16 | 1.4E-04 | 64.77 |  |
| Right-arm FFM | rs35309034 | | 126498265 | A | C | 0.238 | -0.012 | 0.001 | 3.00E-15 | 1.4E-04 | 62.24 |  |
| Right-arm FFM | rs12679359 | | 130724556 | T | G | 0.137 | -0.013 | 0.002 | 3.40E-13 | 1.2E-04 | 52.96 |  |
| Right-arm FFM | rs1057626 | | 10962800 | C | T | 0.520 | -0.009 | 0.001 | 8.20E-14 | 1.2E-04 | 55.76 | BMI |
| Right-arm FFM | rs2721938 | | 116635611 | T | C | 0.600 | -0.011 | 0.001 | 2.90E-19 | 1.8E-04 | 80.51 |  |
| Right-arm FFM | rs10283100 | | 120596023 | G | A | 0.945 | 0.025 | 0.003 | 2.40E-20 | 1.9E-04 | 85.44 |  |
| Right-arm FFM | rs10107388 | | 145004944 | C | T | 0.369 | -0.012 | 0.001 | 6.10E-21 | 1.9E-04 | 88.15 |  |
| Right-arm FFM | rs12545613 | | 6325499 | T | C | 0.135 | 0.011 | 0.002 | 2.40E-09 | 7.8E-05 | 35.58 |  |
| Right-arm FFM | rs7824070 | | 23136793 | T | C | 0.673 | 0.009 | 0.001 | 3.00E-11 | 9.7E-05 | 44.16 |  |
| Right-arm FFM | rs17089329 | | 23390046 | G | C | 0.203 | 0.011 | 0.002 | 3.90E-13 | 1.2E-04 | 52.68 |  |
| Right-arm FFM | rs55796651 | | 25349240 | T | C | 0.257 | -0.009 | 0.001 | 1.50E-09 | 8.0E-05 | 36.49 |  |
| Right-arm FFM | rs13264909 | | 64702385 | T | A | 0.429 | -0.007 | 0.001 | 9.10E-09 | 7.3E-05 | 33.02 | BMI |
| Right-arm FFM | rs4909912 | | 135572297 | T | C | 0.426 | -0.018 | 0.001 | 3.00E-46 | 4.5E-04 | 203.87 |  |
| Right-arm FFM | rs1412234 | | 28410683 | C | T | 0.327 | 0.010 | 0.001 | 2.30E-14 | 1.3E-04 | 58.27 | BMI |
| Right-arm FFM | rs10119967 | | 99268421 | C | A | 0.204 | 0.014 | 0.002 | 5.60E-19 | 1.7E-04 | 79.21 |  |
| Right-arm FFM | rs1341215 | | 111662350 | A | G | 0.137 | 0.011 | 0.002 | 1.90E-09 | 7.9E-05 | 36.02 |  |
| Right-arm FFM | rs4073153 | | 139259349 | G | A | 0.439 | 0.007 | 0.001 | 6.00E-09 | 7.4E-05 | 33.83 |  |
| Right-arm FFM | rs2295794 | | 35700150 | T | C | 0.406 | -0.008 | 0.001 | 1.70E-09 | 8.0E-05 | 36.26 |  |
| Right-arm FFM | rs734764 | | 90852624 | C | G | 0.789 | -0.009 | 0.002 | 2.70E-09 | 7.8E-05 | 35.39 |  |
| Right-arm FFM | rs663344 | | 98316331 | C | G | 0.181 | -0.013 | 0.002 | 4.50E-16 | 1.5E-04 | 66.02 |  |
| Right-arm FFM | rs7033487 | | 119129257 | C | T | 0.198 | -0.021 | 0.002 | 1.40E-41 | 4.0E-04 | 182.42 |  |
| Right-arm FFM | rs13283416 | | 119301607 | G | T | 0.427 | 0.010 | 0.001 | 8.20E-17 | 1.5E-04 | 69.37 |  |
| Right-arm FFM | rs10986292 | | 127016876 | G | T | 0.339 | 0.011 | 0.001 | 9.50E-16 | 1.4E-04 | 64.54 |  |
| Right-arm FFM | rs536171 | | 129653677 | C | T | 0.717 | 0.011 | 0.001 | 1.90E-15 | 1.4E-04 | 63.16 |  |
| Right-arm FFM | rs10820852 | | 94186973 | A | C | 0.276 | -0.011 | 0.001 | 1.80E-16 | 1.5E-04 | 67.83 | BMI |
| Right-arm FFM | rs5015933 | | 128137418 | C | T | 0.456 | -0.009 | 0.001 | 1.00E-12 | 1.1E-04 | 50.76 | BMI |
| Right-arm FFM | rs1927635 | | 16461905 | C | T | 0.354 | 0.009 | 0.001 | 2.20E-12 | 1.1E-04 | 49.30 |  |
| Right-arm FFM | rs10811668 | | 22164991 | A | C | 0.207 | -0.008 | 0.002 | 4.80E-08 | 6.6E-05 | 29.79 |  |
| Right-arm FFM | rs11142700 | | 73757155 | C | T | 0.408 | -0.008 | 0.001 | 1.00E-10 | 9.2E-05 | 41.80 |  |
| Right-arm FFM | rs28620532 | | 98216876 | G | A | 0.349 | 0.016 | 0.001 | 2.40E-34 | 3.3E-04 | 149.37 |  |
| Right-arm FFM | rs11794152 | | 23345347 | G | A | 0.415 | 0.009 | 0.001 | 6.10E-12 | 1.0E-04 | 47.29 |  |
| Right-arm FFM | rs773145 | | 77668969 | C | G | 0.416 | -0.008 | 0.001 | 7.30E-10 | 8.3E-05 | 37.95 |  |
| Right-arm FFM | rs7023690 | | 102623328 | A | C | 0.555 | -0.008 | 0.001 | 1.10E-09 | 8.2E-05 | 37.07 |  |
| Right-arm FFM | rs12344515 | | 113801231 | T | C | 0.240 | -0.009 | 0.001 | 3.20E-10 | 8.7E-05 | 39.53 |  |
| Right-arm FFM | rs10453255 | | 137264712 | G | A | 0.222 | -0.010 | 0.001 | 1.30E-10 | 9.1E-05 | 41.24 |  |
| Right-arm FFM | rs11592463 | | 34518442 | A | G | 0.195 | -0.009 | 0.002 | 1.90E-09 | 7.9E-05 | 36.08 |  |
| Right-arm FFM | rs117543413 | | 79543740 | T | C | 0.018 | -0.033 | 0.005 | 2.70E-12 | 1.1E-04 | 48.89 |  |
| Right-arm FFM | rs332115 | | 28926054 | A | G | 0.280 | -0.008 | 0.001 | 4.10E-09 | 7.6E-05 | 34.57 |  |
| Right-arm FFM | rs1316312 | | 81110417 | C | G | 0.500 | -0.010 | 0.001 | 2.40E-15 | 1.4E-04 | 62.73 |  |
| Right-arm FFM | rs7910211 | | 81228378 | C | T | 0.159 | 0.010 | 0.002 | 4.90E-09 | 7.5E-05 | 34.21 |  |
| Right-arm FFM | rs73601548 | | 18549889 | T | C | 0.115 | 0.014 | 0.002 | 1.30E-12 | 1.1E-04 | 50.27 | BMI |
| Right-arm FFM | rs1890951 | | 62054706 | G | A | 0.620 | 0.007 | 0.001 | 2.30E-08 | 6.9E-05 | 31.19 | BMI |
| Right-arm FFM | rs2902406 | | 102660281 | C | G | 0.432 | 0.015 | 0.001 | 3.80E-33 | 3.2E-04 | 143.89 |  |
| Right-arm FFM | rs2265309 | | 104487871 | C | T | 0.517 | -0.012 | 0.001 | 1.20E-22 | 2.1E-04 | 95.88 |  |
| Right-arm FFM | rs11593630 | | 120491506 | T | C | 0.353 | -0.008 | 0.001 | 3.20E-10 | 8.7E-05 | 39.57 |  |
| Right-arm FFM | rs11245450 | | 126658075 | A | G | 0.422 | -0.010 | 0.001 | 9.40E-16 | 1.4E-04 | 64.55 | BMI |
| Right-arm FFM | rs57866767 | | 96023077 | C | T | 0.436 | 0.012 | 0.001 | 4.60E-21 | 1.9E-04 | 88.69 |  |
| Right-arm FFM | rs75044513 | | 121139812 | C | T | 0.100 | 0.012 | 0.002 | 1.40E-09 | 8.1E-05 | 36.62 |  |
| Right-arm FFM | rs75406471 | | 5257647 | A | G | 0.154 | 0.014 | 0.002 | 2.40E-15 | 1.4E-04 | 62.72 |  |
| Right-arm FFM | rs10740991 | | 22058137 | C | G | 0.718 | -0.010 | 0.001 | 9.10E-14 | 1.2E-04 | 55.55 | BMI |
| Right-arm FFM | rs11014285 | | 25178864 | A | G | 0.165 | 0.017 | 0.002 | 5.70E-25 | 2.3E-04 | 106.50 |  |
| Right-arm FFM | rs61849823 | | 53290991 | C | T | 0.164 | 0.011 | 0.002 | 5.60E-11 | 9.5E-05 | 42.96 |  |
| Right-arm FFM | rs3740591 | | 70287303 | T | C | 0.445 | 0.011 | 0.001 | 2.50E-18 | 1.7E-04 | 76.24 |  |
| Right-arm FFM | rs4255484 | | 77215583 | G | C | 0.559 | -0.012 | 0.001 | 7.80E-21 | 1.9E-04 | 87.64 |  |
| Right-arm FFM | rs146498112 | | 98657257 | G | C | 0.014 | -0.034 | 0.005 | 2.30E-10 | 8.8E-05 | 40.21 |  |
| Right-arm FFM | rs3862012 | | 114682553 | G | A | 0.516 | -0.007 | 0.001 | 1.00E-08 | 7.2E-05 | 32.77 |  |
| Right-arm FFM | rs4752689 | | 124131176 | A | G | 0.584 | 0.011 | 0.001 | 1.50E-17 | 1.6E-04 | 72.65 |  |
| Right-arm FFM | rs77603146 | | 126200090 | A | G | 0.111 | 0.011 | 0.002 | 3.70E-08 | 6.7E-05 | 30.29 |  |
| Right-arm FFM | rs10832961 | | 18653957 | G | C | 0.758 | -0.009 | 0.001 | 7.70E-10 | 8.3E-05 | 37.84 |  |
| Right-arm FFM | rs78287937 | | 64773101 | G | T | 0.090 | 0.013 | 0.002 | 6.40E-10 | 8.4E-05 | 38.20 | BMI |
| Right-arm FFM | rs2450128 | | 77940075 | A | G | 0.152 | -0.011 | 0.002 | 8.30E-10 | 8.3E-05 | 37.69 |  |
| Right-arm FFM | rs11217843 | | 120239937 | G | A | 0.163 | -0.011 | 0.002 | 1.00E-10 | 9.2E-05 | 41.74 |  |
| Right-arm FFM | rs11042725 | | 10325325 | A | C | 0.488 | -0.011 | 0.001 | 6.10E-18 | 1.6E-04 | 74.48 | body fat percentage |
| Right-arm FFM | rs4922819 | | 29349419 | G | A | 0.304 | 0.009 | 0.001 | 4.60E-11 | 9.5E-05 | 43.36 |  |
| Right-arm FFM | rs35251247 | | 43878459 | A | G | 0.292 | 0.011 | 0.001 | 4.60E-15 | 1.4E-04 | 61.43 | BMI |
| Right-arm FFM | rs7952436 | | 67024534 | T | C | 0.082 | -0.032 | 0.002 | 3.00E-46 | 4.5E-04 | 203.88 |  |
| Right-arm FFM | rs1813212 | | 89301382 | G | A | 0.446 | -0.007 | 0.001 | 1.60E-08 | 7.0E-05 | 31.94 |  |
| Right-arm FFM | rs35506085 | | 2165576 | A | G | 0.185 | -0.018 | 0.002 | 3.60E-29 | 2.8E-04 | 125.70 |  |
| Right-arm FFM | rs4929924 | | 8639325 | A | G | 0.645 | 0.008 | 0.001 | 1.80E-09 | 8.0E-05 | 36.16 | BMI |
| Right-arm FFM | rs6265 | | 27679916 | T | C | 0.188 | -0.020 | 0.002 | 2.70E-36 | 3.5E-04 | 158.24 | BMI |
| Right-arm FFM | rs1228024 | | 47951353 | A | C | 0.661 | -0.009 | 0.001 | 5.50E-13 | 1.1E-04 | 52.02 |  |
| Right-arm FFM | rs667515 | | 69449076 | C | G | 0.386 | -0.008 | 0.001 | 4.10E-11 | 9.6E-05 | 43.56 | BMI |
| Right-arm FFM | rs676105 | | 30443688 | C | T | 0.304 | 0.012 | 0.001 | 6.00E-19 | 1.7E-04 | 79.07 | BMI |
| Right-arm FFM | rs7129320 | | 68388220 | A | G | 0.166 | -0.021 | 0.002 | 1.30E-35 | 3.4E-04 | 155.19 |  |
| Right-arm FFM | rs7949333 | | 69916746 | T | C | 0.458 | -0.008 | 0.001 | 7.20E-11 | 9.3E-05 | 42.46 |  |
| Right-arm FFM | rs11049684 | | 28665359 | T | C | 0.299 | -0.010 | 0.001 | 3.60E-14 | 1.3E-04 | 57.40 |  |
| Right-arm FFM | rs3730071 | | 49168798 | A | C | 0.030 | -0.020 | 0.004 | 2.30E-08 | 6.9E-05 | 31.24 | body fat percentage |
| Right-arm FFM | rs12831185 | | 53860781 | G | A | 0.170 | -0.012 | 0.002 | 1.60E-12 | 1.1E-04 | 49.93 |  |
| Right-arm FFM | rs1949549 | | 90170056 | C | T | 0.722 | 0.008 | 0.001 | 5.40E-09 | 7.5E-05 | 34.05 | BMI |
| Right-arm FFM | rs9634212 | | 93993266 | A | C | 0.221 | 0.022 | 0.001 | 9.20E-50 | 4.8E-04 | 219.96 |  |
| Right-arm FFM | rs34118426 | | 94201279 | G | A | 0.304 | -0.011 | 0.001 | 1.20E-16 | 1.5E-04 | 68.68 |  |
| Right-arm FFM | rs11111302 | | 102959705 | T | C | 0.153 | 0.015 | 0.002 | 9.90E-19 | 1.7E-04 | 78.09 |  |
| Right-arm FFM | rs9739533 | | 122475916 | A | G | 0.876 | -0.012 | 0.002 | 3.10E-11 | 9.7E-05 | 44.11 |  |
| Right-arm FFM | rs55726687 | | 991306 | A | G | 0.210 | 0.015 | 0.002 | 6.80E-22 | 2.0E-04 | 92.48 | BMI |
| Right-arm FFM | rs117451679 | | 17245591 | G | A | 0.106 | 0.012 | 0.002 | 5.40E-09 | 7.5E-05 | 34.03 |  |
| Right-arm FFM | rs7134283 | | 24071748 | A | G | 0.283 | -0.011 | 0.001 | 8.20E-17 | 1.5E-04 | 69.35 |  |
| Right-arm FFM | rs10843397 | | 29529523 | T | C | 0.242 | 0.008 | 0.001 | 1.70E-08 | 7.0E-05 | 31.86 |  |
| Right-arm FFM | rs1351394 | | 66351826 | C | T | 0.512 | -0.022 | 0.001 | 9.70E-74 | 7.3E-04 | 329.98 |  |
| Right-arm FFM | rs10878984 | | 69828534 | T | C | 0.345 | 0.010 | 0.001 | 1.70E-14 | 1.3E-04 | 58.89 |  |
| Right-arm FFM | rs2291256 | | 133393323 | T | C | 0.088 | 0.015 | 0.002 | 2.20E-12 | 1.1E-04 | 49.33 |  |
| Right-arm FFM | rs2733287 | | 41880909 | C | A | 0.489 | 0.008 | 0.001 | 1.90E-10 | 8.9E-05 | 40.55 | BMI |
| Right-arm FFM | rs3782232 | | 57116249 | A | G | 0.071 | -0.018 | 0.002 | 5.60E-14 | 1.2E-04 | 56.50 |  |
| Right-arm FFM | rs58035271 | | 66127644 | C | G | 0.243 | -0.010 | 0.001 | 3.50E-13 | 1.2E-04 | 52.92 |  |
| Right-arm FFM | rs310796 | | 77453226 | T | G | 0.681 | 0.010 | 0.001 | 2.00E-14 | 1.3E-04 | 58.49 |  |
| Right-arm FFM | rs11065979 | | 112059557 | T | C | 0.438 | -0.014 | 0.001 | 4.70E-29 | 2.8E-04 | 125.17 |  |
| Right-arm FFM | rs696343 | | 116304907 | G | A | 0.116 | -0.011 | 0.002 | 2.40E-08 | 6.8E-05 | 31.10 |  |
| Right-arm FFM | rs147786161 | | 122583781 | G | A | 0.437 | 0.010 | 0.001 | 1.00E-15 | 1.4E-04 | 64.43 | BMI |
| Right-arm FFM | rs1271309 | | 124820705 | G | A | 0.838 | -0.018 | 0.002 | 1.20E-25 | 2.4E-04 | 109.61 |  |
| Right-arm FFM | rs35756741 | | 12868701 | T | C | 0.092 | -0.013 | 0.002 | 9.00E-10 | 8.3E-05 | 37.53 |  |
| Right-arm FFM | rs7961994 | | 46755112 | T | A | 0.610 | 0.014 | 0.001 | 9.50E-29 | 2.7E-04 | 123.75 |  |
| Right-arm FFM | rs7132908 | | 50263148 | A | G | 0.384 | 0.015 | 0.001 | 6.10E-31 | 2.9E-04 | 133.79 | BMI |
| Right-arm FFM | rs73180805 | | 103036662 | G | A | 0.157 | -0.011 | 0.002 | 8.10E-11 | 9.3E-05 | 42.23 |  |
| Right-arm FFM | rs10861678 | | 107319661 | A | G | 0.266 | -0.010 | 0.001 | 1.30E-12 | 1.1E-04 | 50.27 |  |
| Right-arm FFM | rs11065015 | | 120520863 | T | C | 0.027 | -0.025 | 0.004 | 1.80E-10 | 9.0E-05 | 40.70 | body fat percentage |
| Right-arm FFM | rs1473553 | | 122985057 | T | G | 0.745 | 0.016 | 0.001 | 5.90E-28 | 2.6E-04 | 120.13 | BMI |
| Right-arm FFM | rs11833839 | | 132560957 | T | C | 0.057 | 0.016 | 0.003 | 2.30E-09 | 7.9E-05 | 35.69 |  |
| Right-arm FFM | rs67551338 | | 3393100 | T | C | 0.061 | 0.021 | 0.003 | 1.60E-15 | 1.4E-04 | 63.53 |  |
| Right-arm FFM | rs76895963 | | 4384844 | G | T | 0.021 | 0.086 | 0.005 | 5.00E-72 | 7.1E-04 | 322.14 | BMI |
| Right-arm FFM | rs2900208 | | 11878464 | A | C | 0.354 | 0.014 | 0.001 | 4.40E-28 | 2.7E-04 | 120.70 |  |
| Right-arm FFM | rs774214 | | 56918566 | C | T | 0.663 | -0.008 | 0.001 | 3.30E-09 | 7.7E-05 | 34.98 |  |
| Right-arm FFM | rs7305516 | | 102382052 | G | A | 0.494 | -0.011 | 0.001 | 9.40E-18 | 1.6E-04 | 73.64 |  |
| Right-arm FFM | rs11618507 | | 30172751 | T | G | 0.226 | 0.010 | 0.001 | 5.40E-12 | 1.0E-04 | 47.55 |  |
| Right-arm FFM | rs7321045 | | 97034410 | A | G | 0.449 | 0.009 | 0.001 | 2.60E-12 | 1.1E-04 | 49.00 | BMI |
| Right-arm FFM | rs1889775 | | 110595596 | G | T | 0.081 | 0.013 | 0.002 | 2.20E-08 | 6.9E-05 | 31.30 |  |
| Right-arm FFM | rs77079139 | | 40769698 | A | G | 0.129 | 0.011 | 0.002 | 1.10E-08 | 7.2E-05 | 32.59 |  |
| Right-arm FFM | rs146851424 | | 50377910 | C | A | 0.022 | 0.051 | 0.004 | 6.30E-33 | 3.1E-04 | 142.86 |  |
| Right-arm FFM | rs1576981 | | 65930367 | A | G | 0.455 | -0.007 | 0.001 | 4.30E-08 | 6.6E-05 | 29.99 | BMI |
| Right-arm FFM | rs1924936 | | 78443297 | A | T | 0.774 | 0.013 | 0.001 | 3.00E-18 | 1.7E-04 | 75.87 |  |
| Right-arm FFM | rs1176315 | | 81221665 | C | T | 0.550 | -0.008 | 0.001 | 9.90E-10 | 8.2E-05 | 37.34 |  |
| Right-arm FFM | rs11616283 | | 21493853 | C | T | 0.140 | 0.011 | 0.002 | 3.10E-09 | 7.7E-05 | 35.11 |  |
| Right-arm FFM | rs2225226 | | 51127270 | T | C | 0.216 | -0.025 | 0.002 | 6.10E-63 | 6.2E-04 | 280.42 |  |
| Right-arm FFM | rs9540493 | | 66205704 | G | A | 0.545 | -0.007 | 0.001 | 4.30E-09 | 7.6E-05 | 34.48 | BMI |
| Right-arm FFM | rs9513510 | | 99571922 | C | G | 0.699 | -0.010 | 0.001 | 7.00E-13 | 1.1E-04 | 51.55 |  |
| Right-arm FFM | rs1967772 | | 28036062 | A | G | 0.285 | -0.010 | 0.001 | 2.10E-12 | 1.1E-04 | 49.38 | BMI |
| Right-arm FFM | rs67141907 | | 42805360 | T | C | 0.149 | 0.011 | 0.002 | 4.30E-11 | 9.6E-05 | 43.48 |  |
| Right-arm FFM | rs9596810 | | 54056553 | T | C | 0.435 | -0.009 | 0.001 | 7.70E-12 | 1.0E-04 | 46.83 | BMI |
| Right-arm FFM | rs9317002 | | 59175727 | A | C | 0.515 | 0.010 | 0.001 | 1.40E-16 | 1.5E-04 | 68.37 | BMI |
| Right-arm FFM | rs10870597 | | 114999636 | G | A | 0.235 | -0.010 | 0.001 | 6.70E-11 | 9.4E-05 | 42.61 |  |
| Right-arm FFM | rs875908 | | 23907608 | G | C | 0.642 | 0.008 | 0.001 | 6.90E-10 | 8.4E-05 | 38.04 |  |
| Right-arm FFM | rs3212260 | | 24804088 | T | A | 0.258 | 0.011 | 0.001 | 4.70E-16 | 1.4E-04 | 65.93 |  |
| Right-arm FFM | rs1652946 | | 34655995 | T | A | 0.512 | 0.008 | 0.001 | 9.80E-10 | 8.2E-05 | 37.37 |  |
| Right-arm FFM | rs12879453 | | 47309879 | G | A | 0.509 | -0.008 | 0.001 | 1.10E-11 | 1.0E-04 | 46.23 | BMI |
| Right-arm FFM | rs7141420 | | 79899454 | T | C | 0.516 | 0.012 | 0.001 | 8.70E-22 | 2.0E-04 | 91.99 | BMI |
| Right-arm FFM | rs12879423 | | 25927832 | G | A | 0.679 | 0.015 | 0.001 | 8.00E-28 | 2.6E-04 | 119.53 | BMI |
| Right-arm FFM | rs12881869 | | 50923249 | T | C | 0.071 | -0.017 | 0.002 | 1.20E-12 | 1.1E-04 | 50.45 |  |
| Right-arm FFM | rs4899012 | | 61003889 | C | G | 0.607 | -0.019 | 0.001 | 3.10E-50 | 4.9E-04 | 222.14 |  |
| Right-arm FFM | rs61992671 | | 101531854 | G | A | 0.492 | -0.010 | 0.001 | 1.30E-15 | 1.4E-04 | 63.86 | BMI |
| Right-arm FFM | rs56130943 | | 105906522 | C | A | 0.233 | 0.010 | 0.002 | 5.90E-11 | 9.4E-05 | 42.86 |  |
| Right-arm FFM | rs10141392 | | 35672013 | T | A | 0.098 | -0.013 | 0.002 | 1.40E-10 | 9.0E-05 | 41.12 | BMI |
| Right-arm FFM | rs7150606 | | 68413076 | T | G | 0.142 | -0.010 | 0.002 | 4.10E-08 | 6.6E-05 | 30.09 |  |
| Right-arm FFM | rs11160601 | | 101186641 | T | C | 0.092 | 0.016 | 0.002 | 3.40E-14 | 1.3E-04 | 57.47 | BMI |
| Right-arm FFM | rs2332175 | | 70345411 | A | G | 0.547 | -0.007 | 0.001 | 4.80E-09 | 7.5E-05 | 34.29 |  |
| Right-arm FFM | rs6575340 | | 94023972 | A | G | 0.636 | 0.008 | 0.001 | 4.50E-11 | 9.5E-05 | 43.40 | BMI |
| Right-arm FFM | rs4906203 | | 102928991 | T | C | 0.234 | -0.008 | 0.001 | 4.70E-08 | 6.6E-05 | 29.84 |  |
| Right-arm FFM | rs1135641 | | 23299135 | T | G | 0.531 | -0.007 | 0.001 | 3.30E-09 | 7.7E-05 | 35.00 |  |
| Right-arm FFM | rs2296316 | | 65520246 | C | T | 0.465 | -0.010 | 0.001 | 1.70E-14 | 1.3E-04 | 58.90 |  |
| Right-arm FFM | rs112560164 | | 93112924 | A | G | 0.191 | 0.011 | 0.002 | 2.10E-11 | 9.9E-05 | 44.89 |  |
| Right-arm FFM | rs4145824 | | 103271600 | A | G | 0.653 | -0.008 | 0.001 | 5.20E-10 | 8.5E-05 | 38.58 | BMI |
| Right-arm FFM | rs3809570 | | 67000117 | A | C | 0.243 | 0.014 | 0.001 | 1.90E-21 | 2.0E-04 | 90.48 | body fat percentage |
| Right-arm FFM | rs16942324 | | 89383854 | A | C | 0.027 | -0.035 | 0.004 | 1.60E-20 | 1.9E-04 | 86.22 |  |
| Right-arm FFM | rs11071182 | | 55644676 | G | A | 0.870 | 0.011 | 0.002 | 5.60E-10 | 8.5E-05 | 38.47 |  |
| Right-arm FFM | rs933807 | | 62274940 | C | G | 0.453 | -0.010 | 0.001 | 4.20E-17 | 1.6E-04 | 70.69 |  |
| Right-arm FFM | rs380803 | | 68161273 | C | T | 0.723 | 0.009 | 0.001 | 1.40E-11 | 1.0E-04 | 45.60 | BMI |
| Right-arm FFM | rs1521624 | | 79412399 | A | C | 0.471 | -0.008 | 0.001 | 1.10E-11 | 1.0E-04 | 46.18 | BMI |
| Right-arm FFM | rs1573891 | | 99186488 | C | G | 0.158 | -0.020 | 0.002 | 5.00E-31 | 3.0E-04 | 134.19 |  |
| Right-arm FFM | rs12906197 | | 38492199 | T | C | 0.422 | -0.010 | 0.001 | 1.80E-16 | 1.5E-04 | 67.81 |  |
| Right-arm FFM | rs74841302 | | 42084329 | A | G | 0.017 | -0.037 | 0.005 | 4.20E-15 | 1.4E-04 | 61.60 |  |
| Right-arm FFM | rs35874463 | | 67457698 | G | A | 0.058 | 0.015 | 0.003 | 5.90E-09 | 7.4E-05 | 33.86 |  |
| Right-arm FFM | rs8043280 | | 93425874 | G | C | 0.678 | -0.008 | 0.001 | 3.00E-10 | 8.7E-05 | 39.68 |  |
| Right-arm FFM | rs2663125 | | 99563891 | C | T | 0.691 | -0.008 | 0.001 | 4.40E-09 | 7.6E-05 | 34.43 |  |
| Right-arm FFM | rs12907384 | | 86276000 | C | T | 0.532 | -0.011 | 0.001 | 5.30E-18 | 1.6E-04 | 74.76 |  |
| Right-arm FFM | rs71385734 | | 2160503 | G | T | 0.170 | -0.022 | 0.002 | 5.30E-42 | 4.1E-04 | 184.39 | BMI |
| Right-arm FFM | rs7192870 | | 14397436 | C | T | 0.469 | -0.008 | 0.001 | 7.60E-11 | 9.3E-05 | 42.36 |  |
| Right-arm FFM | rs72774845 | | 15147954 | C | A | 0.307 | -0.011 | 0.001 | 1.00E-17 | 1.6E-04 | 73.45 | BMI |
| Right-arm FFM | rs7204864 | | 19942927 | T | C | 0.143 | -0.016 | 0.002 | 2.30E-19 | 1.8E-04 | 80.98 | BMI |
| Right-arm FFM | rs3751866 | | 84771304 | C | T | 0.247 | -0.008 | 0.001 | 4.40E-08 | 6.6E-05 | 29.97 |  |
| Right-arm FFM | rs8059189 | | 86417349 | A | G | 0.404 | -0.010 | 0.001 | 7.30E-16 | 1.4E-04 | 65.05 |  |
| Right-arm FFM | rs27345 | | 2267877 | A | G | 0.284 | 0.016 | 0.001 | 1.40E-31 | 3.0E-04 | 136.69 |  |
| Right-arm FFM | rs117342986 | | 54267868 | T | C | 0.026 | 0.022 | 0.004 | 3.10E-08 | 6.7E-05 | 30.67 | BMI |
| Right-arm FFM | rs34017457 | | 67166731 | A | G | 0.007 | 0.046 | 0.007 | 7.20E-10 | 8.4E-05 | 37.97 |  |
| Right-arm FFM | rs12443634 | | 81524274 | C | A | 0.714 | -0.009 | 0.001 | 3.70E-10 | 8.6E-05 | 39.27 |  |
| Right-arm FFM | rs10775348 | | 88806348 | G | A | 0.704 | 0.012 | 0.001 | 3.40E-18 | 1.7E-04 | 75.63 |  |
| Right-arm FFM | rs432925 | | 334580 | C | G | 0.283 | -0.008 | 0.001 | 4.80E-08 | 6.6E-05 | 29.79 | BMI |
| Right-arm FFM | rs7205337 | | 780339 | G | A | 0.208 | 0.012 | 0.002 | 3.50E-16 | 1.5E-04 | 66.52 |  |
| Right-arm FFM | rs4788218 | | 30055750 | C | T | 0.401 | 0.020 | 0.001 | 8.10E-56 | 5.4E-04 | 247.74 | BMI |
| Right-arm FFM | rs6500249 | | 49778147 | G | A | 0.735 | -0.009 | 0.001 | 3.90E-10 | 8.6E-05 | 39.17 |  |
| Right-arm FFM | rs3848364 | | 372635 | G | T | 0.266 | -0.010 | 0.001 | 1.00E-11 | 1.0E-04 | 46.33 |  |
| Right-arm FFM | rs2540034 | | 4022694 | T | C | 0.569 | 0.013 | 0.001 | 1.70E-25 | 2.4E-04 | 108.89 | BMI |
| Right-arm FFM | rs56094641 | | 53806453 | G | A | 0.405 | 0.034 | 0.001 | 8.39E-162 | 1.6E-03 | 734.73 | BMI |
| Right-arm FFM | rs2303083 | | 24835168 | A | G | 0.194 | -0.013 | 0.002 | 9.70E-16 | 1.4E-04 | 64.49 | BMI |
| Right-arm FFM | rs72801843 | | 53508802 | A | T | 0.301 | 0.013 | 0.001 | 1.60E-22 | 2.1E-04 | 95.34 |  |
| Right-arm FFM | rs34147411 | | 68081246 | T | C | 0.146 | 0.013 | 0.002 | 4.20E-13 | 1.2E-04 | 52.53 |  |
| Right-arm FFM | rs76513770 | | 72505534 | C | T | 0.128 | -0.015 | 0.002 | 6.20E-16 | 1.4E-04 | 65.36 |  |
| Right-arm FFM | rs112898929 | | 88298795 | A | T | 0.080 | -0.013 | 0.002 | 1.40E-08 | 7.1E-05 | 32.13 |  |
| Right-arm FFM | rs55831773 | | 7559037 | T | C | 0.199 | -0.012 | 0.002 | 3.40E-15 | 1.4E-04 | 61.99 |  |
| Right-arm FFM | rs2075058 | | 36906873 | G | A | 0.492 | 0.008 | 0.001 | 5.10E-11 | 9.5E-05 | 43.16 |  |
| Right-arm FFM | rs76238604 | | 42999702 | T | C | 0.144 | 0.010 | 0.002 | 4.60E-08 | 6.6E-05 | 29.90 |  |
| Right-arm FFM | rs758598 | | 59492714 | G | A | 0.669 | -0.015 | 0.001 | 4.50E-30 | 2.9E-04 | 129.83 |  |
| Right-arm FFM | rs2005172 | | 61996255 | C | A | 0.640 | 0.020 | 0.001 | 2.00E-54 | 5.3E-04 | 241.33 | body fat percentage |
| Right-arm FFM | rs1242507 | | 17365399 | A | G | 0.561 | 0.007 | 0.001 | 2.00E-08 | 6.9E-05 | 31.46 |  |
| Right-arm FFM | rs7223535 | | 29211667 | A | G | 0.270 | -0.022 | 0.001 | 3.40E-58 | 5.7E-04 | 258.61 |  |
| Right-arm FFM | rs9915532 | | 47027359 | G | A | 0.830 | -0.013 | 0.002 | 1.00E-14 | 1.3E-04 | 59.88 |  |
| Right-arm FFM | rs1401795 | | 54839652 | G | A | 0.501 | -0.007 | 0.001 | 4.40E-09 | 7.6E-05 | 34.45 |  |
| Right-arm FFM | rs236587 | | 68203915 | C | T | 0.737 | -0.008 | 0.001 | 1.30E-08 | 7.1E-05 | 32.36 |  |
| Right-arm FFM | rs78378222 | | 7571752 | G | T | 0.012 | 0.076 | 0.006 | 2.50E-40 | 3.9E-04 | 176.70 |  |
| Right-arm FFM | rs147107835 | | 30246737 | T | C | 0.004 | -0.055 | 0.010 | 2.90E-08 | 6.8E-05 | 30.75 |  |
| Right-arm FFM | rs35631456 | | 61784471 | T | C | 0.117 | -0.012 | 0.002 | 1.90E-10 | 8.9E-05 | 40.56 |  |
| Right-arm FFM | rs79680939 | | 80523375 | A | G | 0.045 | 0.017 | 0.003 | 1.40E-08 | 7.1E-05 | 32.20 |  |
| Right-arm FFM | rs11653850 | | 7236124 | A | G | 0.215 | 0.010 | 0.002 | 1.70E-11 | 1.0E-04 | 45.24 |  |
| Right-arm FFM | rs1044977 | | 43227214 | C | T | 0.254 | -0.013 | 0.001 | 3.00E-20 | 1.9E-04 | 84.99 |  |
| Right-arm FFM | rs173135 | | 68172326 | T | C | 0.115 | -0.016 | 0.002 | 2.70E-16 | 1.5E-04 | 66.98 |  |
| Right-arm FFM | rs77093479 | | 79429575 | G | C | 0.165 | -0.011 | 0.002 | 3.50E-11 | 9.7E-05 | 43.89 |  |
| Right-arm FFM | rs4968296 | | 45078593 | T | C | 0.749 | 0.008 | 0.001 | 3.80E-09 | 7.6E-05 | 34.72 |  |
| Right-arm FFM | rs114177791 | | 78555512 | T | G | 0.214 | 0.009 | 0.002 | 3.80E-10 | 8.6E-05 | 39.23 | BMI |
| Right-arm FFM | rs36000545 | | 79093822 | G | A | 0.396 | -0.015 | 0.001 | 1.00E-31 | 3.0E-04 | 137.38 |  |
| Right-arm FFM | rs57126421 | | 2656989 | G | A | 0.238 | -0.009 | 0.001 | 4.80E-10 | 8.5E-05 | 38.75 |  |
| Right-arm FFM | rs11663903 | | 32762592 | A | G | 0.429 | 0.007 | 0.001 | 2.60E-08 | 6.8E-05 | 31.01 |  |
| Right-arm FFM | rs8091287 | | 45753742 | C | T | 0.232 | -0.008 | 0.001 | 2.60E-08 | 6.8E-05 | 31.00 |  |
| Right-arm FFM | rs7229351 | | 46516374 | A | G | 0.368 | -0.009 | 0.001 | 5.60E-13 | 1.1E-04 | 51.97 |  |
| Right-arm FFM | rs11664336 | | 46604851 | T | A | 0.435 | 0.013 | 0.001 | 3.30E-24 | 2.3E-04 | 103.02 |  |
| Right-arm FFM | rs9951619 | | 56882326 | G | T | 0.767 | 0.011 | 0.001 | 9.50E-13 | 1.1E-04 | 50.93 | BMI |
| Right-arm FFM | rs6567160 | | 57829135 | C | T | 0.233 | 0.039 | 0.001 | 2.50E-158 | 1.6E-03 | 718.78 | BMI |
| Right-arm FFM | rs57636386 | | 58048295 | C | T | 0.084 | -0.020 | 0.002 | 1.40E-18 | 1.7E-04 | 77.35 | BMI |
| Right-arm FFM | rs1941697 | | 31251276 | A | G | 0.449 | 0.008 | 0.001 | 1.80E-11 | 9.9E-05 | 45.15 | BMI |
| Right-arm FFM | rs9960619 | | 33040095 | T | C | 0.344 | 0.008 | 0.001 | 4.90E-10 | 8.5E-05 | 38.71 |  |
| Right-arm FFM | rs59360013 | | 74976835 | T | C | 0.042 | -0.024 | 0.003 | 1.20E-14 | 1.3E-04 | 59.46 |  |
| Right-arm FFM | rs7237942 | | 20728049 | G | A | 0.789 | 0.025 | 0.002 | 3.10E-60 | 5.9E-04 | 268.03 |  |
| Right-arm FFM | rs12980774 | | 3410135 | C | T | 0.221 | 0.009 | 0.001 | 2.10E-09 | 7.9E-05 | 35.91 |  |
| Right-arm FFM | rs3843751 | | 10748121 | T | C | 0.664 | -0.012 | 0.001 | 1.20E-20 | 1.9E-04 | 86.73 |  |
| Right-arm FFM | rs62104476 | | 30294716 | A | G | 0.331 | 0.013 | 0.001 | 5.20E-23 | 2.1E-04 | 97.57 | BMI |
| Right-arm FFM | rs7245985 | | 30710410 | G | T | 0.208 | -0.012 | 0.002 | 2.20E-14 | 1.3E-04 | 58.38 |  |
| Right-arm FFM | rs285677 | | 34286666 | C | T | 0.671 | 0.010 | 0.001 | 6.90E-14 | 1.2E-04 | 56.09 |  |
| Right-arm FFM | rs3810291 | | 47569003 | A | G | 0.675 | 0.016 | 0.001 | 4.40E-32 | 3.1E-04 | 138.99 | BMI |
| Right-arm FFM | rs62136802 | | 47459476 | T | A | 0.313 | 0.008 | 0.001 | 2.60E-09 | 7.8E-05 | 35.43 |  |
| Right-arm FFM | rs281385 | | 49217261 | G | A | 0.879 | -0.013 | 0.002 | 2.00E-11 | 9.9E-05 | 45.01 |  |
| Right-arm FFM | rs147110934 | | 55993436 | T | G | 0.024 | -0.029 | 0.004 | 7.40E-13 | 1.1E-04 | 51.43 |  |
| Right-arm FFM | rs11880992 | | 2176403 | A | G | 0.408 | 0.013 | 0.001 | 6.50E-25 | 2.3E-04 | 106.26 |  |
| Right-arm FFM | rs10404726 | | 18834514 | T | C | 0.466 | -0.007 | 0.001 | 6.80E-09 | 7.4E-05 | 33.60 | BMI |
| Right-arm FFM | rs10432304 | | 4458242 | T | A | 0.560 | 0.008 | 0.001 | 9.90E-10 | 8.2E-05 | 37.34 |  |
| Right-arm FFM | rs1864000 | | 7110550 | G | T | 0.140 | -0.014 | 0.002 | 1.50E-14 | 1.3E-04 | 59.10 |  |
| Right-arm FFM | rs62621197 | | 8670147 | T | C | 0.037 | -0.027 | 0.003 | 6.50E-16 | 1.4E-04 | 65.27 | body fat percentage |
| Right-arm FFM | rs7245864 | | 38305660 | T | C | 0.542 | -0.007 | 0.001 | 3.00E-08 | 6.8E-05 | 30.72 |  |
| Right-arm FFM | rs10423120 | | 17422036 | G | A | 0.184 | -0.010 | 0.002 | 2.80E-10 | 8.8E-05 | 39.80 |  |
| Right-arm FFM | rs6136938 | | 20058992 | A | G | 0.436 | -0.009 | 0.001 | 2.10E-13 | 1.2E-04 | 53.90 |  |
| Right-arm FFM | rs34879158 | | 32300634 | C | A | 0.263 | -0.020 | 0.001 | 1.10E-43 | 4.2E-04 | 192.10 |  |
| Right-arm FFM | rs6512577 | | 47865784 | T | C | 0.220 | 0.009 | 0.001 | 4.20E-09 | 7.6E-05 | 34.52 | BMI |
| Right-arm FFM | rs4341996 | | 54377866 | C | A | 0.213 | -0.009 | 0.002 | 1.40E-08 | 7.1E-05 | 32.22 |  |
| Right-arm FFM | rs6142059 | | 32544327 | C | T | 0.492 | 0.009 | 0.001 | 6.70E-14 | 1.2E-04 | 56.14 | BMI |
| Right-arm FFM | rs143384 | | 34025756 | G | A | 0.404 | 0.031 | 0.001 | 1.80E-130 | 1.3E-03 | 590.63 | waist hip ratio |
| Right-arm FFM | rs55830103 | | 57465943 | G | T | 0.175 | -0.011 | 0.002 | 3.20E-11 | 9.7E-05 | 44.06 |  |
| Right-arm FFM | rs2650965 | | 6709838 | G | A | 0.330 | -0.009 | 0.001 | 1.00E-10 | 9.2E-05 | 41.74 |  |
| Right-arm FFM | rs16996657 | | 15816236 | C | T | 0.128 | 0.011 | 0.002 | 7.60E-09 | 7.3E-05 | 33.38 | BMI |
| Right-arm FFM | rs1411297 | | 6488806 | C | A | 0.653 | -0.010 | 0.001 | 5.20E-14 | 1.2E-04 | 56.65 | BMI |
| Right-arm FFM | rs1744769 | | 35769592 | C | T | 0.811 | 0.011 | 0.002 | 4.50E-13 | 1.2E-04 | 52.42 |  |
| Right-arm FFM | rs13037630 | | 51143319 | T | C | 0.183 | -0.018 | 0.002 | 1.00E-29 | 2.8E-04 | 128.16 | BMI |
| Right-arm FFM | rs2252720 | | 21223663 | T | C | 0.674 | -0.012 | 0.001 | 2.20E-20 | 1.9E-04 | 85.64 |  |
| Right-arm FFM | rs73619441 | | 61564901 | G | T | 0.144 | -0.012 | 0.002 | 3.00E-12 | 1.1E-04 | 48.70 | BMI |
| Right-arm FFM | rs2833354 | | 32636072 | A | G | 0.100 | -0.012 | 0.002 | 4.60E-09 | 7.6E-05 | 34.35 |  |
| Right-arm FFM | rs9976812 | | 39690245 | G | C | 0.564 | -0.011 | 0.001 | 1.30E-19 | 1.8E-04 | 82.12 |  |
| Right-arm FFM | rs9636630 | | 37474029 | G | A | 0.233 | -0.008 | 0.001 | 2.40E-08 | 6.8E-05 | 31.10 |  |
| Right-arm FFM | rs4253755 | | 46615376 | A | G | 0.128 | 0.011 | 0.002 | 1.10E-08 | 7.2E-05 | 32.68 | body fat percentage |
| Right-arm FFM | rs165656 | | 19948863 | C | G | 0.517 | 0.008 | 0.001 | 2.50E-10 | 8.8E-05 | 40.06 |  |
| Right-arm FFM | rs41311445 | | 42070374 | C | A | 0.096 | -0.024 | 0.002 | 2.80E-30 | 2.9E-04 | 130.75 |  |
| Right-arm FFM | rs113619763 | | 46399175 | T | A | 0.062 | 0.017 | 0.003 | 1.10E-10 | 9.2E-05 | 41.64 |  |
| Right-arm FFM | rs5771118 | | 50714289 | C | T | 0.742 | 0.008 | 0.001 | 1.60E-08 | 7.0E-05 | 31.89 |  |
| Right-arm FFM | rs35665085 | | 17625915 | A | G | 0.056 | -0.016 | 0.003 | 3.80E-09 | 7.6E-05 | 34.73 |  |
| Right-arm FFM | rs5752989 | | 30365780 | A | G | 0.571 | -0.010 | 0.001 | 2.20E-15 | 1.4E-04 | 62.87 |  |
| Right-arm FFM | rs5753630 | | 31861950 | G | A | 0.440 | 0.007 | 0.001 | 3.40E-08 | 6.7E-05 | 30.49 |  |
|  | |

Abbreviation: SNP, single nucleotide polymorphism; EA, Effect allele; NEA, Non-effect allele; EAF, effect allele frequency; SE, standard error; BMI, body mass index.

*^a^ R^2^* was calculated using the following formula: (2×EAF×(1-EAF)×beta^2^)/[(2×EAF×(1-EAF)×beta^2^)+(2×EAF×(1-EAF)×N×SE^2^)], where EAF is the effect allele

frequency, beta is the estimated effect on urate. Ν is the sample size of the GWAS for the SNP-urate association and SE is the standard error of the estimated effect.

*^b^ F* statistic was calculated using the following formula: *R^2^*(N-2)/(1-*R^2^*), where *R^2^* is the proportion of variance in urate explained by each instrument and N is the sample size of the GWAS for the SNP-urate association.

^c^ SNPs associated with confounding factors were removed after searching Phenoscanner database.

Supplementary Table 10 Characteristics of SNPs used as genetic instruments for left-arm FFM

| Exposure | SNP | | Position | EA | NEA | EAF | SNP-Exposure association | | | R^2 a^ | F-statistic ^b^ | Confounders ^c^ |
| --- | --- | --- | --- | --- | --- | --- | --- | --- | --- | --- | --- | --- |
|  |  | |  |  |  |  | Beta | SE | P value |  |  |  |
| Left-arm FFM | rs56333749 | | 9354520 | A | C | 0.208 | -0.009 | 0.002 | 2.60E-09 | 7.8E-05 | 35.44 |  |
| Left-arm FFM | rs3765351 | | 22445991 | T | C | 0.541 | -0.008 | 0.001 | 1.20E-09 | 8.2E-05 | 37.04 |  |
| Left-arm FFM | rs72660086 | | 39571992 | G | T | 0.211 | 0.011 | 0.002 | 1.60E-12 | 1.1E-04 | 49.88 | BMI |
| Left-arm FFM | rs1229947 | | 56597724 | C | T | 0.232 | 0.012 | 0.001 | 1.10E-15 | 1.4E-04 | 64.29 |  |
| Left-arm FFM | rs12140153 | | 62579891 | T | G | 0.094 | -0.017 | 0.002 | 4.90E-15 | 1.3E-04 | 61.31 | BMI |
| Left-arm FFM | rs56795609 | | 111208718 | T | A | 0.173 | -0.010 | 0.002 | 3.40E-09 | 7.7E-05 | 34.95 |  |
| Left-arm FFM | rs655598 | | 190287713 | A | G | 0.563 | -0.009 | 0.001 | 3.70E-12 | 1.1E-04 | 48.28 | BMI |
| Left-arm FFM | rs6693481 | | 203766395 | C | T | 0.695 | -0.008 | 0.001 | 5.50E-09 | 7.5E-05 | 34.01 |  |
| Left-arm FFM | rs2970592 | | 212220847 | A | G | 0.588 | 0.008 | 0.001 | 1.80E-10 | 8.9E-05 | 40.64 |  |
| Left-arm FFM | rs12072845 | | 214630757 | A | G | 0.395 | -0.013 | 0.001 | 1.20E-23 | 2.2E-04 | 100.45 |  |
| Left-arm FFM | rs41277186 | | 214776830 | G | C | 0.051 | -0.016 | 0.003 | 9.70E-09 | 7.2E-05 | 32.89 |  |
| Left-arm FFM | rs12073468 | | 225566546 | T | C | 0.228 | 0.011 | 0.001 | 8.30E-13 | 1.1E-04 | 51.22 |  |
| Left-arm FFM | rs417237 | | 228532195 | T | G | 0.617 | -0.009 | 0.001 | 4.60E-11 | 9.5E-05 | 43.36 |  |
| Left-arm FFM | rs2295363 | | 1850428 | G | A | 0.548 | 0.010 | 0.001 | 2.60E-16 | 1.5E-04 | 67.08 | BMI |
| Left-arm FFM | rs226250 | | 8022171 | T | C | 0.406 | -0.008 | 0.001 | 1.50E-10 | 9.0E-05 | 40.99 |  |
| Left-arm FFM | rs112750178 | | 26859695 | C | T | 0.238 | -0.009 | 0.001 | 3.80E-10 | 8.6E-05 | 39.21 |  |
| Left-arm FFM | rs10798945 | | 33858873 | T | C | 0.751 | 0.011 | 0.001 | 4.70E-15 | 1.3E-04 | 61.38 |  |
| Left-arm FFM | rs28605759 | | 38399816 | A | G | 0.454 | -0.007 | 0.001 | 5.10E-09 | 7.5E-05 | 34.14 |  |
| Left-arm FFM | rs12090545 | | 51193984 | G | A | 0.090 | 0.019 | 0.002 | 4.50E-18 | 1.7E-04 | 75.11 |  |
| Left-arm FFM | rs12726084 | | 72649154 | T | C | 0.204 | -0.013 | 0.002 | 1.80E-17 | 1.6E-04 | 72.31 | BMI |
| Left-arm FFM | rs12035149 | | 107885018 | G | C | 0.214 | 0.010 | 0.002 | 3.20E-10 | 8.7E-05 | 39.53 |  |
| Left-arm FFM | rs10749659 | | 151033979 | T | C | 0.772 | -0.010 | 0.001 | 8.80E-11 | 9.3E-05 | 42.08 | BMI |
| Left-arm FFM | rs1022523 | | 176788394 | A | G | 0.727 | 0.015 | 0.001 | 1.00E-27 | 2.6E-04 | 119.05 |  |
| Left-arm FFM | rs12731187 | | 202013757 | T | C | 0.358 | -0.008 | 0.001 | 1.60E-09 | 8.0E-05 | 36.46 | BMI |
| Left-arm FFM | rs903908 | | 2202967 | C | T | 0.509 | 0.009 | 0.001 | 1.60E-11 | 1.0E-04 | 45.42 |  |
| Left-arm FFM | rs1205593 | | 11252716 | C | T | 0.759 | -0.014 | 0.001 | 2.40E-21 | 2.0E-04 | 89.97 | BMI |
| Left-arm FFM | rs2885697 | | 41544279 | T | G | 0.665 | -0.018 | 0.001 | 5.00E-43 | 4.2E-04 | 189.08 |  |
| Left-arm FFM | rs4926542 | | 50263773 | T | C | 0.682 | -0.011 | 0.001 | 4.80E-15 | 1.3E-04 | 61.33 | BMI |
| Left-arm FFM | rs6680334 | | 66414440 | T | G | 0.367 | -0.007 | 0.001 | 4.00E-08 | 6.6E-05 | 30.16 | BMI |
| Left-arm FFM | rs34517439 | | 78450517 | A | C | 0.122 | 0.035 | 0.002 | 4.70E-74 | 7.3E-04 | 331.42 | BMI |
| Left-arm FFM | rs12731454 | | 97067652 | G | A | 0.309 | 0.011 | 0.001 | 6.00E-16 | 1.4E-04 | 65.43 | BMI |
| Left-arm FFM | rs4970846 | | 109946095 | C | A | 0.695 | -0.008 | 0.001 | 1.60E-08 | 7.0E-05 | 31.88 |  |
| Left-arm FFM | rs60804050 | | 118870373 | A | G | 0.256 | -0.010 | 0.001 | 2.00E-11 | 9.9E-05 | 45.00 |  |
| Left-arm FFM | rs7513326 | | 159895536 | A | G | 0.508 | -0.007 | 0.001 | 4.00E-08 | 6.6E-05 | 30.13 |  |
| Left-arm FFM | rs17277008 | | 172105162 | C | T | 0.313 | 0.014 | 0.001 | 1.40E-25 | 2.4E-04 | 109.27 |  |
| Left-arm FFM | rs7535501 | | 176462695 | T | C | 0.944 | -0.016 | 0.003 | 9.10E-09 | 7.3E-05 | 33.03 |  |
| Left-arm FFM | rs11240565 | | 205722958 | T | C | 0.398 | 0.011 | 0.001 | 1.20E-18 | 1.7E-04 | 77.74 |  |
| Left-arm FFM | rs35492502 | | 217806224 | A | G | 0.296 | 0.010 | 0.001 | 3.60E-13 | 1.2E-04 | 52.83 |  |
| Left-arm FFM | rs212526 | | 21584941 | C | T | 0.601 | 0.009 | 0.001 | 2.80E-13 | 1.2E-04 | 53.37 |  |
| Left-arm FFM | rs11578046 | | 23425139 | A | G | 0.327 | -0.012 | 0.001 | 6.30E-18 | 1.6E-04 | 74.42 |  |
| Left-arm FFM | rs148981711 | | 32748063 | G | A | 0.030 | 0.023 | 0.004 | 5.00E-10 | 8.5E-05 | 38.66 |  |
| Left-arm FFM | rs2678204 | | 201800511 | G | T | 0.340 | 0.013 | 0.001 | 1.60E-22 | 2.1E-04 | 95.39 | BMI |
| Left-arm FFM | rs3753614 | | 32081785 | A | G | 0.563 | 0.010 | 0.001 | 4.20E-16 | 1.5E-04 | 66.14 |  |
| Left-arm FFM | rs953567 | | 74977425 | G | A | 0.398 | 0.008 | 0.001 | 1.80E-10 | 8.9E-05 | 40.64 | BMI |
| Left-arm FFM | rs17363646 | | 86823503 | G | A | 0.136 | 0.012 | 0.002 | 2.20E-11 | 9.9E-05 | 44.79 |  |
| Left-arm FFM | rs76798800 | | 154994978 | T | G | 0.266 | 0.020 | 0.001 | 4.20E-45 | 4.4E-04 | 198.62 | BMI |
| Left-arm FFM | rs574367 | | 177873210 | T | G | 0.209 | 0.026 | 0.002 | 1.20E-61 | 6.0E-04 | 274.42 | BMI |
| Left-arm FFM | rs11580196 | | 227955276 | G | A | 0.170 | -0.010 | 0.002 | 5.00E-09 | 7.5E-05 | 34.18 |  |
| Left-arm FFM | rs11689727 | | 25458100 | A | C | 0.331 | -0.010 | 0.001 | 4.20E-15 | 1.4E-04 | 61.58 |  |
| Left-arm FFM | rs1260326 | | 27730940 | C | T | 0.604 | 0.018 | 0.001 | 4.10E-43 | 4.2E-04 | 189.47 |  |
| Left-arm FFM | rs1437377 | | 100744924 | G | T | 0.134 | 0.012 | 0.002 | 3.90E-11 | 9.6E-05 | 43.67 | BMI |
| Left-arm FFM | rs7601000 | | 242610773 | A | T | 0.767 | -0.010 | 0.001 | 7.50E-12 | 1.0E-04 | 46.90 |  |
| Left-arm FFM | rs77165542 | | 430975 | T | C | 0.035 | -0.053 | 0.003 | 1.50E-54 | 5.3E-04 | 241.93 | BMI |
| Left-arm FFM | rs115179432 | | 33348679 | G | A | 0.072 | -0.022 | 0.002 | 1.10E-19 | 1.8E-04 | 82.34 |  |
| Left-arm FFM | rs6711568 | | 59291172 | G | T | 0.702 | -0.010 | 0.001 | 3.60E-13 | 1.2E-04 | 52.86 | BMI |
| Left-arm FFM | rs11545482 | | 70315987 | T | C | 0.020 | -0.030 | 0.004 | 1.00E-11 | 1.0E-04 | 46.32 |  |
| Left-arm FFM | rs3771382 | | 71559445 | G | C | 0.571 | -0.010 | 0.001 | 3.70E-16 | 1.5E-04 | 66.40 |  |
| Left-arm FFM | rs12615038 | | 104371856 | G | T | 0.606 | -0.007 | 0.001 | 1.30E-08 | 7.1E-05 | 32.29 | BMI |
| Left-arm FFM | rs72845162 | | 128874657 | A | T | 0.199 | 0.009 | 0.002 | 1.70E-08 | 7.0E-05 | 31.86 |  |
| Left-arm FFM | rs1047891 | | 211540507 | A | C | 0.316 | 0.016 | 0.001 | 6.00E-34 | 3.2E-04 | 147.55 | BMI |
| Left-arm FFM | rs13430869 | | 218146818 | T | G | 0.742 | 0.012 | 0.001 | 4.70E-18 | 1.6E-04 | 75.01 |  |
| Left-arm FFM | rs2197563 | | 233687080 | A | G | 0.595 | 0.009 | 0.001 | 3.80E-13 | 1.2E-04 | 52.74 |  |
| Left-arm FFM | rs6721191 | | 10190115 | G | A | 0.578 | -0.007 | 0.001 | 9.40E-09 | 7.3E-05 | 32.96 |  |
| Left-arm FFM | rs112544217 | | 20222686 | T | C | 0.022 | -0.024 | 0.004 | 3.00E-08 | 6.8E-05 | 30.69 |  |
| Left-arm FFM | rs343954 | | 44999709 | C | T | 0.172 | 0.012 | 0.002 | 1.20E-13 | 1.2E-04 | 55.00 |  |
| Left-arm FFM | rs76733024 | | 46995516 | G | A | 0.065 | -0.014 | 0.003 | 1.50E-08 | 7.1E-05 | 32.07 |  |
| Left-arm FFM | rs17049820 | | 59139507 | C | T | 0.113 | -0.011 | 0.002 | 1.10E-08 | 7.2E-05 | 32.63 | BMI |
| Left-arm FFM | rs1374370 | | 85818273 | A | G | 0.305 | 0.009 | 0.001 | 1.10E-11 | 1.0E-04 | 46.18 |  |
| Left-arm FFM | rs11680549 | | 88926348 | C | G | 0.280 | 0.009 | 0.001 | 2.20E-10 | 8.9E-05 | 40.31 |  |
| Left-arm FFM | rs1837367 | | 111874551 | A | G | 0.483 | 0.007 | 0.001 | 1.60E-08 | 7.0E-05 | 31.93 |  |
| Left-arm FFM | rs10188231 | | 142297493 | G | C | 0.186 | -0.010 | 0.002 | 5.40E-10 | 8.5E-05 | 38.53 | BMI |
| Left-arm FFM | rs2140046 | | 169706079 | C | T | 0.364 | -0.012 | 0.001 | 7.80E-20 | 1.8E-04 | 83.10 |  |
| Left-arm FFM | rs72885917 | | 172416376 | C | A | 0.247 | -0.018 | 0.001 | 6.00E-36 | 3.4E-04 | 156.68 |  |
| Left-arm FFM | rs116337081 | | 183954625 | T | C | 0.070 | 0.014 | 0.002 | 4.30E-08 | 6.6E-05 | 29.99 |  |
| Left-arm FFM | rs12694042 | | 207029825 | T | C | 0.502 | -0.009 | 0.001 | 1.80E-12 | 1.1E-04 | 49.65 |  |
| Left-arm FFM | rs1542224 | | 223963874 | C | T | 0.719 | 0.011 | 0.001 | 1.80E-14 | 1.3E-04 | 58.69 |  |
| Left-arm FFM | rs3116201 | | 233074205 | A | G | 0.098 | -0.017 | 0.002 | 3.90E-16 | 1.5E-04 | 66.27 |  |
| Left-arm FFM | rs6743060 | | 629510 | A | C | 0.828 | 0.033 | 0.002 | 2.90E-88 | 8.7E-04 | 396.66 | BMI |
| Left-arm FFM | rs2372604 | | 36820390 | T | G | 0.380 | 0.008 | 0.001 | 3.00E-10 | 8.7E-05 | 39.66 | body fat percentage |
| Left-arm FFM | rs222478 | | 42656919 | A | C | 0.130 | -0.015 | 0.002 | 1.70E-16 | 1.5E-04 | 67.90 |  |
| Left-arm FFM | rs17400325 | | 178565913 | C | T | 0.042 | 0.020 | 0.003 | 3.20E-10 | 8.7E-05 | 39.54 |  |
| Left-arm FFM | rs10803955 | | 183228114 | G | A | 0.508 | -0.011 | 0.001 | 5.70E-18 | 1.6E-04 | 74.63 |  |
| Left-arm FFM | rs1064213 | | 198950240 | A | G | 0.478 | 0.012 | 0.001 | 9.40E-23 | 2.1E-04 | 96.40 | BMI |
| Left-arm FFM | rs34756249 | | 218279486 | C | T | 0.684 | 0.016 | 0.001 | 8.80E-33 | 3.1E-04 | 142.20 |  |
| Left-arm FFM | rs11684531 | | 219835489 | G | A | 0.133 | -0.010 | 0.002 | 2.90E-08 | 6.8E-05 | 30.80 |  |
| Left-arm FFM | rs7592897 | | 227263582 | T | C | 0.304 | 0.012 | 0.001 | 8.50E-19 | 1.7E-04 | 78.38 |  |
| Left-arm FFM | rs12713004 | | 23896049 | G | A | 0.725 | 0.016 | 0.001 | 1.70E-28 | 2.7E-04 | 122.55 |  |
| Left-arm FFM | rs11678716 | | 33487251 | C | G | 0.068 | -0.018 | 0.002 | 8.40E-13 | 1.1E-04 | 51.18 |  |
| Left-arm FFM | rs59985551 | | 56106928 | T | C | 0.226 | -0.014 | 0.001 | 1.90E-21 | 2.0E-04 | 90.43 |  |
| Left-arm FFM | rs7581335 | | 143958864 | T | A | 0.141 | 0.012 | 0.002 | 1.40E-11 | 1.0E-04 | 45.66 | BMI |
| Left-arm FFM | rs17443541 | | 200402624 | C | T | 0.189 | -0.011 | 0.002 | 1.30E-11 | 1.0E-04 | 45.79 |  |
| Left-arm FFM | rs2270894 | | 9975386 | G | C | 0.203 | -0.014 | 0.002 | 2.40E-18 | 1.7E-04 | 76.35 |  |
| Left-arm FFM | rs2600223 | | 12934932 | G | A | 0.683 | -0.008 | 0.001 | 2.60E-08 | 6.8E-05 | 30.97 | BMI |
| Left-arm FFM | rs6779752 | | 85663849 | A | G | 0.638 | -0.013 | 0.001 | 3.80E-23 | 2.2E-04 | 98.19 | BMI |
| Left-arm FFM | rs4858940 | | 88254820 | C | T | 0.886 | 0.016 | 0.002 | 1.50E-16 | 1.5E-04 | 68.18 | BMI |
| Left-arm FFM | rs4257594 | | 121522944 | T | C | 0.636 | -0.007 | 0.001 | 1.10E-08 | 7.2E-05 | 32.74 |  |
| Left-arm FFM | rs4635681 | | 152310614 | G | A | 0.156 | 0.011 | 0.002 | 1.50E-10 | 9.0E-05 | 40.99 |  |
| Left-arm FFM | rs10936684 | | 171130793 | A | G | 0.660 | -0.008 | 0.001 | 5.40E-10 | 8.5E-05 | 38.53 | BMI |
| Left-arm FFM | rs62246311 | | 9498143 | A | G | 0.102 | 0.013 | 0.002 | 1.00E-09 | 8.2E-05 | 37.32 | BMI |
| Left-arm FFM | rs10084690 | | 33591384 | C | A | 0.035 | 0.021 | 0.003 | 1.30E-09 | 8.1E-05 | 36.78 |  |
| Left-arm FFM | rs6792892 | | 49995518 | C | T | 0.531 | 0.015 | 0.001 | 4.90E-32 | 3.1E-04 | 138.78 | BMI |
| Left-arm FFM | rs11925245 | | 114183579 | G | A | 0.183 | -0.010 | 0.002 | 6.30E-10 | 8.4E-05 | 38.24 |  |
| Left-arm FFM | rs7632381 | | 141106063 | C | T | 0.444 | 0.028 | 0.001 | 1.30E-106 | 1.1E-03 | 480.98 |  |
| Left-arm FFM | rs1092403 | | 153926744 | T | C | 0.538 | 0.010 | 0.001 | 3.40E-14 | 1.3E-04 | 57.47 | BMI |
| Left-arm FFM | rs509035 | | 172163449 | A | G | 0.316 | 0.016 | 0.001 | 2.80E-31 | 3.0E-04 | 135.36 |  |
| Left-arm FFM | rs73175572 | | 185490184 | G | A | 0.112 | 0.025 | 0.002 | 2.00E-35 | 3.4E-04 | 154.28 |  |
| Left-arm FFM | rs73052033 | | 185828465 | C | T | 0.185 | -0.016 | 0.002 | 1.20E-22 | 2.1E-04 | 95.98 | BMI |
| Left-arm FFM | rs7619139 | | 25110415 | A | T | 0.589 | 0.010 | 0.001 | 1.10E-15 | 1.4E-04 | 64.22 | BMI |
| Left-arm FFM | rs3749387 | | 38496193 | C | G | 0.530 | 0.011 | 0.001 | 1.40E-19 | 1.8E-04 | 81.97 |  |
| Left-arm FFM | rs79979130 | | 52551010 | T | C | 0.087 | 0.014 | 0.002 | 1.00E-10 | 9.2E-05 | 41.73 |  |
| Left-arm FFM | rs75451531 | | 61187422 | T | C | 0.147 | -0.013 | 0.002 | 4.30E-14 | 1.3E-04 | 57.04 | BMI |
| Left-arm FFM | rs6772164 | | 196078149 | A | C | 0.358 | 0.009 | 0.001 | 3.80E-11 | 9.6E-05 | 43.72 | BMI |
| Left-arm FFM | rs6762851 | | 56686329 | C | T | 0.357 | -0.011 | 0.001 | 1.50E-17 | 1.6E-04 | 72.65 |  |
| Left-arm FFM | rs6769617 | | 62687746 | T | A | 0.664 | -0.009 | 0.001 | 9.70E-11 | 9.2E-05 | 41.88 | BMI |
| Left-arm FFM | rs4677150 | | 72393982 | G | C | 0.504 | -0.008 | 0.001 | 9.90E-10 | 8.2E-05 | 37.35 |  |
| Left-arm FFM | rs9835772 | | 85766025 | T | A | 0.244 | 0.012 | 0.001 | 3.90E-16 | 1.5E-04 | 66.27 | BMI |
| Left-arm FFM | rs7633464 | | 98715823 | A | G | 0.478 | 0.009 | 0.001 | 4.20E-13 | 1.2E-04 | 52.54 |  |
| Left-arm FFM | rs6762578 | | 128992047 | A | G | 0.778 | 0.014 | 0.002 | 7.90E-20 | 1.8E-04 | 83.06 |  |
| Left-arm FFM | rs2651316 | | 147059334 | A | G | 0.753 | -0.009 | 0.001 | 4.50E-09 | 7.6E-05 | 34.42 |  |
| Left-arm FFM | rs9790159 | | 171908032 | G | A | 0.530 | -0.010 | 0.001 | 5.80E-15 | 1.3E-04 | 60.96 |  |
| Left-arm FFM | rs843374 | | 183997261 | T | A | 0.587 | -0.010 | 0.001 | 8.50E-14 | 1.2E-04 | 55.68 |  |
| Left-arm FFM | rs4076108 | | 13736088 | T | A | 0.245 | 0.008 | 0.001 | 1.40E-08 | 7.1E-05 | 32.22 |  |
| Left-arm FFM | rs9680881 | | 93526697 | A | G | 0.297 | -0.008 | 0.001 | 4.70E-08 | 6.6E-05 | 29.82 | BMI |
| Left-arm FFM | rs11709402 | | 131551027 | G | A | 0.279 | 0.010 | 0.001 | 1.70E-12 | 1.1E-04 | 49.78 | BMI |
| Left-arm FFM | rs113061374 | | 1052662 | T | C | 0.048 | -0.023 | 0.003 | 7.60E-15 | 1.3E-04 | 60.43 |  |
| Left-arm FFM | rs981002 | | 12881731 | A | T | 0.267 | -0.011 | 0.001 | 1.20E-13 | 1.2E-04 | 54.96 |  |
| Left-arm FFM | rs9985795 | | 135213286 | C | T | 0.483 | -0.007 | 0.001 | 9.10E-09 | 7.3E-05 | 33.02 |  |
| Left-arm FFM | rs10516169 | | 4935309 | G | T | 0.378 | 0.009 | 0.001 | 1.40E-11 | 1.0E-04 | 45.62 |  |
| Left-arm FFM | rs7671110 | | 17874089 | T | C | 0.158 | -0.028 | 0.002 | 6.50E-59 | 5.8E-04 | 261.94 |  |
| Left-arm FFM | rs10938397 | | 45182527 | G | A | 0.434 | 0.013 | 0.001 | 4.10E-24 | 2.3E-04 | 102.59 | BMI |
| Left-arm FFM | rs10222924 | | 49016761 | G | A | 0.699 | -0.010 | 0.001 | 2.10E-12 | 1.1E-04 | 49.40 |  |
| Left-arm FFM | rs1841738 | | 88557753 | G | A | 0.518 | -0.011 | 0.001 | 7.10E-18 | 1.6E-04 | 74.19 |  |
| Left-arm FFM | rs2101975 | | 106216667 | G | A | 0.432 | -0.015 | 0.001 | 4.90E-30 | 2.9E-04 | 129.63 |  |
| Left-arm FFM | rs6824633 | | 111019965 | T | C | 0.353 | 0.008 | 0.001 | 5.30E-09 | 7.5E-05 | 34.08 |  |
| Left-arm FFM | rs11098675 | | 123832776 | G | A | 0.806 | -0.014 | 0.002 | 9.50E-20 | 1.8E-04 | 82.71 |  |
| Left-arm FFM | rs111598585 | | 171635471 | T | C | 0.209 | -0.009 | 0.002 | 3.30E-08 | 6.7E-05 | 30.53 |  |
| Left-arm FFM | rs10939792 | | 18483405 | G | C | 0.320 | 0.007 | 0.001 | 3.20E-08 | 6.7E-05 | 30.60 | BMI |
| Left-arm FFM | rs4596205 | | 31012310 | T | C | 0.349 | 0.010 | 0.001 | 2.20E-13 | 1.2E-04 | 53.84 | BMI |
| Left-arm FFM | rs6554194 | | 55499810 | G | T | 0.556 | -0.007 | 0.001 | 2.00E-08 | 6.9E-05 | 31.53 | BMI |
| Left-arm FFM | rs10020631 | | 69353863 | A | G | 0.248 | -0.008 | 0.001 | 6.50E-09 | 7.4E-05 | 33.69 |  |
| Left-arm FFM | rs17556750 | | 82155568 | A | C | 0.288 | 0.012 | 0.001 | 2.70E-18 | 1.7E-04 | 76.10 |  |
| Left-arm FFM | rs4240326 | | 145839264 | G | A | 0.550 | -0.020 | 0.001 | 3.70E-56 | 5.5E-04 | 249.27 |  |
| Left-arm FFM | rs7683836 | | 180167906 | A | G | 0.557 | -0.007 | 0.001 | 2.90E-08 | 6.8E-05 | 30.77 | BMI |
| Left-arm FFM | rs4865465 | | 1745486 | A | G | 0.535 | 0.008 | 0.001 | 1.60E-10 | 9.0E-05 | 40.93 |  |
| Left-arm FFM | rs73213484 | | 28489339 | T | A | 0.141 | -0.013 | 0.002 | 4.60E-13 | 1.2E-04 | 52.39 | BMI |
| Left-arm FFM | rs1296328 | | 137083193 | C | A | 0.559 | -0.009 | 0.001 | 2.80E-12 | 1.1E-04 | 48.82 | BMI |
| Left-arm FFM | rs10434434 | | 54271611 | C | A | 0.148 | -0.014 | 0.002 | 3.90E-16 | 1.5E-04 | 66.28 |  |
| Left-arm FFM | rs140493137 | | 87666916 | A | G | 0.060 | 0.018 | 0.003 | 3.90E-12 | 1.1E-04 | 48.15 |  |
| Left-arm FFM | rs11097755 | | 102709308 | C | T | 0.443 | 0.008 | 0.001 | 7.40E-11 | 9.3E-05 | 42.42 | body fat percentage |
| Left-arm FFM | rs6821305 | | 122713863 | C | A | 0.399 | 0.012 | 0.001 | 2.80E-21 | 2.0E-04 | 89.71 |  |
| Left-arm FFM | rs36089326 | | 160028812 | T | A | 0.327 | 0.009 | 0.001 | 6.90E-12 | 1.0E-04 | 47.05 |  |
| Left-arm FFM | rs62372052 | | 42724294 | G | A | 0.110 | 0.025 | 0.002 | 2.90E-36 | 3.5E-04 | 158.10 |  |
| Left-arm FFM | rs1582931 | | 122657199 | A | G | 0.473 | -0.017 | 0.001 | 1.30E-40 | 3.9E-04 | 178.02 | BMI |
| Left-arm FFM | rs9327336 | | 123990270 | C | T | 0.343 | 0.009 | 0.001 | 6.80E-11 | 9.4E-05 | 42.57 |  |
| Left-arm FFM | rs247008 | | 131447104 | G | A | 0.670 | 0.011 | 0.001 | 3.50E-17 | 1.6E-04 | 71.05 |  |
| Left-arm FFM | rs33967909 | | 137603293 | A | G | 0.216 | 0.012 | 0.002 | 5.60E-15 | 1.3E-04 | 61.05 |  |
| Left-arm FFM | rs6867471 | | 3574564 | T | C | 0.362 | -0.008 | 0.001 | 3.40E-09 | 7.7E-05 | 34.96 | BMI |
| Left-arm FFM | rs6450346 | | 55014771 | C | T | 0.701 | -0.011 | 0.001 | 6.00E-15 | 1.3E-04 | 60.89 |  |
| Left-arm FFM | rs12188627 | | 60720682 | G | A | 0.488 | -0.009 | 0.001 | 6.70E-14 | 1.2E-04 | 56.16 |  |
| Left-arm FFM | rs9291926 | | 67599656 | G | T | 0.532 | -0.012 | 0.001 | 1.40E-21 | 2.0E-04 | 91.07 |  |
| Left-arm FFM | rs4588572 | | 77631634 | G | A | 0.228 | 0.009 | 0.002 | 3.30E-10 | 8.7E-05 | 39.48 |  |
| Left-arm FFM | rs61749613 | | 82815170 | G | A | 0.041 | 0.025 | 0.003 | 7.20E-15 | 1.3E-04 | 60.53 | body fat percentage |
| Left-arm FFM | rs35267052 | | 87949118 | G | T | 0.103 | 0.014 | 0.002 | 9.10E-12 | 1.0E-04 | 46.52 | BMI |
| Left-arm FFM | rs299370 | | 134339065 | C | T | 0.307 | -0.008 | 0.001 | 6.80E-09 | 7.4E-05 | 33.60 |  |
| Left-arm FFM | rs13173394 | | 161385636 | C | T | 0.631 | 0.008 | 0.001 | 1.00E-09 | 8.2E-05 | 37.25 |  |
| Left-arm FFM | rs111365325 | | 170865229 | T | C | 0.231 | -0.013 | 0.001 | 4.40E-17 | 1.6E-04 | 70.61 |  |
| Left-arm FFM | rs55758152 | | 171317318 | A | G | 0.326 | 0.009 | 0.001 | 8.30E-11 | 9.3E-05 | 42.18 |  |
| Left-arm FFM | rs252938 | | 5496480 | T | C | 0.649 | -0.008 | 0.001 | 1.80E-10 | 9.0E-05 | 40.68 |  |
| Left-arm FFM | rs7731023 | | 36181627 | G | A | 0.574 | 0.007 | 0.001 | 3.00E-08 | 6.8E-05 | 30.68 |  |
| Left-arm FFM | rs365352 | | 77401152 | A | G | 0.244 | -0.013 | 0.001 | 6.90E-19 | 1.7E-04 | 78.78 | BMI |
| Left-arm FFM | rs10515237 | | 95751549 | G | A | 0.282 | 0.013 | 0.001 | 6.80E-21 | 1.9E-04 | 87.92 | BMI |
| Left-arm FFM | rs2952615 | | 112138888 | C | G | 0.618 | -0.012 | 0.001 | 9.40E-20 | 1.8E-04 | 82.74 |  |
| Left-arm FFM | rs17115481 | | 153358226 | A | G | 0.270 | -0.009 | 0.001 | 1.20E-10 | 9.1E-05 | 41.42 | BMI |
| Left-arm FFM | rs4282339 | | 168256240 | A | G | 0.208 | -0.018 | 0.002 | 2.20E-31 | 3.0E-04 | 135.81 |  |
| Left-arm FFM | rs1990657 | | 171220503 | C | T | 0.428 | -0.008 | 0.001 | 4.50E-10 | 8.6E-05 | 38.90 |  |
| Left-arm FFM | rs12657771 | | 36787962 | A | G | 0.439 | -0.011 | 0.001 | 7.70E-18 | 1.6E-04 | 74.02 |  |
| Left-arm FFM | rs2307111 | | 75003678 | C | T | 0.395 | -0.017 | 0.001 | 2.40E-40 | 3.9E-04 | 176.79 | BMI |
| Left-arm FFM | rs7442885 | | 87682877 | G | C | 0.214 | -0.017 | 0.002 | 5.70E-27 | 2.5E-04 | 115.63 | BMI |
| Left-arm FFM | rs6874142 | | 172753555 | G | T | 0.114 | 0.016 | 0.002 | 1.90E-14 | 1.3E-04 | 58.63 |  |
| Left-arm FFM | rs244711 | | 176509193 | T | C | 0.686 | 0.017 | 0.001 | 5.60E-31 | 2.9E-04 | 133.96 |  |
| Left-arm FFM | rs11739036 | | 32723478 | A | G | 0.342 | -0.011 | 0.001 | 1.90E-15 | 1.4E-04 | 63.21 |  |
| Left-arm FFM | rs6898801 | | 111250615 | G | A | 0.658 | -0.009 | 0.001 | 3.20E-12 | 1.1E-04 | 48.55 |  |
| Left-arm FFM | rs3822742 | | 139059017 | A | C | 0.371 | 0.013 | 0.001 | 1.30E-22 | 2.1E-04 | 95.82 | BMI |
| Left-arm FFM | rs12213070 | | 12131542 | A | G | 0.346 | -0.008 | 0.001 | 7.70E-10 | 8.3E-05 | 37.84 | BMI |
| Left-arm FFM | rs12209223 | | 76164589 | A | C | 0.101 | 0.015 | 0.002 | 6.20E-13 | 1.1E-04 | 51.80 |  |
| Left-arm FFM | rs4946936 | | 109003321 | C | T | 0.713 | 0.022 | 0.001 | 2.30E-56 | 5.5E-04 | 250.22 | BMI |
| Left-arm FFM | rs7755185 | | 152339615 | G | A | 0.311 | 0.008 | 0.001 | 3.00E-08 | 6.8E-05 | 30.69 |  |
| Left-arm FFM | rs9379084 | | 7231843 | A | G | 0.116 | -0.015 | 0.002 | 1.60E-13 | 1.2E-04 | 54.46 |  |
| Left-arm FFM | rs62396185 | | 26180634 | C | G | 0.260 | -0.021 | 0.001 | 4.10E-48 | 4.7E-04 | 212.39 | BMI |
| Left-arm FFM | rs1150780 | | 34215228 | A | G | 0.257 | -0.008 | 0.001 | 1.80E-08 | 7.0E-05 | 31.68 |  |
| Left-arm FFM | rs2395792 | | 41852187 | G | T | 0.956 | -0.027 | 0.003 | 4.00E-18 | 1.7E-04 | 75.34 |  |
| Left-arm FFM | rs72892910 | | 50816887 | T | G | 0.172 | 0.020 | 0.002 | 3.10E-34 | 3.3E-04 | 148.86 | BMI |
| Left-arm FFM | rs578366 | | 81591034 | G | A | 0.420 | -0.010 | 0.001 | 2.10E-16 | 1.5E-04 | 67.55 |  |
| Left-arm FFM | rs6902789 | | 105358192 | A | G | 0.368 | 0.008 | 0.001 | 6.20E-10 | 8.4E-05 | 38.24 |  |
| Left-arm FFM | rs10457469 | | 126083658 | A | G | 0.523 | 0.014 | 0.001 | 1.20E-29 | 2.8E-04 | 127.85 | BMI |
| Left-arm FFM | rs9388498 | | 126873423 | T | G | 0.186 | 0.016 | 0.002 | 1.80E-22 | 2.1E-04 | 95.07 |  |
| Left-arm FFM | rs6570509 | | 142716286 | T | G | 0.287 | -0.013 | 0.001 | 2.50E-21 | 2.0E-04 | 89.94 | BMI |
| Left-arm FFM | rs394487 | | 160778639 | T | C | 0.278 | 0.010 | 0.001 | 1.40E-11 | 1.0E-04 | 45.69 | BMI |
| Left-arm FFM | rs5019542 | | 51738352 | T | C | 0.636 | -0.007 | 0.001 | 1.20E-08 | 7.1E-05 | 32.43 |  |
| Left-arm FFM | rs10943915 | | 83617970 | T | A | 0.297 | 0.008 | 0.001 | 8.90E-09 | 7.3E-05 | 33.07 |  |
| Left-arm FFM | rs11153171 | | 109653825 | T | C | 0.356 | -0.012 | 0.001 | 1.90E-19 | 1.8E-04 | 81.38 |  |
| Left-arm FFM | rs7740107 | | 130374461 | A | T | 0.736 | -0.028 | 0.001 | 9.00E-86 | 8.5E-04 | 385.25 |  |
| Left-arm FFM | rs603321 | | 140256669 | G | A | 0.244 | -0.012 | 0.001 | 5.00E-17 | 1.5E-04 | 70.34 |  |
| Left-arm FFM | rs3853252 | | 152170247 | A | G | 0.455 | 0.015 | 0.001 | 7.40E-31 | 2.9E-04 | 133.41 |  |
| Left-arm FFM | rs9457777 | | 160352921 | C | T | 0.228 | 0.009 | 0.001 | 1.10E-09 | 8.2E-05 | 37.19 |  |
| Left-arm FFM | rs766164 | | 164107208 | T | C | 0.662 | 0.008 | 0.001 | 1.40E-08 | 7.1E-05 | 32.16 |  |
| Left-arm FFM | rs11243202 | | 7719065 | C | T | 0.486 | 0.017 | 0.001 | 7.10E-43 | 4.1E-04 | 188.40 |  |
| Left-arm FFM | rs12216497 | | 19028623 | T | C | 0.561 | -0.011 | 0.001 | 2.50E-19 | 1.8E-04 | 80.76 |  |
| Left-arm FFM | rs41271299 | | 19839415 | T | C | 0.051 | 0.040 | 0.003 | 1.40E-45 | 4.4E-04 | 200.77 |  |
| Left-arm FFM | rs3131115 | | 30468791 | T | C | 0.366 | 0.009 | 0.001 | 2.10E-12 | 1.1E-04 | 49.40 |  |
| Left-arm FFM | rs41268896 | | 32070069 | A | G | 0.298 | -0.016 | 0.001 | 1.40E-32 | 3.1E-04 | 141.30 |  |
| Left-arm FFM | rs1539111 | | 120080096 | G | A | 0.242 | 0.008 | 0.001 | 2.20E-08 | 6.9E-05 | 31.27 |  |
| Left-arm FFM | rs687694 | | 153472227 | G | T | 0.495 | -0.008 | 0.001 | 5.30E-10 | 8.5E-05 | 38.55 |  |
| Left-arm FFM | rs3099282 | | 166584842 | G | A | 0.208 | -0.009 | 0.002 | 1.50E-08 | 7.1E-05 | 32.11 |  |
| Left-arm FFM | rs2744956 | | 34618937 | C | T | 0.139 | 0.035 | 0.002 | 2.10E-84 | 8.3E-04 | 378.97 | BMI |
| Left-arm FFM | rs34045288 | | 40369081 | T | C | 0.334 | 0.012 | 0.001 | 1.30E-18 | 1.7E-04 | 77.57 | BMI |
| Left-arm FFM | rs1832897 | | 44741714 | A | G | 0.597 | -0.007 | 0.001 | 2.00E-08 | 6.9E-05 | 31.49 |  |
| Left-arm FFM | rs9350850 | | 81050236 | C | T | 0.080 | 0.022 | 0.002 | 5.20E-22 | 2.0E-04 | 93.03 |  |
| Left-arm FFM | rs6940643 | | 82575450 | G | A | 0.404 | 0.008 | 0.001 | 4.30E-10 | 8.6E-05 | 38.99 |  |
| Left-arm FFM | rs1006399 | | 101039470 | A | G | 0.459 | -0.009 | 0.001 | 2.20E-12 | 1.1E-04 | 49.31 |  |
| Left-arm FFM | rs35575481 | | 163007746 | A | T | 0.138 | -0.012 | 0.002 | 5.50E-11 | 9.5E-05 | 42.99 | BMI |
| Left-arm FFM | rs2457982 | | 166313684 | A | G | 0.279 | 0.009 | 0.001 | 1.60E-09 | 8.0E-05 | 36.36 |  |
| Left-arm FFM | rs10242866 | | 17920613 | T | C | 0.399 | 0.008 | 0.001 | 5.70E-10 | 8.5E-05 | 38.43 |  |
| Left-arm FFM | rs723149 | | 46577056 | G | A | 0.563 | -0.010 | 0.001 | 4.90E-15 | 1.3E-04 | 61.30 |  |
| Left-arm FFM | rs236628 | | 75023850 | T | G | 0.236 | 0.010 | 0.002 | 2.50E-11 | 9.8E-05 | 44.57 | BMI |
| Left-arm FFM | rs1701827 | | 113040467 | G | A | 0.270 | -0.008 | 0.001 | 9.10E-09 | 7.3E-05 | 33.03 | BMI |
| Left-arm FFM | rs836532 | | 6472354 | A | G | 0.202 | 0.010 | 0.002 | 5.60E-11 | 9.4E-05 | 42.95 | BMI |
| Left-arm FFM | rs10950207 | | 70105168 | T | C | 0.384 | 0.008 | 0.001 | 1.10E-10 | 9.2E-05 | 41.71 | BMI |
| Left-arm FFM | rs4729085 | | 76592670 | G | C | 0.868 | -0.015 | 0.002 | 2.90E-16 | 1.5E-04 | 66.90 | BMI |
| Left-arm FFM | rs60667771 | | 92471179 | C | T | 0.160 | 0.010 | 0.002 | 2.70E-09 | 7.8E-05 | 35.41 |  |
| Left-arm FFM | rs28547519 | | 121964618 | C | T | 0.366 | 0.011 | 0.001 | 2.80E-16 | 1.5E-04 | 66.92 | BMI |
| Left-arm FFM | rs62621812 | | 127015083 | A | G | 0.020 | 0.039 | 0.005 | 1.40E-17 | 1.6E-04 | 72.89 | BMI |
| Left-arm FFM | rs6946415 | | 150684548 | G | A | 0.627 | 0.018 | 0.001 | 8.30E-44 | 4.2E-04 | 192.68 | BMI |
| Left-arm FFM | rs2533879 | | 2859847 | A | G | 0.300 | -0.017 | 0.001 | 8.10E-35 | 3.3E-04 | 151.51 | body fat percentage |
| Left-arm FFM | rs2140910 | | 39079473 | C | T | 0.884 | -0.012 | 0.002 | 3.90E-10 | 8.6E-05 | 39.14 |  |
| Left-arm FFM | rs74876583 | | 50602539 | G | T | 0.117 | 0.017 | 0.002 | 1.80E-17 | 1.6E-04 | 72.34 |  |
| Left-arm FFM | rs10260993 | | 55884295 | G | T | 0.196 | -0.009 | 0.002 | 8.40E-09 | 7.3E-05 | 33.18 |  |
| Left-arm FFM | rs10269774 | | 92253972 | A | G | 0.326 | 0.024 | 0.001 | 4.30E-70 | 6.9E-04 | 313.22 |  |
| Left-arm FFM | rs10243988 | | 99528761 | A | G | 0.195 | -0.010 | 0.002 | 6.60E-10 | 8.4E-05 | 38.12 |  |
| Left-arm FFM | rs822549 | | 148649180 | C | T | 0.267 | 0.011 | 0.001 | 3.40E-14 | 1.3E-04 | 57.47 |  |
| Left-arm FFM | rs10226050 | | 8061334 | G | A | 0.457 | 0.007 | 0.001 | 3.40E-09 | 7.7E-05 | 34.96 |  |
| Left-arm FFM | rs34776209 | | 23513093 | T | C | 0.247 | -0.014 | 0.001 | 8.10E-23 | 2.1E-04 | 96.68 |  |
| Left-arm FFM | rs12700901 | | 28783171 | A | C | 0.406 | -0.008 | 0.001 | 1.30E-09 | 8.1E-05 | 36.87 |  |
| Left-arm FFM | rs13240065 | | 73015369 | A | G | 0.128 | 0.018 | 0.002 | 8.70E-22 | 2.0E-04 | 91.98 |  |
| Left-arm FFM | rs115946508 | | 150497496 | A | C | 0.111 | -0.012 | 0.002 | 4.10E-09 | 7.6E-05 | 34.60 |  |
| Left-arm FFM | rs508347 | | 28212824 | C | T | 0.703 | -0.010 | 0.001 | 3.40E-14 | 1.3E-04 | 57.49 |  |
| Left-arm FFM | rs3778934 | | 39445385 | C | A | 0.341 | -0.008 | 0.001 | 2.90E-09 | 7.8E-05 | 35.23 | BMI |
| Left-arm FFM | rs7776917 | | 93089120 | A | G | 0.477 | 0.011 | 0.001 | 6.00E-17 | 1.5E-04 | 69.96 | BMI |
| Left-arm FFM | rs12375196 | | 103416541 | A | C | 0.424 | 0.010 | 0.001 | 6.30E-15 | 1.3E-04 | 60.82 | BMI |
| Left-arm FFM | rs55674305 | | 89435868 | A | G | 0.306 | -0.010 | 0.001 | 3.00E-12 | 1.1E-04 | 48.66 | BMI |
| Left-arm FFM | rs13271368 | | 126506140 | T | C | 0.237 | -0.012 | 0.002 | 3.90E-15 | 1.4E-04 | 61.76 |  |
| Left-arm FFM | rs12156265 | | 144848756 | A | G | 0.590 | 0.008 | 0.001 | 1.60E-09 | 8.0E-05 | 36.44 |  |
| Left-arm FFM | rs17089329 | | 23390046 | G | C | 0.203 | 0.012 | 0.002 | 1.10E-13 | 1.2E-04 | 55.12 |  |
| Left-arm FFM | rs13264909 | | 64702385 | T | A | 0.429 | -0.008 | 0.001 | 2.60E-10 | 8.8E-05 | 39.96 | BMI |
| Left-arm FFM | rs17828687 | | 73454767 | A | C | 0.573 | -0.010 | 0.001 | 3.00E-15 | 1.4E-04 | 62.25 | BMI |
| Left-arm FFM | rs4909912 | | 135572297 | T | C | 0.426 | -0.017 | 0.001 | 1.80E-41 | 4.0E-04 | 182.00 |  |
| Left-arm FFM | rs6984820 | | 144993324 | T | C | 0.422 | -0.011 | 0.001 | 1.40E-16 | 1.5E-04 | 68.33 |  |
| Left-arm FFM | rs11777007 | | 10801857 | T | C | 0.547 | -0.010 | 0.001 | 3.00E-15 | 1.4E-04 | 62.28 | BMI |
| Left-arm FFM | rs11997525 | | 49401982 | A | T | 0.167 | 0.014 | 0.002 | 1.70E-17 | 1.6E-04 | 72.51 |  |
| Left-arm FFM | rs72656010 | | 57122215 | C | T | 0.132 | -0.032 | 0.002 | 7.40E-65 | 6.4E-04 | 289.22 |  |
| Left-arm FFM | rs7842996 | | 78107140 | A | T | 0.284 | 0.017 | 0.001 | 2.40E-35 | 3.4E-04 | 153.91 |  |
| Left-arm FFM | rs62515438 | | 57161608 | G | T | 0.228 | 0.017 | 0.002 | 2.50E-30 | 2.9E-04 | 130.97 |  |
| Left-arm FFM | rs11779459 | | 123980551 | T | C | 0.355 | 0.008 | 0.001 | 1.20E-08 | 7.1E-05 | 32.45 |  |
| Left-arm FFM | rs12679359 | | 130724556 | T | G | 0.137 | -0.013 | 0.002 | 9.80E-13 | 1.1E-04 | 50.89 |  |
| Left-arm FFM | rs7843128 | | 22473465 | C | T | 0.357 | -0.008 | 0.001 | 6.30E-10 | 8.4E-05 | 38.22 |  |
| Left-arm FFM | rs4439140 | | 23113908 | A | G | 0.418 | -0.009 | 0.001 | 2.40E-11 | 9.8E-05 | 44.57 |  |
| Left-arm FFM | rs3925 | | 38281658 | A | G | 0.245 | -0.009 | 0.001 | 6.20E-09 | 7.4E-05 | 33.78 |  |
| Left-arm FFM | rs61729527 | | 77761919 | T | C | 0.052 | -0.021 | 0.003 | 3.40E-13 | 1.2E-04 | 52.99 | BMI |
| Left-arm FFM | rs16916881 | | 95566270 | A | C | 0.235 | -0.011 | 0.001 | 2.90E-13 | 1.2E-04 | 53.28 | BMI |
| Left-arm FFM | rs3808424 | | 116518399 | C | T | 0.218 | -0.015 | 0.002 | 4.30E-23 | 2.2E-04 | 97.95 | BMI |
| Left-arm FFM | rs1599473 | | 120475358 | T | G | 0.244 | -0.013 | 0.001 | 9.10E-18 | 1.6E-04 | 73.71 |  |
| Left-arm FFM | rs773145 | | 77668969 | C | G | 0.416 | -0.007 | 0.001 | 3.30E-08 | 6.7E-05 | 30.51 |  |
| Left-arm FFM | rs2482357 | | 94179978 | A | G | 0.432 | -0.010 | 0.001 | 2.20E-15 | 1.4E-04 | 62.89 | BMI |
| Left-arm FFM | rs10979612 | | 111687379 | C | T | 0.073 | 0.017 | 0.002 | 3.10E-12 | 1.1E-04 | 48.61 |  |
| Left-arm FFM | rs10985962 | | 126080226 | T | C | 0.633 | -0.008 | 0.001 | 4.40E-09 | 7.6E-05 | 34.44 |  |
| Left-arm FFM | rs4073153 | | 139259349 | G | A | 0.439 | 0.008 | 0.001 | 3.80E-10 | 8.6E-05 | 39.21 |  |
| Left-arm FFM | rs11794152 | | 23345347 | G | A | 0.415 | 0.009 | 0.001 | 2.50E-13 | 1.2E-04 | 53.60 |  |
| Left-arm FFM | rs1412234 | | 28410683 | C | T | 0.327 | 0.011 | 0.001 | 4.00E-16 | 1.5E-04 | 66.23 | BMI |
| Left-arm FFM | rs2380784 | | 33580929 | C | T | 0.062 | 0.015 | 0.003 | 9.60E-09 | 7.2E-05 | 32.92 |  |
| Left-arm FFM | rs13299559 | | 128041828 | T | C | 0.450 | -0.009 | 0.001 | 6.70E-13 | 1.1E-04 | 51.62 |  |
| Left-arm FFM | rs1927635 | | 16461905 | C | T | 0.354 | 0.010 | 0.001 | 4.90E-15 | 1.3E-04 | 61.28 |  |
| Left-arm FFM | rs11142700 | | 73757155 | C | T | 0.408 | -0.008 | 0.001 | 2.00E-10 | 8.9E-05 | 40.48 |  |
| Left-arm FFM | rs28457693 | | 98217348 | G | A | 0.107 | 0.023 | 0.002 | 1.50E-28 | 2.7E-04 | 122.84 |  |
| Left-arm FFM | rs1008158 | | 113828811 | G | A | 0.342 | 0.008 | 0.001 | 6.50E-09 | 7.4E-05 | 33.69 |  |
| Left-arm FFM | rs7033487 | | 119129257 | C | T | 0.198 | -0.020 | 0.002 | 4.90E-38 | 3.7E-04 | 166.24 |  |
| Left-arm FFM | rs111821073 | | 99084793 | T | C | 0.157 | 0.016 | 0.002 | 8.30E-21 | 1.9E-04 | 87.53 |  |
| Left-arm FFM | rs56141370 | | 127056213 | T | C | 0.115 | 0.016 | 0.002 | 3.60E-15 | 1.4E-04 | 61.91 |  |
| Left-arm FFM | rs1056747 | | 35690102 | G | A | 0.412 | -0.007 | 0.001 | 2.50E-08 | 6.8E-05 | 31.10 |  |
| Left-arm FFM | rs10746837 | | 90873653 | A | G | 0.581 | -0.011 | 0.001 | 1.30E-16 | 1.5E-04 | 68.40 |  |
| Left-arm FFM | rs532436 | | 136149830 | A | G | 0.185 | -0.010 | 0.002 | 1.10E-10 | 9.2E-05 | 41.60 |  |
| Left-arm FFM | rs10881583 | | 137267439 | C | T | 0.241 | -0.009 | 0.001 | 3.50E-10 | 8.7E-05 | 39.40 |  |
| Left-arm FFM | rs2165468 | | 3516105 | A | C | 0.114 | -0.012 | 0.002 | 4.20E-09 | 7.6E-05 | 34.51 |  |
| Left-arm FFM | rs75406471 | | 5257647 | A | G | 0.154 | 0.013 | 0.002 | 4.20E-14 | 1.3E-04 | 57.08 |  |
| Left-arm FFM | rs11014285 | | 25178864 | A | G | 0.165 | 0.017 | 0.002 | 6.20E-23 | 2.1E-04 | 97.22 |  |
| Left-arm FFM | rs7906411 | | 35047919 | C | T | 0.261 | -0.008 | 0.001 | 1.20E-08 | 7.2E-05 | 32.55 |  |
| Left-arm FFM | rs1937684 | | 53680085 | A | T | 0.654 | 0.008 | 0.001 | 4.50E-09 | 7.6E-05 | 34.39 | BMI |
| Left-arm FFM | rs7099526 | | 79657705 | G | C | 0.307 | -0.008 | 0.001 | 2.60E-09 | 7.8E-05 | 35.43 |  |
| Left-arm FFM | rs11245450 | | 126658075 | A | G | 0.422 | -0.011 | 0.001 | 3.50E-16 | 1.5E-04 | 66.51 | BMI |
| Left-arm FFM | rs73601548 | | 18549889 | T | C | 0.115 | 0.015 | 0.002 | 1.50E-13 | 1.2E-04 | 54.53 | BMI |
| Left-arm FFM | rs10995319 | | 52762887 | C | T | 0.234 | -0.008 | 0.001 | 2.10E-08 | 6.9E-05 | 31.44 |  |
| Left-arm FFM | rs7085079 | | 70198027 | G | A | 0.782 | 0.013 | 0.002 | 2.00E-17 | 1.6E-04 | 72.14 |  |
[truncated: 37,284 more chars]
